# The prediction of early preeclampsia: Results from a longitudinal proteomics study

**DOI:** 10.1371/journal.pone.0217273

**Published:** 2019-06-04

**Authors:** Adi L. Tarca, Roberto Romero, Neta Benshalom-Tirosh, Nandor Gabor Than, Dereje W. Gudicha, Bogdan Done, Percy Pacora, Tinnakorn Chaiworapongsa, Bogdan Panaitescu, Dan Tirosh, Nardhy Gomez-Lopez, Sorin Draghici, Sonia S. Hassan, Offer Erez

**Affiliations:** 1 Perinatology Research Branch, Division of Obstetrics and Maternal-Fetal Medicine, Division of Intramural Research, *Eunice Kennedy Shriver* National Institute of Child Health and Human Development, National Institutes of Health, U.S. Department of Health and Human Services (NICHD/NIH/DHHS), Bethesda, Maryland, and Detroit, Michigan, United States of America; 2 Department of Obstetrics and Gynecology, Wayne State University School of Medicine, Detroit, Michigan, United States of America; 3 Department of Computer Science, Wayne State University College of Engineering, Detroit, Michigan, United States of America; 4 Department of Obstetrics and Gynecology, University of Michigan, Ann Arbor, Michigan, United States of America; 5 Department of Epidemiology and Biostatistics, Michigan State University, East Lansing, Michigan, United States of America; 6 Center for Molecular Medicine and Genetics, Wayne State University, Detroit, Michigan, United States of America; 7 Systems Biology of Reproduction Research Group, Institute of Enzymology, Research Centre for Natural Sciences, Hungarian Academy of Sciences, Budapest, Hungary; 8 First Department of Pathology and Experimental Cancer Research, Semmelweis University, Budapest, Hungary; 9 Maternity Clinic, Kutvolgyi Clinical Block, Semmelweis University, Budapest, Hungary; 10 C.S. Mott Center for Human Growth and Development, Wayne State University, Detroit, Michigan, United States of America; 11 Department of Biochemistry, Microbiology, and Immunology, Wayne State University School of Medicine, Detroit, Michigan, United States of America; 12 Department of Physiology, Wayne State University School of Medicine, Detroit, Michigan, United States of America; 13 Maternity Department "D," Division of Obstetrics and Gynecology, Soroka University Medical Center, School of Medicine, Faculty of Health Sciences, Ben Gurion University of the Negev, Beer-Sheva, Israel; Universitat de Barcelona, SPAIN

## Abstract

**Objectives:**

To identify maternal plasma protein markers for early preeclampsia (delivery <34 weeks of gestation) and to determine whether the prediction performance is affected by disease severity and presence of placental lesions consistent with maternal vascular malperfusion (MVM) among cases.

**Study design:**

This longitudinal case-control study included 90 patients with a normal pregnancy and 33 patients with early preeclampsia. Two to six maternal plasma samples were collected throughout gestation from each woman. The abundance of 1,125 proteins was measured using high-affinity aptamer-based proteomic assays, and data were modeled using linear mixed-effects models. After data transformation into multiples of the mean values for gestational age, parsimonious linear discriminant analysis risk models were fit for each gestational-age interval (8–16, 16.1–22, 22.1–28, 28.1–32 weeks). Proteomic profiles of early preeclampsia cases were also compared to those of a combined set of controls and late preeclampsia cases (n = 76) reported previously. Prediction performance was estimated via bootstrap.

**Results:**

We found that 1) multi-protein models at 16.1–22 weeks of gestation predicted early preeclampsia with a sensitivity of 71% at a false-positive rate (FPR) of 10%. High abundance of matrix metalloproteinase-7 and glycoprotein IIbIIIa complex were the most reliable predictors at this gestational age; 2) at 22.1–28 weeks of gestation, lower abundance of placental growth factor (PlGF) and vascular endothelial growth factor A, isoform 121 (VEGF-121), as well as elevated sialic acid binding immunoglobulin-like lectin 6 (siglec-6) and activin-A, were the best predictors of the subsequent development of early preeclampsia (81% sensitivity, FPR = 10%); 3) at 28.1–32 weeks of gestation, the sensitivity of multi-protein models was 85% (FPR = 10%) with the best predictors being activated leukocyte cell adhesion molecule, siglec-6, and VEGF-121; 4) the increase in siglec-6, activin-A, and VEGF-121 at 22.1–28 weeks of gestation differentiated women who subsequently developed early preeclampsia from those who had a normal pregnancy or developed late preeclampsia (sensitivity 77%, FPR = 10%); 5) the sensitivity of risk models was higher for early preeclampsia with placental MVM lesions than for the entire early preeclampsia group (90% versus 71% at 16.1–22 weeks; 87% versus 81% at 22.1–28 weeks; and 90% versus 85% at 28.1–32 weeks, all FPR = 10%); and 6) the sensitivity of prediction models was higher for severe early preeclampsia than for the entire early preeclampsia group (84% versus 71% at 16.1–22 weeks).

**Conclusion:**

We have presented herein a catalogue of proteome changes in maternal plasma proteome that precede the diagnosis of preeclampsia and can distinguish among early and late phenotypes. The sensitivity of maternal plasma protein models for early preeclampsia is higher in women with underlying vascular placental disease and in those with a severe phenotype.

## Introduction

Preeclampsia is a major obstetrical syndrome [1–3], classified according to the time of its clinical manifestation as “early preeclampsia” if it occurs prior to 34 weeks of gestation and, otherwise, as “late preeclampsia” [4–10]. The 34-week cut-off is most commonly used [9,11,12] given the substantial decline in maternal [6,13–17] and neonatal [8,13,18–24] morbidity compared to later gestational ages.

Early preeclampsia accounts for approximately 10% of the cases [8], and its pathophysiology involves both maternal predisposing factors and disorders of deep placentation [25,26]. Indeed, in early preeclampsia, the frequency of placental vascular lesions consistent with maternal vascular malperfusion (MVM) is higher than in late preeclampsia [27–30], suggesting that the underlying pathological processes leading to this phenotype begin in the early stages of gestation and involve an angiogenic imbalance [11,31–37]. This finding has clinical implications given that patients identified to be at risk by the end of the first trimester can benefit from treatment [38–41].

Current prediction models for preeclampsia combine maternal risk factors, Doppler velocimetry of the uterine arteries, and maternal blood proteins [32,37,42–46]. Although the detection rate of these models [12,47–50] for the identification of patients at risk for early/preterm preeclampsia is sufficient to enable preventive strategies [40], the contribution of biochemical markers in these models is limited. Moreover, Doppler velocimetry required in the current screening models [47,51–57] to compensate for the sub-optimal prediction by biochemical markers may not be available in all clinical settings.

Therefore, we used a novel high-affinity aptamer-based proteomic platform to identify longitudinal changes in maternal plasma proteins that have the potential to improve prediction of early preeclampsia and to distinguish between the early and late phenotypes. We also investigated whether the predictive performance of protein markers is impacted by disease severity and the presence of placental lesions consistent with MVM among cases.

## Materials and methods

### Study design

A nested case-control study was conducted, including patients diagnosed with early preeclampsia (cases, n = 33) and those with a normal pregnancy (controls, n = 90). Women were enrolled as participants of a longitudinal cohort study conducted at the Center for Advanced Obstetrical Care and Research of the Perinatology Research Branch, NICHD/NIH/DHHS, the Detroit Medical Center, and Wayne State University. Women with a multiple gestation, severe chronic maternal morbidity (i.e., renal insufficiency, congestive heart disease, and/or chronic respiratory insufficiency), acute maternal morbidity (i.e., asthma exacerbation requiring systemic steroids and/or active hepatitis), or fetal chromosomal abnormalities and congenital anomalies were excluded from the study.

Plasma samples were collected at the time of each prenatal visit scheduled at four-week intervals from the first or early second trimester until delivery. All patients provided written informed consent prior to sample collection. The plasma proteome of each patient was profiled in two to six samples collected from each patient and included, for some of the cases, the sample collected after the diagnosis of early preeclampsia. Although data collected after diagnosis are displayed in longitudinal plots, all analyses reported herein were based only on samples collected prior to the diagnosis [median (interquartile range or IQR) of 3 (2–4) for cases and 2 (2–5) for controls].

The analysis presented in this manuscript is based on data and specimens collected under the protocol entitled “Biological Markers of Disease in the Prediction of Preterm Delivery, Preeclampsia and Intra-Uterine Growth Restriction: A Longitudinal Study.” The study was approved by the Institutional Review Boards of Wayne State University (WSU IRB#110605MP2F) and NICHD/NIH/DHHS (OH97-CH-N067).

### Clinical definitions

Preeclampsia was defined as new-onset hypertension that developed after 20 weeks of gestation (systolic or diastolic blood pressure ≥140 mm Hg and/or ≥90 mm Hg, respectively, measured on at least two occasions, 4 hours to 1 week apart) and proteinuria (≥300 mg in a 24-hour urine collection, or two random urine specimens obtained 4 hours to 1 week apart containing ≥1+ by dipstick or one dipstick demonstrating ≥2+ protein) [58].

Early preeclampsia was defined as preeclampsia diagnosed and delivered before 34 weeks of gestation, and late preeclampsia was defined as preeclampsia delivered at or after 34 weeks of gestation [4]. Severe preeclampsia was diagnosed as preeclampsia with systolic blood pressure ≥ 160 mmHg, or diastolic blood pressure ≥ 110 mmHg, platelet count < 100,000 per mm^3^, elevated liver enzymes, renal insufficiency, pulmonary edema or cyanosis, new-onset cerebral/visual disturbances, and/or right upper quadrant or epigastric pain [9,59].

### Histologic placental examination

Placentas were examined according to standardized protocols by perinatal pathologists blinded to clinical diagnoses and obstetrical outcomes, as previously described [60]. Placental lesions were diagnosed using criteria established by the Perinatal Section of the Society for Pediatric Pathology [61] and the terminology was updated to be consistent with that recommended by the Amsterdam Placental Workshop Group consensus statement [62]. The definitions of lesions consistent with MVM were previously described [63].

### Proteomics analysis

Maternal plasma protein abundance was determined by using the SOMAmer (Slow Off-rate Modified Aptamer) platform and reagents to profile 1,125 proteins [64,65]. Proteomics profiling services were provided by Somalogic, Inc. (Boulder, CO, USA). The plasma samples were diluted and then incubated with the respective SOMAmer mixes, and after following a suite of steps described elsewhere [64,65], the signal from the SOMAmer reagents was measured using microarrays.

### Statistical analysis

#### Demographics data analysis

Clinical characteristics of the patient population were summarized as median and IQRs for continuous variables or as percentages for categorical variables. The comparison of demographic variables between the groups was performed using the Fisher’s exact test for binary variables and the Wilcoxon rank-sum test for continuous variables.

#### Proteomic data transformation

The raw protein abundance data consisted of relative fluorescence units obtained from scanning the microarrays with a laser scanner. A sample-by-sample adjustment in the overall signal within a single plate (85 samples processed per plate/run) was performed in three steps: *Hybridization Control Normalization*, *Median Signal Normalization*, and *Calibration*, using the manufacturer’s protocol. Outlier values (larger than 2×the 98^th^ percentile of all samples) were set to 2×the 98^th^ percentile of all samples (*data thresholding*). Protein abundance was then log_2_ transformed to improve normality. Linear mixed-effects models with cubic splines (number of knots = 3) were used to model protein abundance in the control group as a function of gestational age using the *lme4* package [66] under the R statistical language and environment (www.r-project.org). Data for all samples were then expressed as multiple of the mean (MoM) values for the corresponding gestational age in the normal pregnancy group. Longitudinal protein abundance averages and confidence intervals in sub-groups (MVM vs non-MVM, and severe vs mild preeclampsia) were estimated using generalized additive mixed models implemented in the *mgcv* package and illustrated using *ggplot2* package in R.

#### Development of multi-marker prediction models

To develop proteomics prediction models based on protein abundance collected in each gestational-age interval (8–16, 16.1–22, 22.1–28, 28.1–32, 32.1–36 weeks) and, at the same time, to obtain unbiased prediction performance estimates on the available dataset, we implemented advances in predictive modeling with omics data [67–69]. Log_2_ MoM values for one protein at a time were used to fit a linear discriminant analysis (LDA) model and to compute by leave-one-out cross-validation, a classification performance measure for each protein. With leave-one-out cross-validation, data from one patient at a time is left out when fitting the LDA model, and then the fitted model is applied to the data of the subject left out. The resulting predictions were combined over all patients to calculate prediction performance. The performance measure considered was the partial area under the curve (pAUC) of the receiver operating characteristic (ROC) curve (false-positive rate [FPR] <50%). Proteins that failed to reach at least a 10% change in the average MoM value between the study groups were filtered out from the analysis. Next, LDA models were fit by using increasing sets of up to five of the top proteins ranked by the pAUC. To enforce model parsimony, the inclusion of each additional protein was conditioned on the increase of 0.01 units in the pAUC statistic.

To obtain an unbiased estimate of the prediction performance of multi-marker models, we used bootstrap (200 iterations). Each iteration involved the following steps: 1) draw a random sample with the replacement of 33 cases and 90 controls to create a *training set* and consider all patients not selected in the bootstrap sample as a *test set*; 2) apply all analytical steps involved in the prediction model development described above (including the selection of predictor proteins) for each gestational-age interval using the training set; 3) apply the resulting prediction model and determine its prediction performance on data from patients in the test set. The average performance over 200 test sets was reported as a robust estimate of the prediction performance. Alternatively, instead of creating training and test partitions via bootstrap, repeated (n = 67 times) 3-fold cross-validation was used to generate 201 training and test set pairs, while keeping all other parameters of the analysis the same as described above for bootstrap.

#### Differential abundance analysis

The classifier development pipeline described above identifies a parsimonious set of proteins that predict early preeclampsia, yet it will not necessarily retain all proteins showing evidence of differential abundance between groups. Therefore, a complementary analysis was performed to identify all proteins with significant differences in mean log_2_ MoM values between the cases and controls at each gestational-age interval. Linear models with coefficient significance evaluated via moderated t-tests were applied using the *limma* package [70] of Bioconductor [71]. Significance was inferred based on the FDR-adjusted p-value (q-value) <0.1 after adjusting for body mass index, smoking status, maternal age, and parity.

Both prediction model development and differential abundance analyses described above were also applied, including only controls and early preeclampsia cases i) with placental MVM lesions and ii) those with a severe phenotype.

#### Comparison between the proteomic profiles of early and late preeclampsia

To identify protein changes specific to early onset, but not late onset, of the disease, data from the early preeclampsia (n = 33) group were compared to a combined group that included both late preeclampsia cases (N = 76) [72] and normal pregnancies (n = 90).

#### Gene ontology and pathway analysis

Proteins were mapped to Entrez gene identifiers [73] based on Somalogic, Inc. annotation and, subsequently, to gene ontology [74]. Biological processes over-represented among the proteins that changed with early preeclampsia were identified using a Fisher’s exact test. Gene ontology terms with three or more hits and a q-value < 0.1 were considered significantly enriched. Identification of signaling pathways from the Kyoto Encyclopedia of Genes and Genomes (KEGG) pathway database [75] that were enriched in proteins with differential abundance was performed using a pathway impact analysis method previously described [76,77]. The analysis was conducted using the web-based implementation available in *iPathwayGuide* (http://www.advaitabio.com). All enrichment analyses used, as reference, the set of all 1,125 proteins that were profiled on the Somalogic platform.

## Results

In the early preeclampsia group, 33% (11/33) of the women delivered a small-for-gestational-age neonate, 73% (24/33) had placental lesions consistent with MVM and 70% (23/33) were severe cases. Cases were diagnosed from 24.6 to 33.4 weeks of gestation. Other characteristics of the study population classified by outcome and presence of placental MVM lesions are shown in **Table 1**.

**Table 1:** Demographic characteristics of the study population.

Characteristic	Normal pregnancy (n = 90)	Early PE (n = 33) With MVM (n = 24) Without MVM (n = 9)	
		With MVM (n = 24)	With MVM (n = 24) Without MVM (n = 9)
Gestational age at enrolment (weeks)	9.1 (8.0–10.1)	10.4 (8.3–15.2) [p = 0.024]	13.1 (8.4–14.6) [p = 0.042]
Gestational age at delivery (weeks)	39.4 (39.0–40.4)	31.2 (28.3–33.0) [p<0.001]	33.4 (32.1–33.6) [p<0.001]
Body mass index (kg/m^2^)	26.5 (22.8–33.2)	26.3 (20.5–30.6) [p = 0.27]	28.2 (22.3–32.9) [p = 0.62]
Maternal age (years)	24 (21.0–27.8)	22 (19.0–25.5) [p = 0.05]	24 (22.0–30.0) [p = 0.88]
Smoking status	18 (20%)	5 (20.83%) [p = 1]	5 (55.56%) [p = 0.03]
Nulliparity	26 (28.9%)	15 (62.5%) [p = 0.004]	1 (11.11%) [p = 0.44]

Data are presented as median (interquartile range) or number (percentage); P-values are given for the comparison to the normal pregnancy group. Early PE: early preeclampsia; MVM: maternal vascular malperfusion.

### Proteomic prediction models for early preeclampsia by gestational age at blood draw

**Fig 1:**
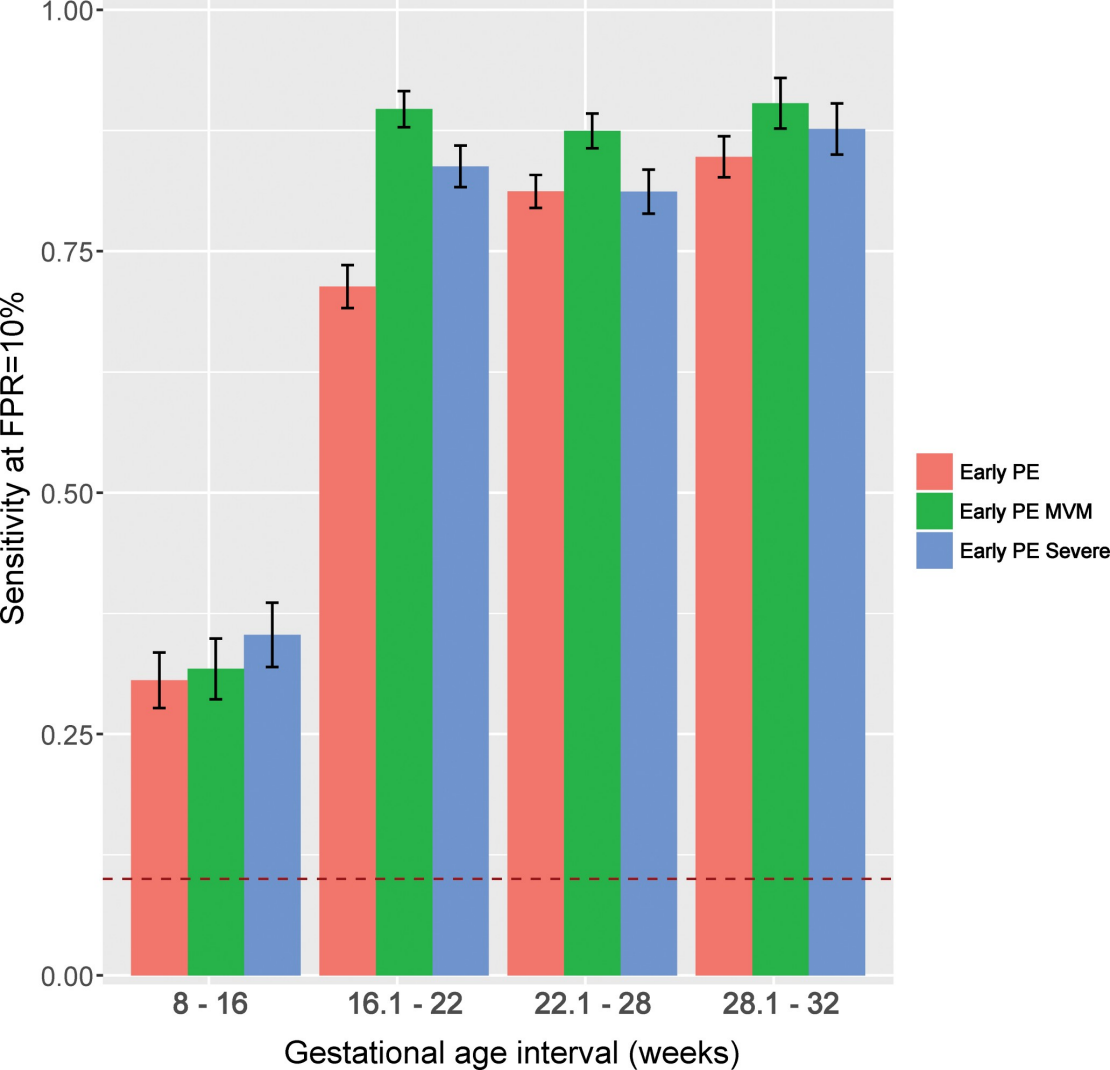
Sensitivity for early preeclampsia using multi-protein markers. Sensitivity (y-axis) at a 10% FPR are shown by gestational-age interval (x-axis) for early preeclampsia (PE), early PE with placental lesions consistent with MVM, and severe early PE. The vertical bars represent the average (with 95% confidence intervals) of sensitivity obtained from 200 bootstrap iterations. Early PE: early preeclampsia; FPR: false-positive rate; MVM: maternal vascular malperfusion.

The prediction performance indices of the multi-marker models involving up to five proteins were estimated by bootstrap and are illustrated in **Fig 1**and **Table 2**. **Fig 1**presents the sensitivity (10% FPR) of multi-marker models for early preeclampsia at each gestational-age interval.

**Table 2:** Summary of bootstrap results for prediction of early preeclampsia vs normal pregnancy.

Outcome	Sample GA	AUC	Sensitivity	Specificity	Predictor Symbols (% inclusion in best combination)
	(weeks)				
	8–16	0.64	0.31	0.90	MMP-7(42%), gpIIbIIIa(23%), HMG-1(10%), vWF(10%)
All	16.1–22	0.88	0.71	0.90	MMP-7(90%), gpIIbIIIa(18%), Soggy-1(10%),
Early PE	22.1–28	0.90	0.81	0.90	Siglec-6(58%), PlGF(52%), Activin A(25%), VEGF121(18%)
	28.1–32	0.94	0.85	0.90	ALCAM(38%), VEGF121(32%), Siglec-6(32%)
	8–16	0.63	0.32	0.90	MMP-7(33%), gpIIbIIIa(26%), ACE2(18%)
Early PE	16.1–22	0.96	0.90	0.90	MMP-7(99%),
MVM	22.1–28	0.95	0.87	0.92	Siglec-6(76%), PlGF(21%), Activin A(14%)
	28.1–32	0.95	0.90	0.90	Siglec-6(63%), VEGF121(33%), ALCAM(10%)
	8–16	0.67	0.35	0.90	MMP-7(44%); gpIIbIIIa(17%); Glutathione S-transferase Pi(12%); SMAC(10%); C4b(10%)
Early PE	16.1–22	0.94	0.84	0.90	MMP-7(97%); gpIIbIIIa(14%)
Severe	22.1–28	0.89	0.81	0.91	Siglec-6(68%); PlGF(34%); VEGF121(24%); Activin A(14%)
	28.1–32	0.95	0.88	0.90	Siglec-6(52%); VEGF121(26%); ALCAM(22%)

The number in parentheses following the name of each protein (column Predictor Symbols) represents the percentage of bootstrap iterations in which the protein was selected in the best model. Only proteins selected in 10% or more of the 200 bootstrap iterations are listed. ACE2: angiotensin converting enzyme 2; ALCAM: activated leukocyte cell adhesion molecule; AUC: area under the receiver operating characteristic curve; GA: gestational age; gpIIbIIIa: glycoprotein IIb/IIIa; HMG-1: high-mobility group protein 1; MMP: matrix metalloproteinase; early PE: early preeclampsia; MVM: maternal vascular malperfusion; PE: preeclampsia; PlGF: placental growth factor; Siglec-6: sialic acid binding immunoglobulin-like lectin; VEGF121: vascular endothelial growth factor A, isoform 121; vWF: von Willebrand factor; SMAC: Diablo homolog, mitochondrial; C4b: Complement C4b.

**Fig 2:**
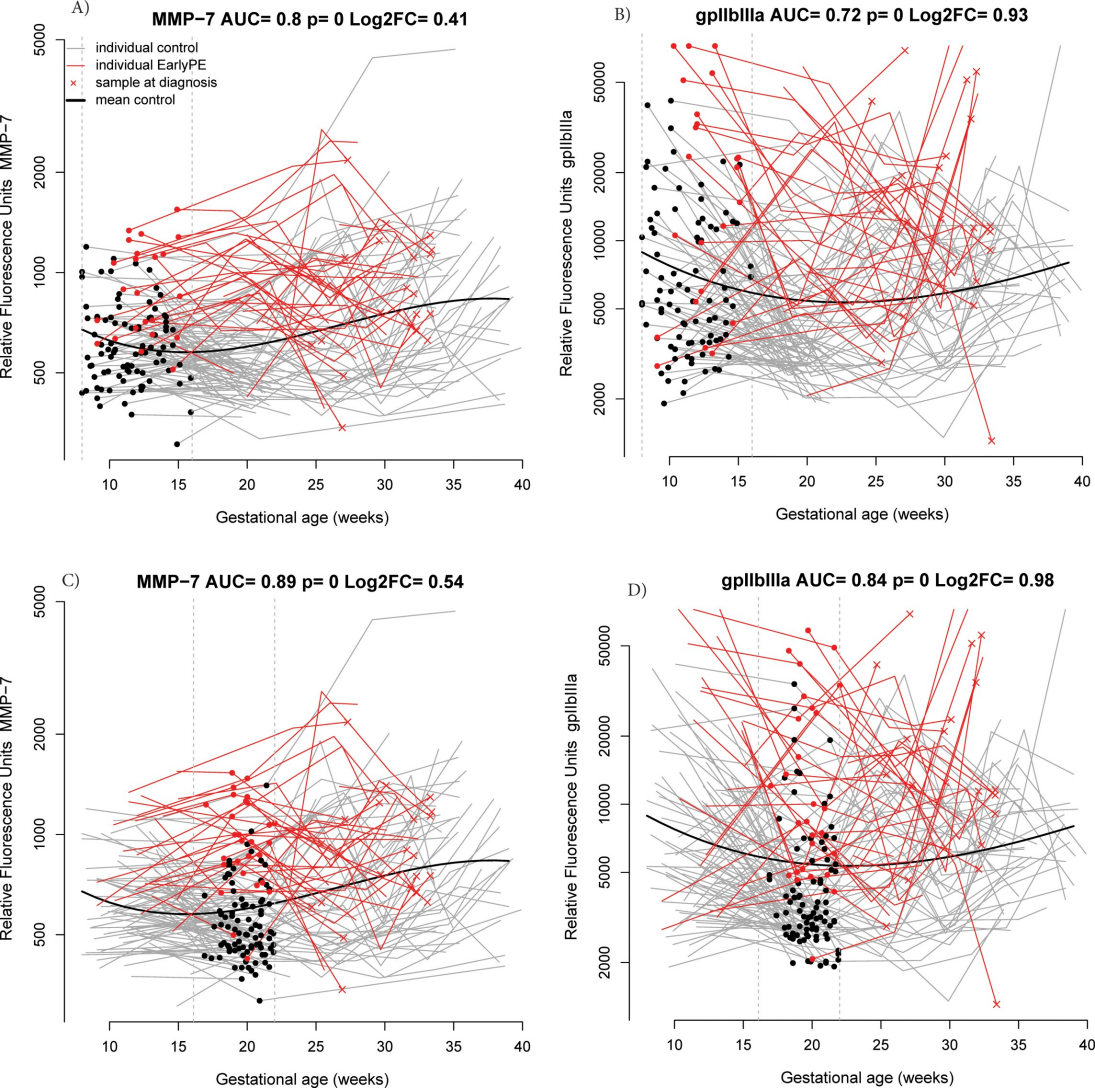
Longitudinal maternal plasma abundance of MMP-7 and gpIIbIIIA in normal pregnancy and early preeclampsia. Each line corresponds to a single patient (grey = normal pregnancy, red = early preeclampsia). Individual dots represent samples at 8–16 weeks (**A, B**) and 16.1–22 weeks (**C, D**) of gestation. Samples taken at the time of diagnosis with early preeclampsia are marked with an “x” and were not included in the analysis but only displayed. The thick black line represents the mean value in normal pregnancy. AUC: area under the receiver operating characteristic curve of the protein using data in the current interval; early PE: early preeclampsia; FC: fold change; gpIIbIIIa: glycoprotein IIb/IIIa; MMP-7: matrix metalloproteinase 7; MoM: multiples of the mean; p: the nominal significance p-value comparing mean MoM values between groups with a moderated t-test. Log_2_FC is the log (base 2) of the fold change between the cases and control groups, with negative values denoting lower MoM values in cases than in controls.

At 8–16 weeks of gestation, multi-marker proteomics models predicted early preeclampsia with 31% sensitivity (FPR = 10%), which was higher than that of PlGF alone (17%). The importance of individual proteins in the prediction models was evaluated by the percentage of the 200 bootstrap iterations in which they were included in the best LDA prediction model. Matrix metalloproteinase 7 (MMP-7) and glycoprotein IIbIIIa (gpIIbIIIa) were chosen in the best model in 42% and 23% of the iterations, respectively, while high-mobility group protein 1 (HMG-1) and von Willebrand factor were selected in 10% of the iterations (**Table 2**). Individual patient longitudinal profiles of MMP-7 and gpIIbIIIa protein abundance are presented in **Fig 2A and 2B**, respectively.

At 16.1–22 weeks of gestation, multi-marker prediction models identified women at risk to develop early preeclampsia with a sensitivity of 71% (FPR = 10%) which was again higher than the estimate for PlGF alone (18%). MMP-7, gpIIbIIIa, and Soggy-1 were selected in the best model 90%, 18%, and 10% of the time, respectively. The longitudinal profiles of MMP-7 and gpIIbIIIa, emphasizing the differences in the samples taken between 16.1 to 22 weeks of gestation, are presented in **Fig 2C and 2D**.

**Fig 3:**
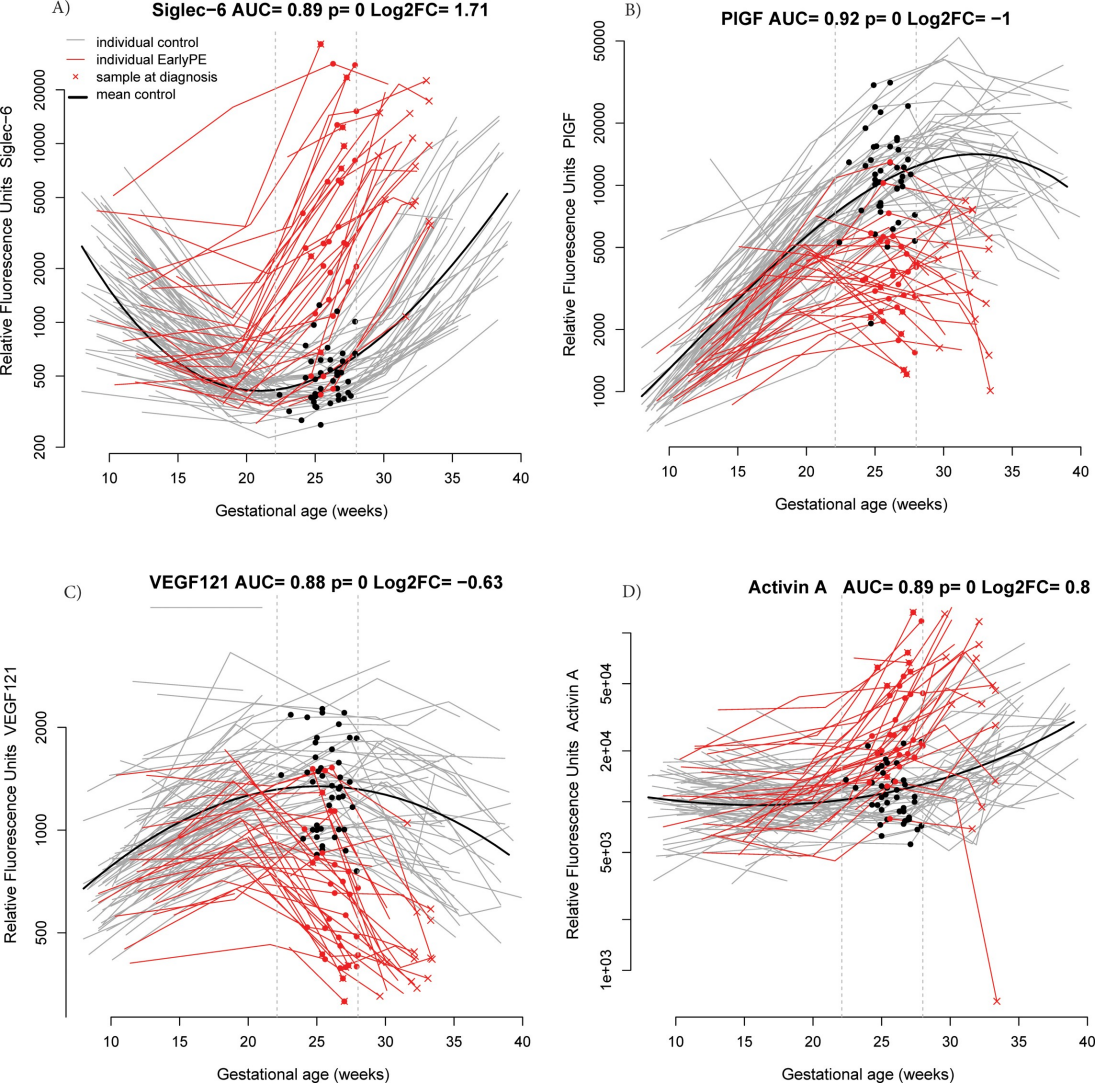
**Longitudinal maternal plasma abundance of siglec−6 (A), PlGF (B), VEGF121 (C), and activin-A (D) in normal pregnancy and early preeclampsia cases, highlighting differences at 22.1–28 weeks.** AUC: area under the receiver operating characteristic curve; early PE: early preeclampsia; FC: fold change; PlGF: placental growth factor; Siglec-6: sialic acid binding immunoglobulin-like lectin; VEGF121: vascular endothelial growth factor A, isoform 121.

At 22.1–28 weeks of gestation, the proteins most often selected in the best risk model for early preeclampsia out of 200 bootstrap iterations were sialic acid binding immunoglobulin-like lectin 6 (siglec-6) (58%), PlGF (52%), activin-A (25%), and VEGF121 (18%). Longitudinal profiles of these four proteins emphasizing the differences in the samples taken between 22.1 and 28 weeks of gestation are shown in **Fig 3**.

**Fig 4:**
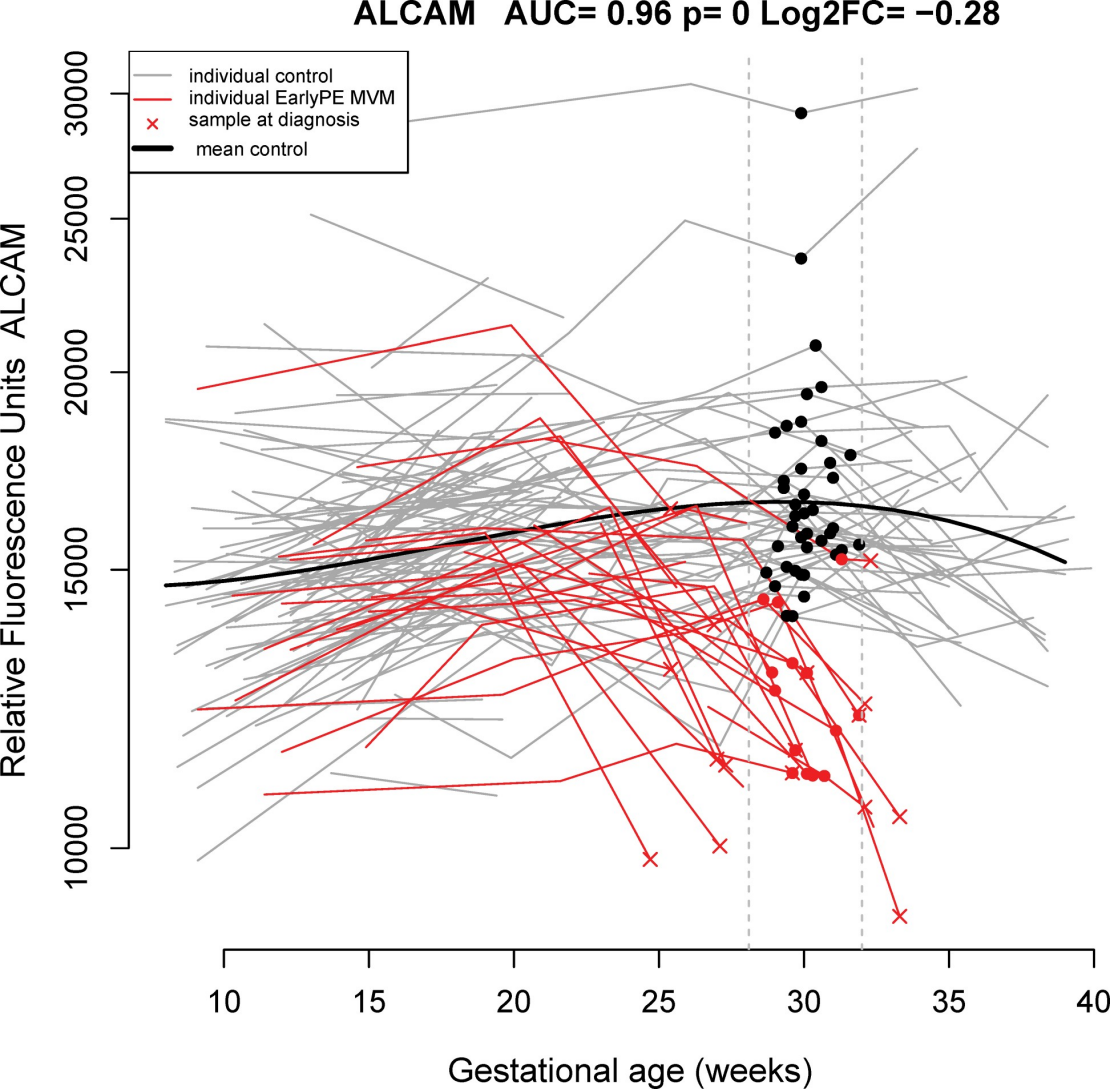
Longitudinal maternal plasma ALCAM abundance in normal pregnancy and early preeclampsia cases, highlighting differences at 28.1–32 weeks. ALCAM: activated leukocyte cell adhesion molecule; AUC: area under the receiver operating characteristic curve; early PE: early preeclampsia; FC: fold change; MVM: maternal vascular malperfusion.

At 28.1–32 weeks of gestation, the bootstrap-estimated sensitivity of multi-marker risk models was 85% (FPR = 10%), with activated leukocyte cell-adhesion molecule (ALCAM), siglec-6, and VEGF121 being the most frequently selected markers (38%, 32%, and 32% of the bootstrap iterations, respectively). The longitudinal profiles of ALCAM are depicted in **Fig 4**.

Of note, prediction performance estimates for early preeclampsia were slightly higher when estimated by repeated cross-validation (**S1 Table**) than by bootstrap (**Table 2**), yet the variance of the estimates with the former method was somewhat higher (data not shown). The most predictive proteins retained in the prediction models were similar between the two approaches (see **Tables 2**and **S1**).

### Prediction of early preeclampsia according to the presence of placental lesions consistent with maternal vascular malperfusion

To determine whether the sub-classification of early preeclampsia cases by placental lesions can lead to different protein markers and/or better prediction performance, a secondary analysis was performed that included the control group and only cases with placental lesions consistent with MVM. Bootstrap-based sensitivity estimates (at a fixed FPR of 10%) were higher for cases with MVM compared to those for the overall early preeclampsia group (16.1–22 weeks: 90% versus 71%; 22.1–28 weeks: 87% versus 81%; and 28.1–32 weeks: 90% versus 85%) (see bars in **Fig 1**and **Table 2**).

**Fig 5:**
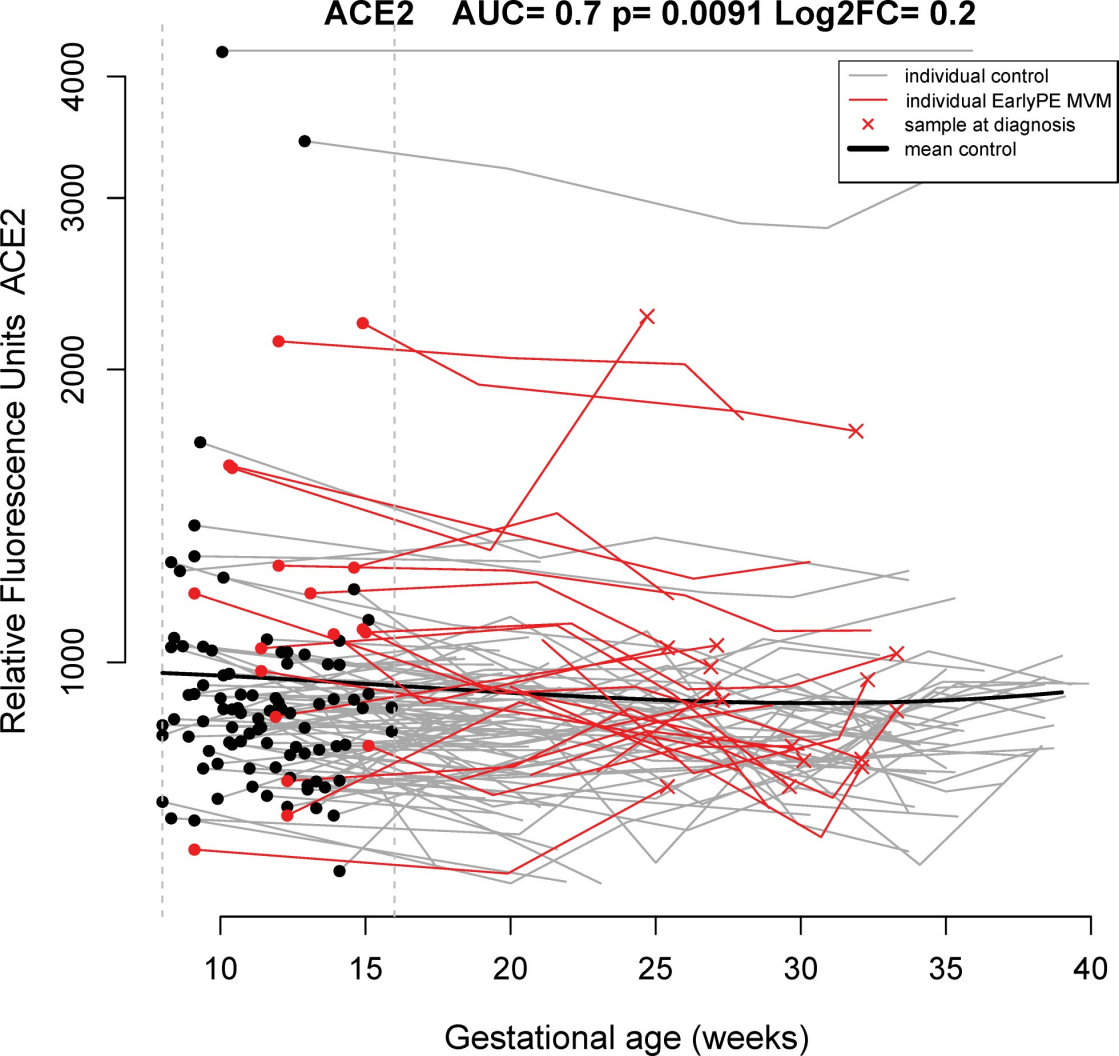
Longitudinal maternal plasma ACE2 abundance in normal pregnancy and early preeclampsia cases, highlighting differences at 8–16 weeks of gestation. See Fig 2 legend for more details. ACE2: angiotensin-converting enzyme 2; AUC: area under the receiver operating characteristic curve; early PE: early preeclampsia; FC: fold change; MVM: maternal vascular malperfusion.

In addition to a higher sensitivity for cases with placental MVM lesions compared to the overall early preeclampsia group, differences in the sets of best predictors also emerged at particular intervals of gestation (**Table 2**). For example, angiotensin-converting enzyme 2 (ACE2) at 8–16 weeks (see raw data in **Fig 5**) and siglec-6 at 22.1–32 weeks of gestation were more frequently selected as the best markers for early preeclampsia with MVM lesions than for overall early preeclampsia (see **Table 2**).

### Prediction of early preeclampsia according to disease severity

When only severe early preeclampsia cases were included in the analysis and compared to normal pregnancy cases, the sensitivity of analysis (10% FPR) was significantly higher than for overall early preeclampsia (90% vs 71%) in the 16.1–22 week interval. At this gestational-age interval, but unlike early preeclampsia with MVM that was predicted mostly by an increase in MMP-7, the prediction for severe early preeclampsia also involved the increase in gpIIbIIIa for 14% of the models trained on bootstrap samples of the original dataset. Other differences in the set of best predictors for severe early preeclampsia compared to overall early preeclampsia were noted in the 8–16 weeks gestational-age interval (see **Table 2**).

### Proteomic markers that differentiate between early and late preeclampsia

Discrimination between early preeclampsia and both normal pregnancy and late preeclampsia was rather low in the 8-16-week and 16.1-22-week intervals (21% and 31% sensitivity, respectively, FPR = 10%) and involved different sets of proteins than those found when the comparison was only against the normal pregnancy group (**Table 3**). However, later in gestation, the sensitivity of multi-marker models to discriminate between early preeclampsia and both the controls and late preeclampsia increased to 77% and 82% at 16.1-22-week and 22.1-28-week intervals, respectively (FPR = 10%).

**Fig 6:**
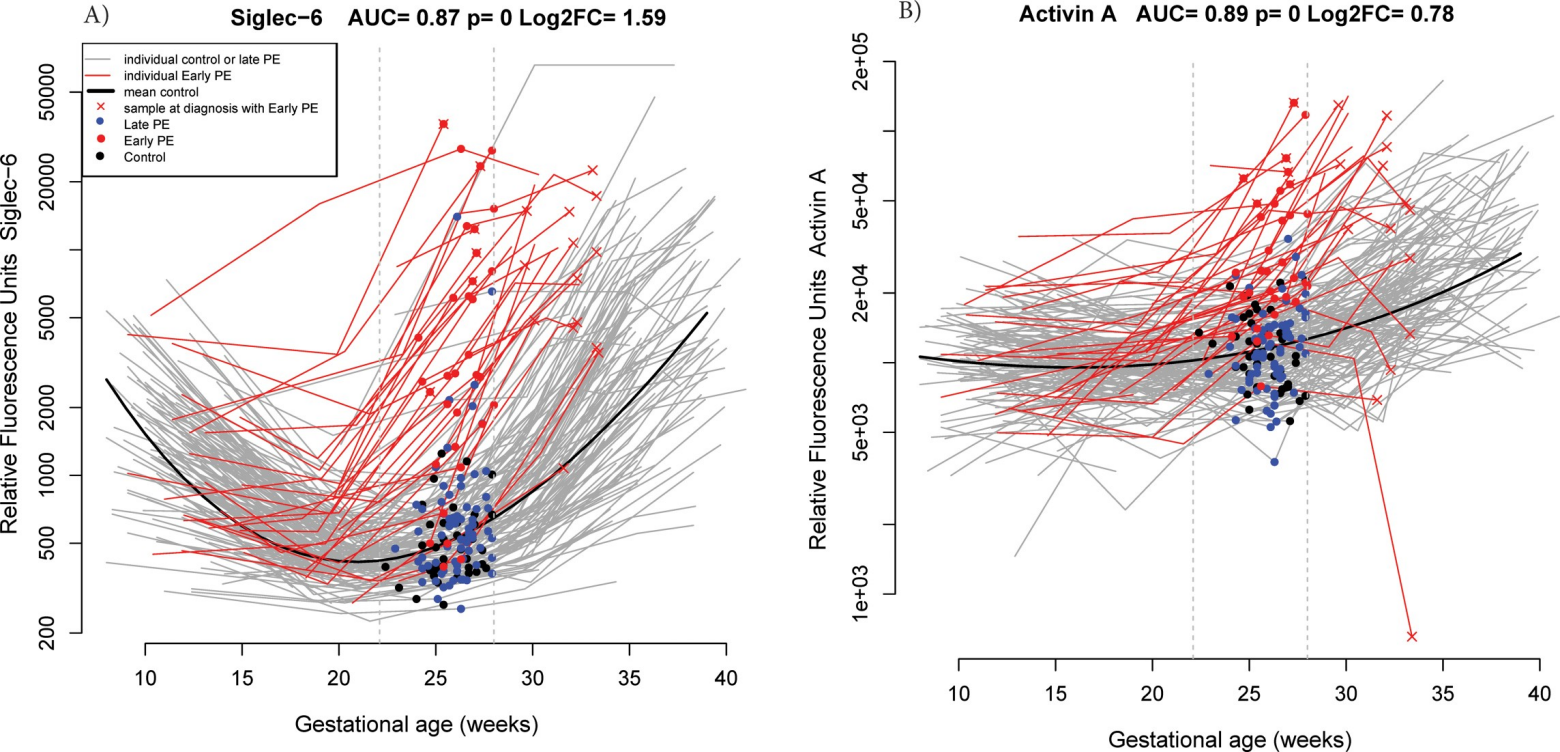
**Longitudinal maternal plasma abundance of siglec-6 (A) and activin-A (B) in normal pregnancy and early preeclampsia, highlighting differences at 22.1–28 weeks.** Blue dots correspond to samples taken from late preeclampsia cases. AUC: area under the receiver operating characteristic curve; early PE: early preeclampsia; FC: fold change; late PE: late preeclampsia; Siglec-6: sialic acid binding immunoglobulin-like lectin.

Of note, discriminating early preeclampsia from both normal pregnancy and late preeclampsia cases involved more stringent cut-offs for the same proteins (see **Fig 6**) and also new proteins such as ficolin 2 (FCN2) (see **Table 3**).

**Table 3:** Summary of bootstrap results for prediction of early preeclampsia versus normal pregnancy and late preeclampsia.

Outcome	Sample GA (weeks)	AUC	Sensitivity	Specificity	Predictor Symbols (% inclusion in best combination)
Early PE	8–16	0.55	0.21	0.90	gpIIbIIIa(34%)
Early PE	16.1–22	0.65	0.31	0.90	Soggy-1(26%); IMDH2(20%); Siglec-6(14%); PKC-D(12%); MMP-12(10%); RBP(10%)
Early PE	22.1–28	0.89	0.77	0.90	Siglec-6(72%); Activin A(63%); VEGF121(34%)
Early PE	28.1–32	0.93	0.82	0.90	Siglec-6(72%); ALCAM(15%); FCN2(14%); VEGF121(12%)

ALCAM: activated leukocyte cell adhesion molecule; AUC: area under the receiver operating characteristic curve; early PE: early preeclampsia; *FCN2*: ficolin 2; GA: gestational age; gpIIbIIIa: glycoprotein IIb/IIIa; IMDH2: inosine-5'-monophosphate dehydrogenase (IMDH2); MMP: matrix metalloproteinase; PKC-D: protein kinase C delta type; RBP: retinol binding protein; Siglec-6: sialic acid binding immunoglobulin-like lectin; VEGF121: vascular endothelial growth factor A, isoform 121. Only proteins selected in 10% or more of the 200 bootstrap iterations are listed.

### Differential protein abundance summary

In addition to the proteins included in the parsimonious models predictive of early preeclampsia at different gestational-age intervals (**Table 2**), other proteins (total, n = 175) had a significant differential abundance (after adjustment for body mass index, smoking status, maternal age, and parity) in at least one gestational-age interval (q-value < 0.1).

**Fig 7:**
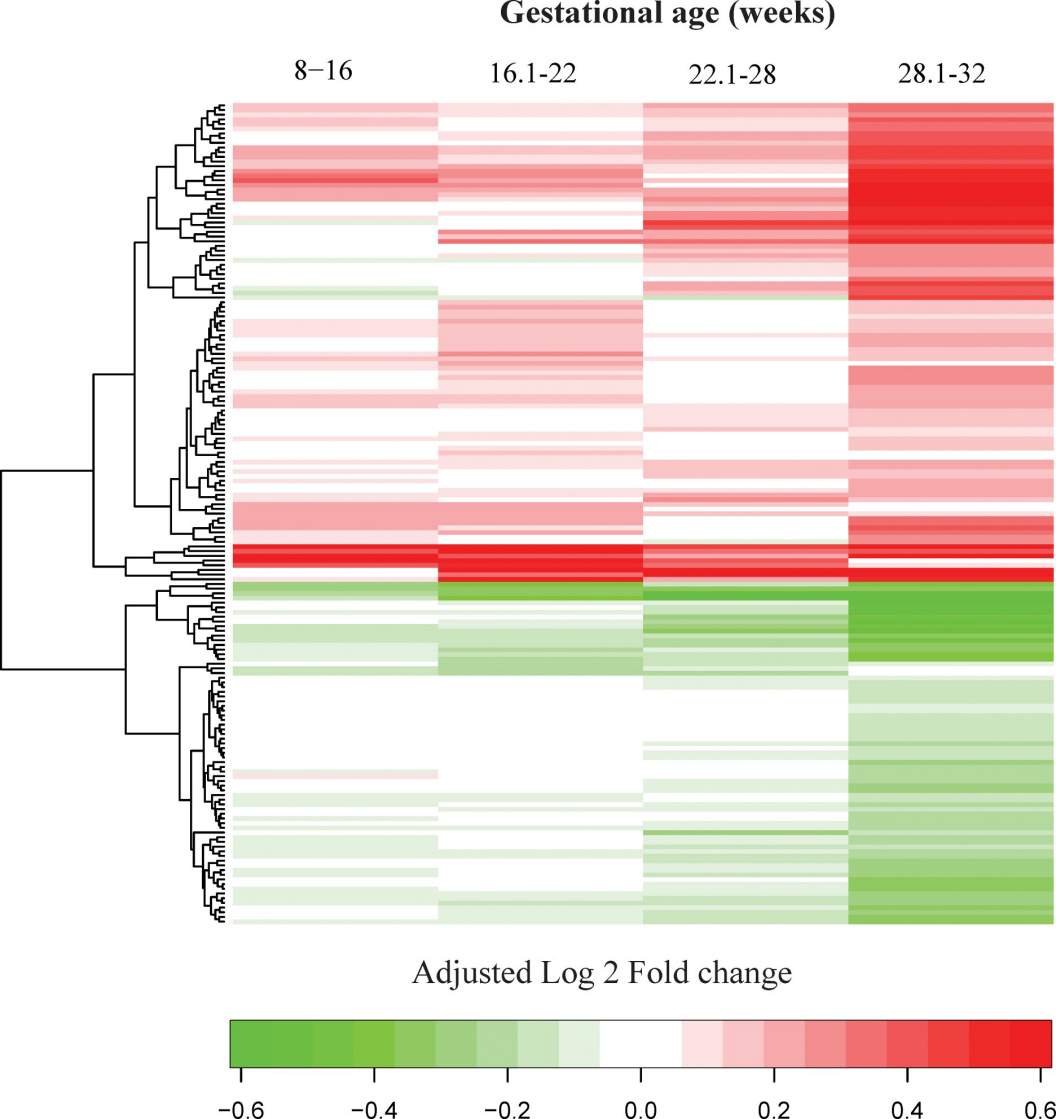
A summary of differential protein abundance between early preeclampsia and normal pregnancy throughout gestation. The values shown using a color scheme represent the log_2_ fold change in MoM values between the cases and controls (green = lower, red = higher mean MoM in cases versus controls). Fold changes >1.5 (absolute log_2_ fold change >0.58) were reset to 1.5 to enhance visualization of the data.

**S2 Table** presents the linear fold changes of MoM values between the early preeclampsia and normal pregnancy groups as well as the nominal and FDR-adjusted p-values (q-values) for each gestational-age interval. Additionally, the heatmap presented in **Fig 7**summarizes the differential abundance patterns across all gestational-age intervals included in this study. There were 2, 37, 20, and 153 proteins associated with early preeclampsia at 8–16, 16.1–22, 22.1–28, and 28.1–32 weeks of gestation, respectively.

MMP-7 was elevated in three of the four gestational-age intervals. IL-1 R4 (interleukin-1 receptor-like 1), siglec-6, and activin-A were elevated while FCN2, MMP-12, VEGF121, and PlGF were lower in all three intervals from 16.1 weeks of gestation onward. Differential abundance analyses were also summarized for early preeclampsia with MVM (**S3 Table** and **S1 Fig**), as well as for severe early preeclampsia (**S4 Table** and **S1 Fig**) compared to normal pregnancy.

### Biological processes and pathways perturbed in early preeclampsia during gestation

Gene ontology analysis of the proteins that changed significantly between patients with a normal pregnancy and those with early preeclampsia was performed for each gestational-age interval. At 16.1–22 weeks of gestation, there were 6; at 22.1–28 weeks, there were 7; and at 28.1–32 weeks, there were 30 biological processes significantly associated with early preeclampsia (**Table 4**). Biological processes associated with protein changes in at least one gestational age interval included *cell adhesion*, *response to hypoxia*, *positive regulation of endothelial cell proliferation*, *extracellular matrix disassembly*, and *vascular endothelial growth factor receptor signaling pathway* (all: q < 0.1) (**Table 4**).

**Table 4:** Biological processes enriched in proteins with a differential abundance between early preeclampsia and normal pregnancy.

Interval	Name	N	OR	p	q
	xenobiotic metabolic process	3	47.1	0.000	0.008
	negative chemotaxis	3	31.5	0.001	0.008
**16.1–22**	small molecule metabolic process	10	3.1	0.006	0.0485
**weeks**	regulation of transcription from RNA polymerase II promoter	3	9.5	0.007	0.0485
	integrin-mediated signaling pathway	3	7.3	0.014	0.071
	extracellular matrix disassembly	5	3.7	0.019	0.0838
	positive regulation of endothelial cell proliferation	4	11.7	0.001	0.0128
	cellular calcium ion homeostasis	3	7.0	0.014	0.0866
	response to hypoxia	3	5.1	0.031	0.0866
**22.1–28**	cell adhesion	5	3.3	0.033	0.0866
**weeks**	response to drug	4	3.7	0.036	0.0866
	positive regulation of angiogenesis	3	4.6	0.040	0.0866
	extracellular matrix disassembly	3	4.1	0.053	0.0976
	blood coagulation	36	2.7	0.000	0.0042
	platelet degranulation	18	3.9	0.000	0.0045
	blood coagulation, intrinsic pathway	8	8.9	0.000	0.0123
	sprouting angiogenesis	6	13.1	0.000	0.0218
	platelet activation	22	2.5	0.001	0.036
	vascular endothelial growth factor signaling pathway	4	25.9	0.001	0.063
	positive regulation of endothelial cell migration	7	5.8	0.002	0.0683
	response to cold	3	Inf	0.002	0.0703
	plasminogen activation	3	Inf	0.002	0.0703
	nervous system development	12	3.1	0.003	0.071
	blood circulation	5	8.1	0.003	0.071
	negative regulation of cell-substrate adhesion	4	13.0	0.004	0.071
	positive regulation of macrophage activation	4	13.0	0.004	0.071
**28.1–32**	positive regulation of synapse assembly	4	13.0	0.004	0.071
**weeks**	liver development	6	5.6	0.004	0.071
	fibrinolysis	7	4.6	0.004	0.071
	response to hypoxia	12	2.9	0.005	0.071
	hematopoietic progenitor cell differentiation	4	8.6	0.008	0.086
	response to vitamin D	4	8.6	0.008	0.086
	negative regulation of fat cell differentiation	4	8.6	0.008	0.086
	positive regulation of acute inflammatory response	3	19.3	0.009	0.086
	cell-substrate junction assembly	3	19.3	0.009	0.086
	negative regulation of ossification	3	19.3	0.009	0.086
	negative regulation of B cell differentiation	3	19.3	0.009	0.086
	cellular response to follicle-stimulating hormone stimulus	3	19.3	0.009	0.086
	negative regulation of angiogenesis	7	3.8	0.009	0.086
	negative regulation of cysteine-type endopeptidase activity involved in apoptotic process	7	3.8	0.009	0.086
	positive regulation of neuron differentiation	6	4.4	0.010	0.0895
	positive regulation of blood vessel endothelial cell migration	5	5.4	0.010	0.0895
	positive regulation of MAPK cascade	9	3.0	0.011	0.0953

ID: Gene Ontology (GO) biological processes identifier; N: number of significant proteins assigned to the GO term; OR: odds ratio for enrichment; p: p-value; q: false discovery rate-adjusted p-value.

No signaling pathways documented in the KEGG database [75] were found to be perturbed given the differential protein abundance observed in each interval of gestation.

## Discussion

### Principal findings of the study

The principal findings of the study are as follows: 1) At 16.1–22 weeks of gestation, multi-protein models predicted early preeclampsia with a sensitivity of 71% (FPR = 10%). The most reliable predictors in this interval were an elevated MMP-7 and gpIIbIIIa complex; 2) the best predictors of the subsequent development of early preeclampsia at 22.1–28 weeks of gestation were lower PlGF and VEGF121 as well as elevated siglec-6 and activin-A (81% sensitivity, FPR = 10%); 3) at 28.1–32 weeks of gestation, the sensitivity of multi-protein models was 85% (FPR = 10%) with the most reliable predictors being ALCAM, siglec-6, and VEGF121; 4) the increase in siglec-6, activin-A, and VEGF121 at 22.1–28 weeks of gestation differentiated women who subsequently developed early preeclampsia from those who had a normal pregnancy or late preeclampsia (sensitivity 77%, FPR = 10%); 5) the sensitivity of proteomic models for early preeclampsia in women with placental lesions consistent with MVM was higher than that of the models reported for the overall early preeclampsia group from 16.1 weeks of gestation onward; and 6) the sensitivity of prediction models was higher for severe early preeclampsia than for the entire early preeclampsia group (84% versus 71% at 16.1–22 weeks).

Of note, differential protein abundance results and, hence, downstream enrichment analyses are expected to vary among the different intervals of gestation due to several factors, such as: 1) differences in the sets of patients that contributed one sample in each interval, due to sample availability or to exclusion from analysis of samples at/or past the gestational age at diagnosis (see Methods); 2) differences in the magnitude of underlying disease-specific maternal plasma protein changes with preeclampsia; and 3) differences in the level of noise in the data, contributing non-biological variability.

### Proteomics prediction models for the identification of patients with preeclampsia

Biomarkers for the identification of patients at risk for obstetrical syndromes such as small-for-gestational-age neonates [34,78–82], spontaneous preterm birth [83–94], fetal death [95–105], and preeclampsia [12,47,49,50,56,72,106–113] have been proposed. For preeclampsia, prediction models have evolved from ones that used maternal background characteristics alone (e.g., obstetrical history, chronic hypertension, familial history of preeclampsia, obesity) [114,115] to those that combine maternal demographic characteristics, obstetrical history [116,117], mean blood pressure [118], uterine artery Doppler studies [52,54,119], and molecular biomarkers [56,120–122] (e.g., PAPP-A [88,123–125] and inhibin-A [124,126–128]). Some of the most predictive biochemical markers include angiogenic and anti-angiogenic factors [33,129–134] (PlGF [34,135–137], sVEGFR-1[138–142], and endoglin [143–148]), or their ratios [34,129,149–155]. A limitation of current screening methods for preeclampsia is the requirement of Doppler velocimetry, which is not readily available in middle- and low-resource populations. The detection rate for early preeclampsia drops to 77% and 57% at FPRs of 10% and 5%, respectively, in the absence of Doppler information [156]. Therefore, there would still be a benefit in developing accurate prediction models based solely on molecular information.

Discovery of molecular markers for obstetrical complications is often undertaken using “omics” technologies [157–165]: genomics [166,167], transcriptomics [168–175], proteomics [72,165,176–187], metabolomics [188–192], peptidomics [193–198], and lipidomics [199,200]. In particular, maternal proteomic profiles in preeclampsia were reported in maternal serum/plasma [175–177,180,201–210], urine [211–213], amniotic fluid [214,215], and the placenta [179,182,216–228]. However, most maternal plasma/serum proteomics studies to date did not involve samples collected longitudinally to determine how early molecular markers change their profiles prior to the disease onset and whether these changes are consistent throughout pregnancy, or the studies involved a small sample size.

The current study is one of the largest in this field and uses a new proteomics technology based on aptamers that allows the measurement of 1,125 proteins. Using this platform (Somalogic, Inc.), we and other investigators reported the stereotypic longitudinal changes of the maternal plasma proteome in normal pregnancy [229,230] and late preeclampsia [72]. Our current report observing that an increased maternal plasma abundance of MMP-7 and gpIIbIIIa is predictive of early preeclampsia during the first half of pregnancy is novel.

### Increased maternal plasma MMP-7 precedes diagnosis of preeclampsia

A possible explanation for the increased maternal plasma MMP-7 in preeclampsia is that it is a marker of abnormal placentation. MMP-7 is expressed in the decidua and trophoblast [231,232] and has been proposed to play a role in the process of transformation of the spiral arteries [233,234]. There is also histological evidence to support the involvement of MMP-7 in the processes associated with the development of preeclampsia [231] and early preeclampsia [233]. Additionally, MMP-7 can act as a sheddase for syndecan-1 [235,236], a major transmembrane heparan sulfate proteoglycan expressed on the surface (glycocalyx) of epithelial, endothelial, and syncytiotrophoblast cells [237–239], which are implicated in the pathophysiology of preeclampsia [240–243]. MMP-7 may also be involved in processes leading to the formation of atherosclerotic plaques [244] that show characteristics (e.g., lipid-laden macrophages) similar to acute atherosis of the spiral arteries associated with preeclampsia [245,246]. Of note in our previous study that used the same proteomics platform, MMP-7 was found to be a sensitive biomarker during the first half of pregnancy for the detection of patients who subsequently developed late preeclampsia [72]; herein, we showed that is also the case for early preeclampsia.

### The role of glycoprotein IIbIIIa in early preeclampsia

To our knowledge, this is the first study to report that changes in the abundance of gpIIbIIIa in the maternal plasma are predictive of subsequent development of early preeclampsia. In this patient population, at 8–16 weeks of gestation, gpIIbIIIa performed better than PlGF (currently used to screen for preeclampsia) [48,50,51,137] for the detection of patients who subsequently developed early preeclampsia when profiled with the Somalogic platform (AUC = 0.60 for PlGF and 0.72 for gpIIbIIIa, see **Table 2**and **Fig 2B**).

Glycoprotein IIb-IIIa is a membrane glycoprotein [247], the most common platelet receptor [247,248]. After a conformational change occurring during platelet activation [249], it interacts with ligands (e.g., von Willebrand factor and fibrinogen) to play a critical role in platelet aggregation and the cross-linkage of platelets into a hemostatic plug or thrombus [250–253]. Aspirin inhibits the expression of gpIIbIIIa by platelets [254]. This fact is important given that aspirin is currently recommended by regulatory bodies in the United States for the prevention of preeclampsia [255–257]; moreover, this medication has recently been reported to reduce the rate of preterm preeclampsia by 62% [40]. Our findings suggest that gpIIbIIIa inhibitors could be further developed for the prevention of early preeclampsia.

### Presence of placental lesions of maternal vascular malperfusion and disease severity increases the sensitivity of proteomic models for early preeclampsia

The sensitivity of the proteomic models at each gestational-age interval from 16.1 weeks onward was higher for cases that had placental lesions consistent with MVM than for the overall group of women with early preeclampsia and even compared to those with severe early preeclampsia. Maternal vascular malperfusion is a prevalent placental histologic finding in patients with early preeclampsia [28], and 73% (24/33) of cases in the current study had these lesions. These results further support a previous observation that the prediction performance of angiogenic index-1 (PlGF/sVEGFR-1) for preterm delivery (<34 weeks) is higher for women with these types of placental lesions [63].

Of interest, even when only patients with lesions consistent with MVM were compared to those with a normal pregnancy, proteins of placental origin (e.g., PlGF and siglec-6) were still the most predictive of early preeclampsia, but only after 22 weeks of gestation. This finding is consistent with our earlier study in late preeclampsia [72] and with previous longitudinal studies of angiogenic and anti-angiogenic factors [35,46,151]. Moreover, the data presented herein also support our previous systems biology study in early preeclampsia showing that siglec-6 expression in the placenta increased in the second half of pregnancy due to a hypoxic-ischemic trophoblastic response to placental malperfusion [258].

### Clinical implications

The current study demonstrates the potential of maternal plasma protein changes to identify women at risk of early preeclampsia based on a single blood test. The use of disease-risk models based solely on proteomic markers would be similar to first- and second-trimester aneuploidy tests [259–262]. Such an approach can be implemented in various clinical settings, especially in low-resource areas, where Doppler velocimetry of the uterine arteries is not readily available. Moreover, the proteomics biomarkers identified in this study may assist in the introduction of novel therapeutic agents (e.g., gpIIbIIIa inhibitors) for the prevention of early preeclampsia.

### Strengths and limitations of the study

The major strengths of this study are its longitudinal design, the number of patients and their stratification according to placental histology, and the large number of proteins tested. In addition, best practices in terms of model development and validation were based on our award-winning classifier development pipeline [67–69]. A limitation of this study is the fact that the aptamer-based assays did not include internal standards to generate protein concentrations (as opposed to fluorescence-based abundance); hence, further studies would be needed to generate protein concentration cut-offs. Additionally, the majority of the patients included in this study were of African-American lineage, and the generalization of findings to other ethnic groups needs to be further examined. Lastly, for three of the 33 early preeclampsia cases, the information regarding 24-hour proteinuria was not available; hence, we were reliant on dipstick evaluation.

## Conclusions

Aptamer-based proteomic profiling of maternal plasma identified novel as well as previously known markers for early preeclampsia. At 16.1–22 weeks of gestation, more than two-thirds of patients who subsequently develop early preeclampsia can be identified by an elevated MMP-7 and gpIIbIIIa in maternal plasma (10% FPR). High abundance of siglec-6, VEGF121, and activin-A observed in the maternal circulation at 22.1–28 weeks of gestation was more specific to early rather than late preeclampsia. Proteomic markers were more sensitive for early preeclampsia cases with placental lesions consistent with MVM as well as those with a severe phenotype.

## Supporting information

S1 Table Summary of cross-validation results for prediction of early preeclampsia vs normal pregnancy. The number in parentheses following the name of each protein (column Predictor Symbols) represents the percentage of folds in which the protein was selected in the best model. Only proteins selected in 10% or more of the 3x67 = 201 folds are listed. ACE2: angiotensin converting enzyme 2; ALCAM: activated leukocyte cell adhesion molecule; AUC: area under the receiver operating characteristic curve; GA: gestational age; gpIIbIIIa: glycoprotein IIb/IIIa; HMG-1: high-mobility group protein 1; MMP: matrix metalloproteinase; early PE: early preeclampsia; MVM: maternal vascular malperfusion; PE: preeclampsia; PlGF: placental growth factor; Siglec-6: sialic acid binding immunoglobulin-like lectin; VEGF121: vascular endothelial growth factor A, isoform 121; vWF: von Willebrand factor.
(XLSX)

S2 Table Summary of the differential abundance analysis between early preeclampsia and normal pregnancy in four intervals of gestation. List of 175 proteins with significantly different abundance between early preeclampsia and normal pregnancy (q < 0.1) in at least one interval, after adjustment for body mass index, maternal age, parity and smoking status. FC: linear fold change, with negative values denoting lower levels while positive values denote higher levels in cases than in controls.
(XLSX)

S3 Table Summary of the differential abundance analysis between early preeclampsia and normal pregnancy in four intervals of gestation. List of 76 proteins with significantly different abundance between early preeclampsia with MVM and normal pregnancy (q < 0.1) in at least one interval, after adjustment for body mass index, maternal age, parity and smoking status. FC: linear fold change, with negative values denoting lower levels while positive values denote higher levels in cases than in controls.
(XLSX)

S4 Table Summary of the differential abundance analysis between early preeclampsia and normal pregnancy in four intervals of gestation. List of 130 proteins with significantly different abundance between severe early preeclampsia and normal pregnancy (q < 0.1) in at least one interval, after adjustment for body mass index, maternal age, parity and smoking status. FC: linear fold change, with negative values denoting lower levels while positive values denote higher levels in cases than in controls.
(XLSX)

S1 File Proteomics data used in the analyses presented in this study. Protein abundance data for each sample (rows) and each of the 1125 proteins is given in this table. Note, unlike for the early preeclampsia group, data for normal pregnancy group is the same as in in [72], and included in this file for convenience. ID: anonymized identifier indicator of the patient, GA: gestational age at sample, GADiagnosis: gestational age at diagnosis for cases; EarlyPE: is 1 for early preeclampsia and 0 for normal pregnancy. EarlyPE_MVM: is 1 for early preeclampsia with maternal vascular malperfusion and 0 for normal pregnancy or early preeclampsia without maternal vascular malperfusion; EarlyPE_Severe: is 1 for severe early preeclampsia cases; Protein symbol and names provided by Somalogic, Inc, are the same as S1 File in [72].
(CSV)

S1 Fig Differential protein abundance analysis by generalized additive mixed models. Longitudinal differences in protein abundance assessed generalized additive mixed models are shown for proteins listed in **Table 2.** For each protein, differences are shown between early preeclampsia (PE) and controls (top left) as well as between mild or severe PE and controls (top right) and between PE with or without maternal vascular malperfusion (MVM) and controls. Thick lines show averages while grey bands give the 95% confidence interval.
(PDF)
