# Supplementary material for: The prediction of early preeclampsia: Results from a longitudinal proteomics study
Source: PLoS One. 2019 Jun 4;14(6):e0217273. doi: 10.1371/journal.pone.0217273 (PMC6548389; doi:10.1371/journal.pone.0217273)
Supplement: S1 Fig — Longitudinal differences in protein abundance assessed generalized additive mixed models are shown for proteins listed in Table 2. For each protein, differences are shown between early preeclampsia (PE) and controls (top left) as well as between mild or severe PE and controls (top right) and between PE with or without maternal vascular malperfusion (MVM) and controls. Thick lines show averages while grey bands give the 95% confidence interval. (PDF) [file pone.0217273.s006.pdf]

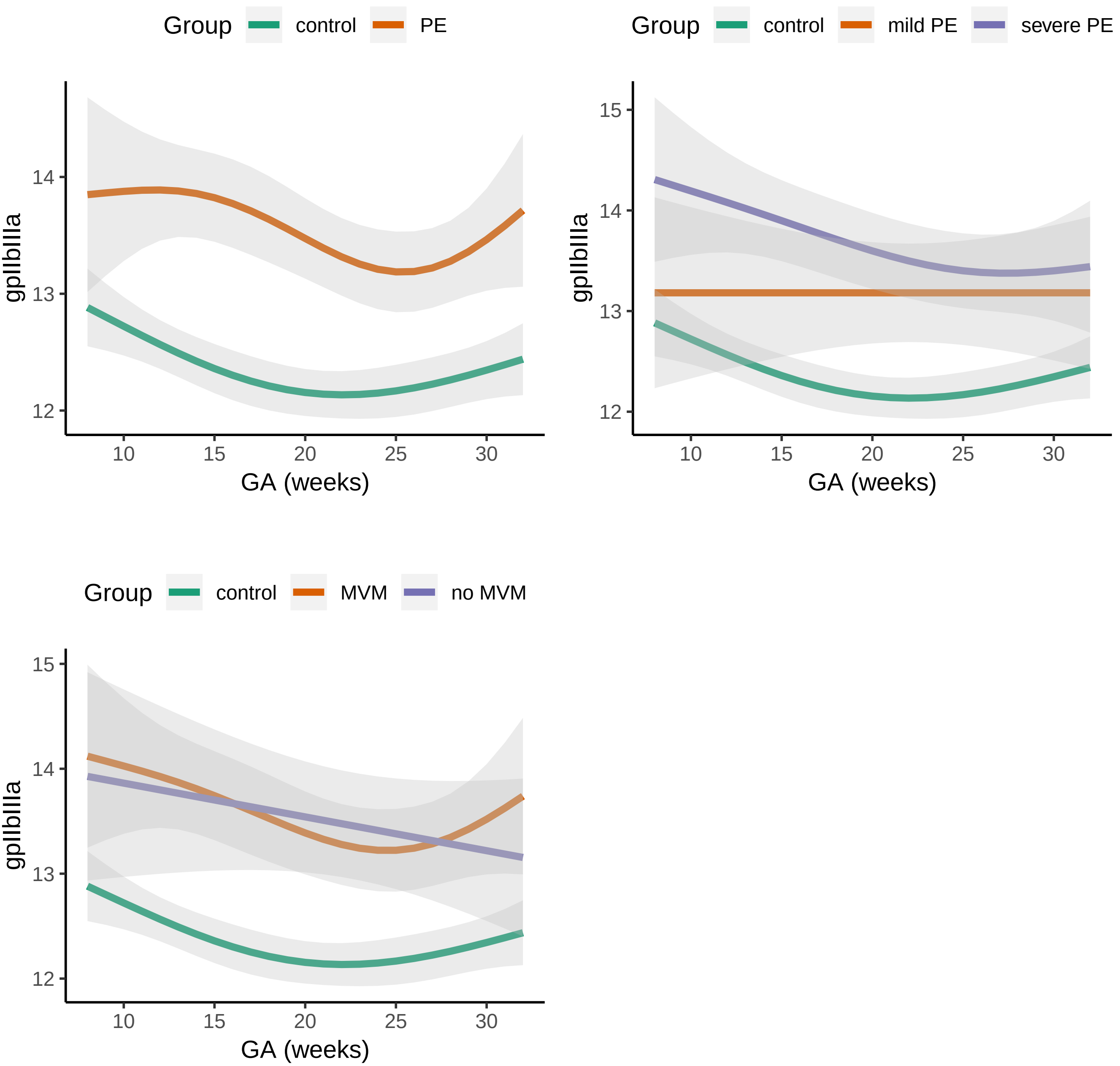

Group
control
PE
gpIIbIIIa
GA (weeks)
Group
control
mild PE
severe PE
gpIIbIIIa
GA (weeks)
Group
control
MVM
no MVM
gpIIbIIIa
GA (weeks)

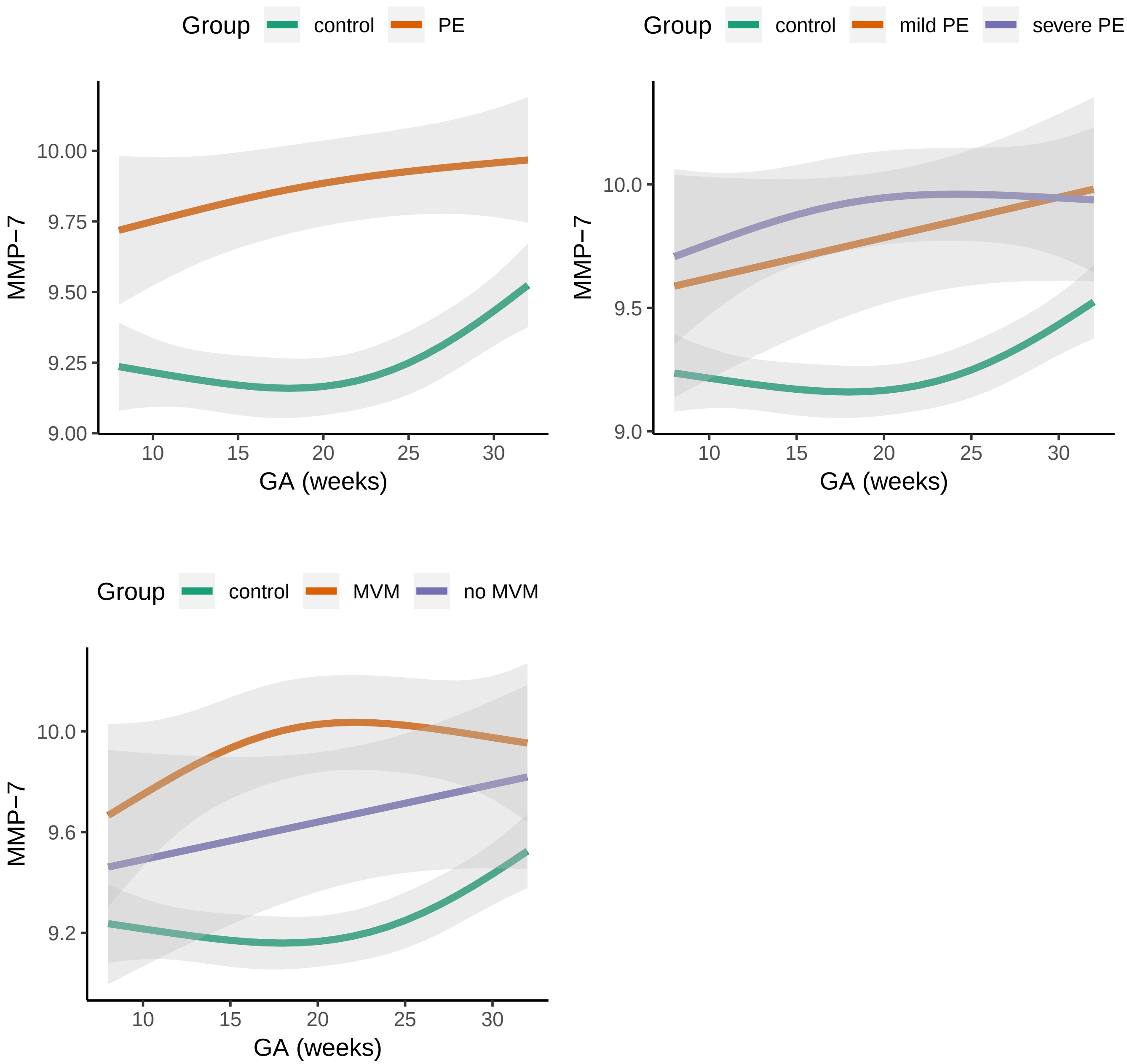

Group
control
PE
MMP−7
10.00
9.75
9.50
9.25
9.00
10
15
20
25
30
GA (weeks)
Group
control
mild PE
severe PE
MMP−7
10.0
9.5
9.0
10
15
20
25
30
GA (weeks)
Group
control
MVM
no MVM
MMP−7
10.0
9.6
9.2
10
15
20
25
30
GA (weeks)

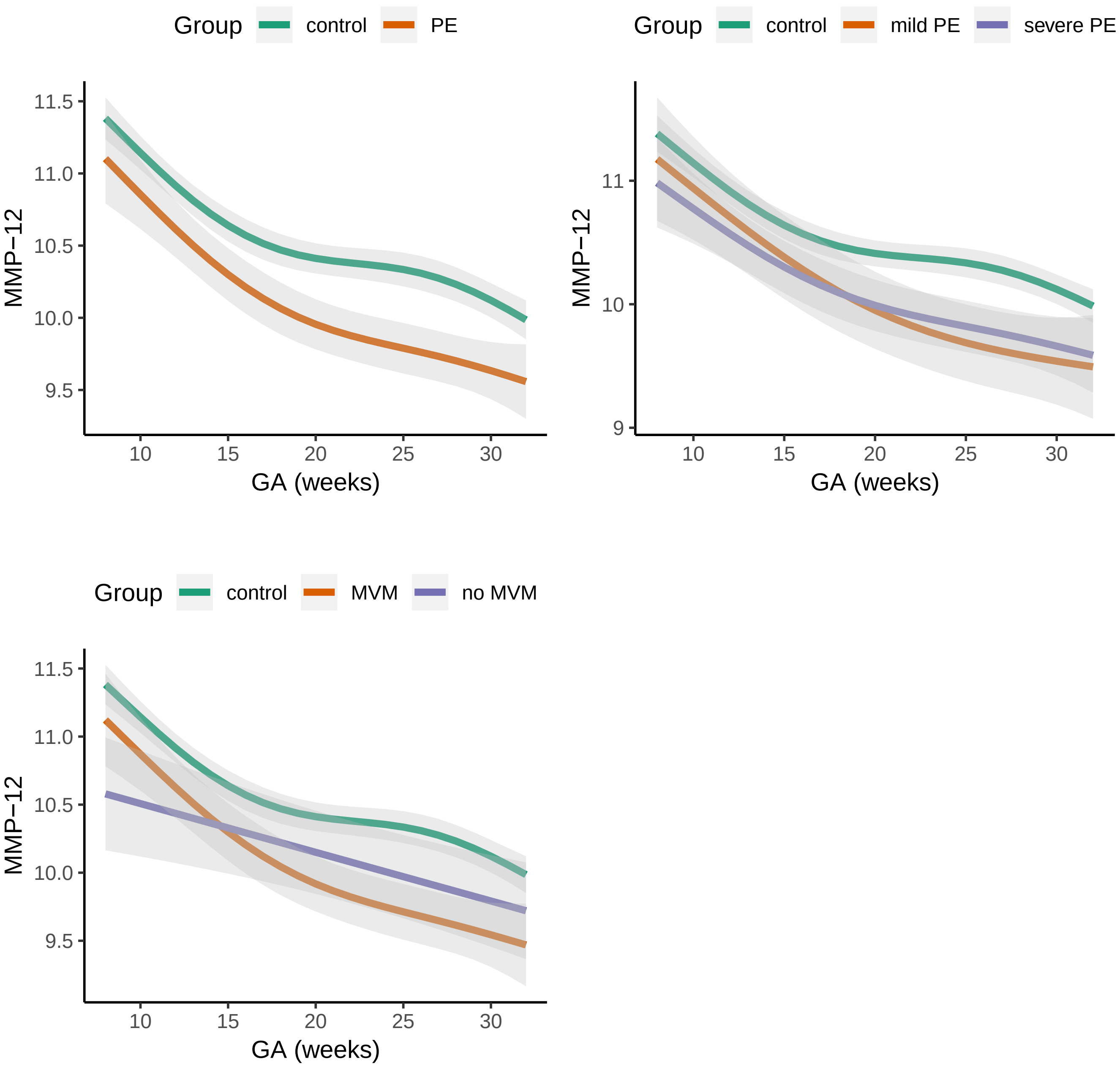

Group
control
PE
MMP−12
11.5
11.0
10.5
10.0
9.5
10
15
20
25
30
GA (weeks)
Group
control
mild PE
severe PE
MMP−12
11
10
9
10
15
20
25
30
GA (weeks)
Group
control
MVM
no MVM
MMP−12
11.5
11.0
10.5
10.0
9.5
10
15
20
25
30
GA (weeks)

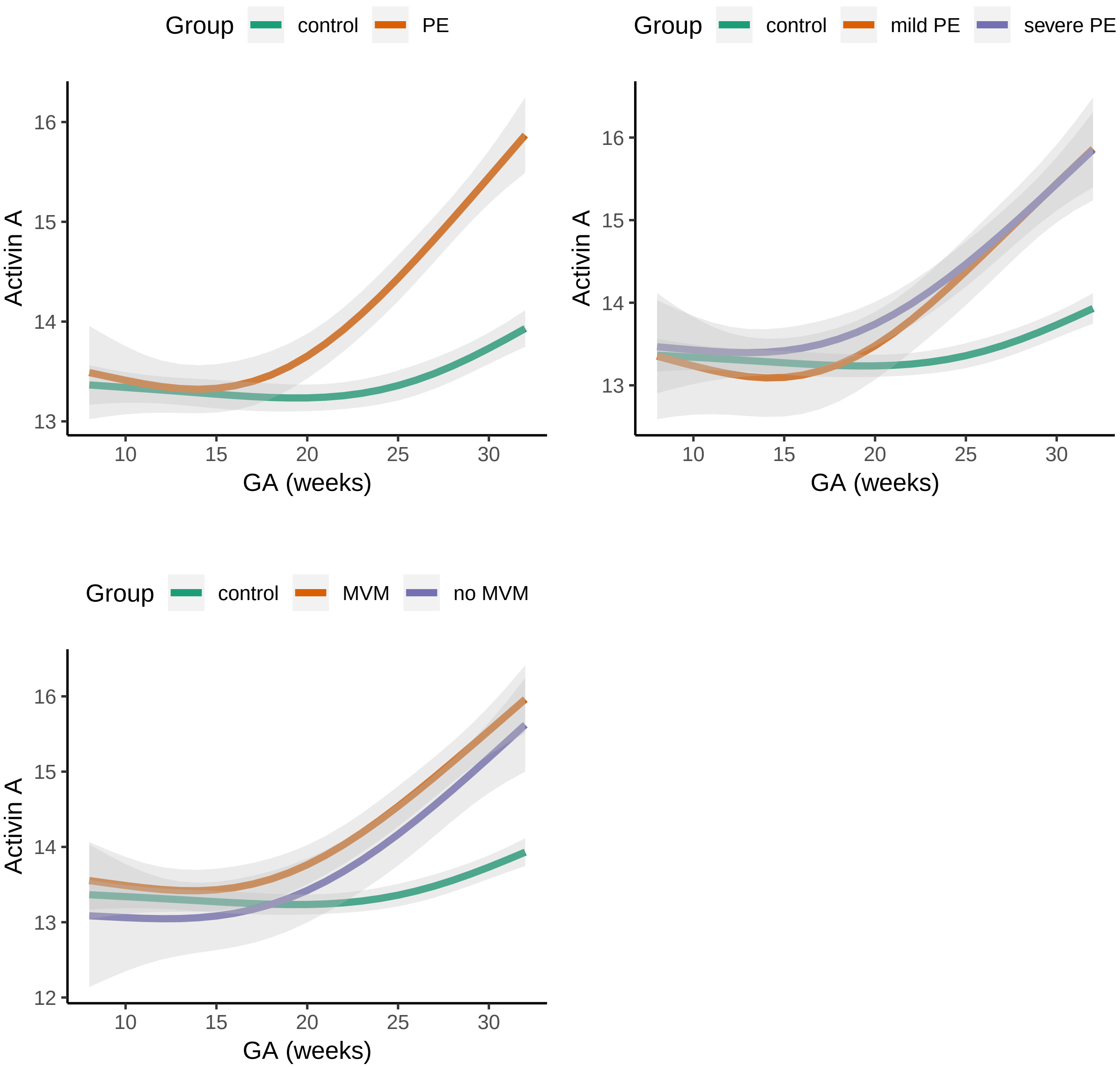

Group
control
PE
Activin A
GA (weeks)
Group
control
mild PE
severe PE
Activin A
GA (weeks)
Group
control
MVM
no MVM
Activin A
GA (weeks)

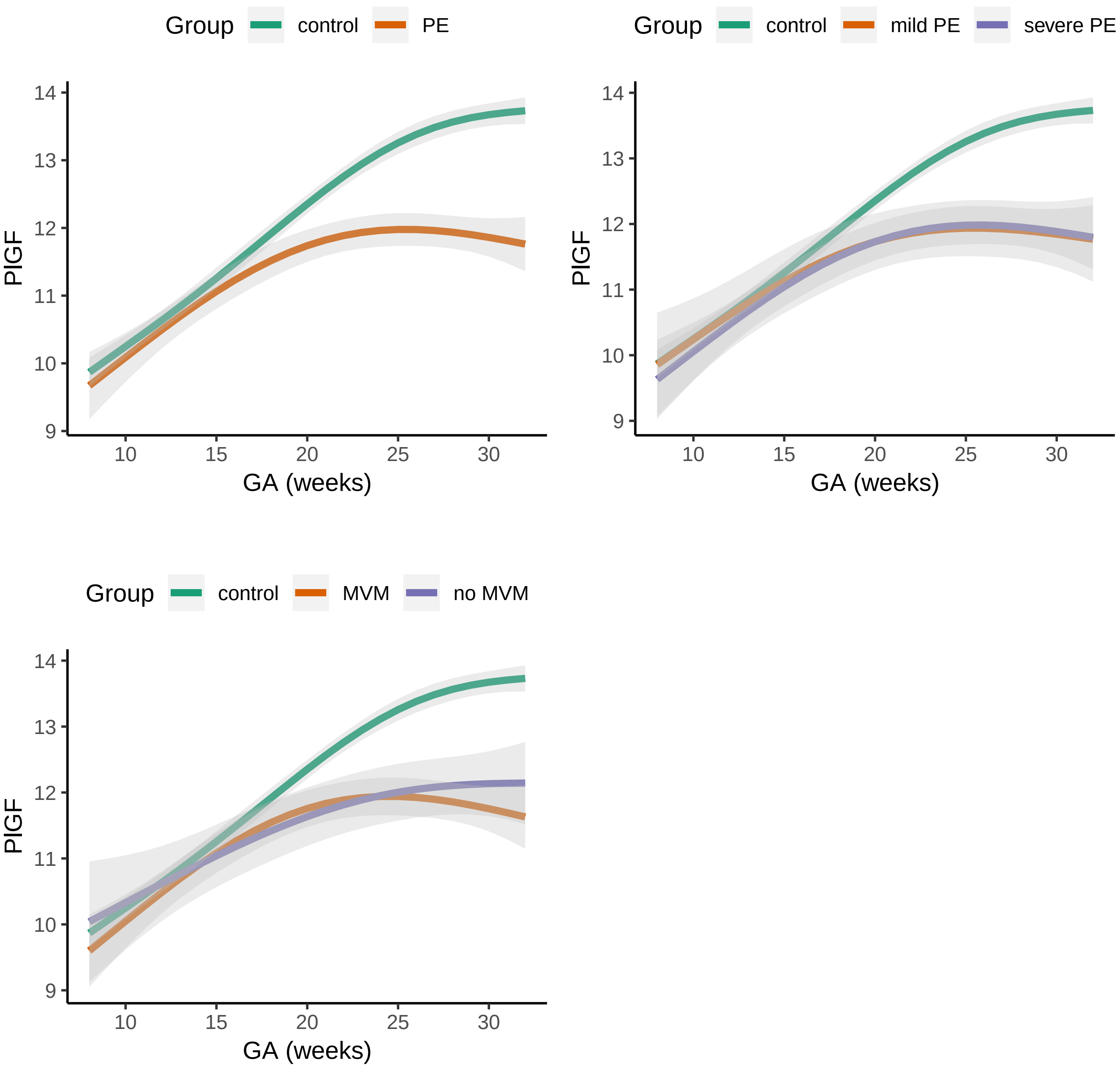
Group
control
PE
PlGF
GA (weeks)
Group
control
mild PE
severe PE
PlGF
GA (weeks)
Group
control
MVM
no MVM
PlGF
GA (weeks)
9
10
11
12
13
14
10
15
20
25
30

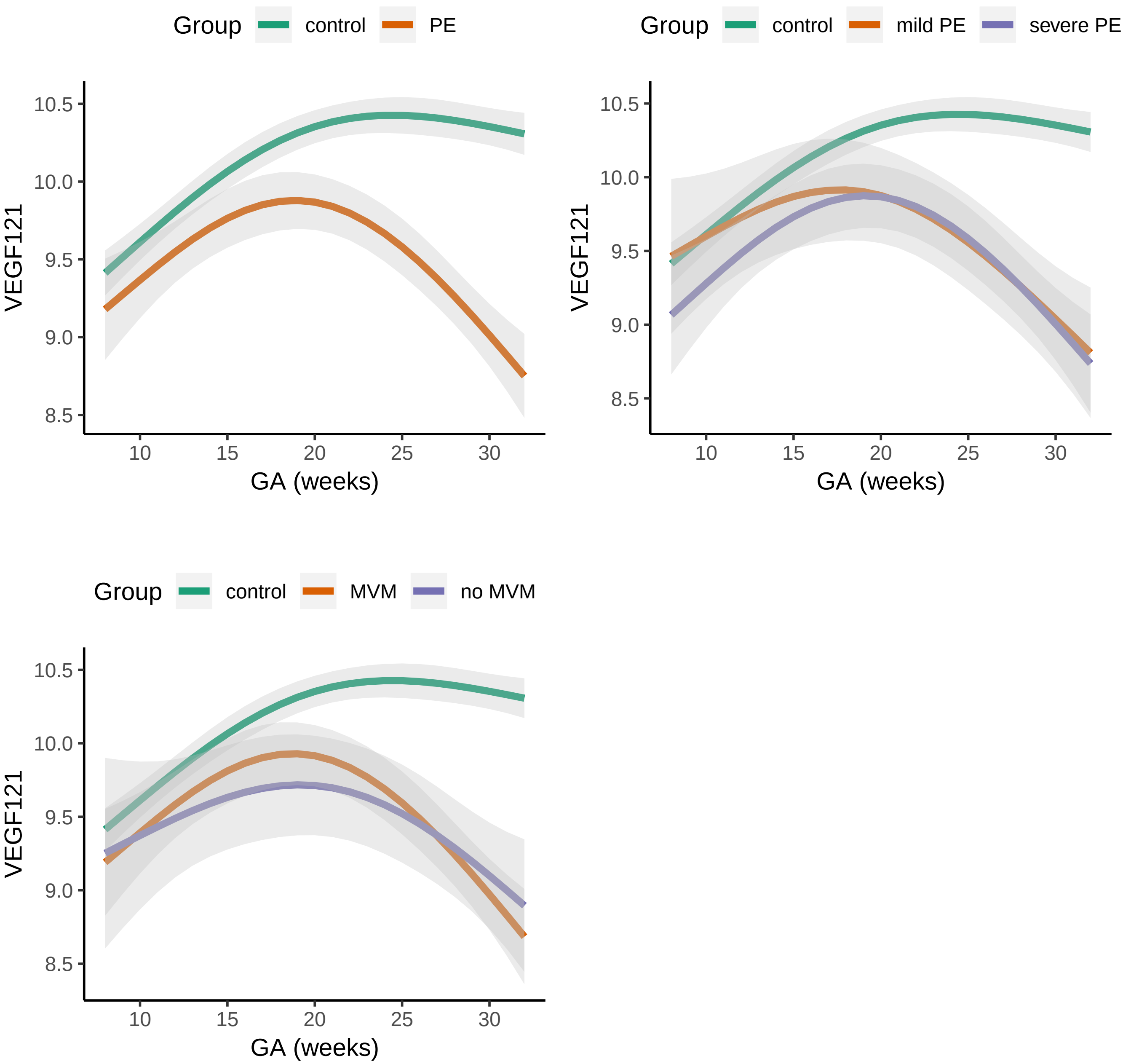

Group
control
PE
10.5
10.0
9.5
9.0
8.5
VEGF121
10
15
20
25
30
GA (weeks)
Group
control
mild PE
severe PE
10.5
10.0
9.5
9.0
8.5
VEGF121
10
15
20
25
30
GA (weeks)
Group
control
MVM
no MVM
10.5
10.0
9.5
9.0
8.5
VEGF121
10
15
20
25
30
GA (weeks)

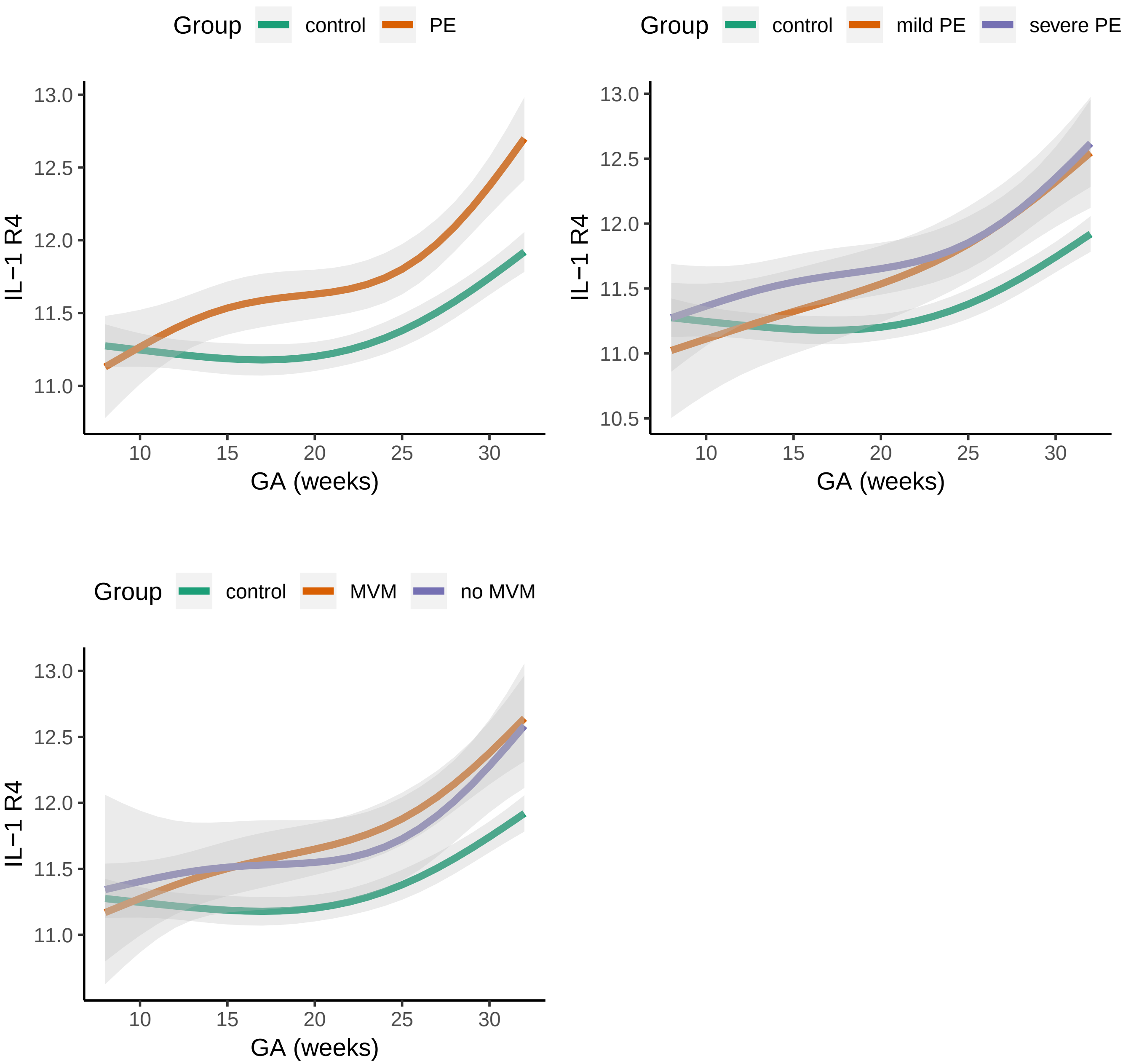
Group
control
PE
IL−1 R4
13.0
12.5
12.0
11.5
11.0
10
15
20
25
30
GA (weeks)
Group
control
mild PE
severe PE
IL−1 R4
13.0
12.5
12.0
11.5
11.0
10.5
10
15
20
25
30
GA (weeks)
Group
control
MVM
no MVM
IL−1 R4
13.0
12.5
12.0
11.5
11.0
10
15
20
25
30
GA (weeks)

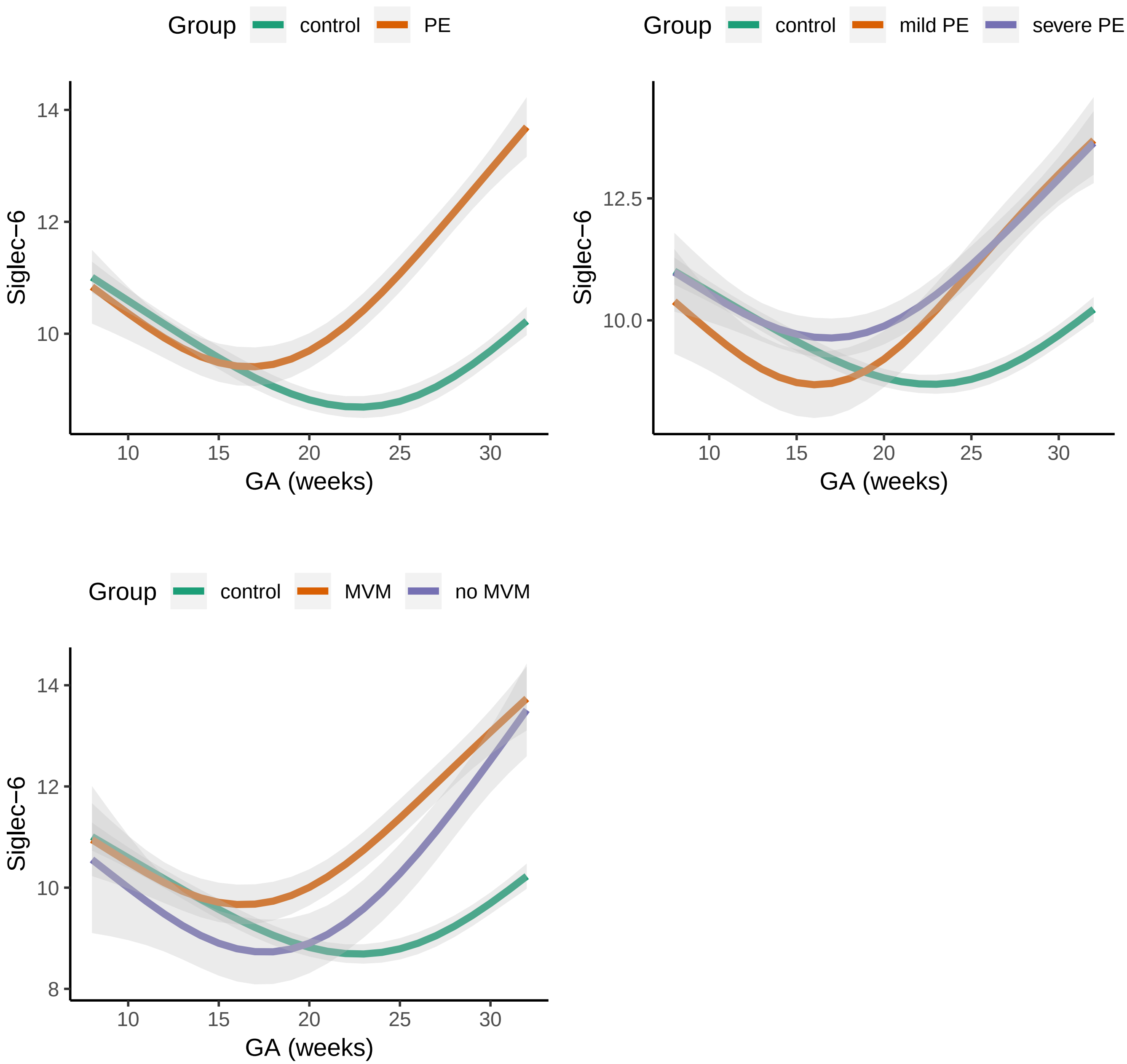

Group
control
PE
Siglec−6
14
12
10
10
15
20
25
30
GA (weeks)
Group
control
mild PE
severe PE
Siglec−6
12.5
10.0
10
15
20
25
30
GA (weeks)
Group
control
MVM
no MVM
Siglec−6
14
12
10
8
10
15
20
25
30
GA (weeks)

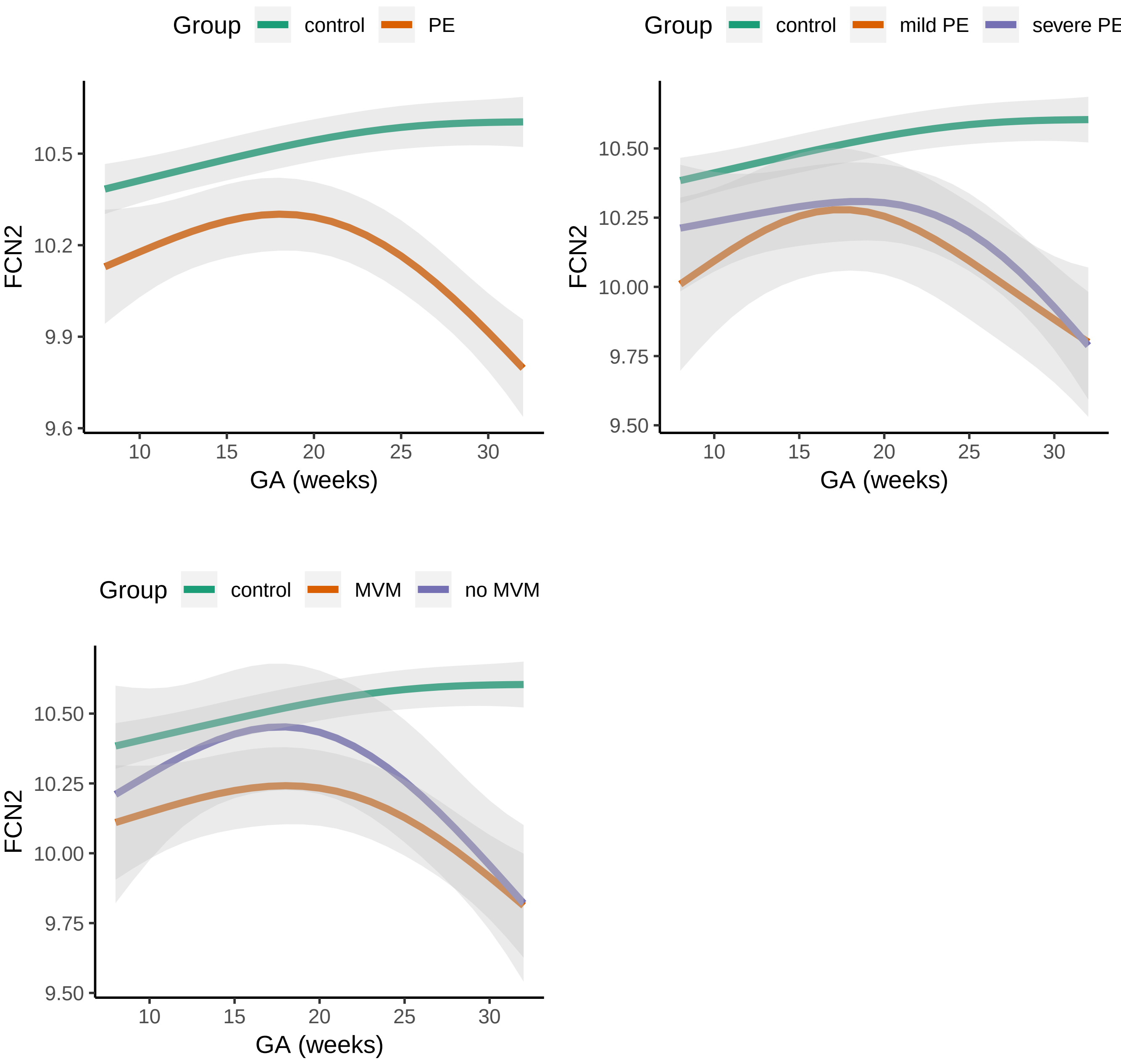

Group
control
PE
10.5
10.2
9.9
9.6
FCN2
10
15
20
25
30
GA (weeks)
Group
control
mild PE
severe PE
10.50
10.25
10.00
9.75
9.50
FCN2
10
15
20
25
30
GA (weeks)
Group
control
MVM
no MVM
10.50
10.25
10.00
9.75
9.50
FCN2
10
15
20
25
30
GA (weeks)

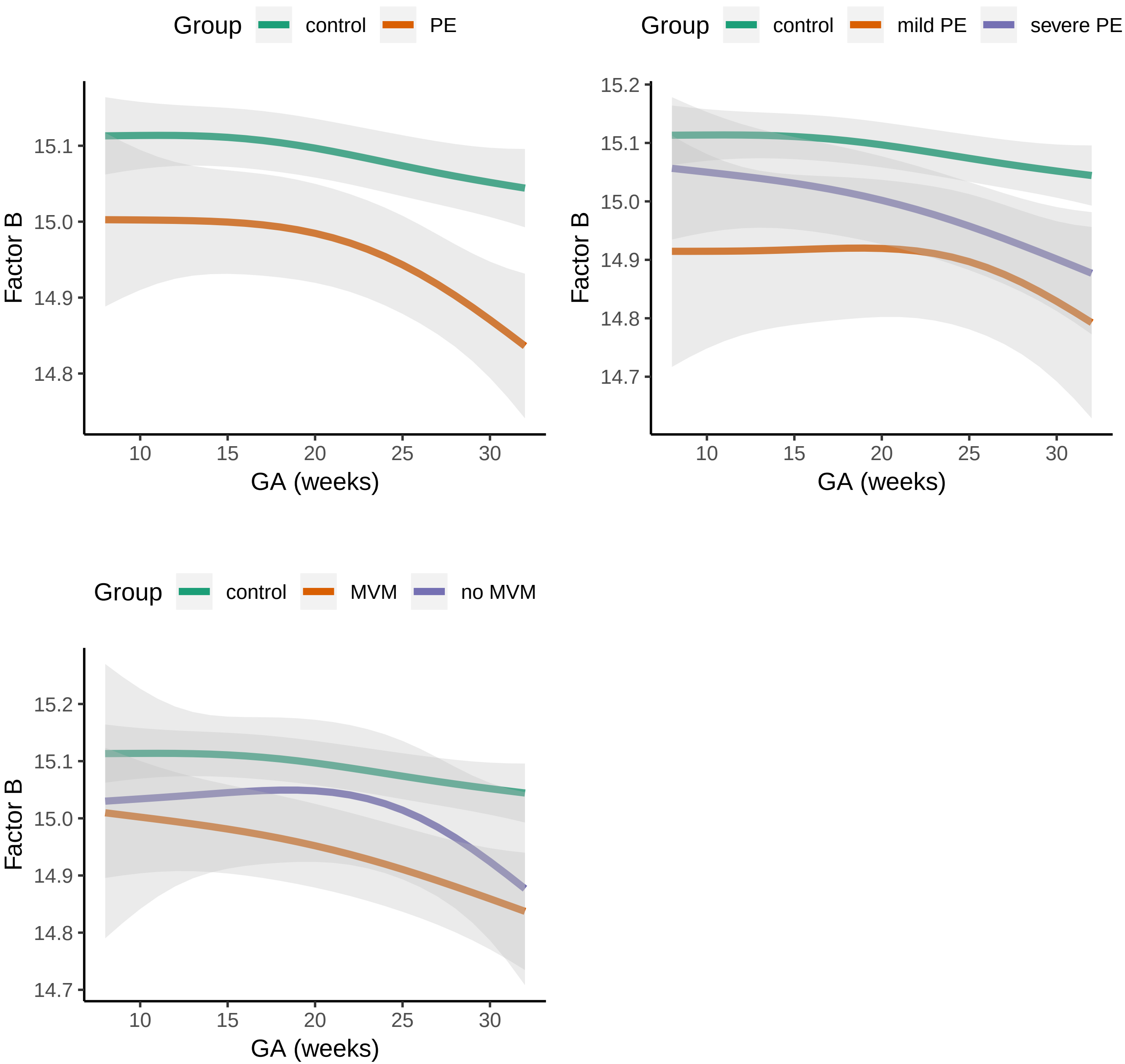

Group
control
PE
Group
control
mild PE
severe PE
Group
control
MVM
no MVM
Factor B
GA (weeks)
15.2
15.1
15.0
14.9
14.8
14.7
10
15
20
25
30

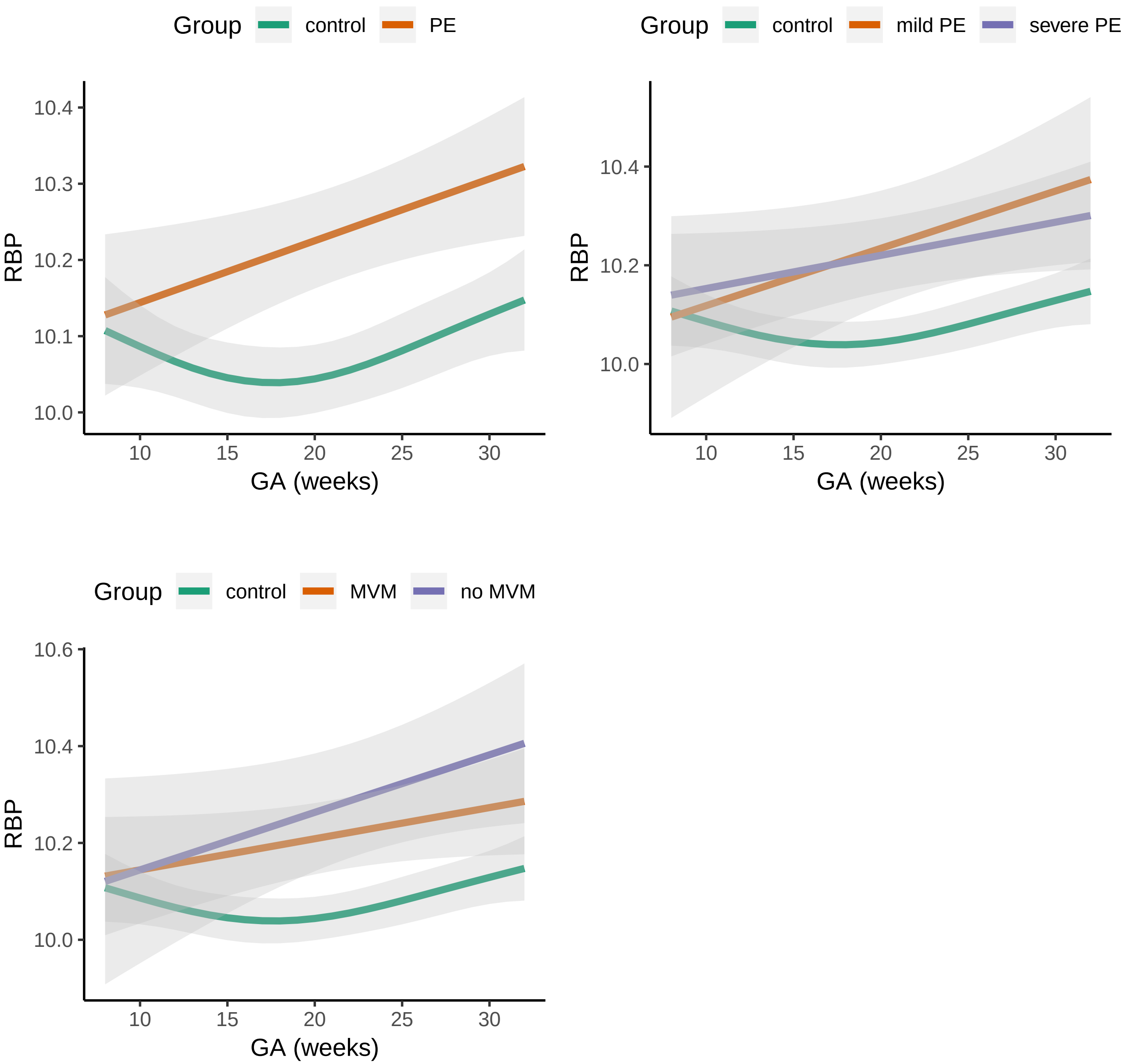

Group
control
PE
10.4
10.3
10.2
10.1
10.0
RBP
10
15
20
25
30
GA (weeks)
Group
control
mild PE
severe PE
10.4
10.2
10.0
RBP
10
15
20
25
30
GA (weeks)
Group
control
MVM
no MVM
10.6
10.4
10.2
10.0
RBP
10
15
20
25
30
GA (weeks)

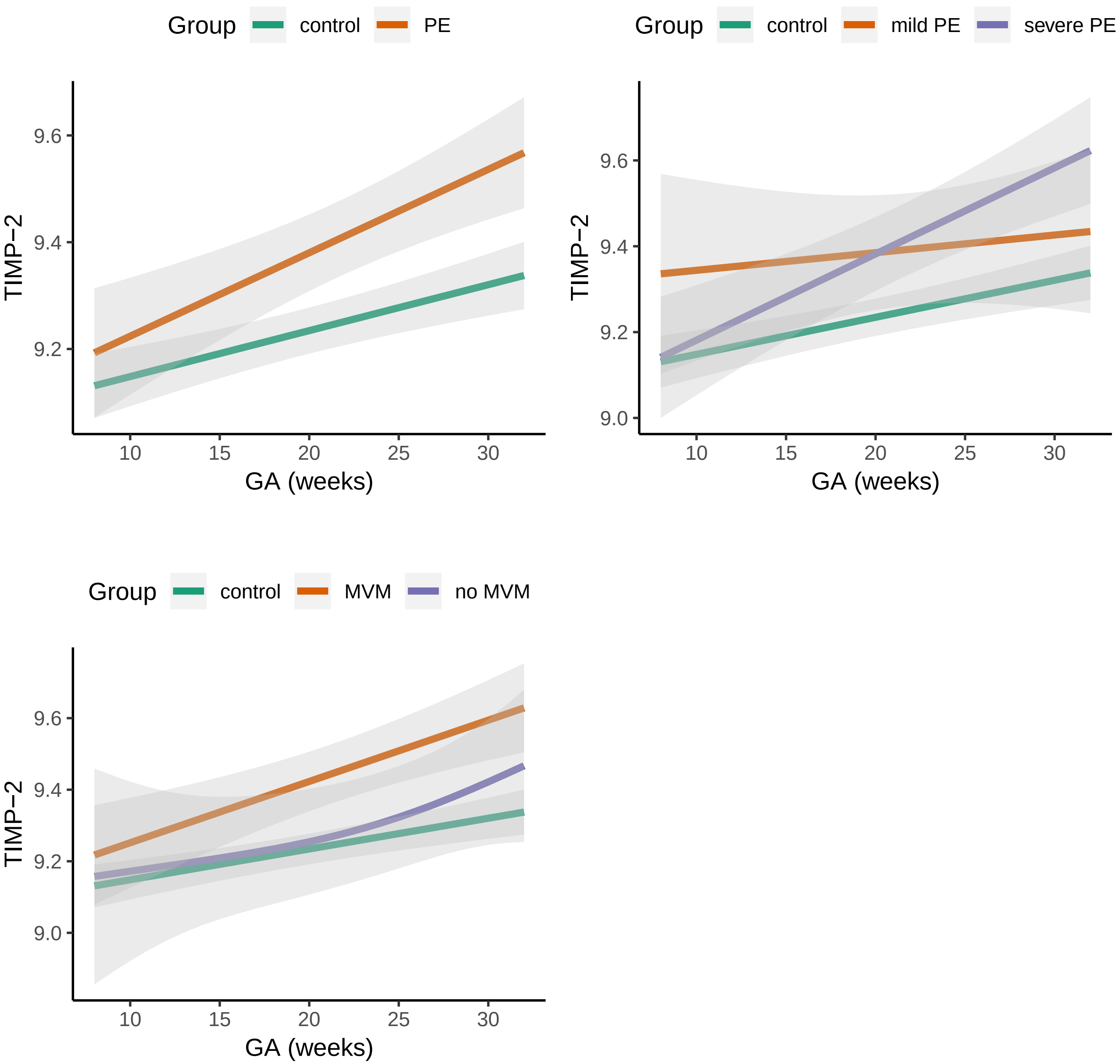
Group
control
PE
TIMP−2
9.2
9.4
9.6
10
15
20
25
30
GA (weeks)
Group
control
mild PE
severe PE
TIMP−2
9.0
9.2
9.4
9.6
10
15
20
25
30
GA (weeks)
Group
control
MVM
no MVM
TIMP−2
9.0
9.2
9.4
9.6
10
15
20
25
30
GA (weeks)

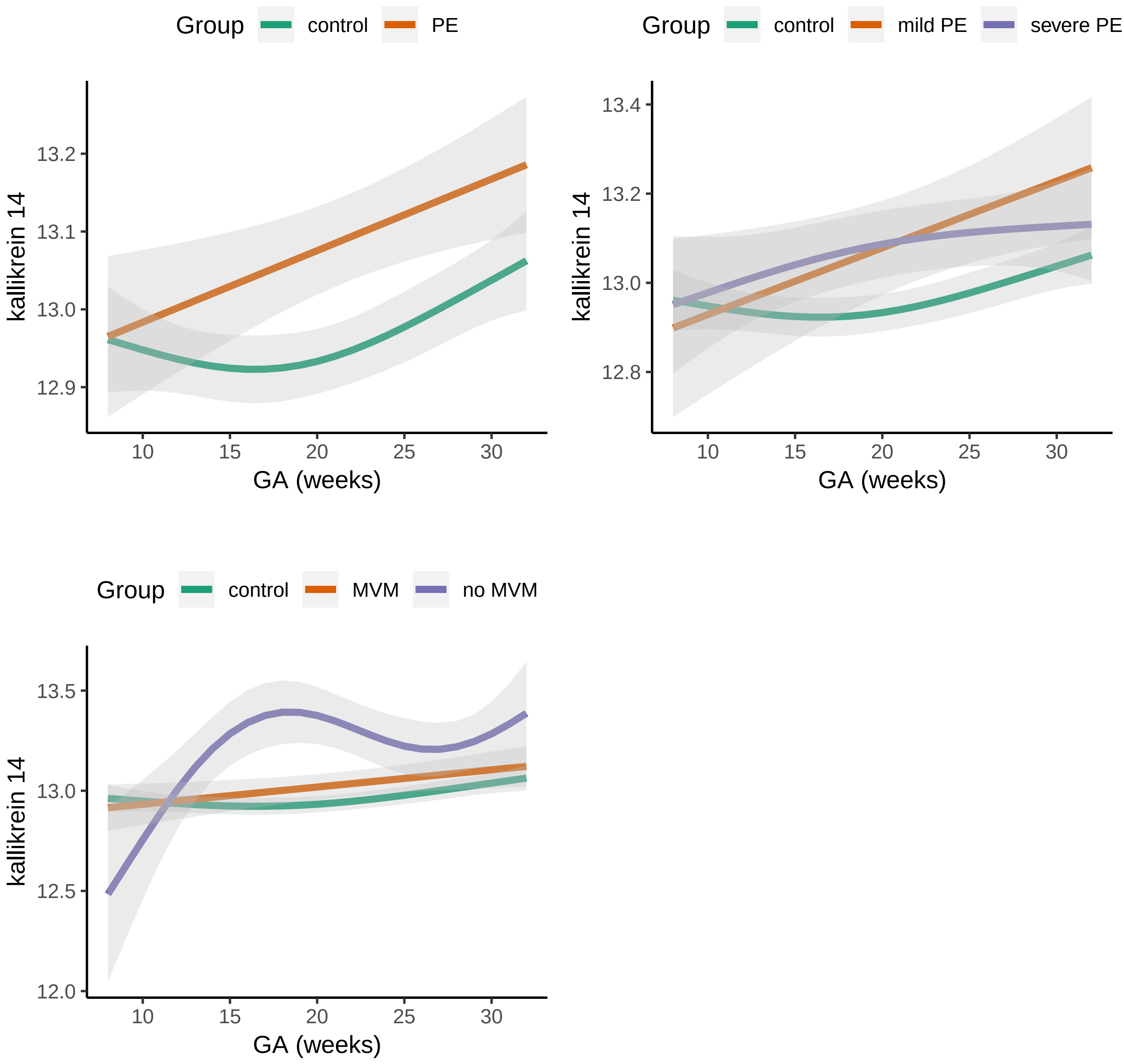
Group
control
PE
kallikrein 14
13.2
13.1
13.0
12.9
10
15
20
25
30
GA (weeks)
Group
control
mild PE
severe PE
kallikrein 14
13.4
13.2
13.0
12.8
10
15
20
25
30
GA (weeks)
Group
control
MVM
no MVM
kallikrein 14
13.5
13.0
12.5
12.0
10
15
20
25
30
GA (weeks)

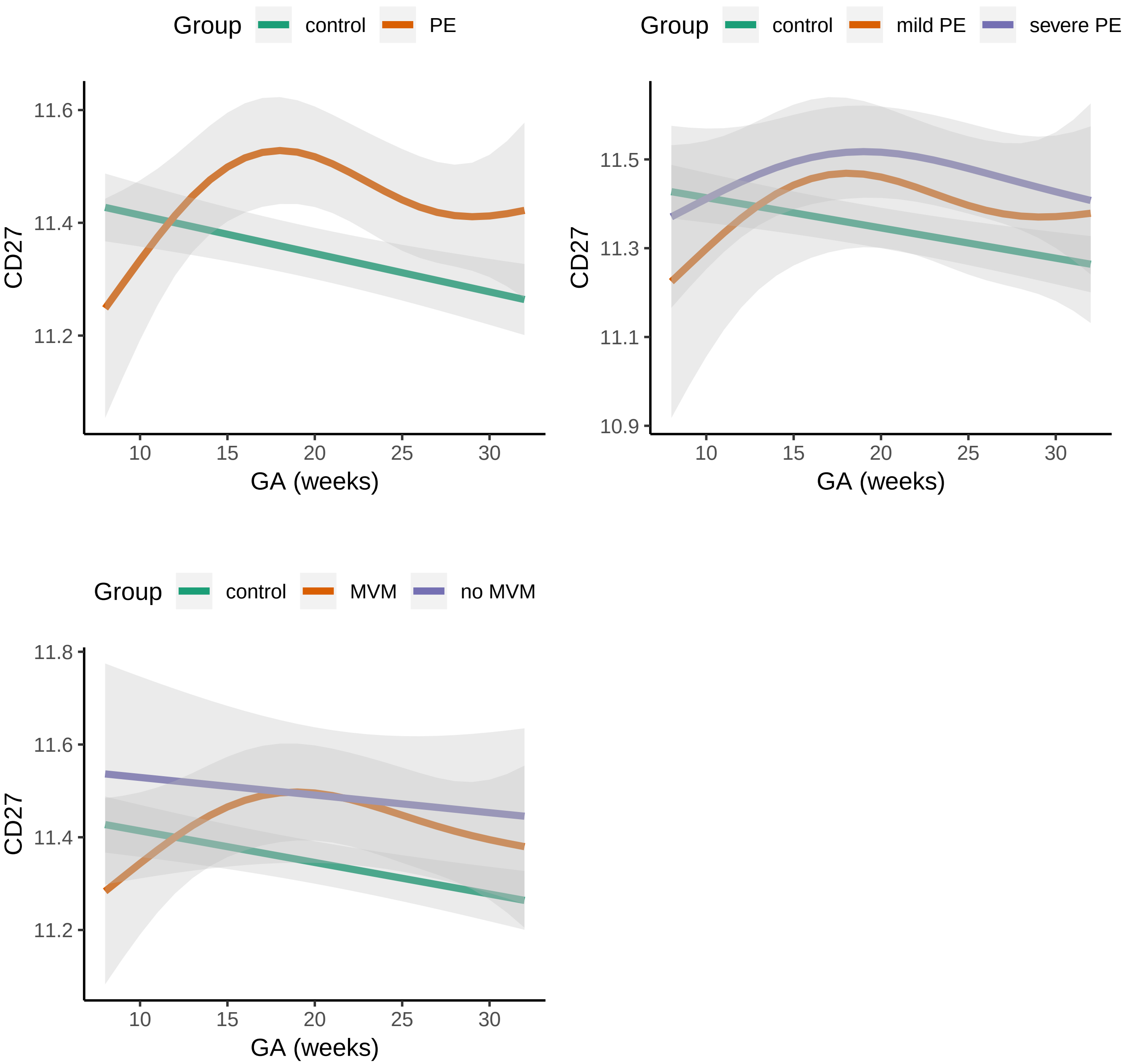

Group
control
PE
11.6
11.4
11.2
CD27
10
15
20
25
30
GA (weeks)
Group
control
mild PE
severe PE
11.5
11.3
11.1
10.9
CD27
10
15
20
25
30
GA (weeks)
Group
control
MVM
no MVM
11.8
11.6
11.4
11.2
CD27
10
15
20
25
30
GA (weeks)

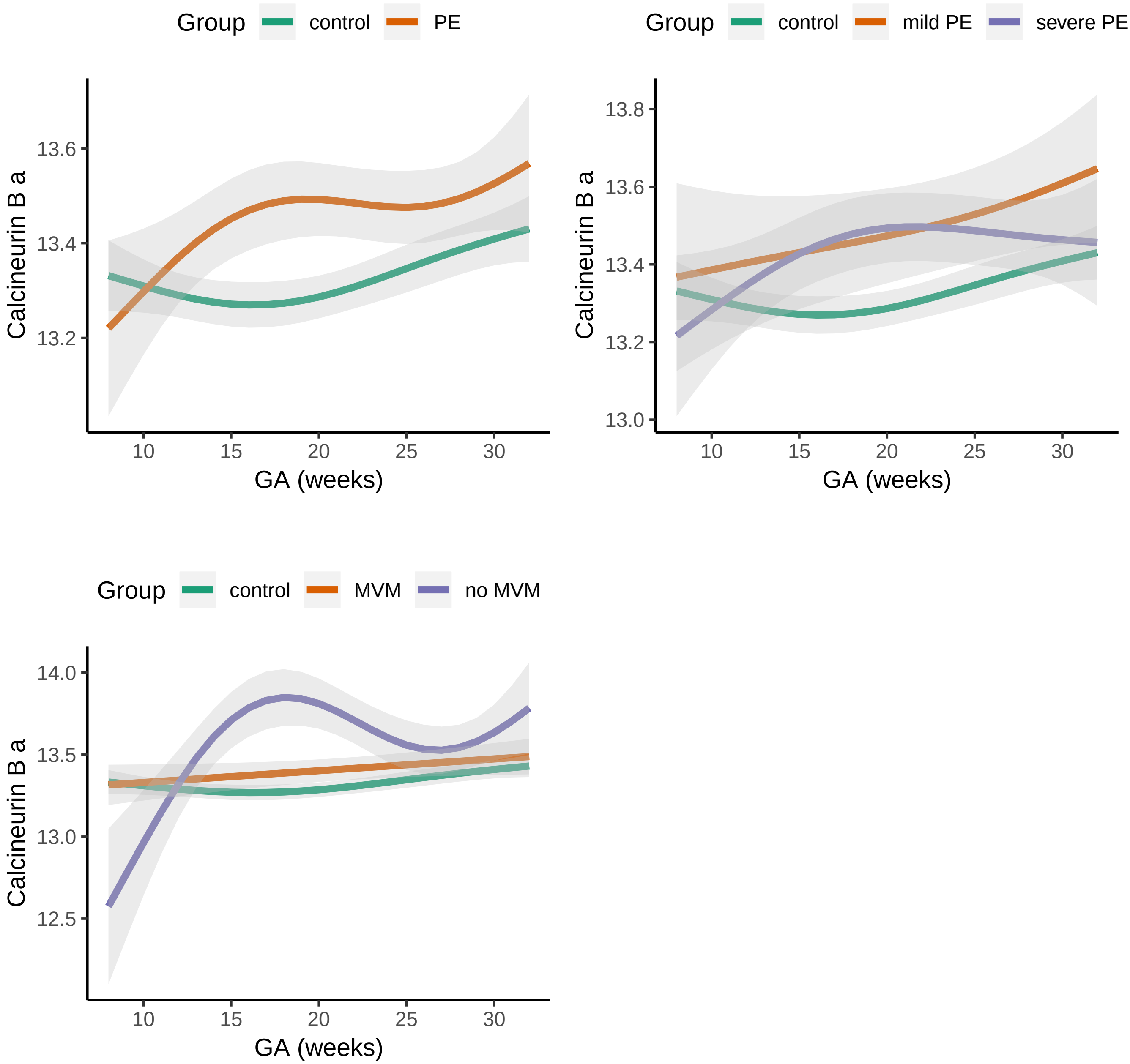

Group
control
PE
Calcineurin B a
13.2
13.4
13.6
10
15
20
25
30
GA (weeks)
Group
control
mild PE
severe PE
Calcineurin B a
13.0
13.2
13.4
13.6
13.8
10
15
20
25
30
GA (weeks)
Group
control
MVM
no MVM
Calcineurin B a
12.5
13.0
13.5
14.0
10
15
20
25
30
GA (weeks)

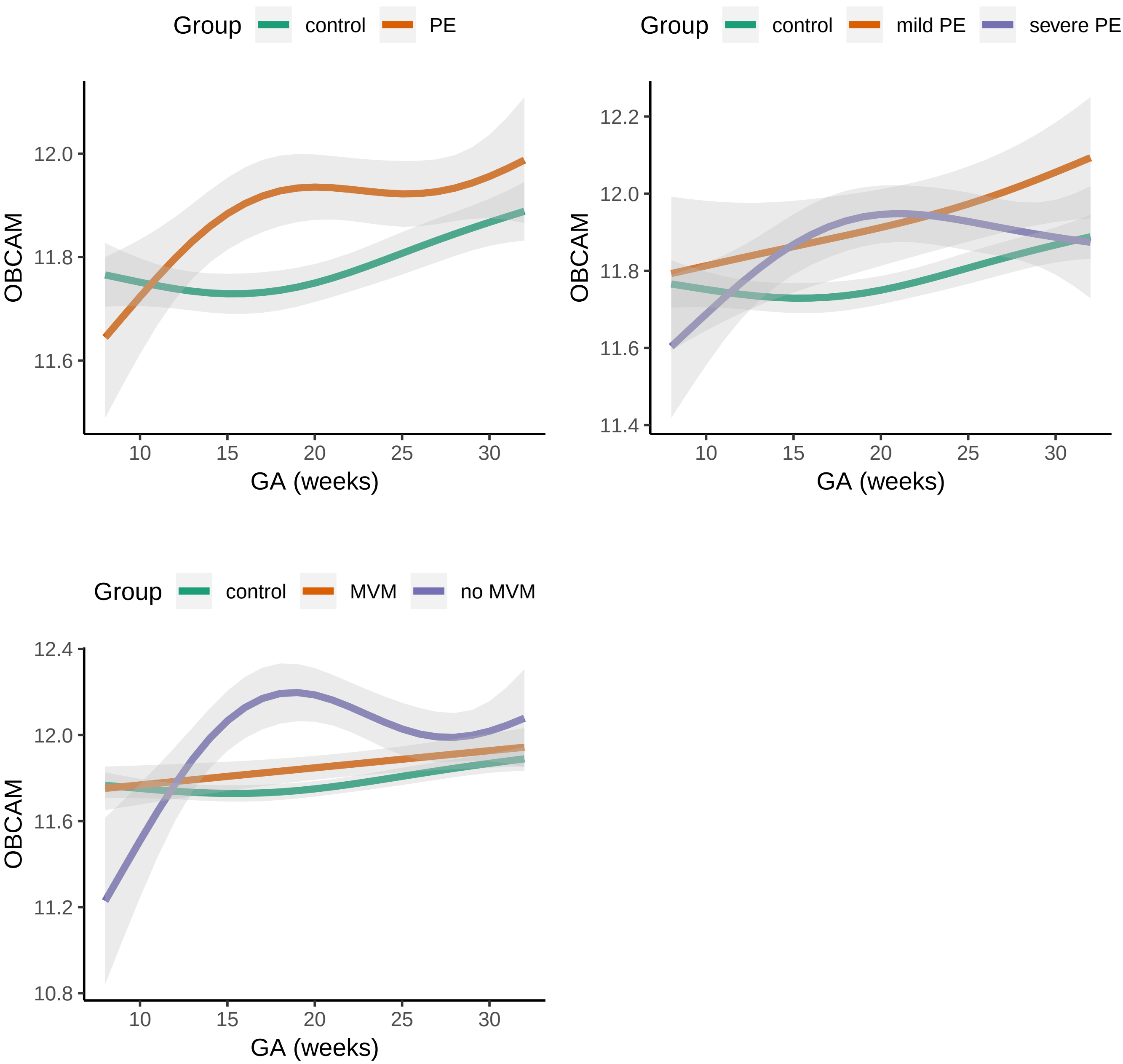

Group
control
PE
OBCAM
12.0
11.8
11.6
10
15
20
25
30
GA (weeks)
Group
control
mild PE
severe PE
OBCAM
12.2
12.0
11.8
11.6
11.4
10
15
20
25
30
GA (weeks)
Group
control
MVM
no MVM
OBCAM
12.4
12.0
11.6
11.2
10.8
10
15
20
25
30
GA (weeks)

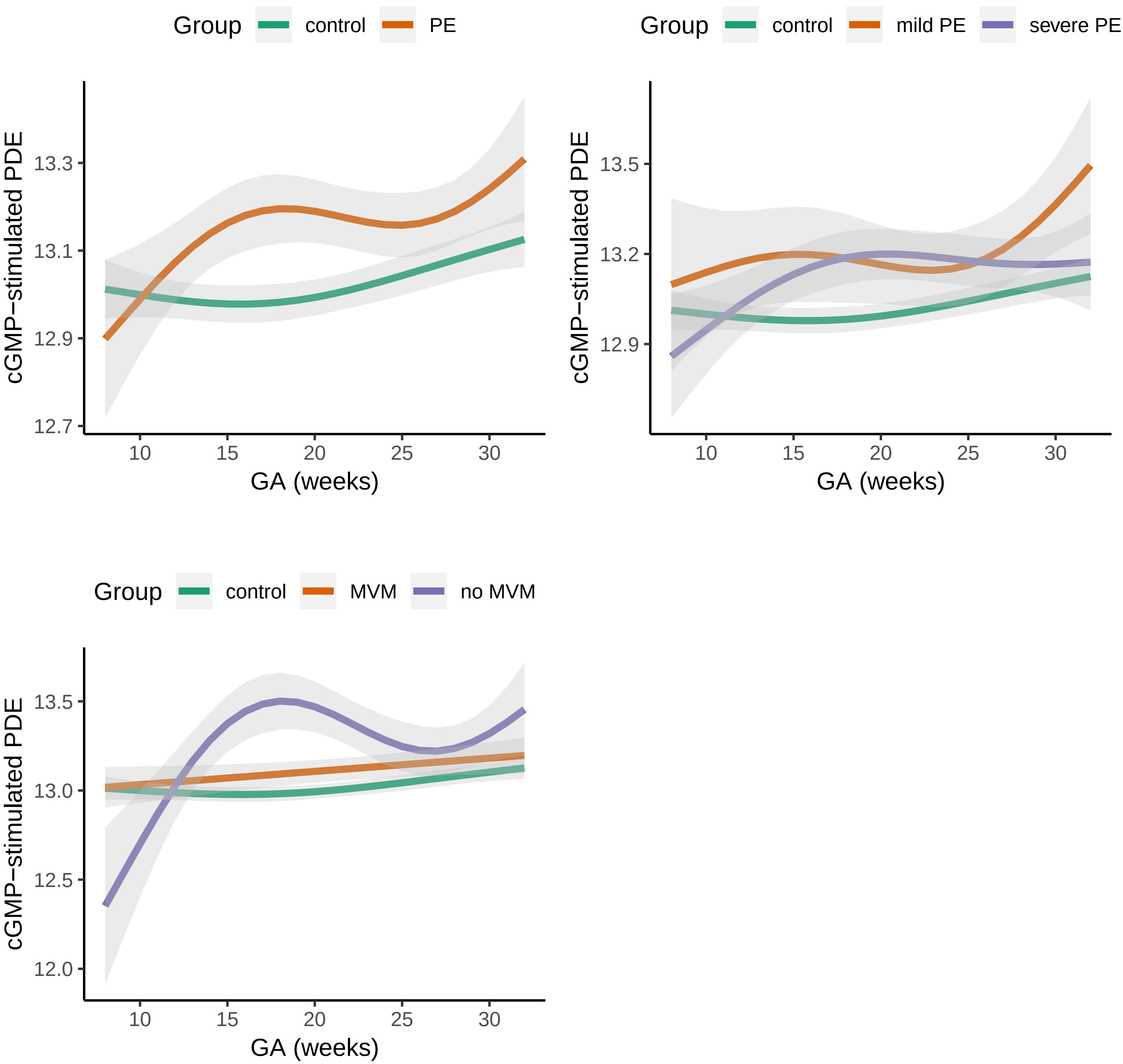
Group
control
PE
cGMP−stimulated PDE
13.3
13.1
12.9
12.7
10
15
20
25
30
GA (weeks)
Group
control
mild PE
severe PE
cGMP−stimulated PDE
13.5
13.2
12.9
10
15
20
25
30
GA (weeks)
Group
control
MVM
no MVM
cGMP−stimulated PDE
13.5
13.0
12.5
12.0
10
15
20
25
30
GA (weeks)

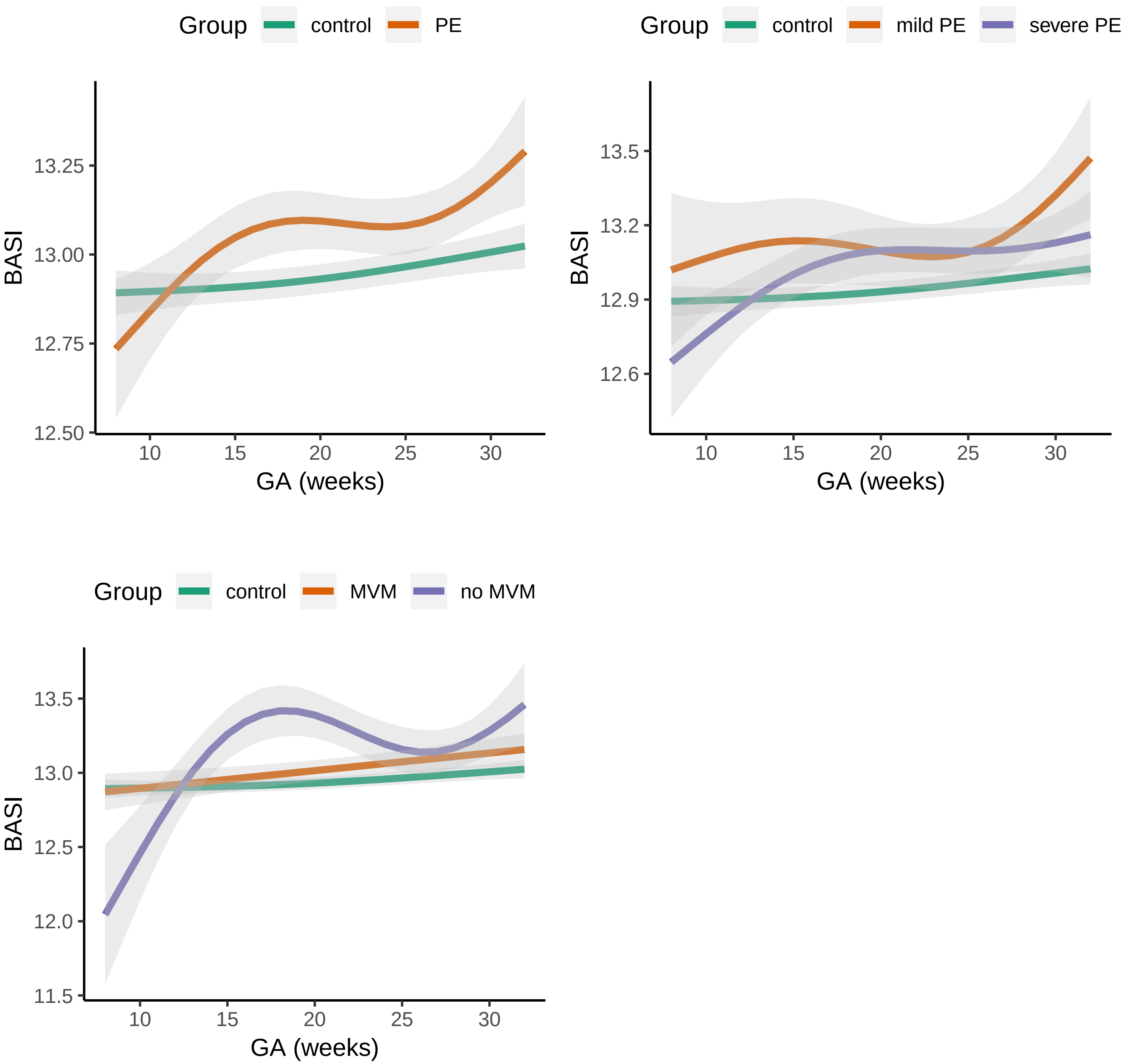

Group
control
PE
BASI
13.25
13.00
12.75
12.50
10
15
20
25
30
GA (weeks)
Group
control
mild PE
severe PE
BASI
13.5
13.2
12.9
12.6
10
15
20
25
30
GA (weeks)
Group
control
MVM
no MVM
BASI
13.5
13.0
12.5
12.0
11.5
10
15
20
25
30
GA (weeks)

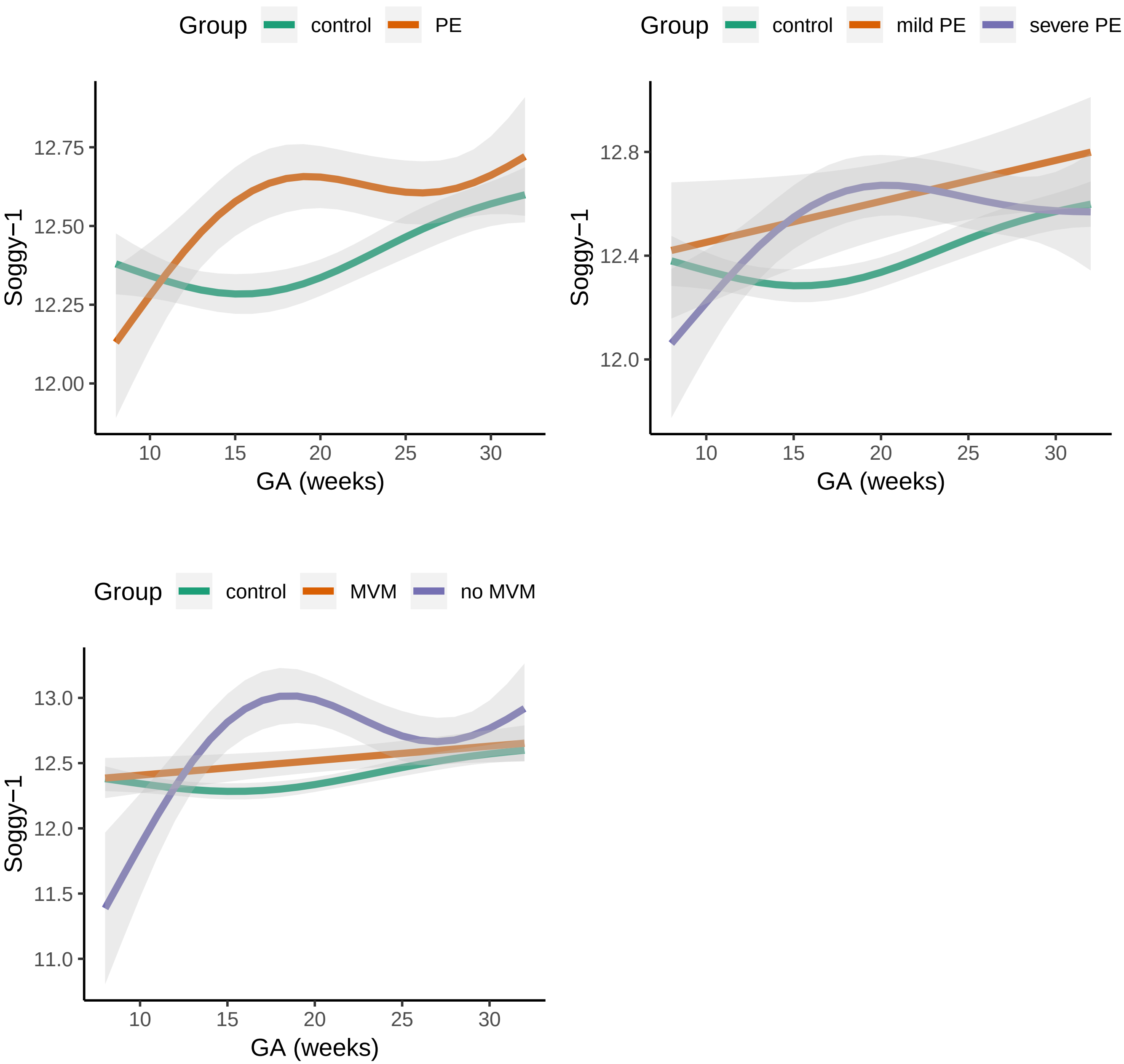

Group
control
PE
Soggy−1
12.75
12.50
12.25
12.00
10
15
20
25
30
GA (weeks)
Group
control
mild PE
severe PE
Soggy−1
12.8
12.4
12.0
10
15
20
25
30
GA (weeks)
Group
control
MVM
no MVM
Soggy−1
13.0
12.5
12.0
11.5
11.0
10
15
20
25
30
GA (weeks)

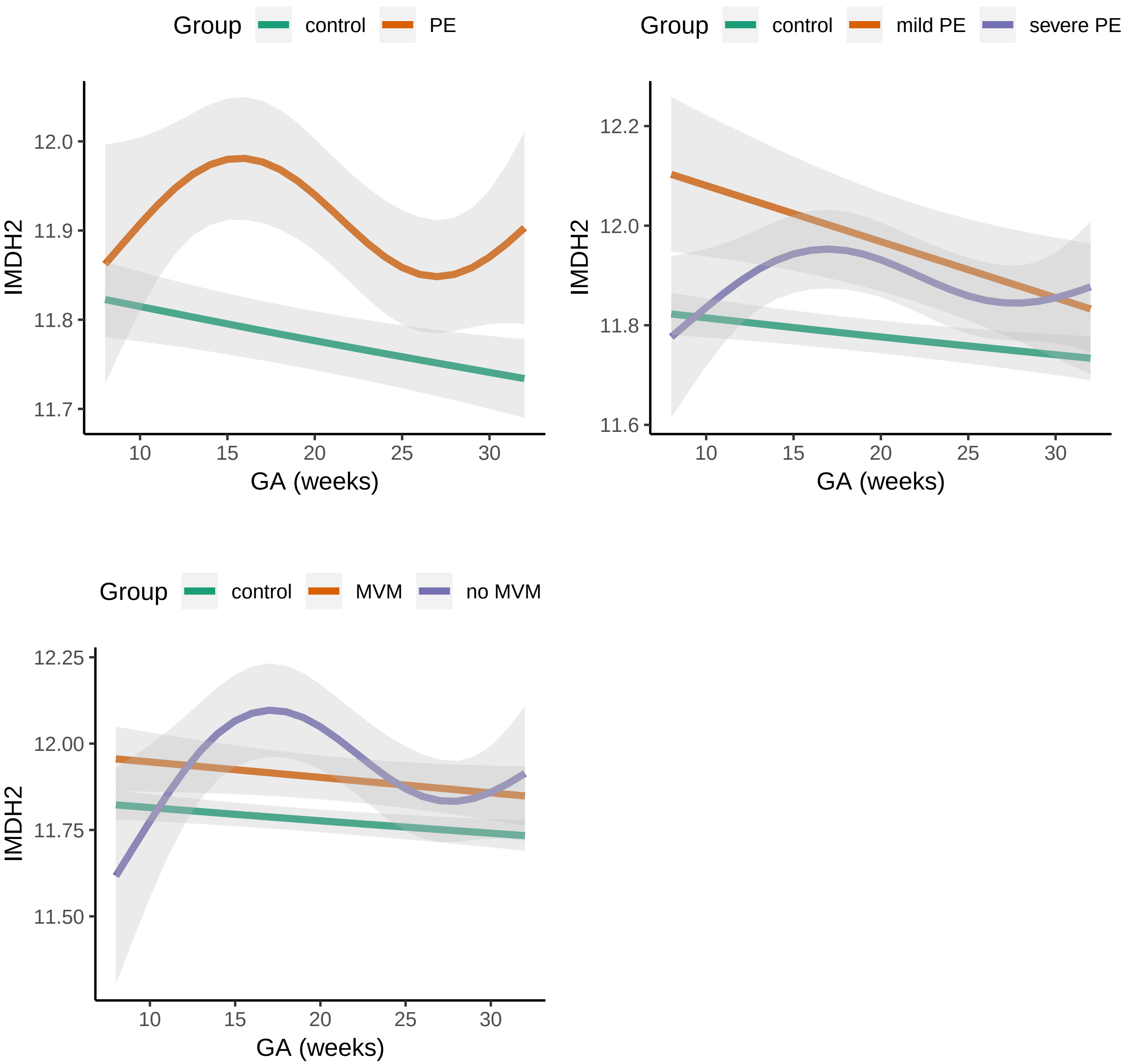

Group
control
PE
12.0
11.9
11.8
11.7
IMDH2
10
15
20
25
30
GA (weeks)
Group
control
mild PE
severe PE
12.2
12.0
11.8
11.6
IMDH2
10
15
20
25
30
GA (weeks)
Group
control
MVM
no MVM
12.25
12.00
11.75
11.50
IMDH2
10
15
20
25
30
GA (weeks)

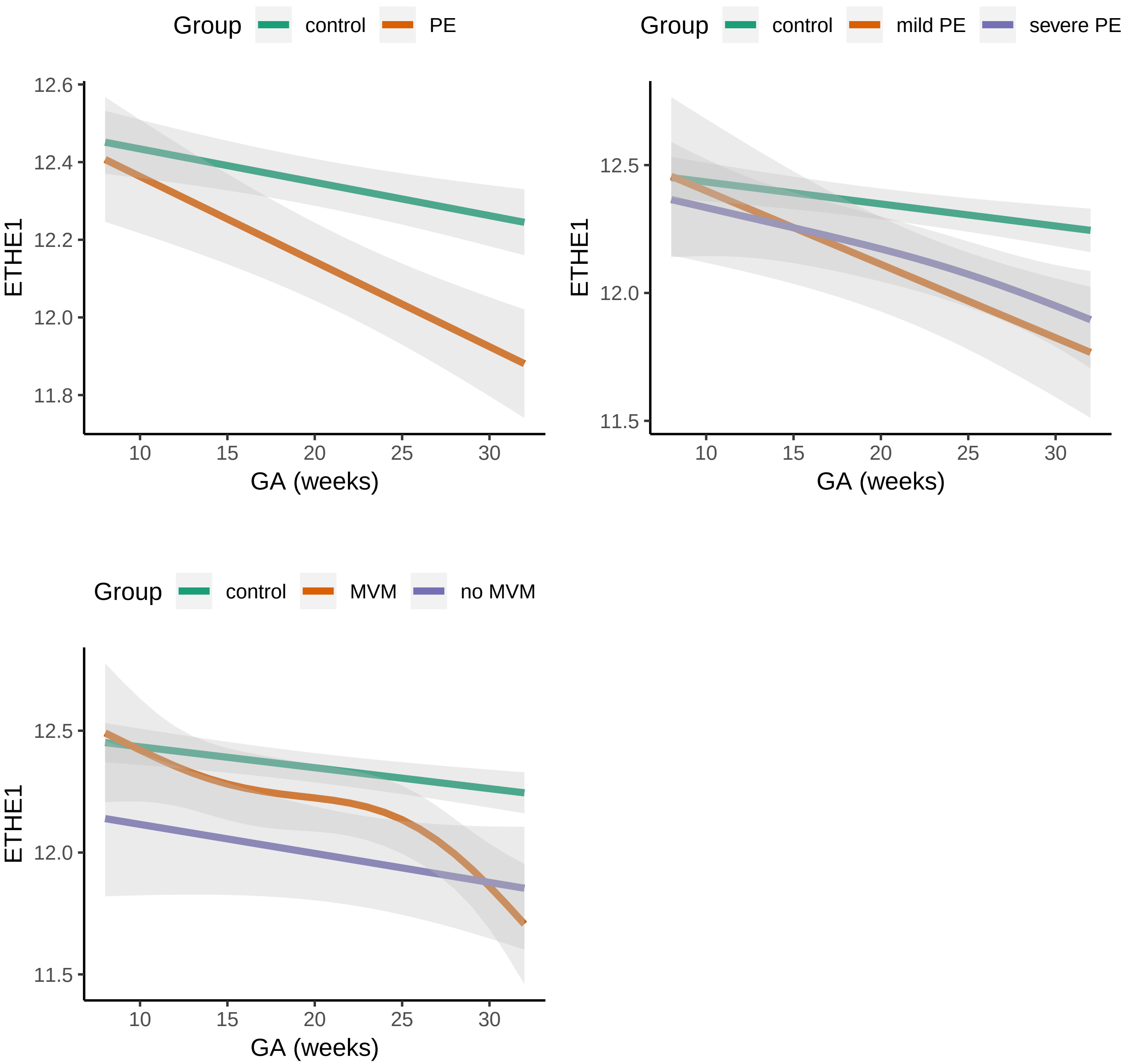

Group
control
PE
ETHE1
12.6
12.4
12.2
12.0
11.8
10
15
20
25
30
GA (weeks)
Group
control
mild PE
severe PE
ETHE1
12.5
12.0
11.5
10
15
20
25
30
GA (weeks)
Group
control
MVM
no MVM
ETHE1
12.5
12.0
11.5
10
15
20
25
30
GA (weeks)

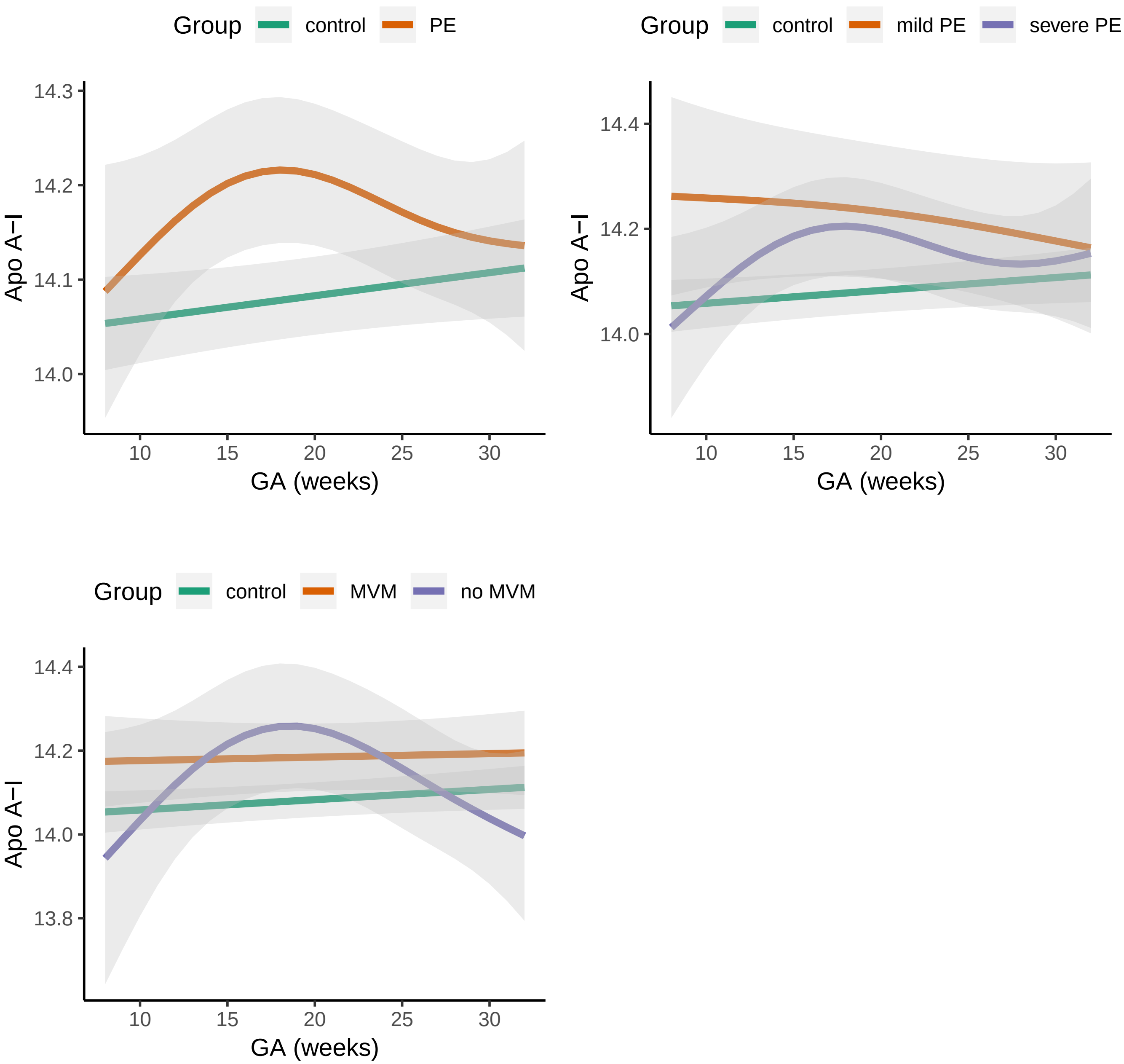

Group
control
PE
Apo A−I
14.3
14.2
14.1
14.0
10
15
20
25
30
GA (weeks)
Group
control
mild PE
severe PE
Apo A−I
14.4
14.2
14.0
10
15
20
25
30
GA (weeks)
Group
control
MVM
no MVM
Apo A−I
14.4
14.2
14.0
13.8
10
15
20
25
30
GA (weeks)

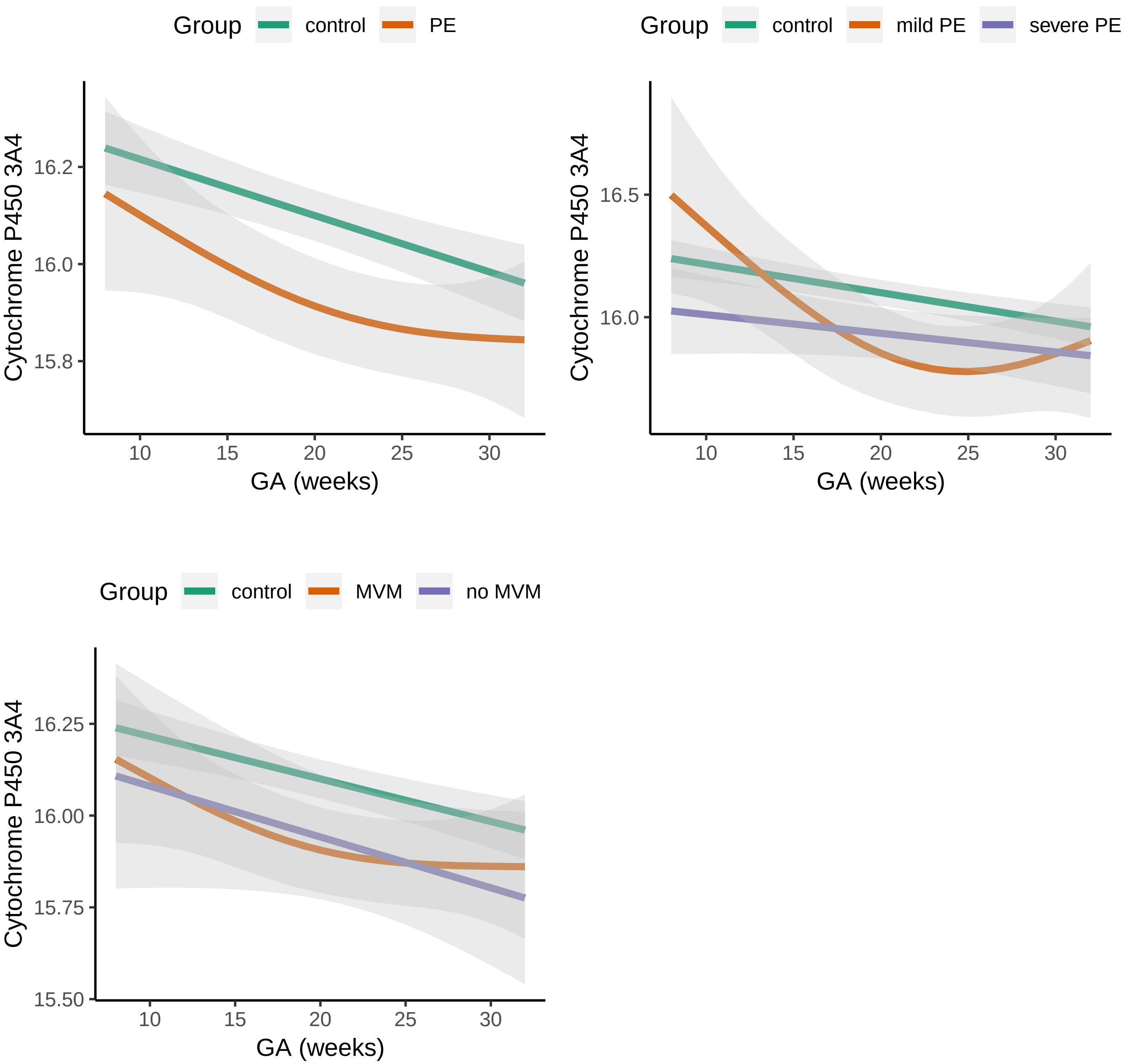
Group
control
PE
Cytochrome P450 3A4
16.2
16.0
15.8
10
15
20
25
30
GA (weeks)
Group
control
mild PE
severe PE
Cytochrome P450 3A4
16.5
16.0
10
15
20
25
30
GA (weeks)
Group
control
MVM
no MVM
Cytochrome P450 3A4
16.25
16.00
15.75
15.50
10
15
20
25
30
GA (weeks)

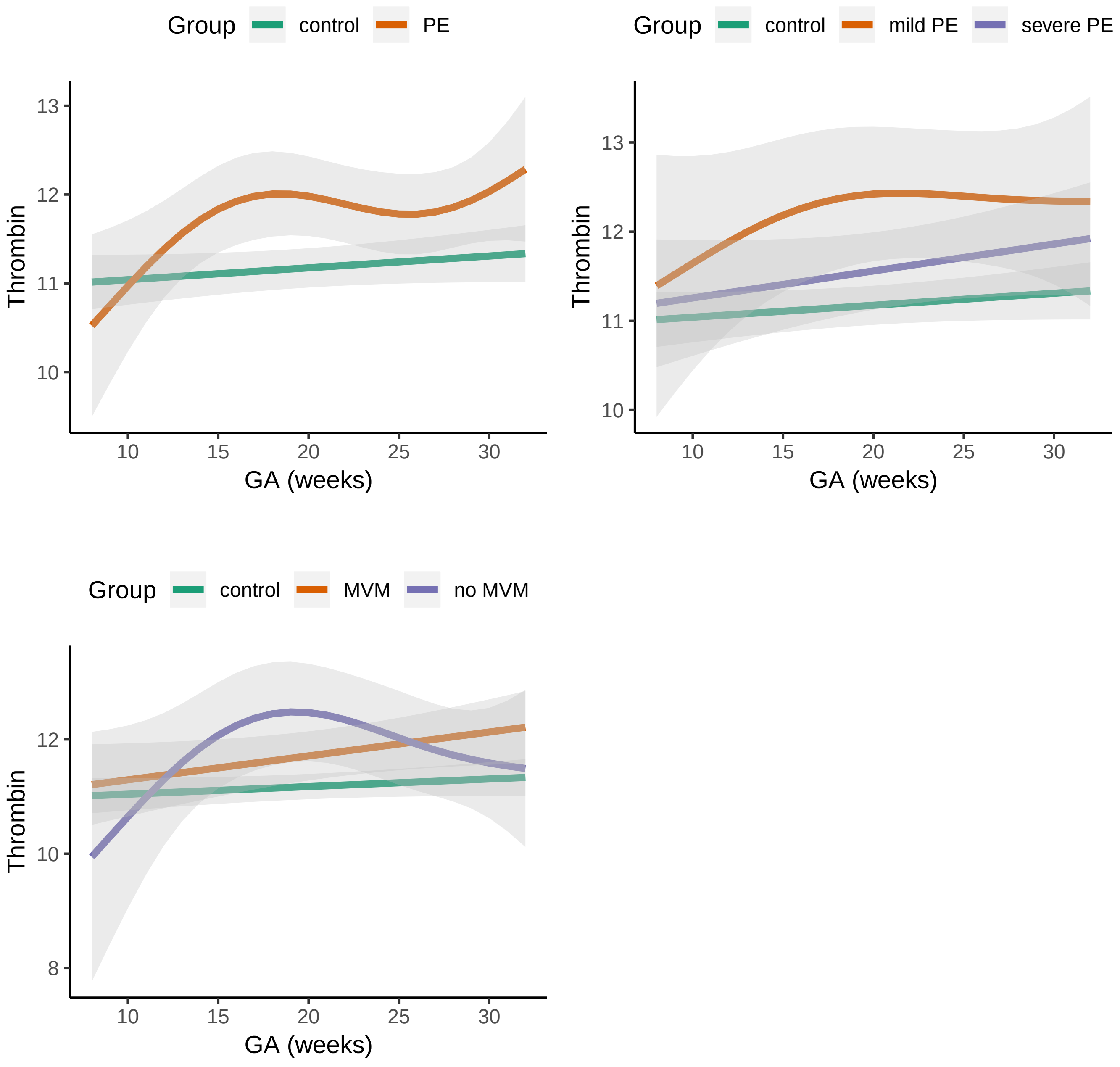
Group
control
PE
Thrombin
13
12
11
10
10
15
20
25
30
GA (weeks)
Group
control
mild PE
severe PE
Thrombin
13
12
11
10
10
15
20
25
30
GA (weeks)
Group
control
MVM
no MVM
Thrombin
12
10
8
10
15
20
25
30
GA (weeks)

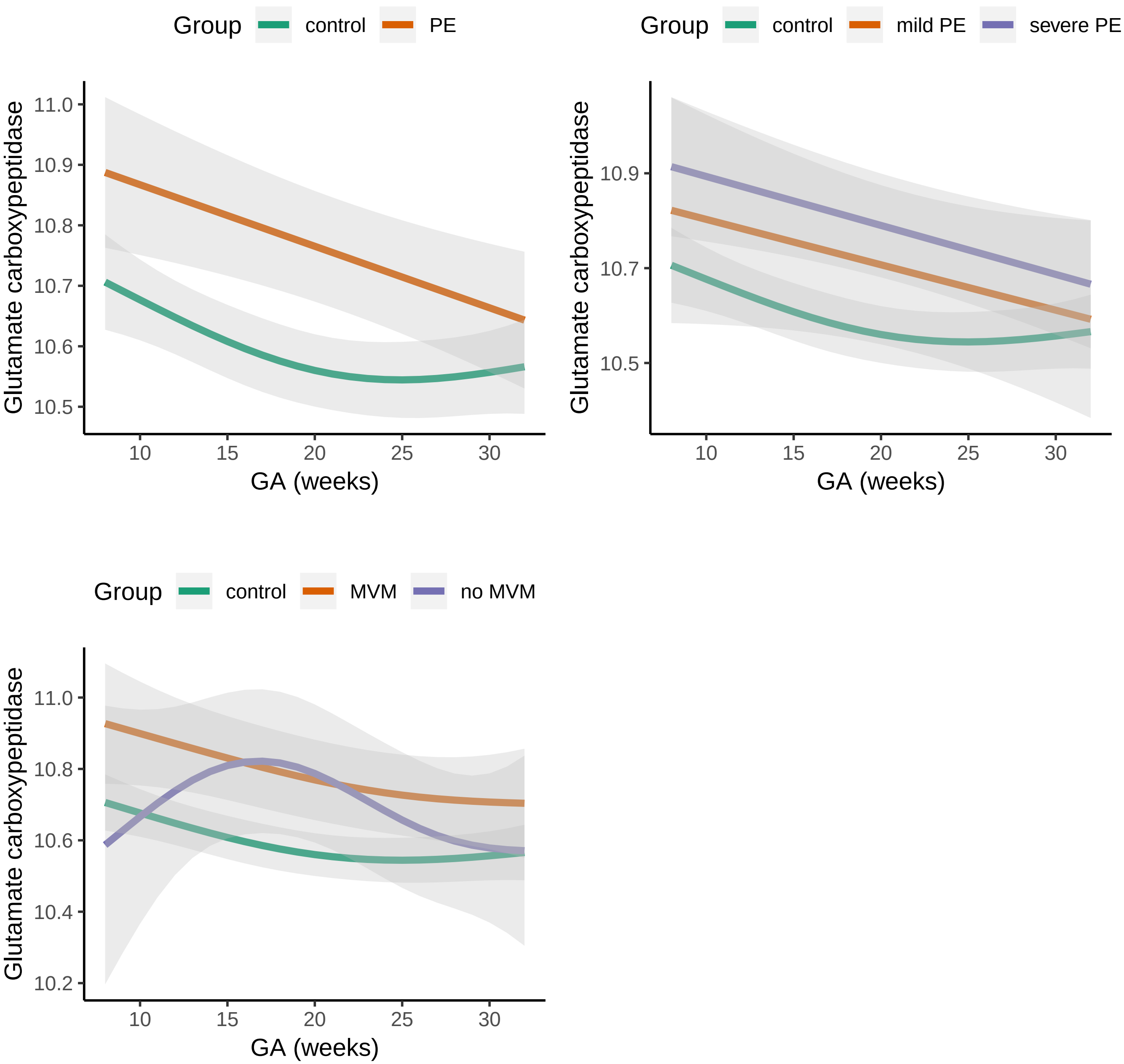
Group
control
PE
Glutamate carboxypeptidase
11.0
10.9
10.8
10.7
10.6
10.5
10
15
20
25
30
GA (weeks)
Group
control
mild PE
severe PE
Glutamate carboxypeptidase
10.9
10.7
10.5
10
15
20
25
30
GA (weeks)
Group
control
MVM
no MVM
Glutamate carboxypeptidase
11.0
10.8
10.6
10.4
10.2
10
15
20
25
30
GA (weeks)

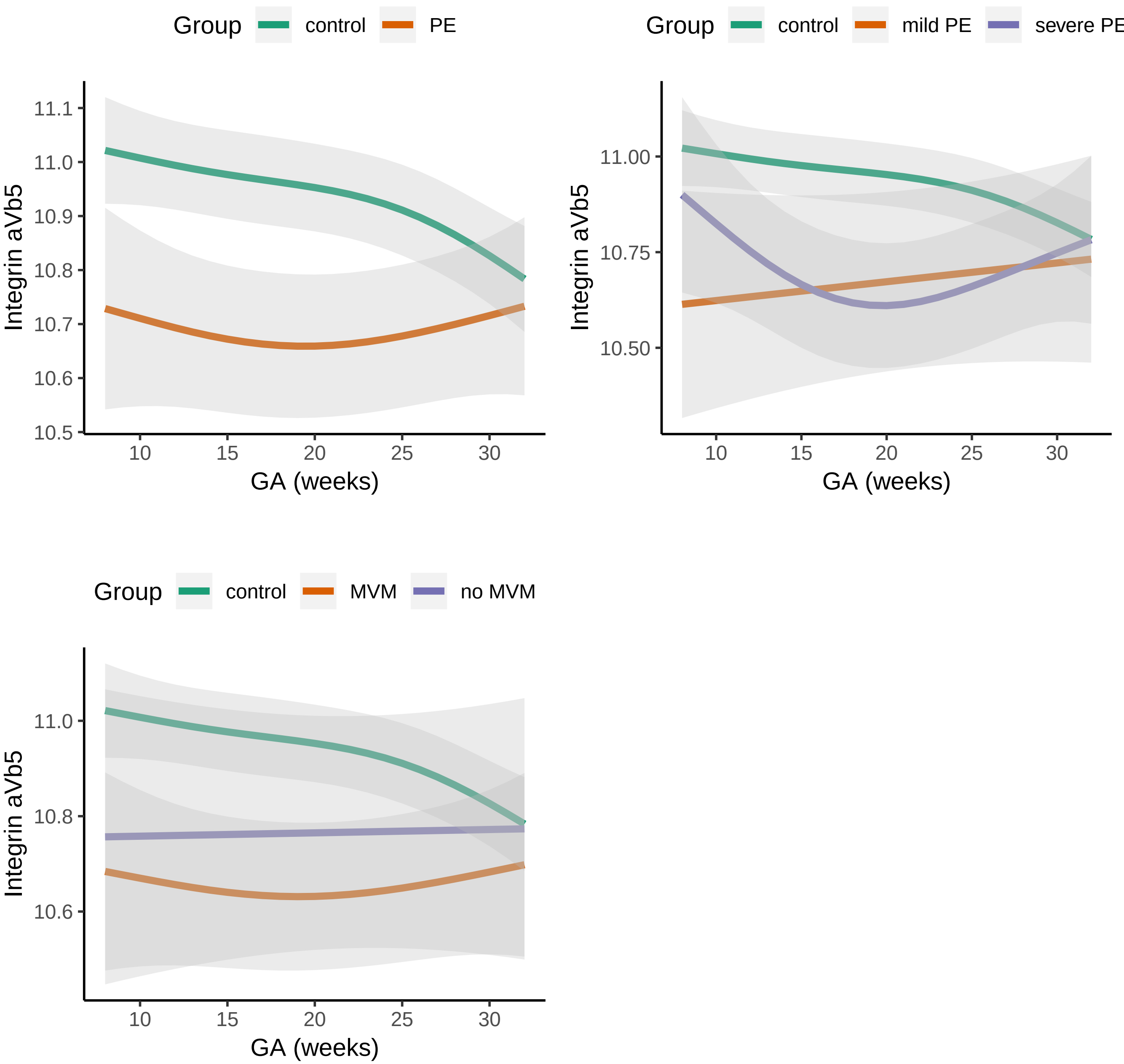
Group
control
PE
Integrin aVb5
11.1
11.0
10.9
10.8
10.7
10.6
10.5
10
15
20
25
30
GA (weeks)
Group
control
mild PE
severe PE
Integrin aVb5
11.00
10.75
10.50
10
15
20
25
30
GA (weeks)
Group
control
MVM
no MVM
Integrin aVb5
11.0
10.8
10.6
10
15
20
25
30
GA (weeks)

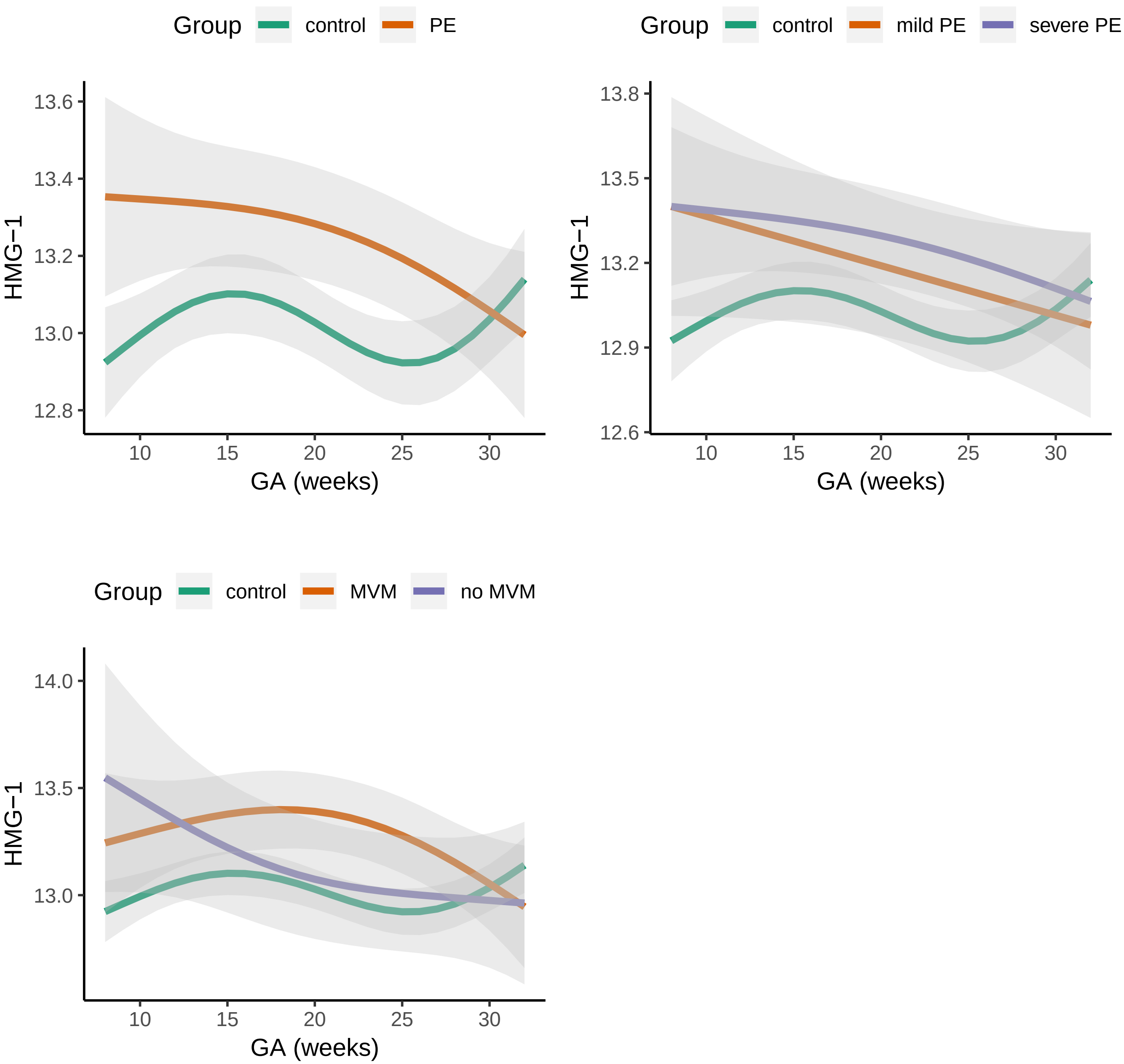
Group
control
PE
mild PE
severe PE
MVM
no MVM
HMG−1
GA (weeks)
13.6
13.4
13.2
13.0
12.8
13.8
13.5
12.9
12.6
14.0
10
15
20
25
30

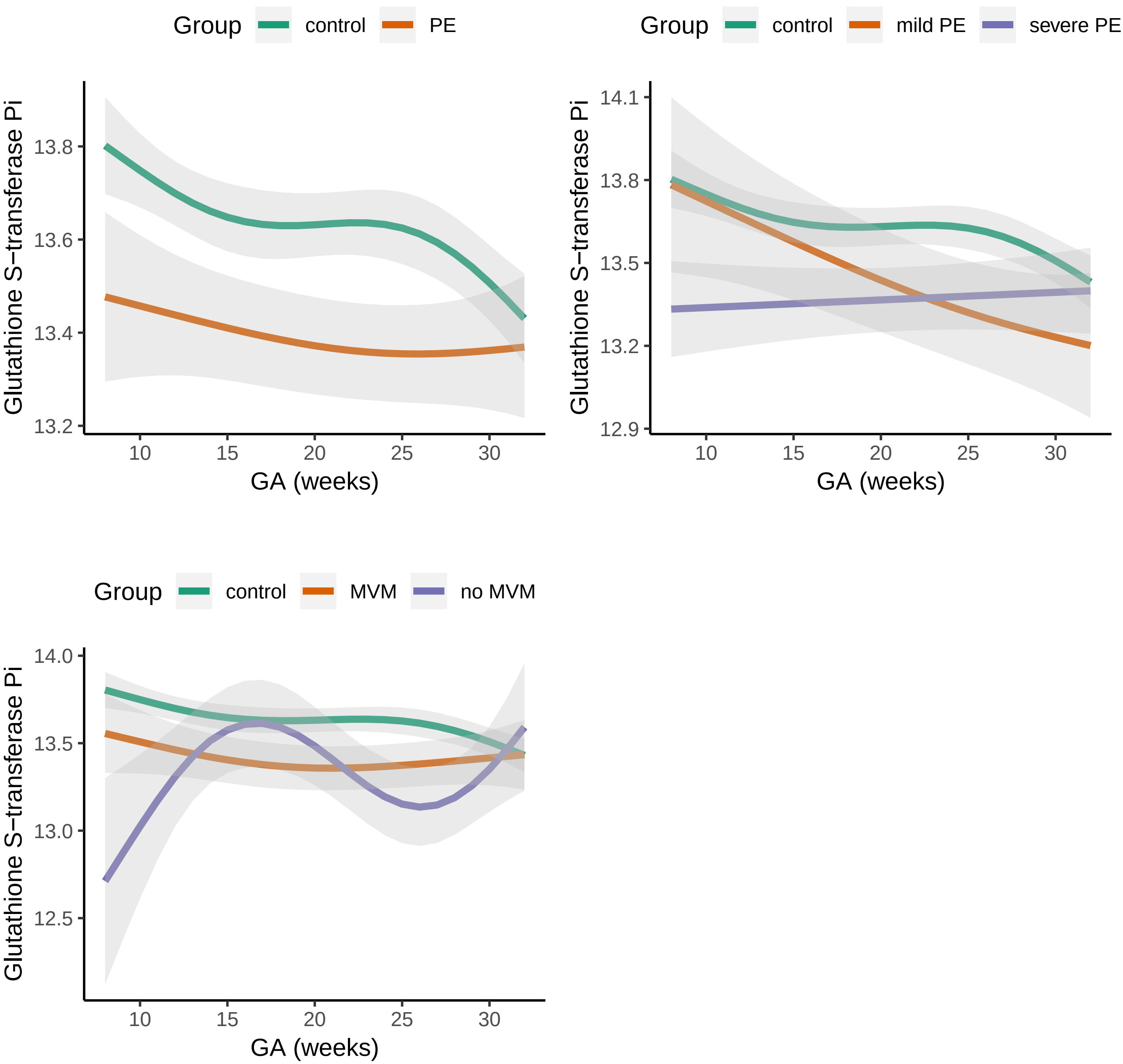

Group
control
PE
Glutathione S−transferase Pi
13.2
13.4
13.6
13.8
10
15
20
25
30
GA (weeks)
Group
control
mild PE
severe PE
Glutathione S−transferase Pi
12.9
13.2
13.5
13.8
14.1
10
15
20
25
30
GA (weeks)
Group
control
MVM
no MVM
Glutathione S−transferase Pi
12.5
13.0
13.5
14.0
10
15
20
25
30
GA (weeks)

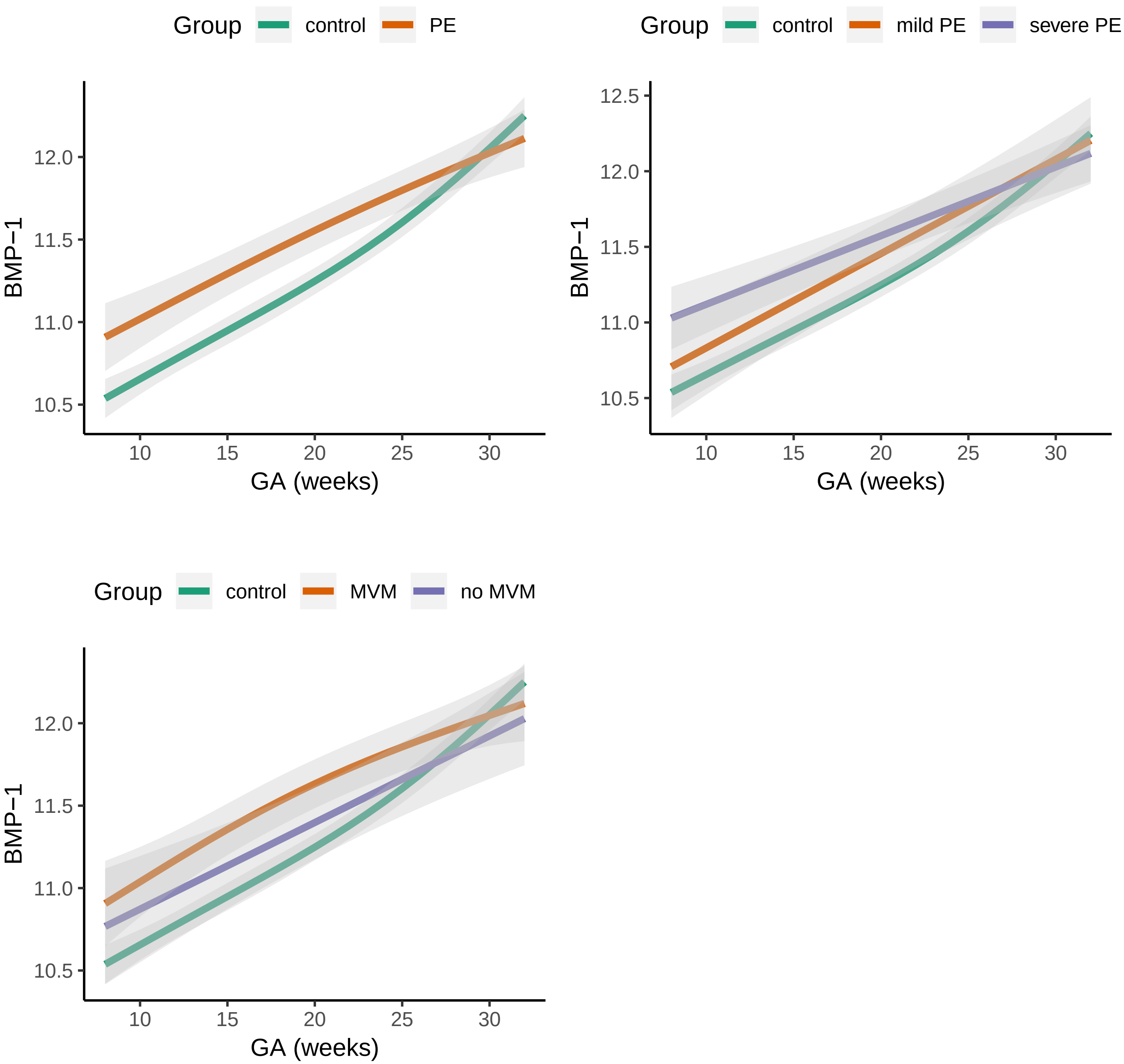
Group
control
PE
Group
control
mild PE
severe PE
Group
control
MVM
no MVM
BMP−1
GA (weeks)
10.5
11.0
11.5
12.0
12.5
10
15
20
25
30

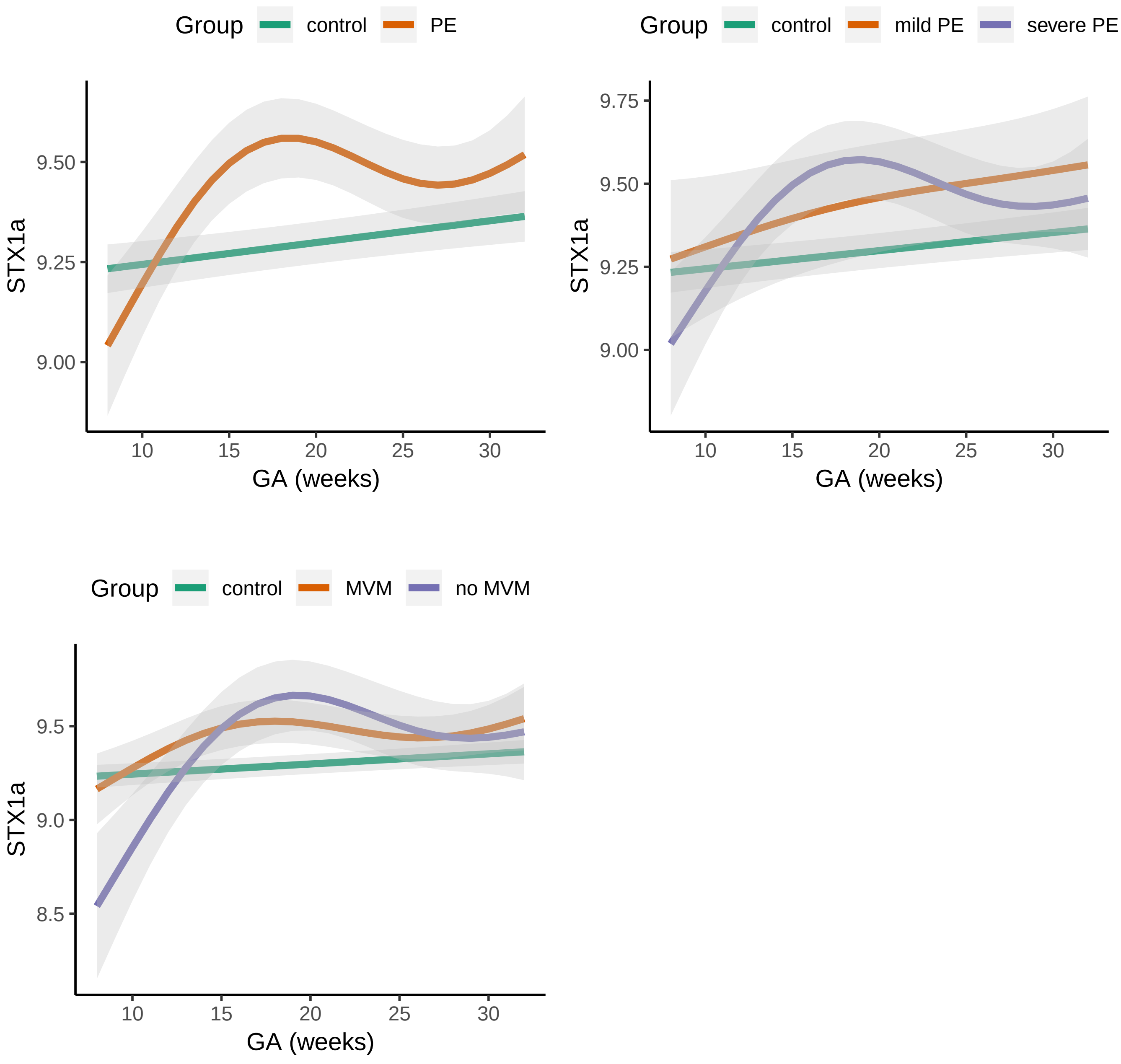

Group
control
PE
STX1a
9.50
9.25
9.00
10
15
20
25
30
GA (weeks)
Group
control
mild PE
severe PE
STX1a
9.75
9.50
9.25
9.00
10
15
20
25
30
GA (weeks)
Group
control
MVM
no MVM
STX1a
9.5
9.0
8.5
10
15
20
25
30
GA (weeks)

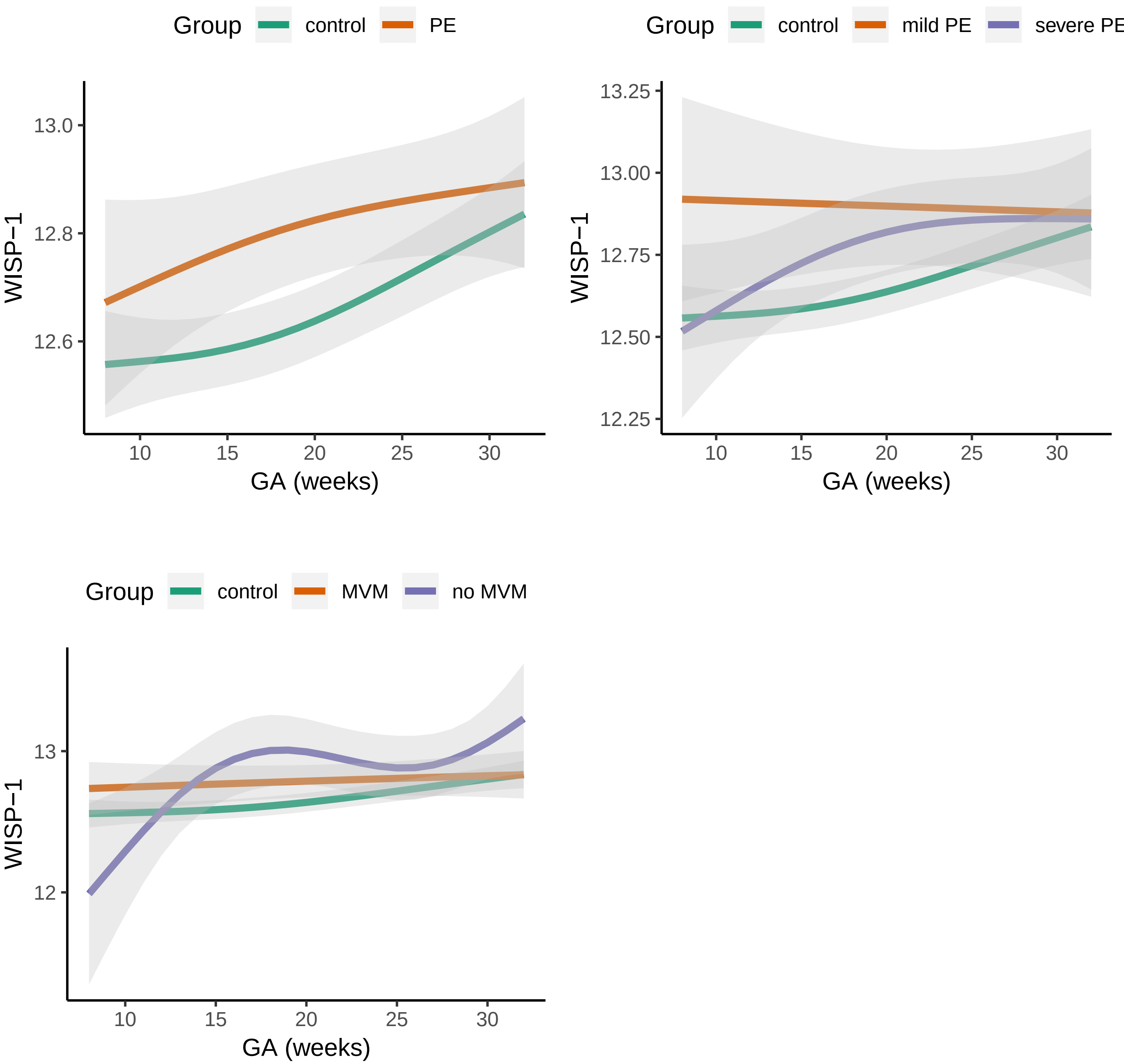

Group
control
PE
WISP−1
13.0
12.8
12.6
10
15
20
25
30
GA (weeks)
Group
control
mild PE
severe PE
WISP−1
13.25
13.00
12.75
12.50
12.25
10
15
20
25
30
GA (weeks)
Group
control
MVM
no MVM
WISP−1
13
12
10
15
20
25
30
GA (weeks)

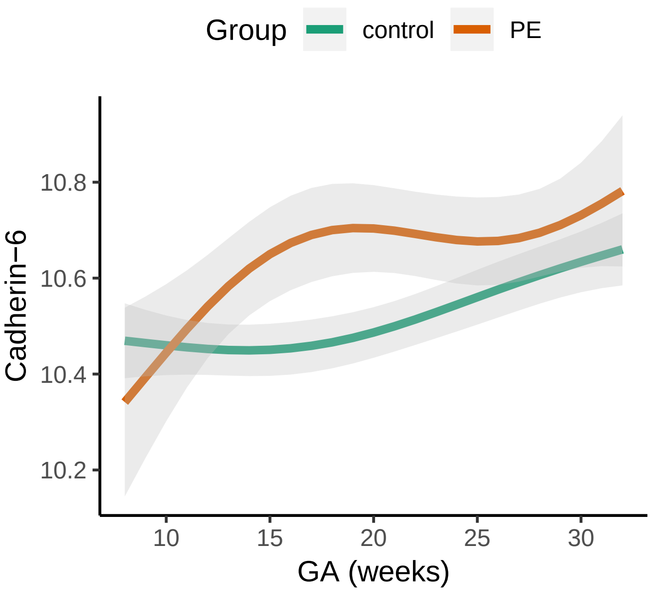

Group
control
PE
10.8
10.6
10.4
10.2
Cadherin−6
10
15
20
25
30
GA (weeks)

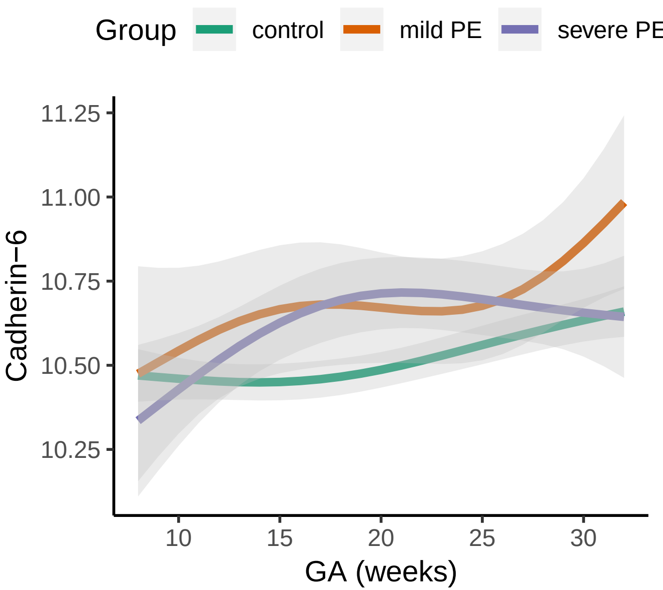

Group
control
mild PE
severe PE
11.25
11.00
10.75
10.50
10.25
Cadherin−6
10
15
20
25
30
GA (weeks)

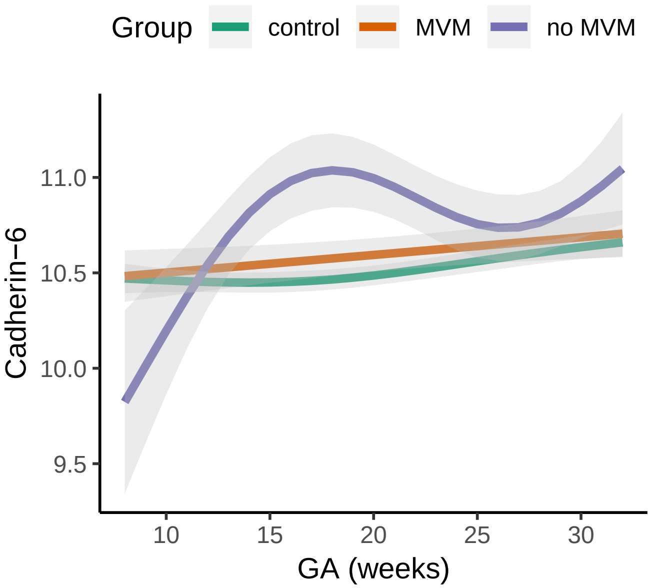

Group
control
MVM
no MVM
11.0
10.5
10.0
9.5
Cadherin−6
10
15
20
25
30
GA (weeks)

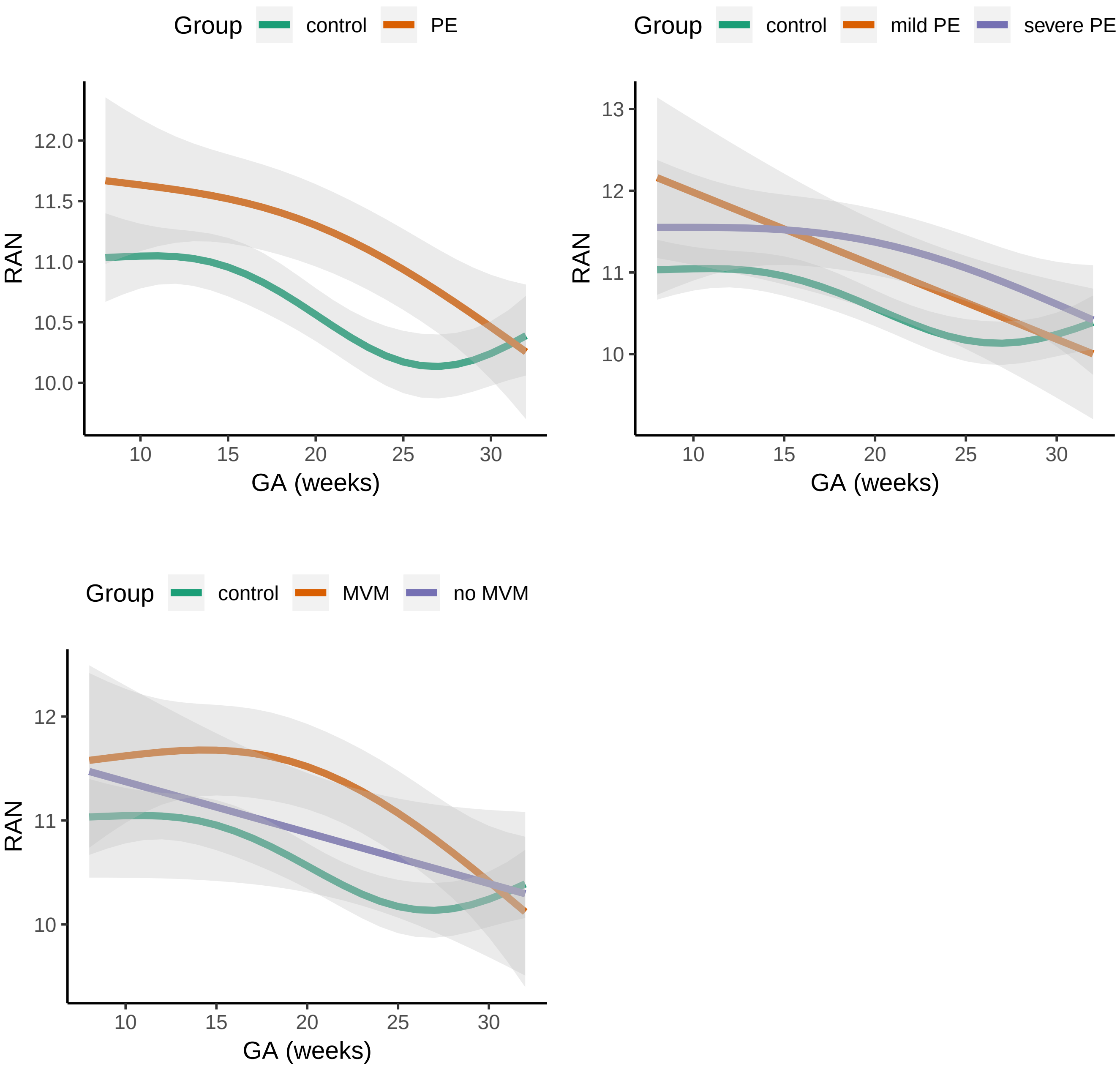
Group
control
PE
RAN
GA (weeks)
Group
control
mild PE
severe PE
RAN
GA (weeks)
Group
control
MVM
no MVM
RAN
GA (weeks)

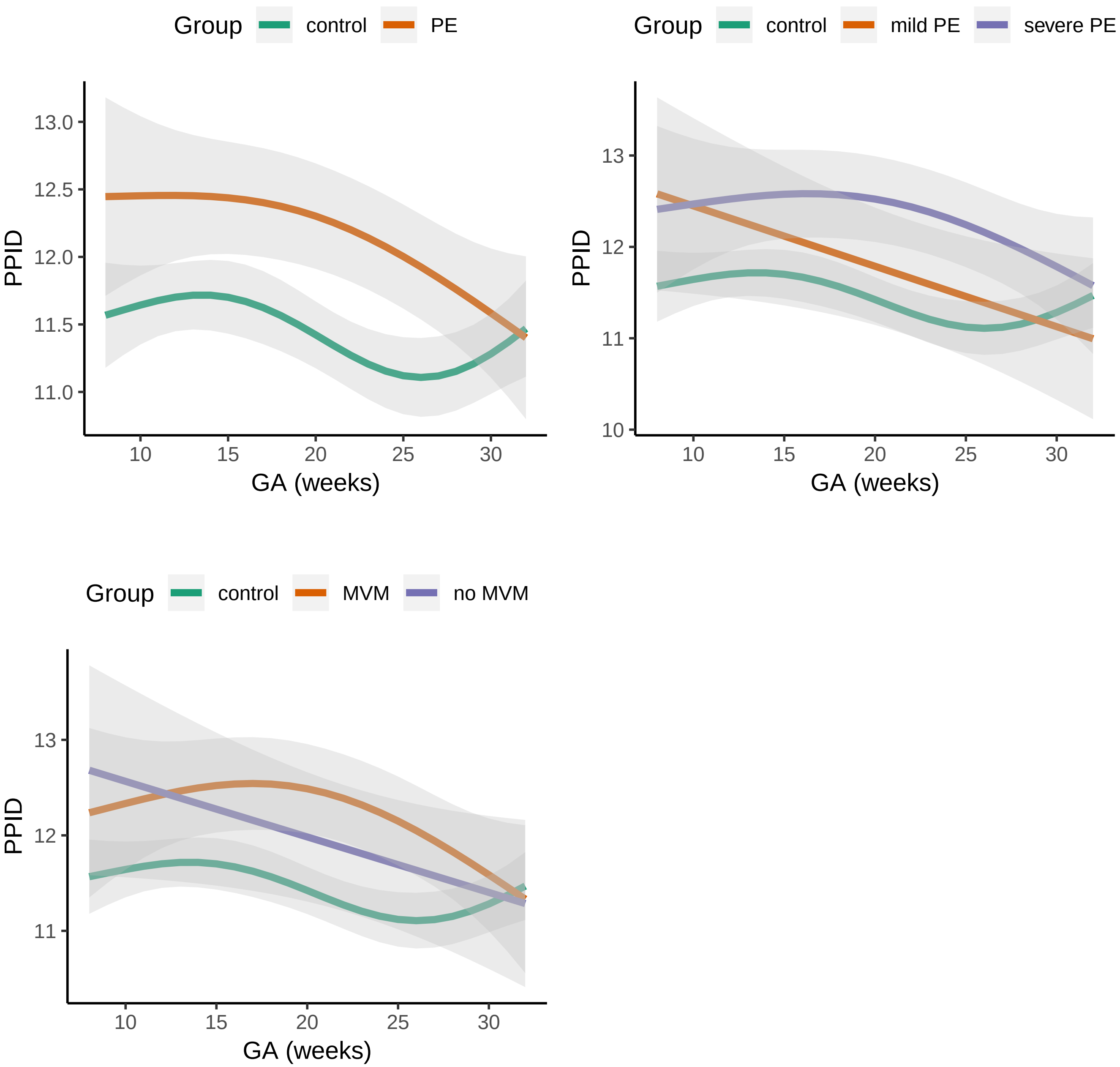
Group control PE
PPID
13.0
12.5
12.0
11.5
11.0
10
15
20
25
30
GA (weeks)
Group control mild PE severe PE
PPID
13
12
11
10
10
15
20
25
30
GA (weeks)
Group control MVM no MVM
PPID
13
12
11
10
15
20
25
30
GA (weeks)

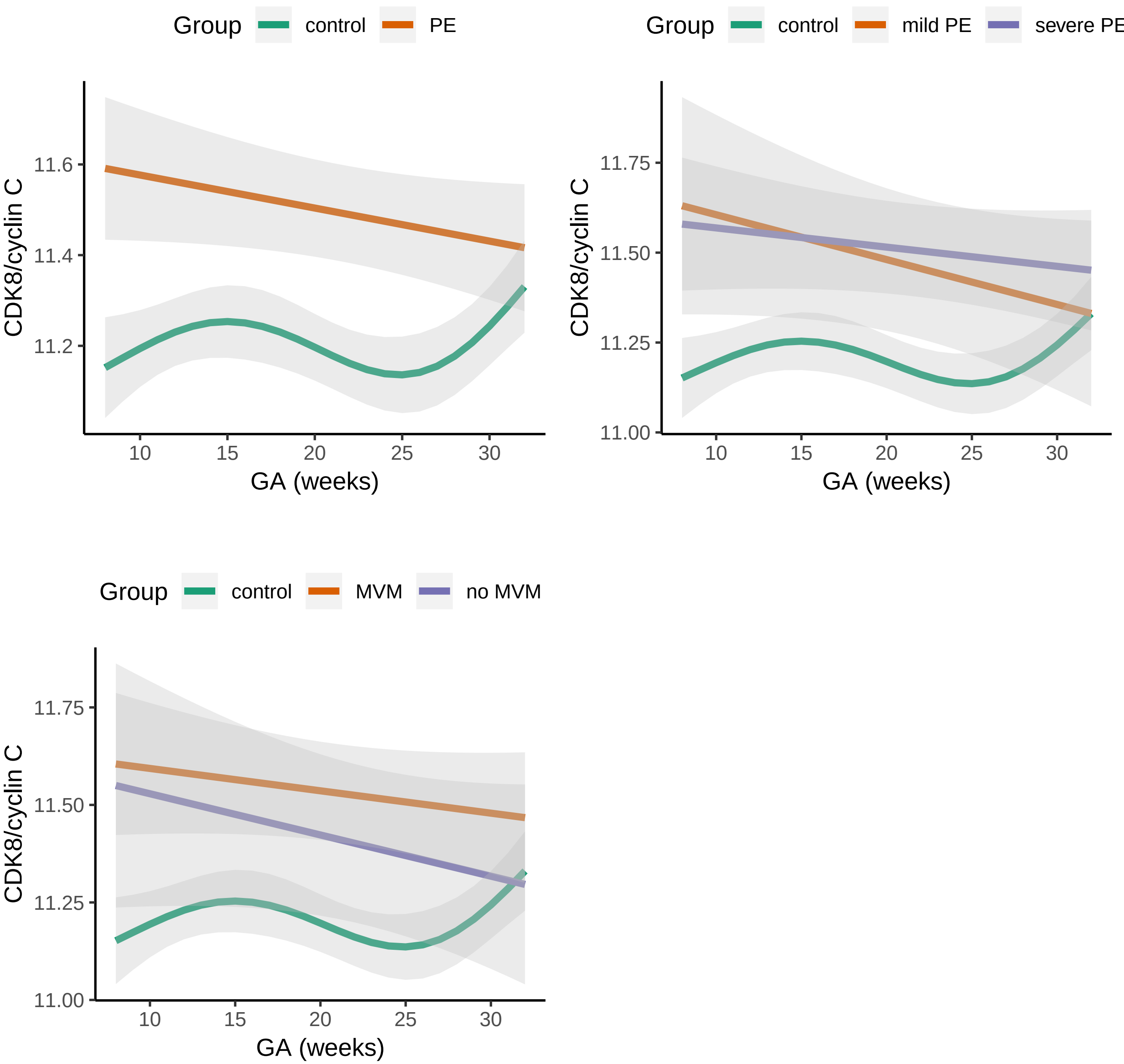

Group
control
PE
CDK8/cyclin C
11.2
11.4
11.6
10
15
20
25
30
GA (weeks)
Group
control
mild PE
severe PE
CDK8/cyclin C
11.00
11.25
11.50
11.75
10
15
20
25
30
GA (weeks)
Group
control
MVM
no MVM
CDK8/cyclin C
11.00
11.25
11.50
11.75
10
15
20
25
30
GA (weeks)

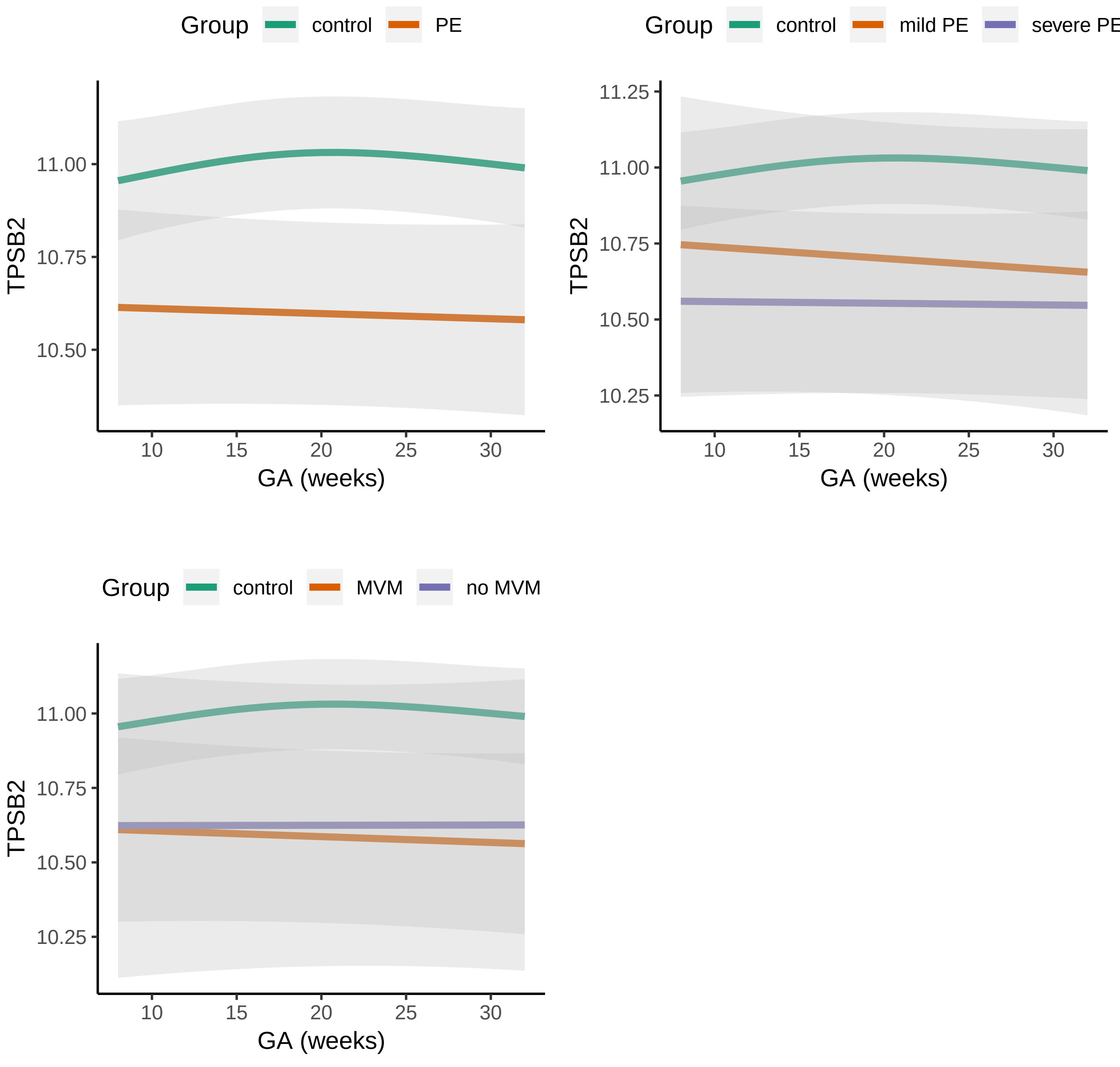
Group
control
PE
TPSB2
11.00
10.75
10.50
10
15
20
25
30
GA (weeks)
Group
control
mild PE
severe PE
TPSB2
11.25
11.00
10.75
10.50
10.25
10
15
20
25
30
GA (weeks)
Group
control
MVM
no MVM
TPSB2
11.00
10.75
10.50
10.25
10
15
20
25
30
GA (weeks)

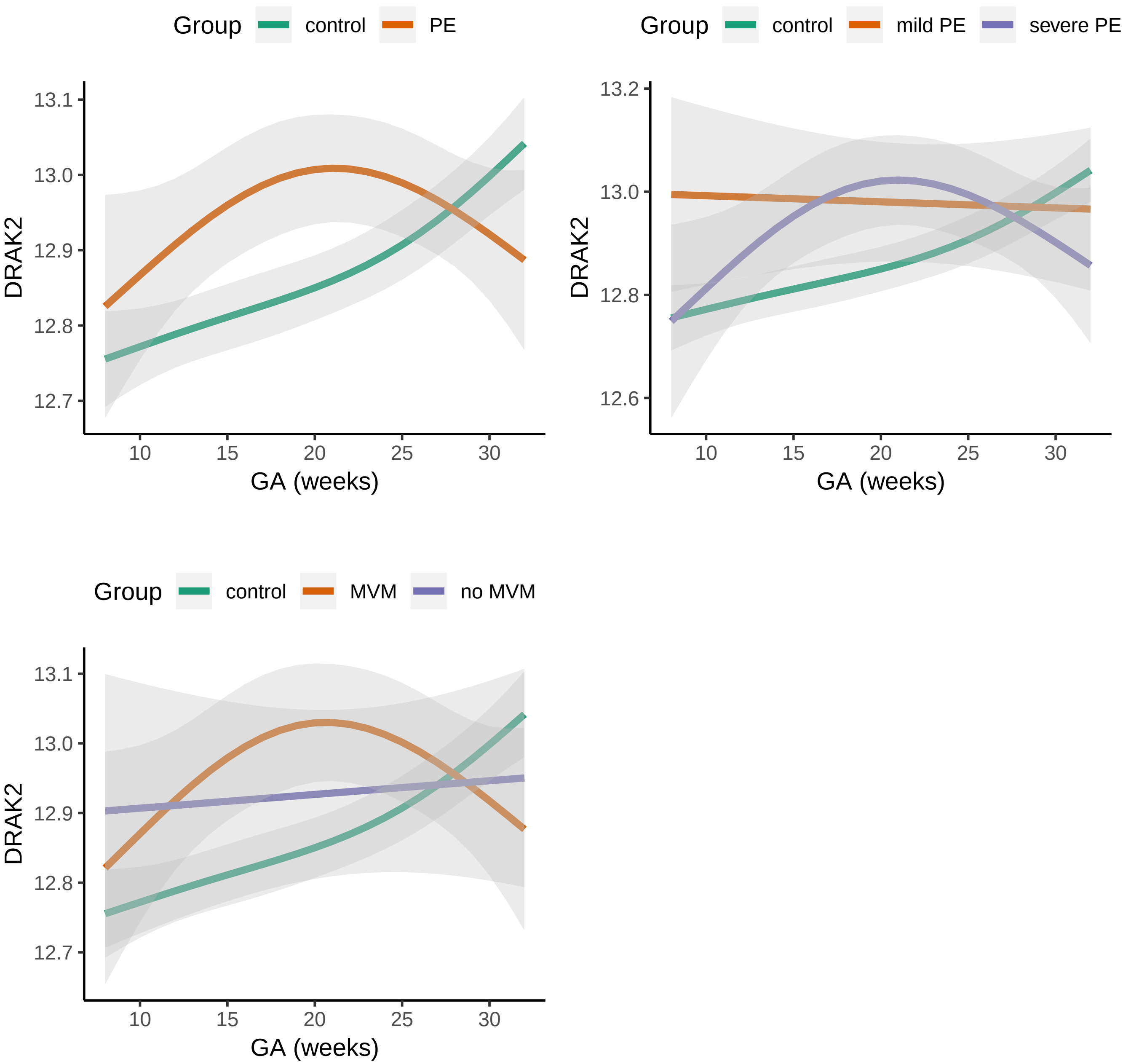

Group
control
PE
mild PE
severe PE
MVM
no MVM
DRAK2
GA (weeks)
13.2
13.1
13.0
12.9
12.8
12.7
12.6
10
15
20
25
30

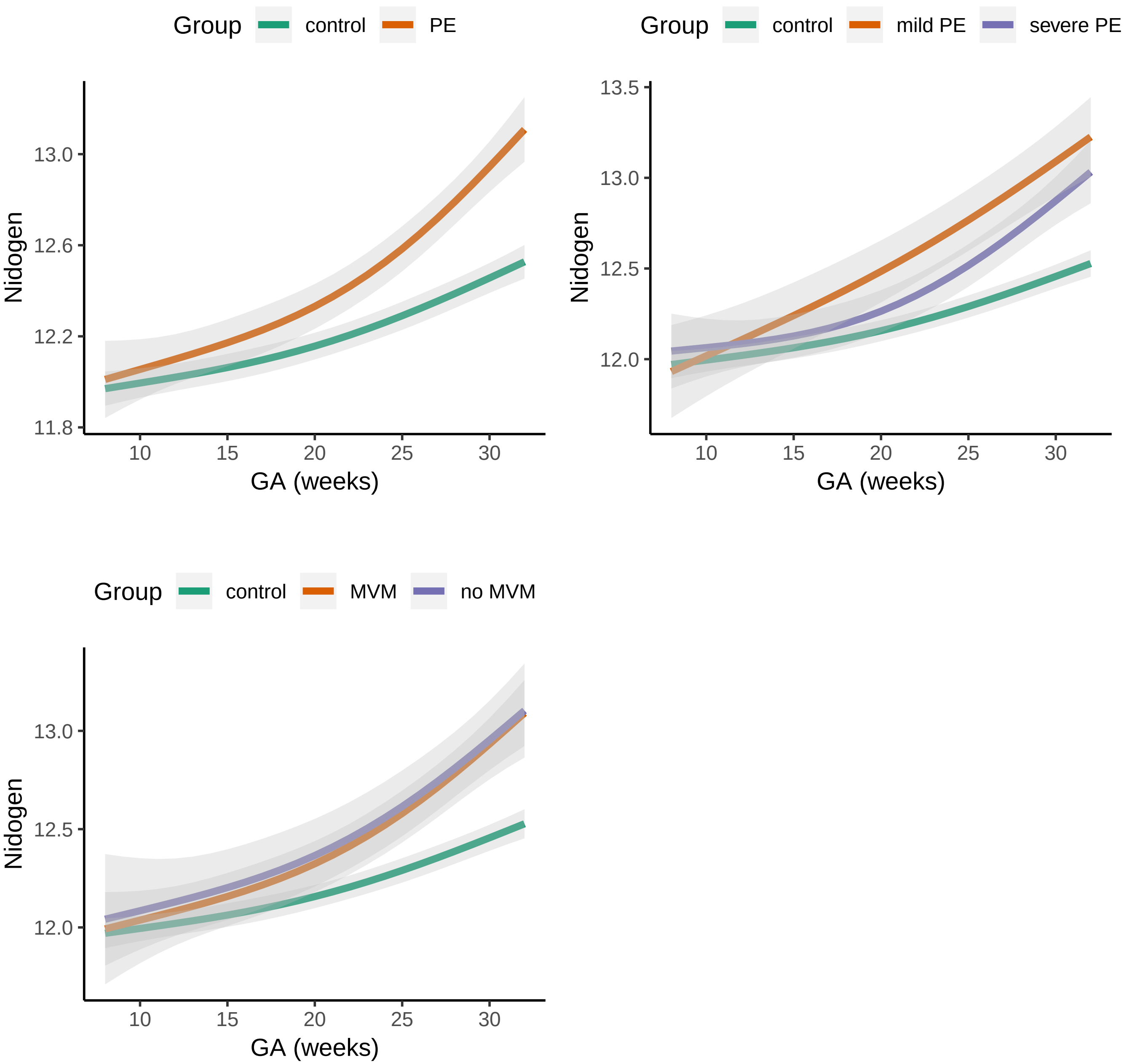

Group
control
PE
Nidogen
11.8
12.2
12.6
13.0
GA (weeks)
10
15
20
25
30
Group
control
mild PE
severe PE
Nidogen
12.0
12.5
13.0
13.5
GA (weeks)
10
15
20
25
30
Group
control
MVM
no MVM
Nidogen
12.0
12.5
13.0
GA (weeks)
10
15
20
25
30

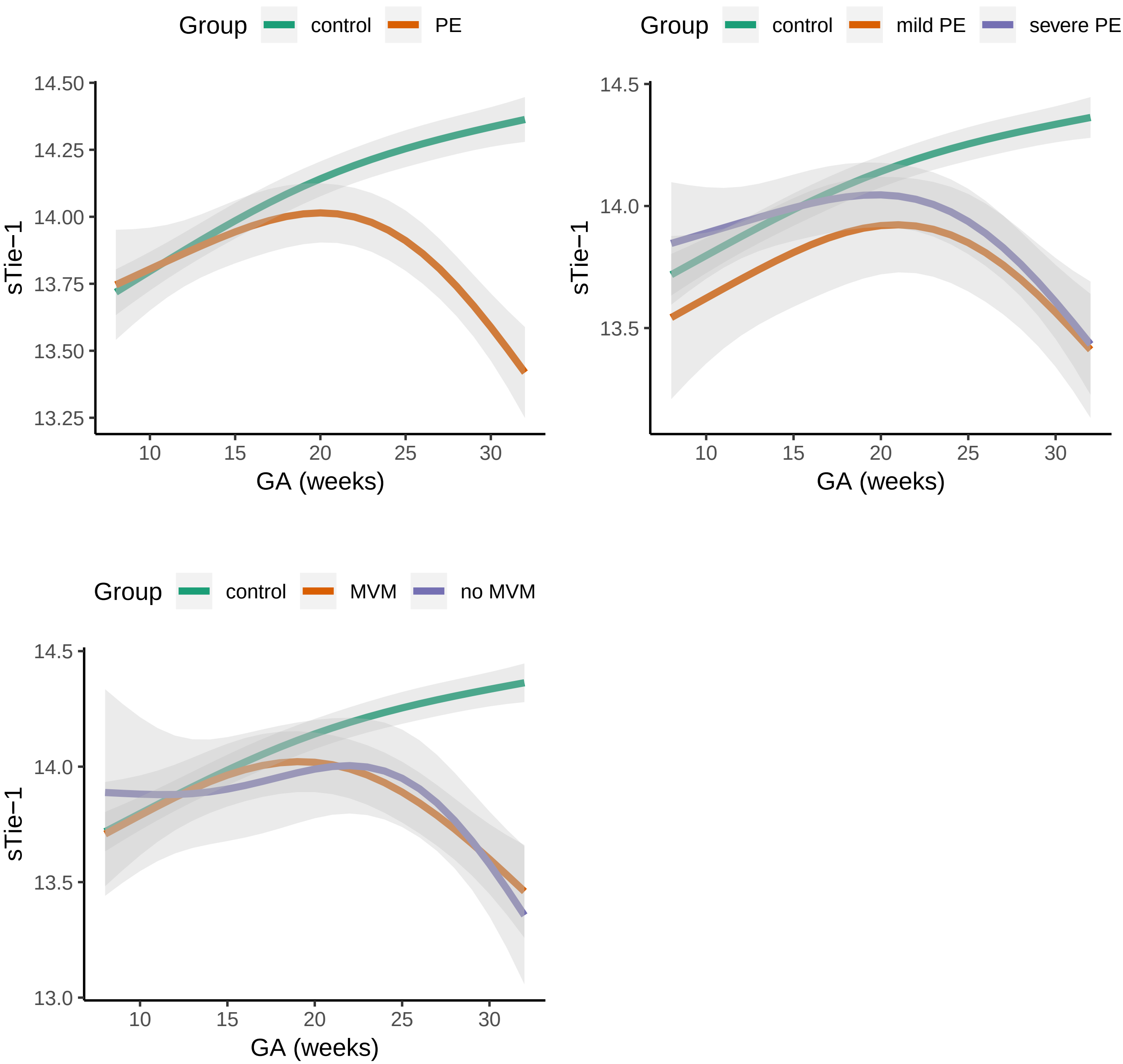

Group
control
PE
14.50
14.25
14.00
13.75
13.50
13.25
sTie−1
10
15
20
25
30
GA (weeks)
Group
control
mild PE
severe PE
14.5
14.0
13.5
sTie−1
10
15
20
25
30
GA (weeks)
Group
control
MVM
no MVM
14.5
14.0
13.5
13.0
sTie−1
10
15
20
25
30
GA (weeks)

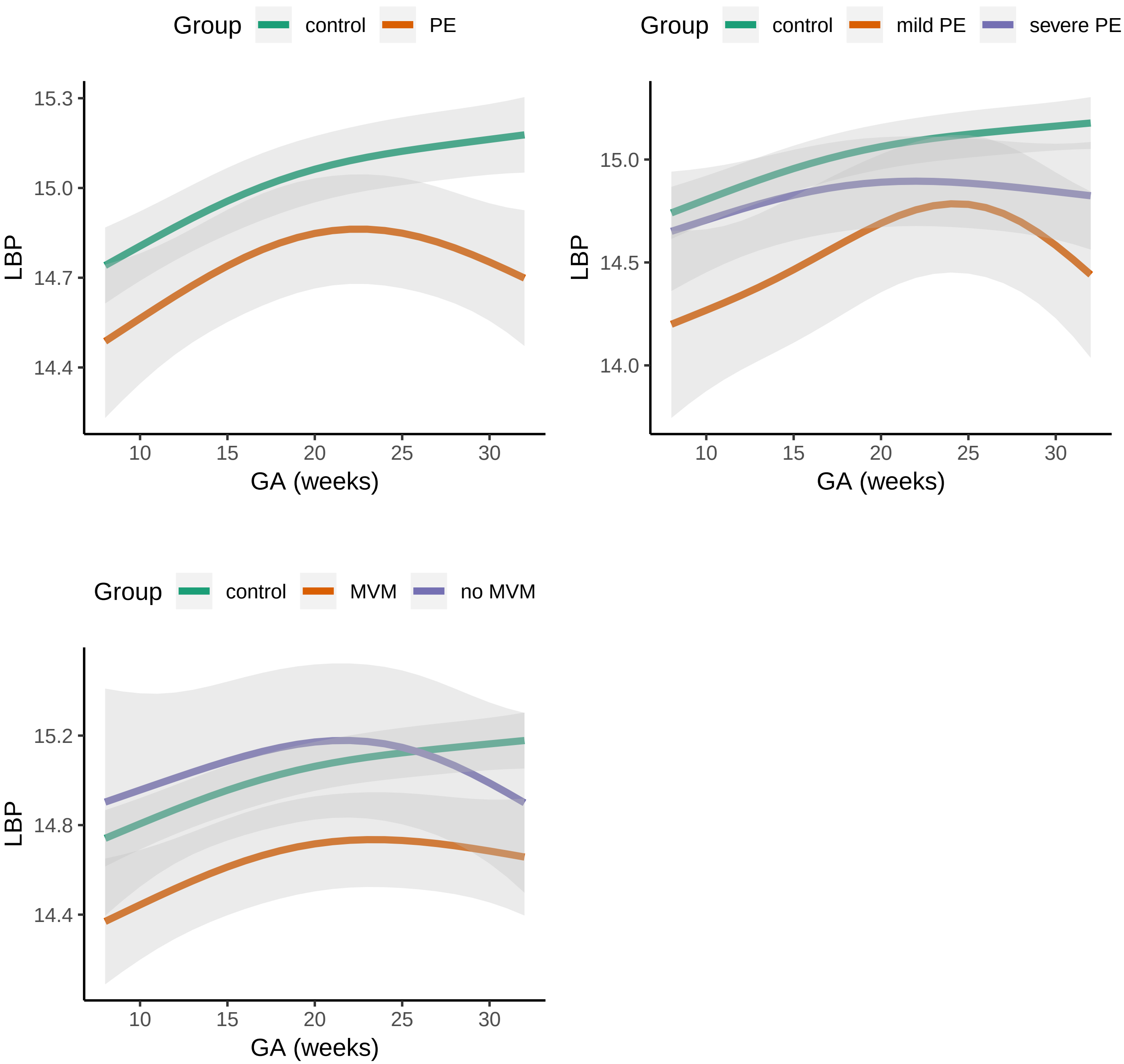

Group
control
PE
LBP
15.3
15.0
14.7
14.4
10
15
20
25
30
GA (weeks)
Group
control
mild PE
severe PE
LBP
15.0
14.5
14.0
10
15
20
25
30
GA (weeks)
Group
control
MVM
no MVM
LBP
15.2
14.8
14.4
10
15
20
25
30
GA (weeks)

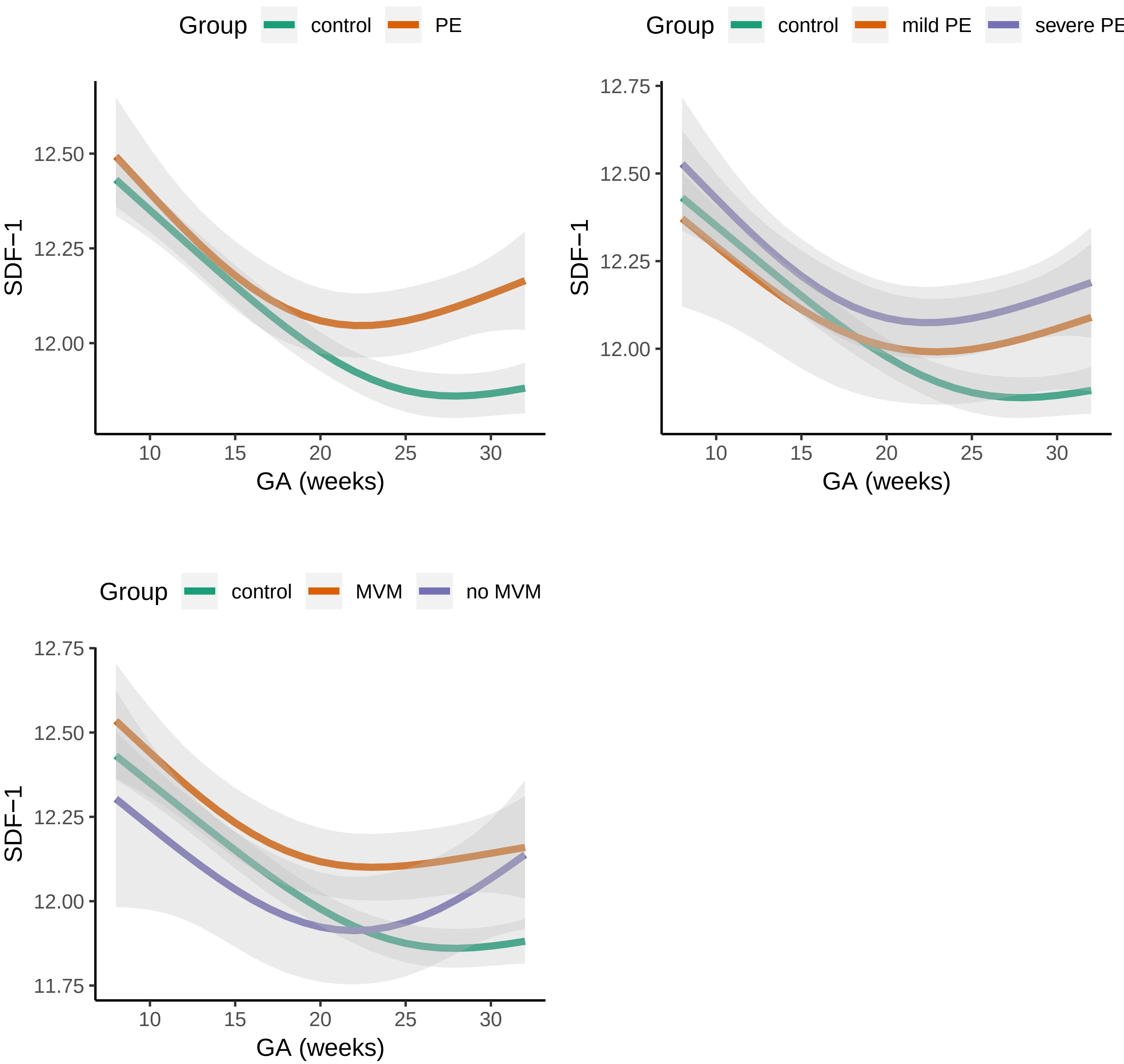
Group control PE
SDF−1
12.50
12.25
12.00
10 15 20 25 30
GA (weeks)
Group control mild PE severe PE
SDF−1
12.75
12.50
12.25
12.00
10 15 20 25 30
GA (weeks)
Group control MVM no MVM
SDF−1
12.75
12.50
12.25
12.00
11.75
10 15 20 25 30
GA (weeks)

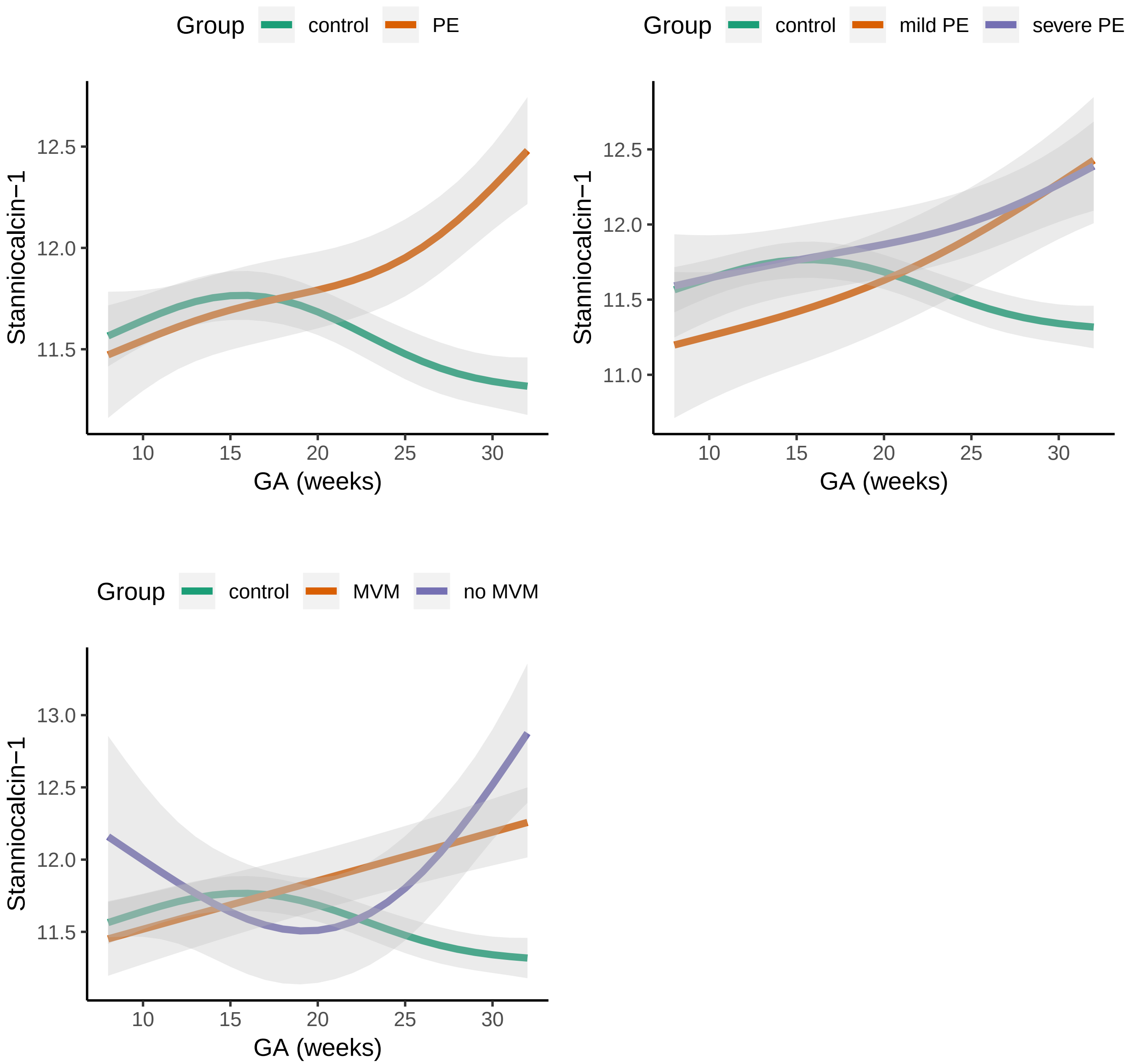
Group
control
PE
Stanniocalcin−1
GA (weeks)
Group
control
mild PE
severe PE
Stanniocalcin−1
GA (weeks)
Group
control
MVM
no MVM
Stanniocalcin−1
GA (weeks)

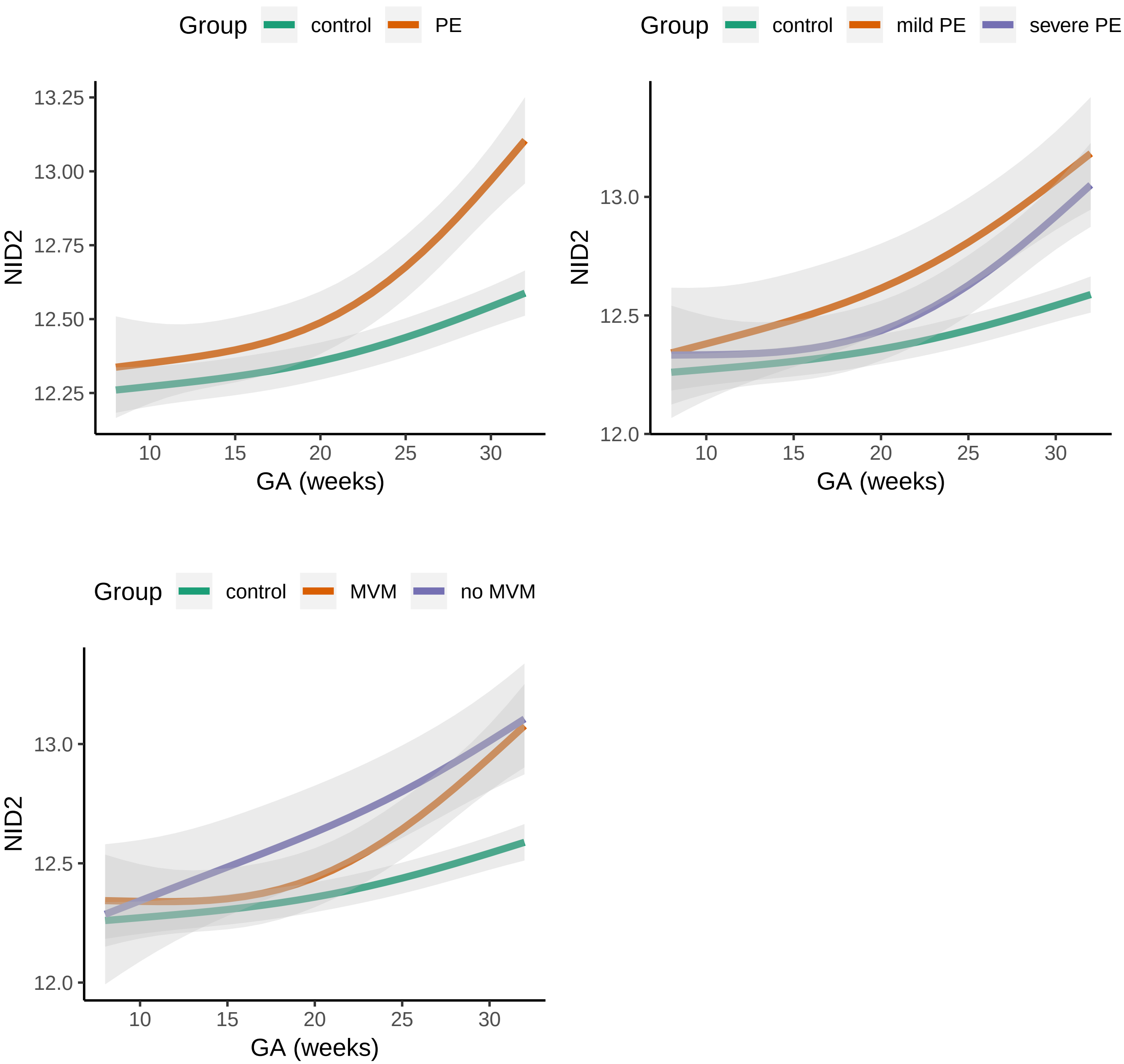
Group
control
PE
NID2
13.25
13.00
12.75
12.50
12.25
10
15
20
25
30
GA (weeks)
Group
control
mild PE
severe PE
NID2
13.0
12.5
12.0
10
15
20
25
30
GA (weeks)
Group
control
MVM
no MVM
NID2
13.0
12.5
12.0
10
15
20
25
30
GA (weeks)

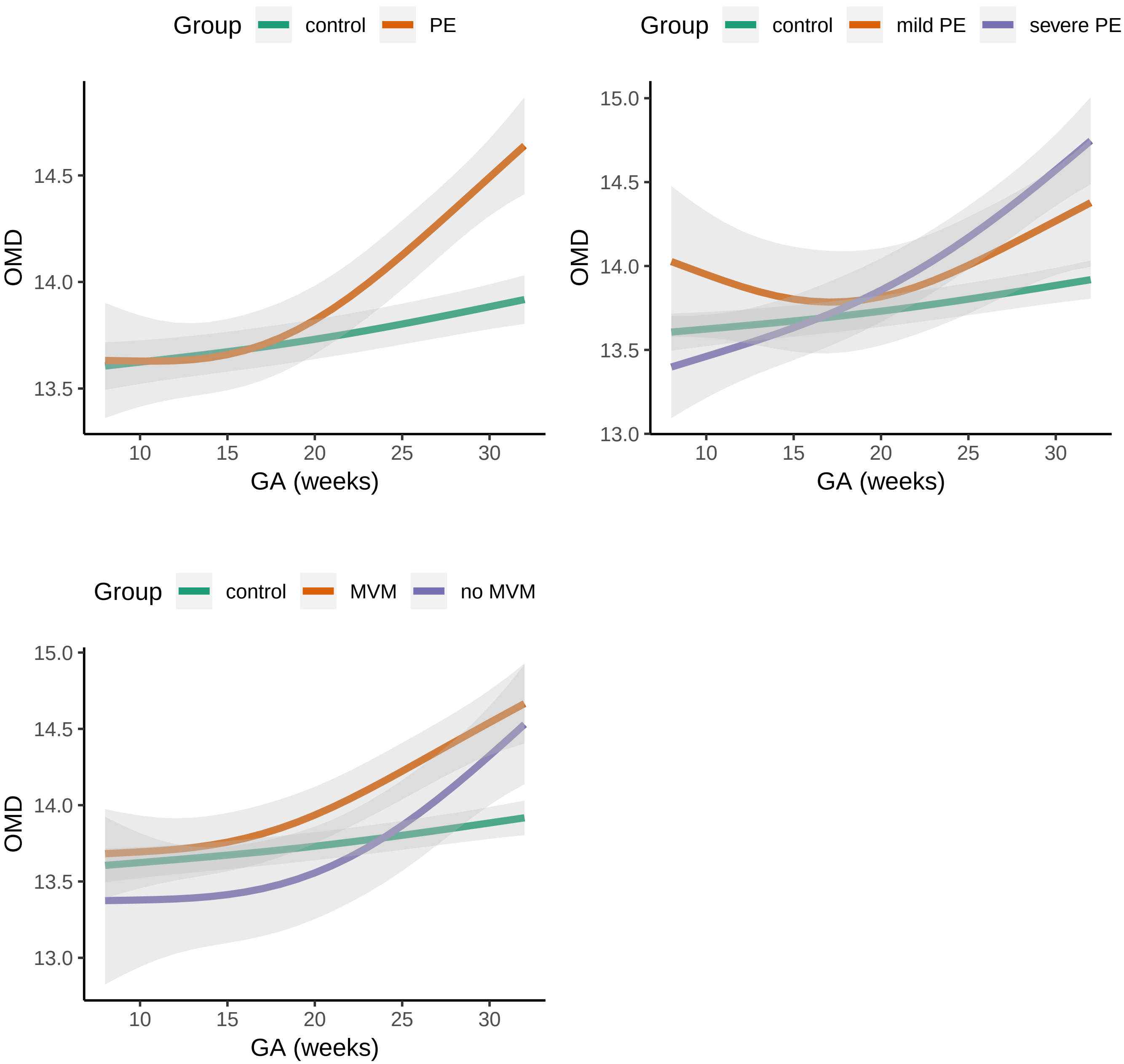

Group
control
PE
OMD
14.5
14.0
13.5
10
15
20
25
30
GA (weeks)
Group
control
mild PE
severe PE
OMD
15.0
14.5
14.0
13.5
13.0
10
15
20
25
30
GA (weeks)
Group
control
MVM
no MVM
OMD
15.0
14.5
14.0
13.5
13.0
10
15
20
25
30
GA (weeks)

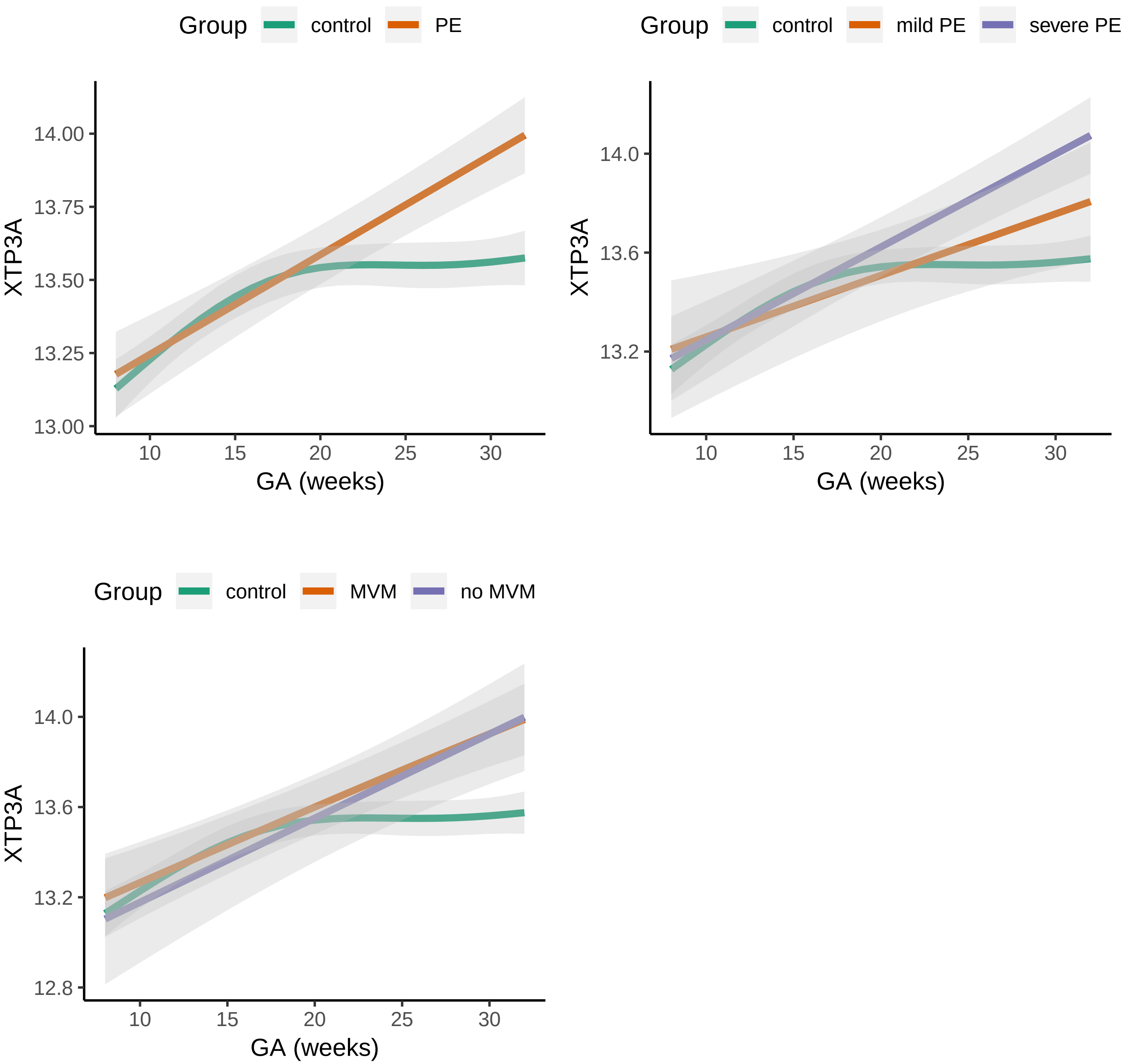

Group
control
PE
XTP3A
14.00
13.75
13.50
13.25
13.00
10
15
20
25
30
GA (weeks)
Group
control
mild PE
severe PE
XTP3A
14.0
13.6
13.2
10
15
20
25
30
GA (weeks)
Group
control
MVM
no MVM
XTP3A
14.0
13.6
13.2
12.8
10
15
20
25
30
GA (weeks)

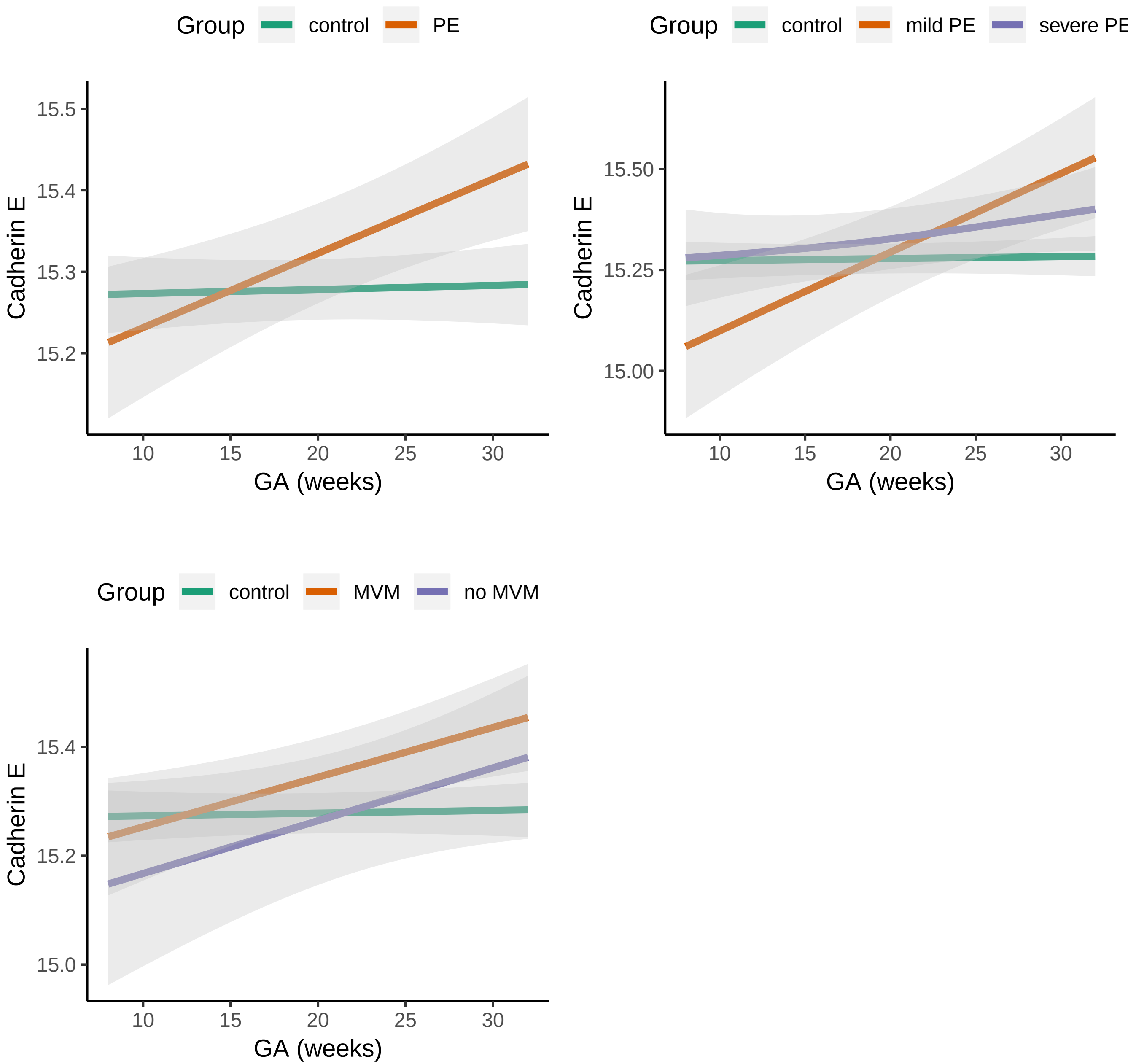

Group
control
PE
Cadherin E
15.5
15.4
15.3
15.2
10
15
20
25
30
GA (weeks)
Group
control
mild PE
severe PE
Cadherin E
15.50
15.25
15.00
10
15
20
25
30
GA (weeks)
Group
control
MVM
no MVM
Cadherin E
15.4
15.2
15.0
10
15
20
25
30
GA (weeks)

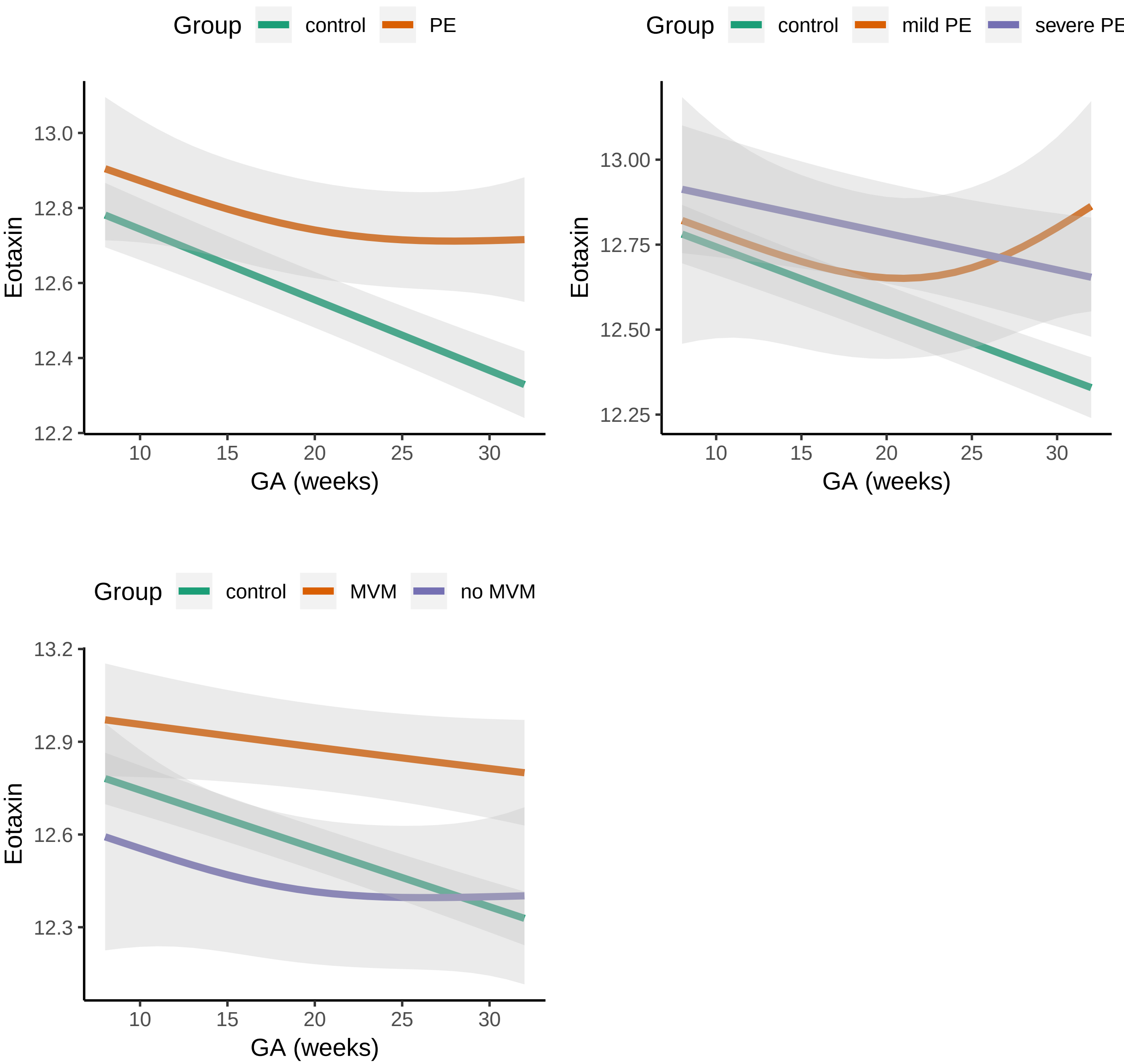

Group
control
PE
Eotaxin
13.0
12.8
12.6
12.4
12.2
10
15
20
25
30
GA (weeks)
Group
control
mild PE
severe PE
Eotaxin
13.00
12.75
12.50
12.25
10
15
20
25
30
GA (weeks)
Group
control
MVM
no MVM
Eotaxin
13.2
12.9
12.6
12.3
10
15
20
25
30
GA (weeks)

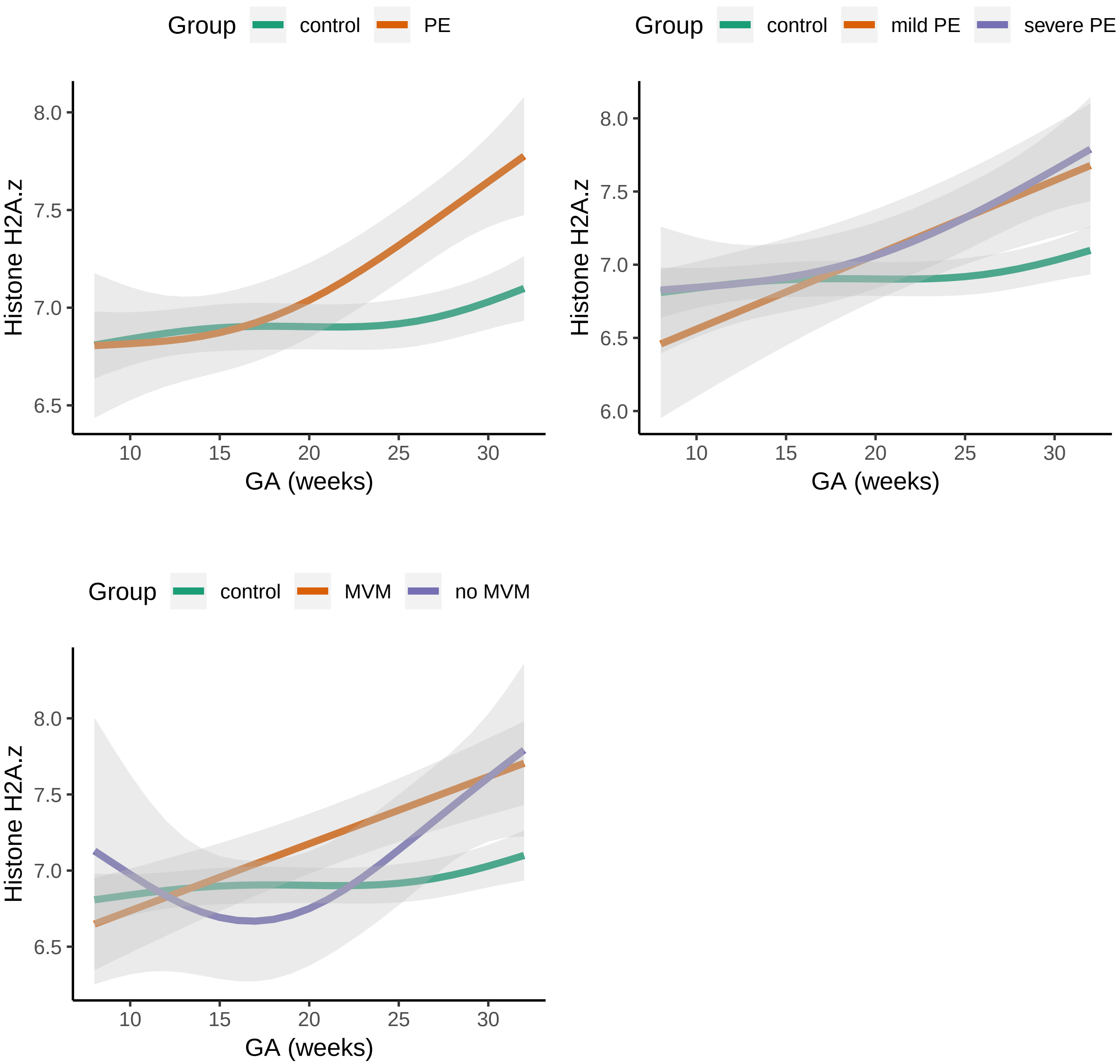

Group
control
PE
Histone H2A.z
8.0
7.5
7.0
6.5
10
15
20
25
30
GA (weeks)
Group
control
mild PE
severe PE
Histone H2A.z
8.0
7.5
7.0
6.5
6.0
10
15
20
25
30
GA (weeks)
Group
control
MVM
no MVM
Histone H2A.z
8.0
7.5
7.0
6.5
10
15
20
25
30
GA (weeks)

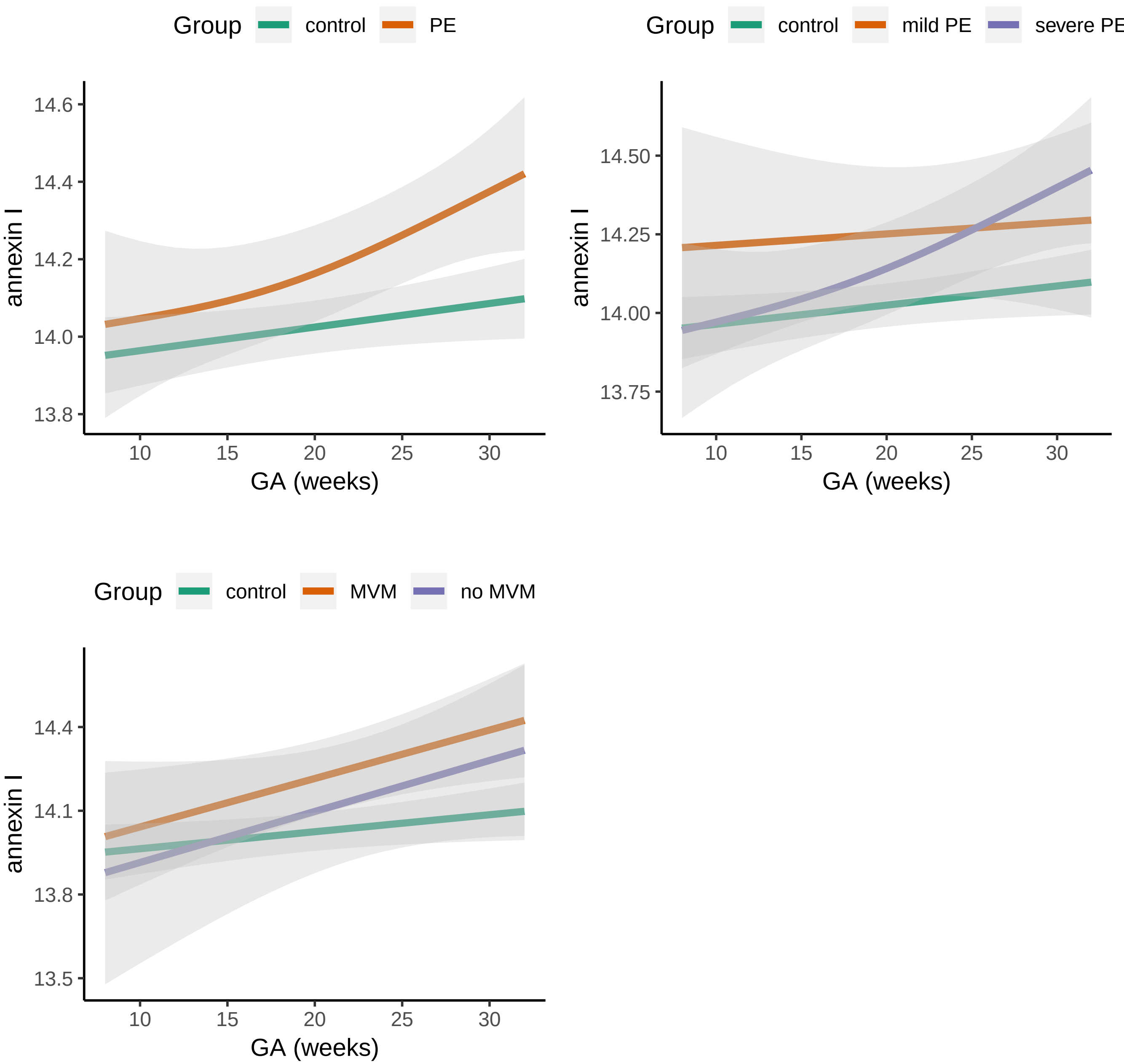
Group
control
PE
annexin I
14.6
14.4
14.2
14.0
13.8
10
15
20
25
30
GA (weeks)
Group
control
mild PE
severe PE
annexin I
14.50
14.25
14.00
13.75
10
15
20
25
30
GA (weeks)
Group
control
MVM
no MVM
annexin I
14.4
14.1
13.8
13.5
10
15
20
25
30
GA (weeks)

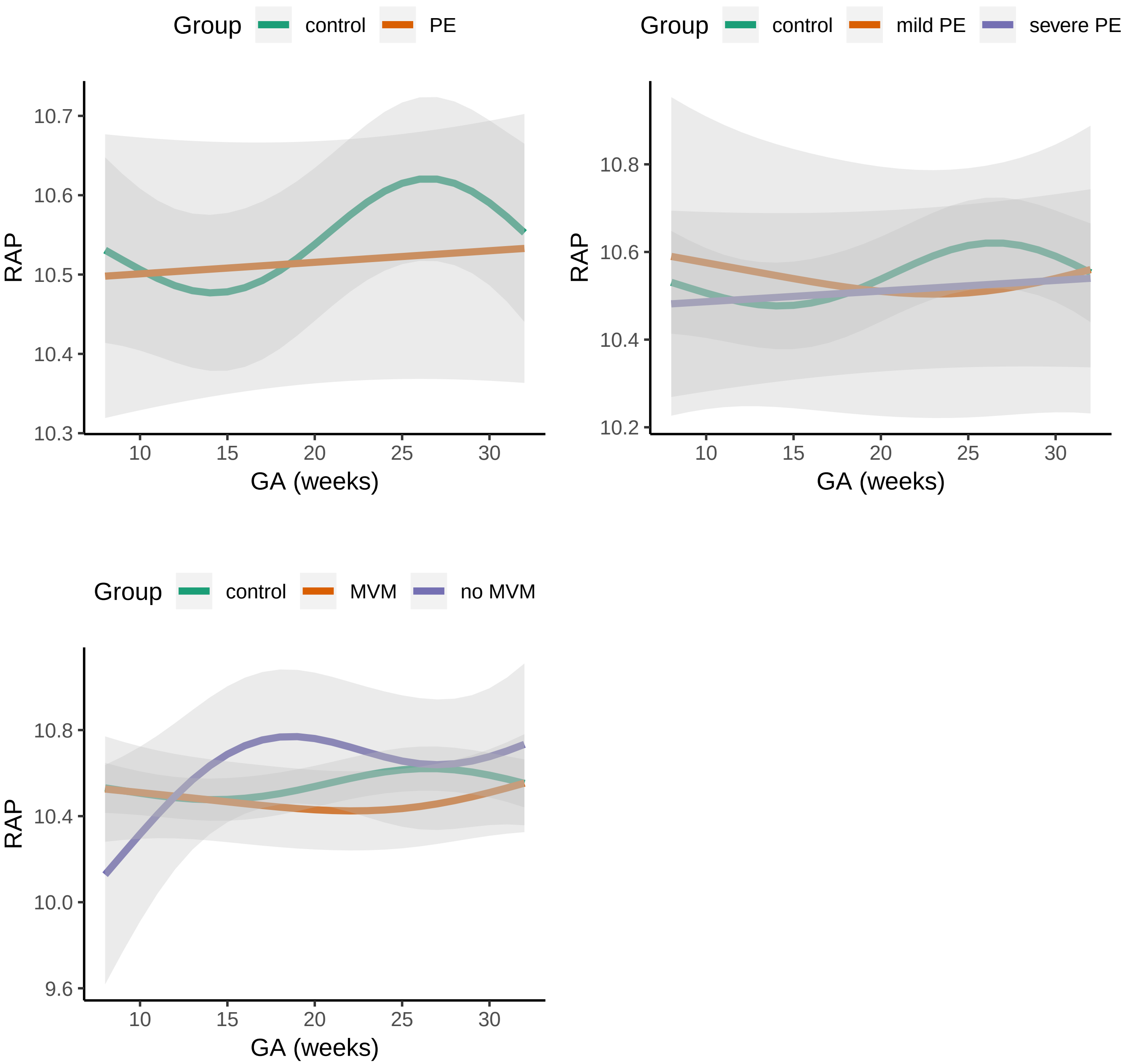

Group
control
PE
10.7
10.6
10.5
10.4
10.3
RAP
10
15
20
25
30
GA (weeks)
Group
control
mild PE
severe PE
10.8
10.6
10.4
10.2
RAP
10
15
20
25
30
GA (weeks)
Group
control
MVM
no MVM
10.8
10.4
10.0
9.6
RAP
10
15
20
25
30
GA (weeks)

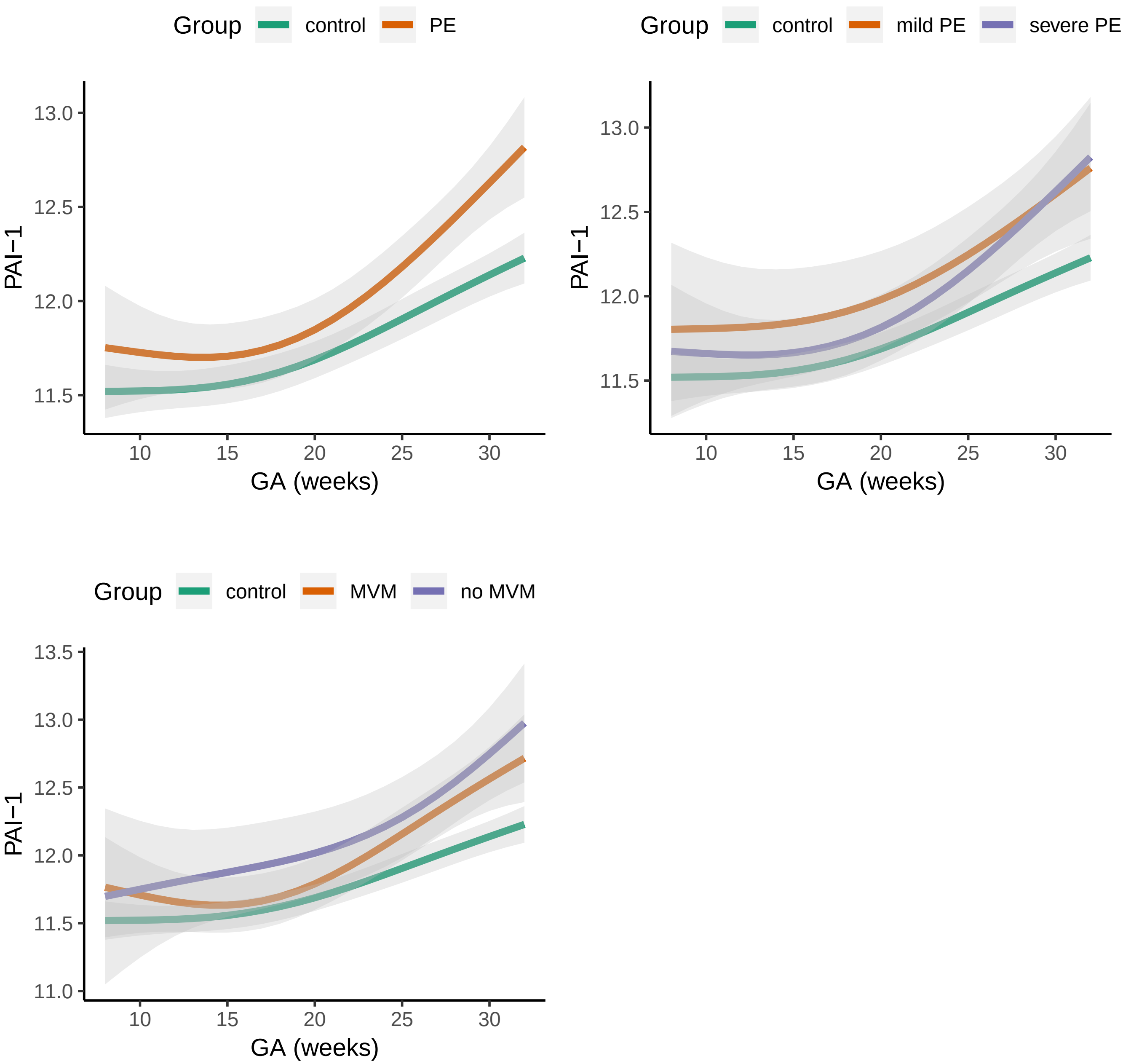

Group
control
PE
PAI−1
13.0
12.5
12.0
11.5
10
15
20
25
30
GA (weeks)
Group
control
mild PE
severe PE
PAI−1
13.0
12.5
12.0
11.5
10
15
20
25
30
GA (weeks)
Group
control
MVM
no MVM
PAI−1
13.5
13.0
12.5
12.0
11.5
11.0
10
15
20
25
30
GA (weeks)

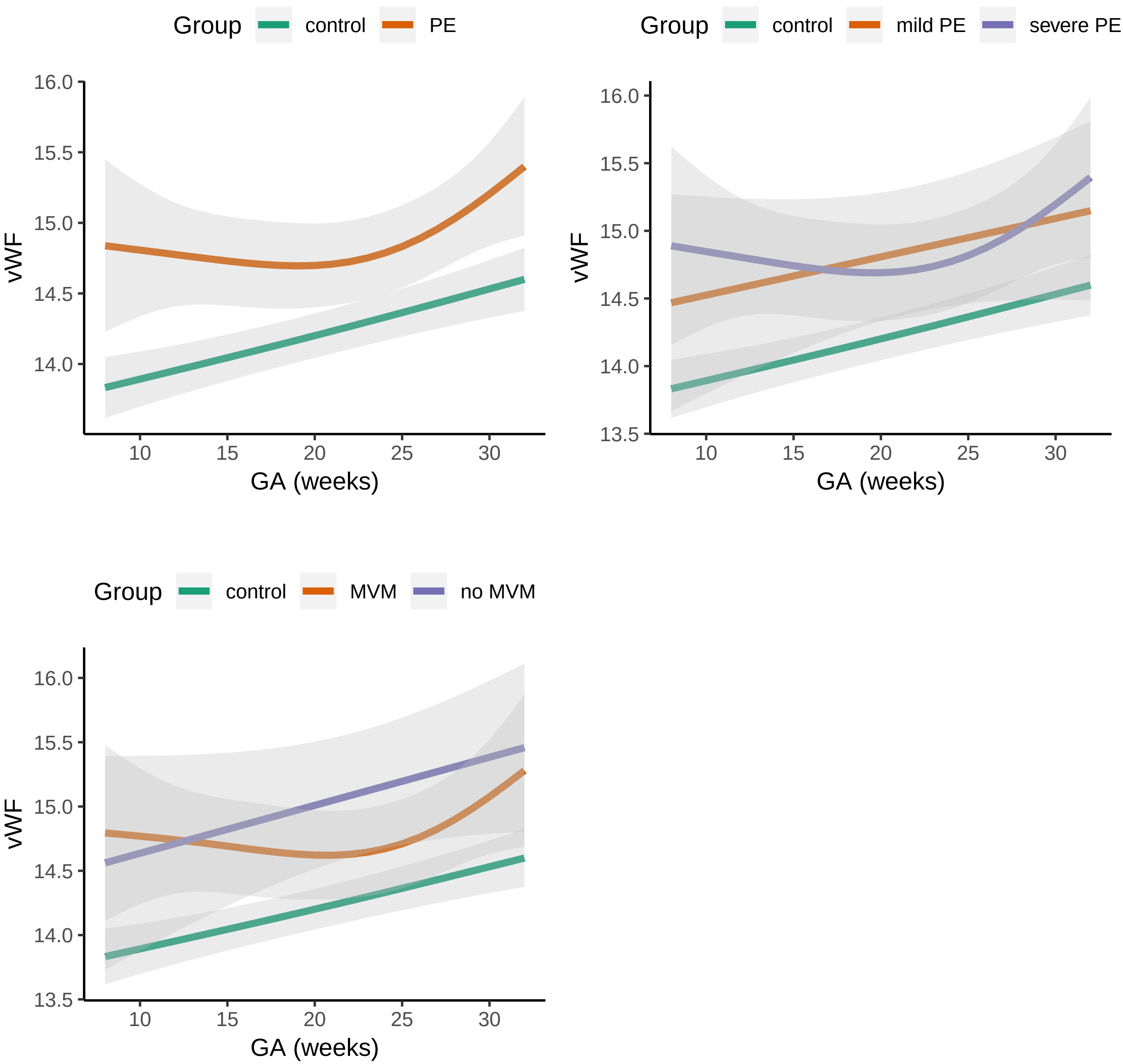
Group
control
PE
vWF
16.0
15.5
15.0
14.5
14.0
10
15
20
25
30
GA (weeks)
Group
control
mild PE
severe PE
vWF
16.0
15.5
15.0
14.5
14.0
13.5
10
15
20
25
30
GA (weeks)
Group
control
MVM
no MVM
vWF
16.0
15.5
15.0
14.5
14.0
13.5
10
15
20
25
30
GA (weeks)

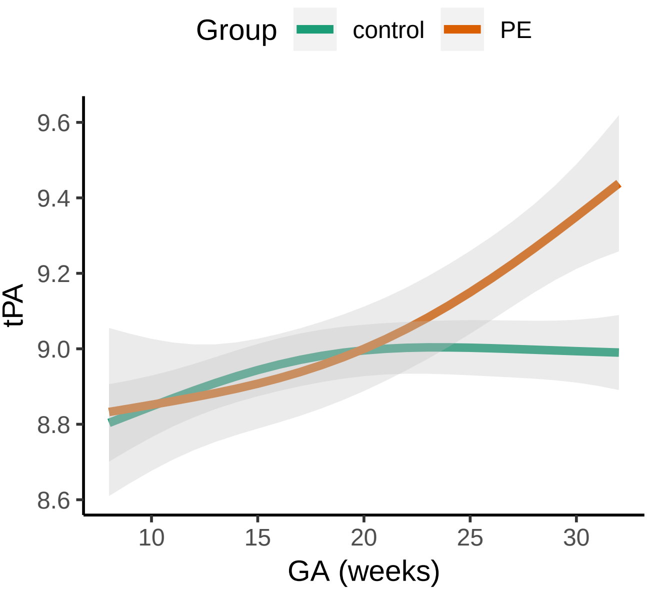

Group
control
PE
tPA
9.6
9.4
9.2
9.0
8.8
8.6
10
15
20
25
30
GA (weeks)

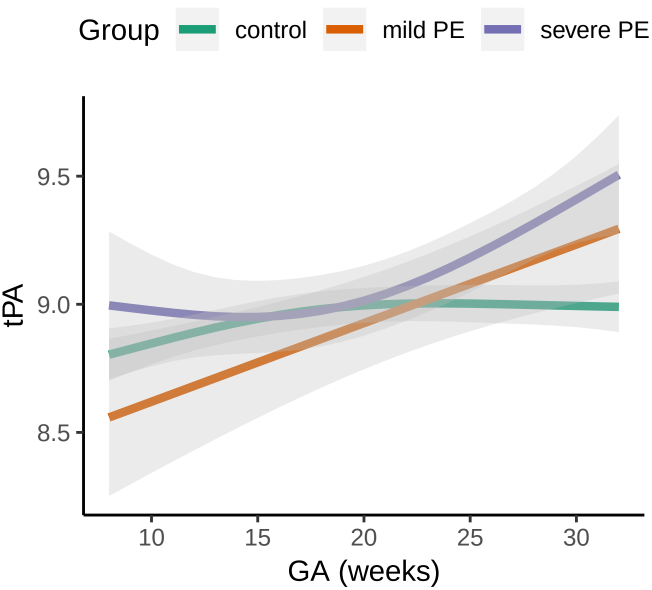

Group
control
mild PE
severe PE
tPA
9.5
9.0
8.5
10
15
20
25
30
GA (weeks)

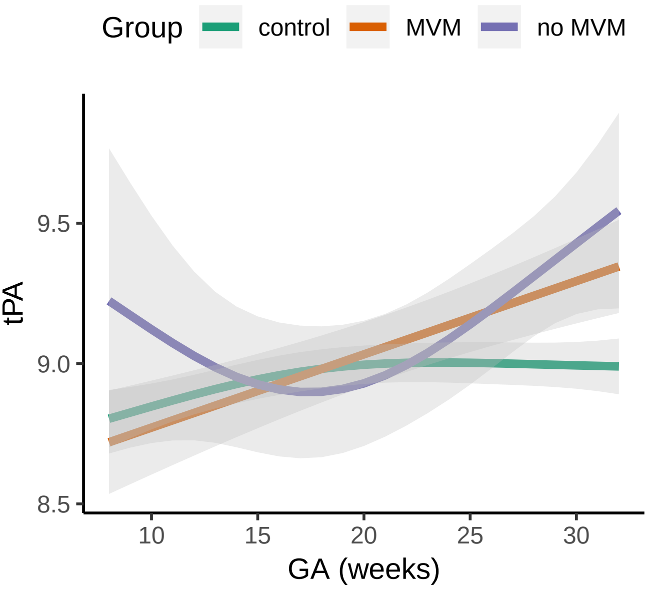

Group
control
MVM
no MVM
tPA
9.5
9.0
8.5
10
15
20
25
30
GA (weeks)

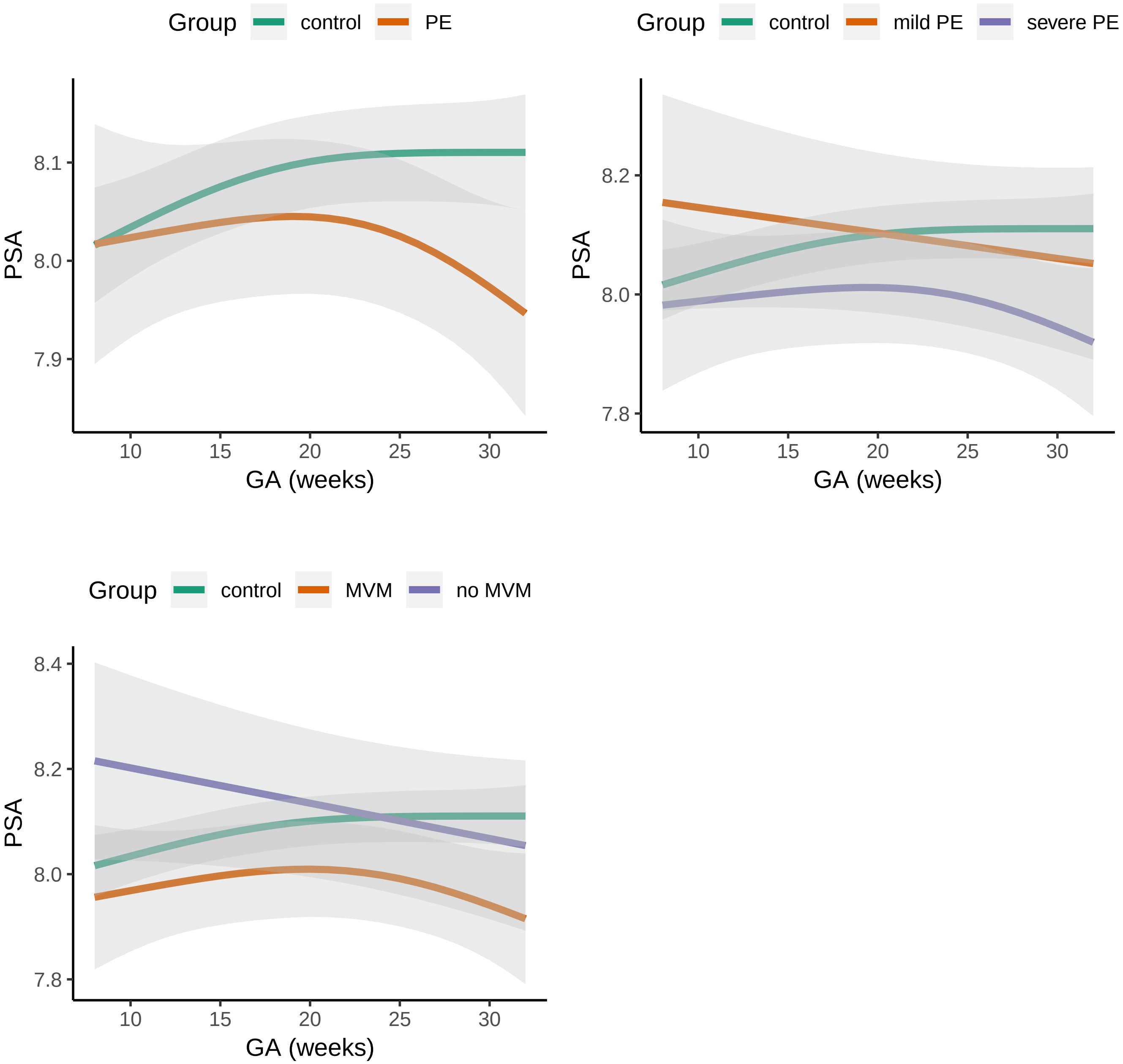

Group
control
PE
PSA
8.1
8.0
7.9
10
15
20
25
30
GA (weeks)
Group
control
mild PE
severe PE
PSA
8.2
8.0
7.8
10
15
20
25
30
GA (weeks)
Group
control
MVM
no MVM
PSA
8.4
8.2
8.0
7.8
10
15
20
25
30
GA (weeks)

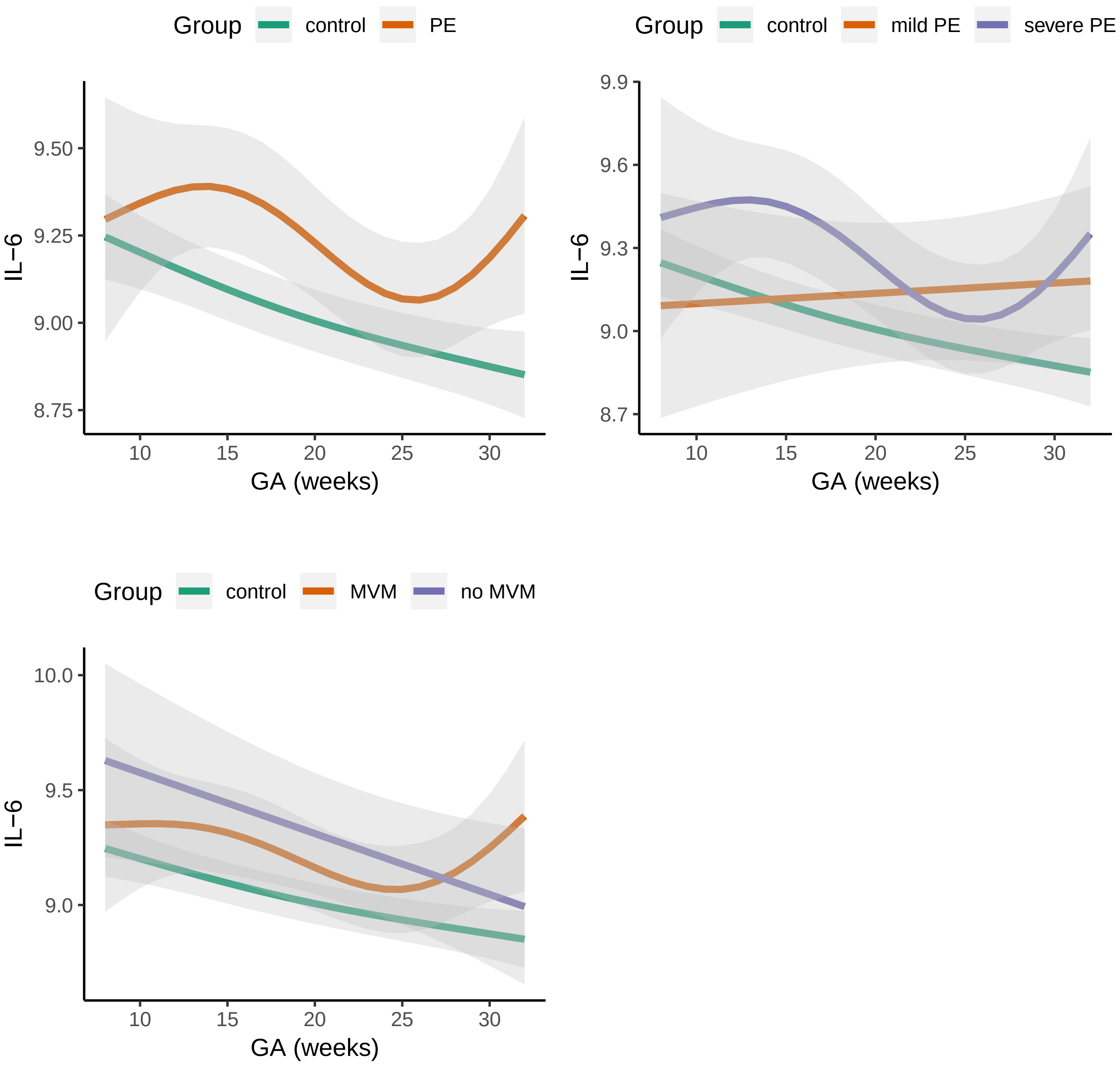

Group
control
PE
IL−6
9.50
9.25
9.00
8.75
10
15
20
25
30
GA (weeks)
Group
control
mild PE
severe PE
IL−6
9.9
9.6
9.3
9.0
8.7
10
15
20
25
30
GA (weeks)
Group
control
MVM
no MVM
IL−6
10.0
9.5
9.0
10
15
20
25
30
GA (weeks)

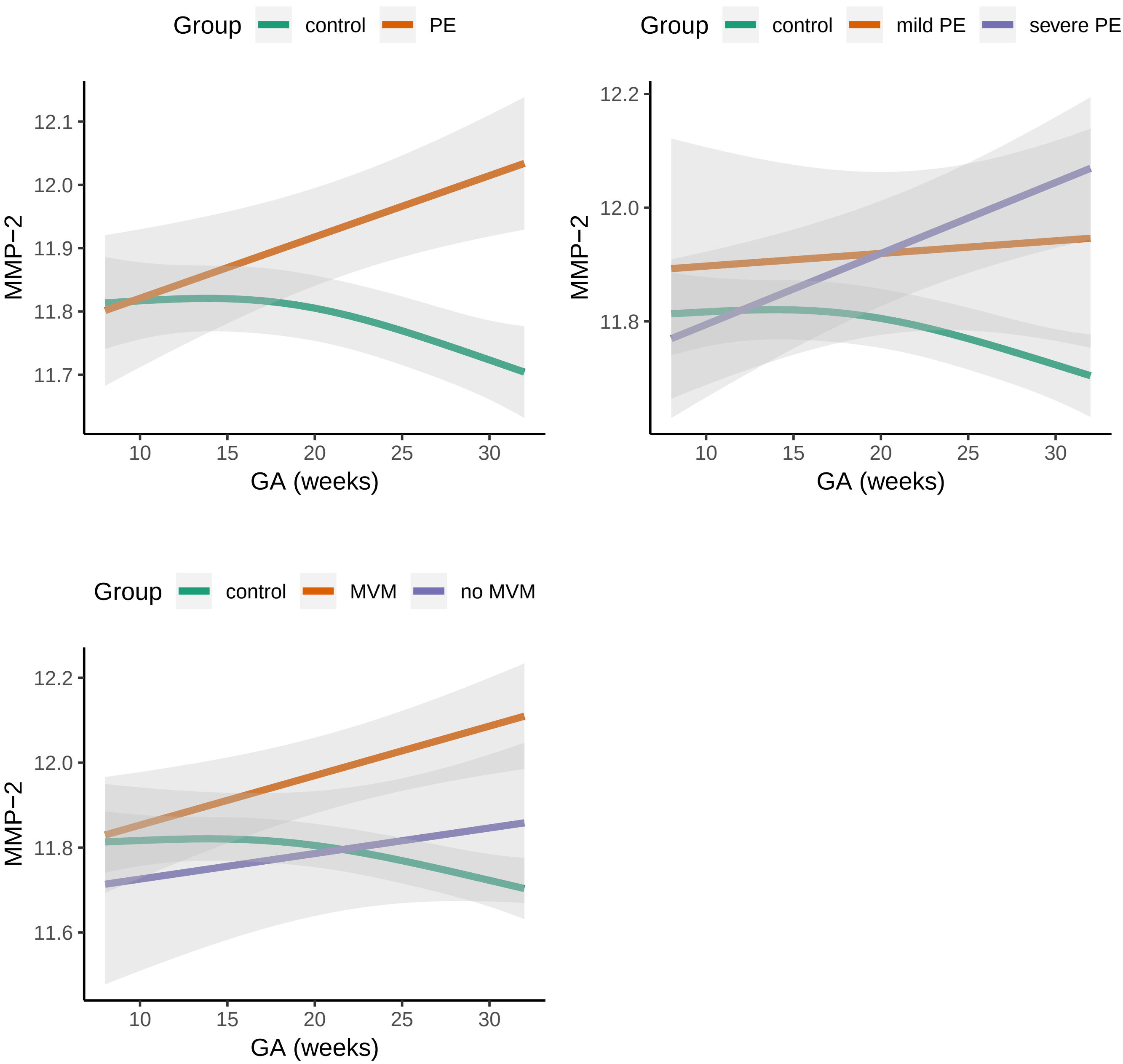
Group
control
PE
MMP−2
12.1
12.0
11.9
11.8
11.7
10
15
20
25
30
GA (weeks)
Group
control
mild PE
severe PE
MMP−2
12.2
12.0
11.8
10
15
20
25
30
GA (weeks)
Group
control
MVM
no MVM
MMP−2
12.2
12.0
11.8
11.6
10
15
20
25
30
GA (weeks)

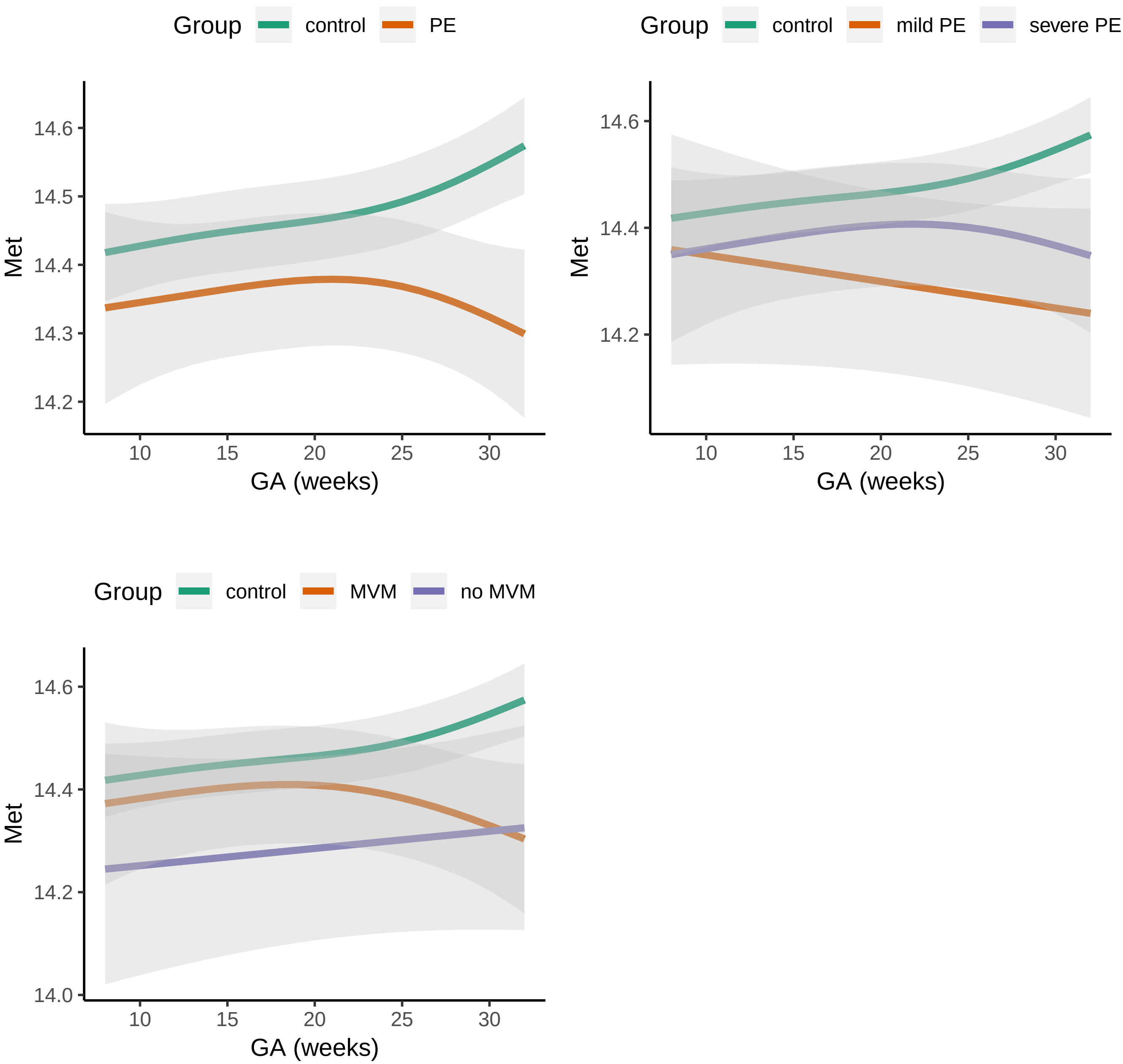

Group
control
PE
Met
14.6
14.5
14.4
14.3
14.2
10
15
20
25
30
GA (weeks)
Group
control
mild PE
severe PE
Met
14.6
14.4
14.2
10
15
20
25
30
GA (weeks)
Group
control
MVM
no MVM
Met
14.6
14.4
14.2
14.0
10
15
20
25
30
GA (weeks)

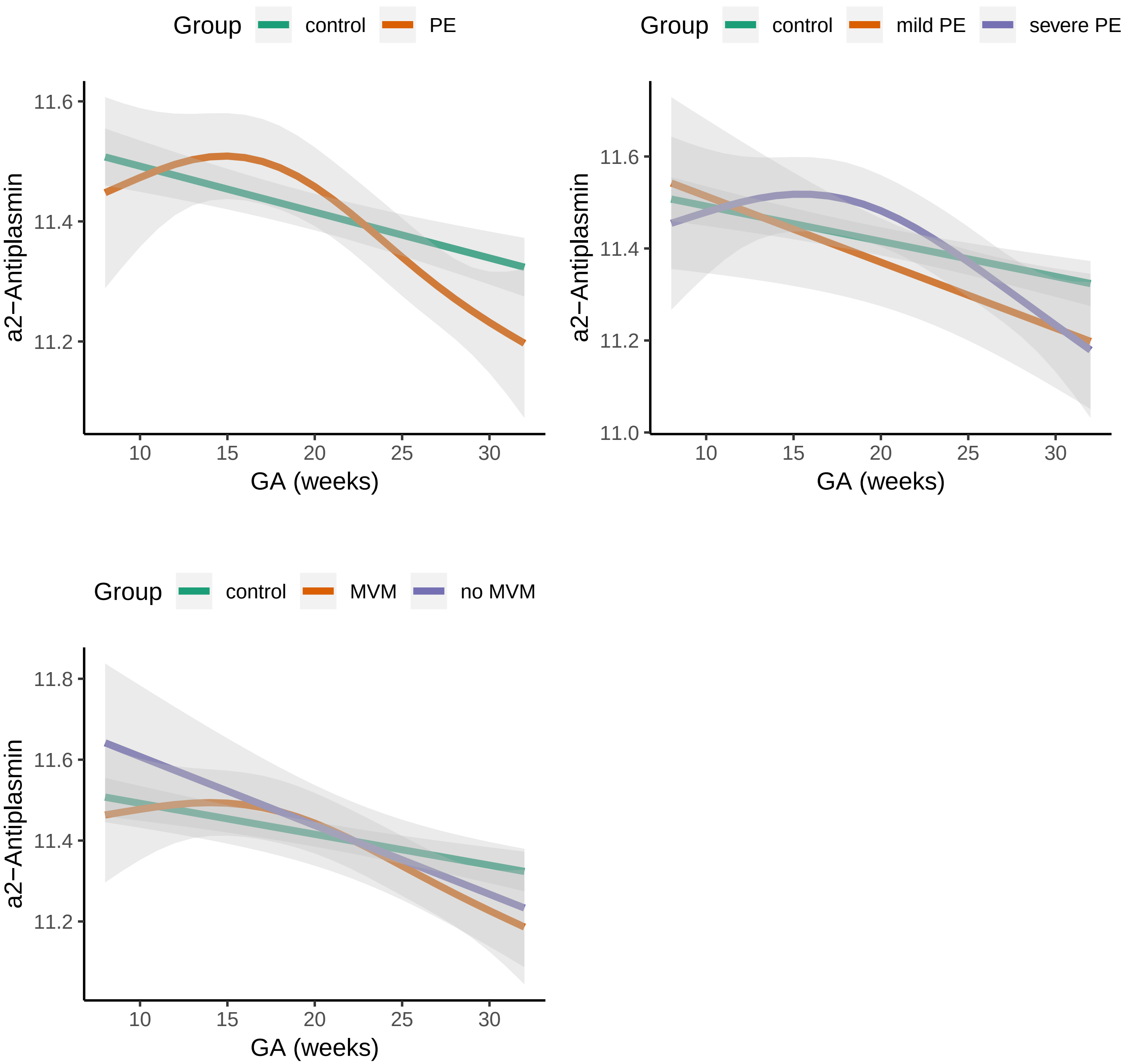

Group
control
PE
a2−Antiplasmin
11.6
11.4
11.2
10
15
20
25
30
GA (weeks)
Group
control
mild PE
severe PE
a2−Antiplasmin
11.6
11.4
11.2
11.0
10
15
20
25
30
GA (weeks)
Group
control
MVM
no MVM
a2−Antiplasmin
11.8
11.6
11.4
11.2
10
15
20
25
30
GA (weeks)

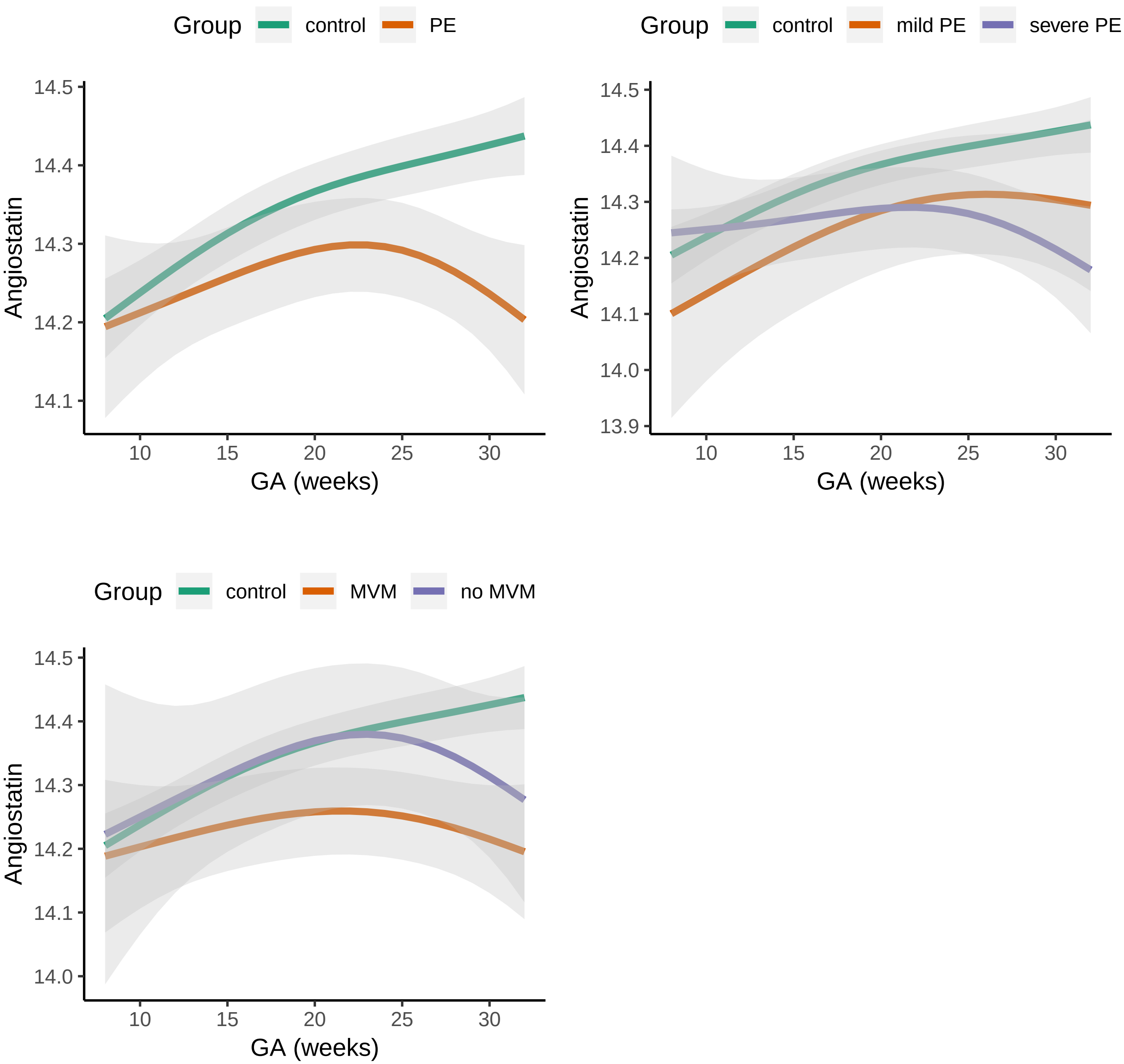

Group
control
PE
Angiostatin
14.5
14.4
14.3
14.2
14.1
10
15
20
25
30
GA (weeks)
Group
control
mild PE
severe PE
Angiostatin
14.5
14.4
14.3
14.2
14.1
14.0
13.9
10
15
20
25
30
GA (weeks)
Group
control
MVM
no MVM
Angiostatin
14.5
14.4
14.3
14.2
14.1
14.0
10
15
20
25
30
GA (weeks)

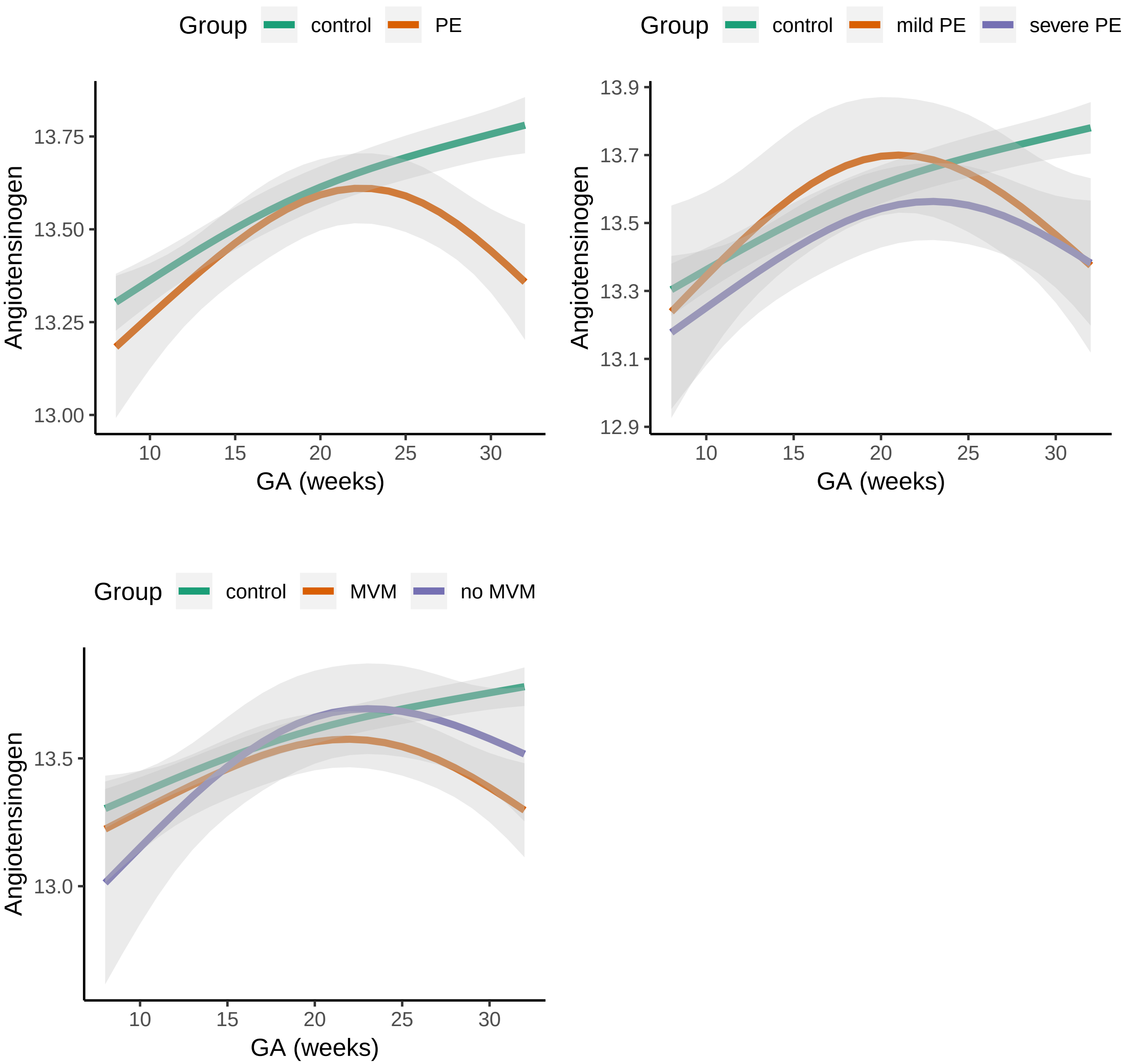

Group
control
PE
13.75
13.50
13.25
13.00
Angiotensinogen
10
15
20
25
30
GA (weeks)
Group
control
mild PE
severe PE
13.9
13.7
13.5
13.3
13.1
12.9
Angiotensinogen
10
15
20
25
30
GA (weeks)
Group
control
MVM
no MVM
13.5
13.0
Angiotensinogen
10
15
20
25
30
GA (weeks)

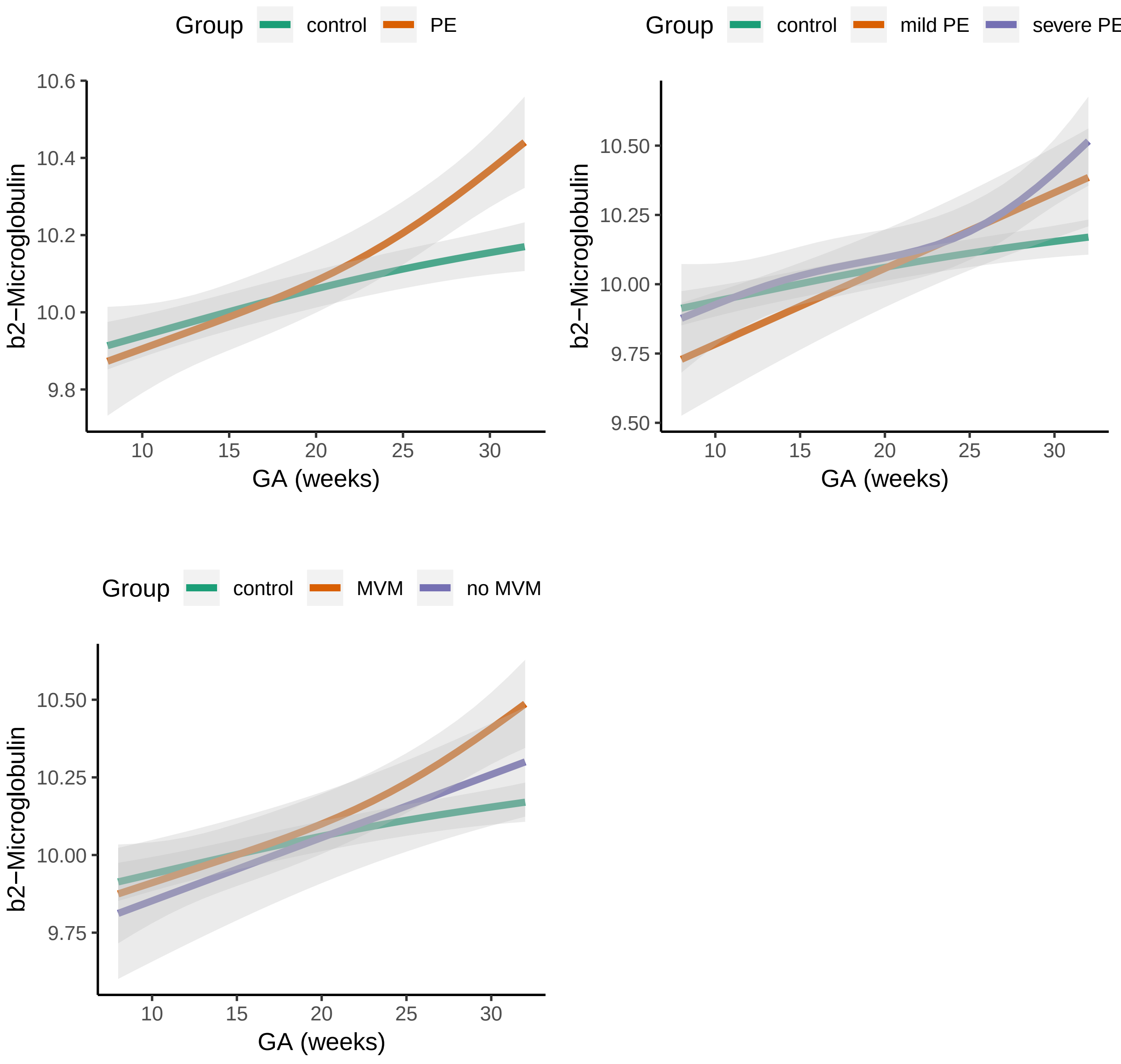

Group
control
PE
b2−Microglobulin
10.6
10.4
10.2
10.0
9.8
10
15
20
25
30
GA (weeks)
Group
control
mild PE
severe PE
b2−Microglobulin
10.50
10.25
10.00
9.75
9.50
10
15
20
25
30
GA (weeks)
Group
control
MVM
no MVM
b2−Microglobulin
10.50
10.25
10.00
9.75
10
15
20
25
30
GA (weeks)

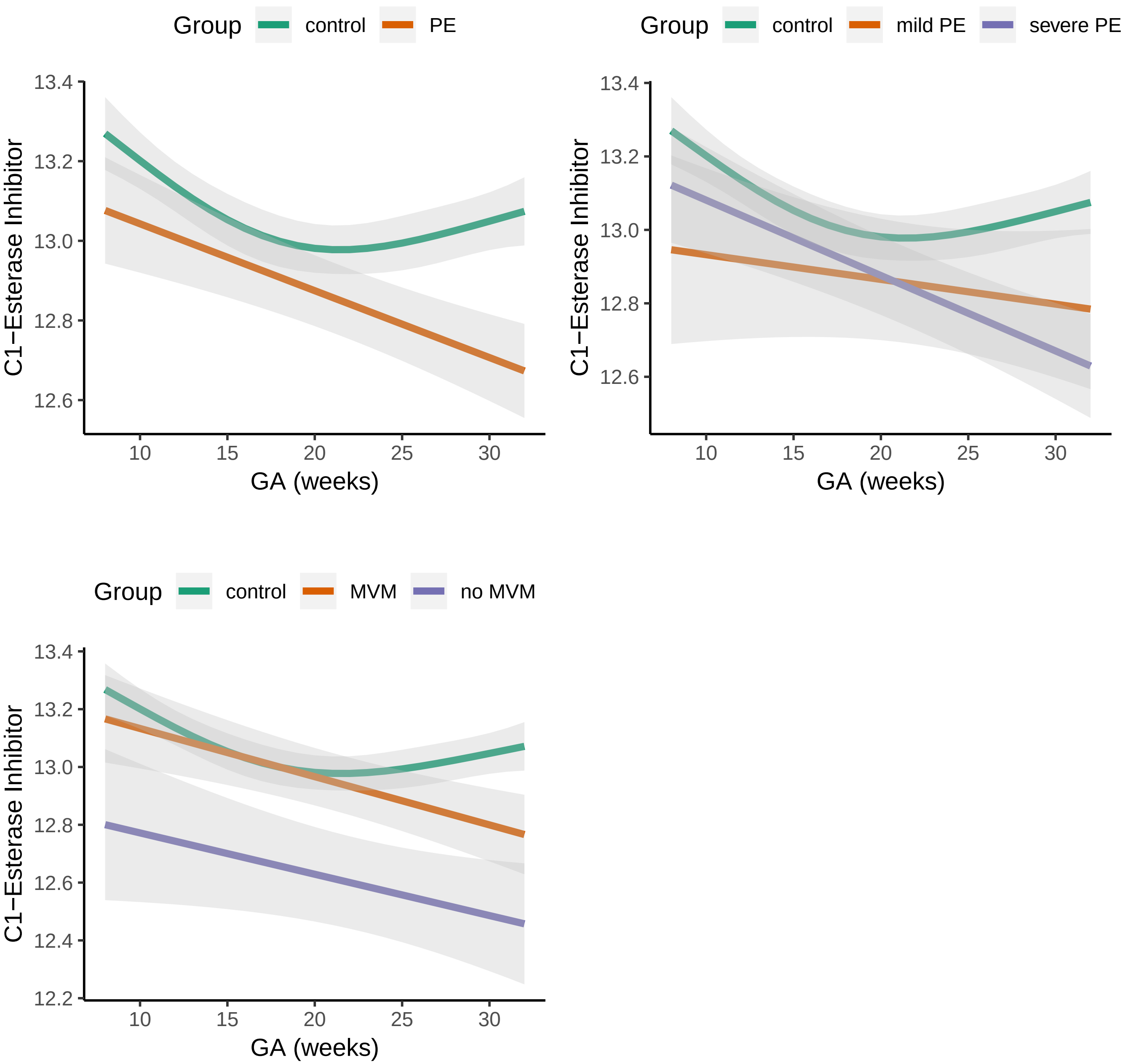

Group
control
PE
C1−Esterase Inhibitor
13.4
13.2
13.0
12.8
12.6
10
15
20
25
30
GA (weeks)
Group
control
mild PE
severe PE
C1−Esterase Inhibitor
13.4
13.2
13.0
12.8
12.6
10
15
20
25
30
GA (weeks)
Group
control
MVM
no MVM
C1−Esterase Inhibitor
13.4
13.2
13.0
12.8
12.6
12.4
12.2
10
15
20
25
30
GA (weeks)

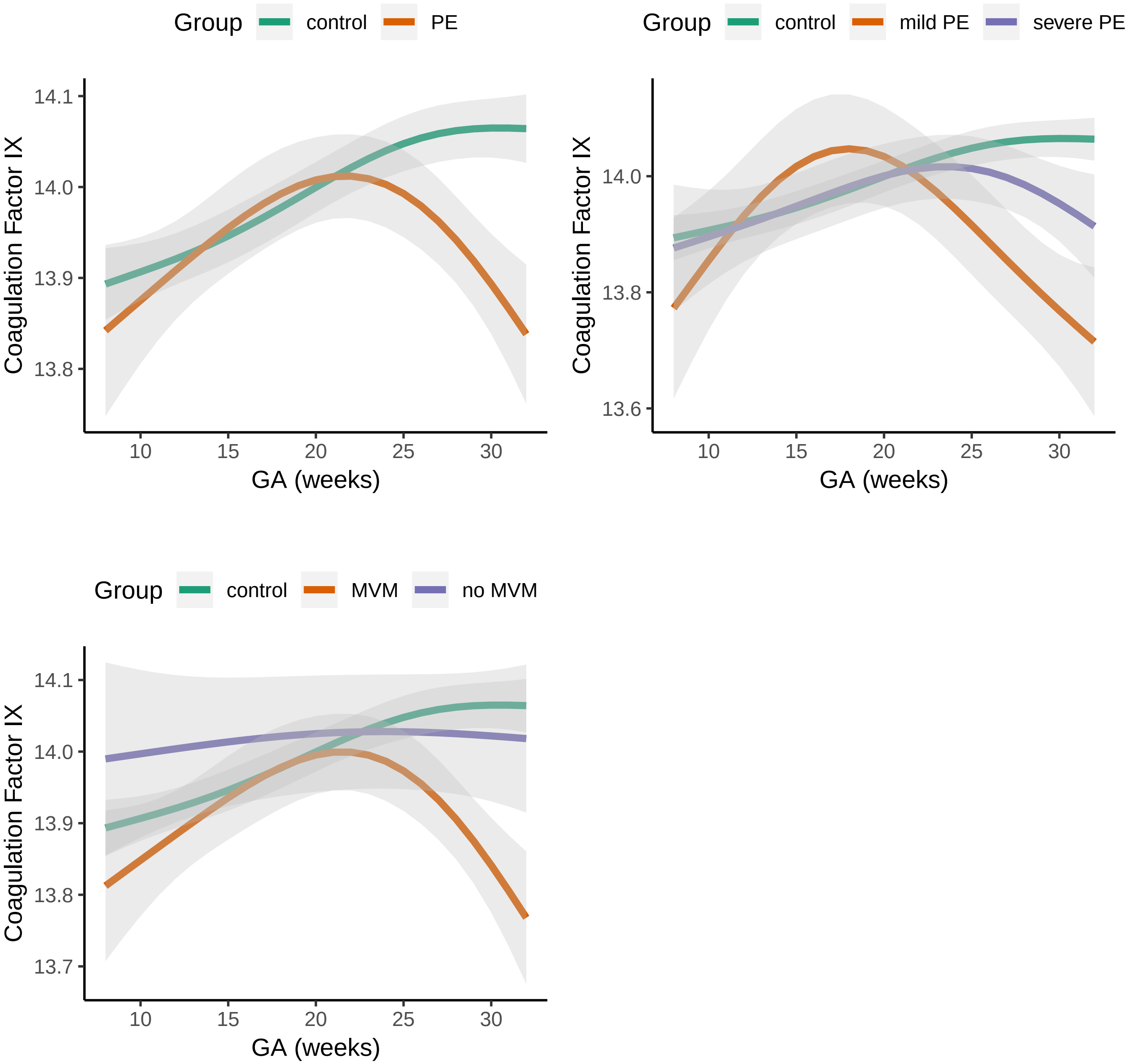

Group
control
PE
Coagulation Factor IX
14.1
14.0
13.9
13.8
10
15
20
25
30
GA (weeks)
Group
control
mild PE
severe PE
Coagulation Factor IX
14.0
13.8
13.6
10
15
20
25
30
GA (weeks)
Group
control
MVM
no MVM
Coagulation Factor IX
14.1
14.0
13.9
13.8
13.7
10
15
20
25
30
GA (weeks)

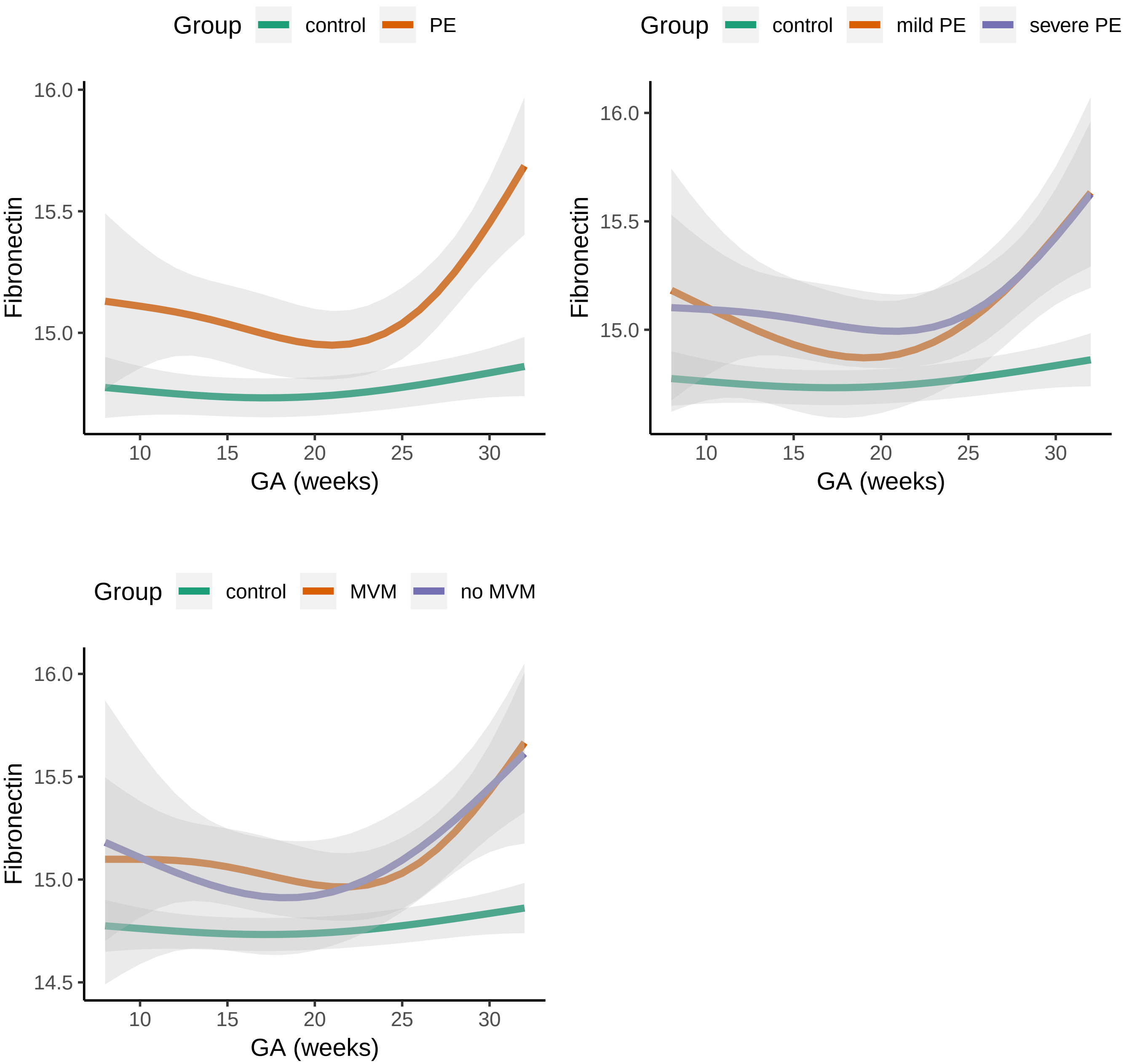

Group
control
PE
Fibronectin
16.0
15.5
15.0
10
15
20
25
30
GA (weeks)
Group
control
mild PE
severe PE
Fibronectin
16.0
15.5
15.0
10
15
20
25
30
GA (weeks)
Group
control
MVM
no MVM
Fibronectin
16.0
15.5
15.0
14.5
10
15
20
25
30
GA (weeks)

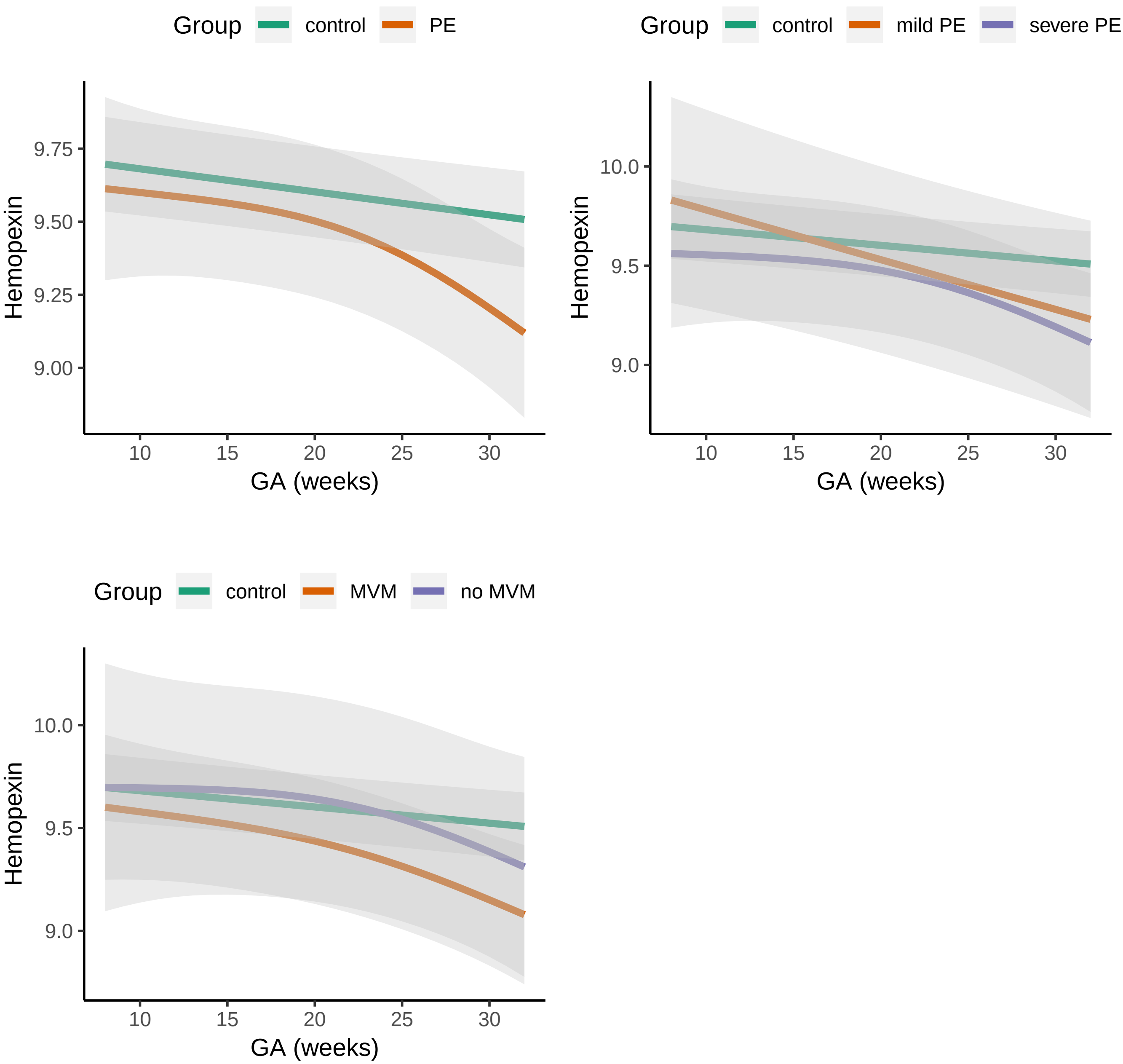

Group
control
PE
Hemopexin
9.75
9.50
9.25
9.00
10
15
20
25
30
GA (weeks)
Group
control
mild PE
severe PE
Hemopexin
10.0
9.5
9.0
10
15
20
25
30
GA (weeks)
Group
control
MVM
no MVM
Hemopexin
10.0
9.5
9.0
10
15
20
25
30
GA (weeks)

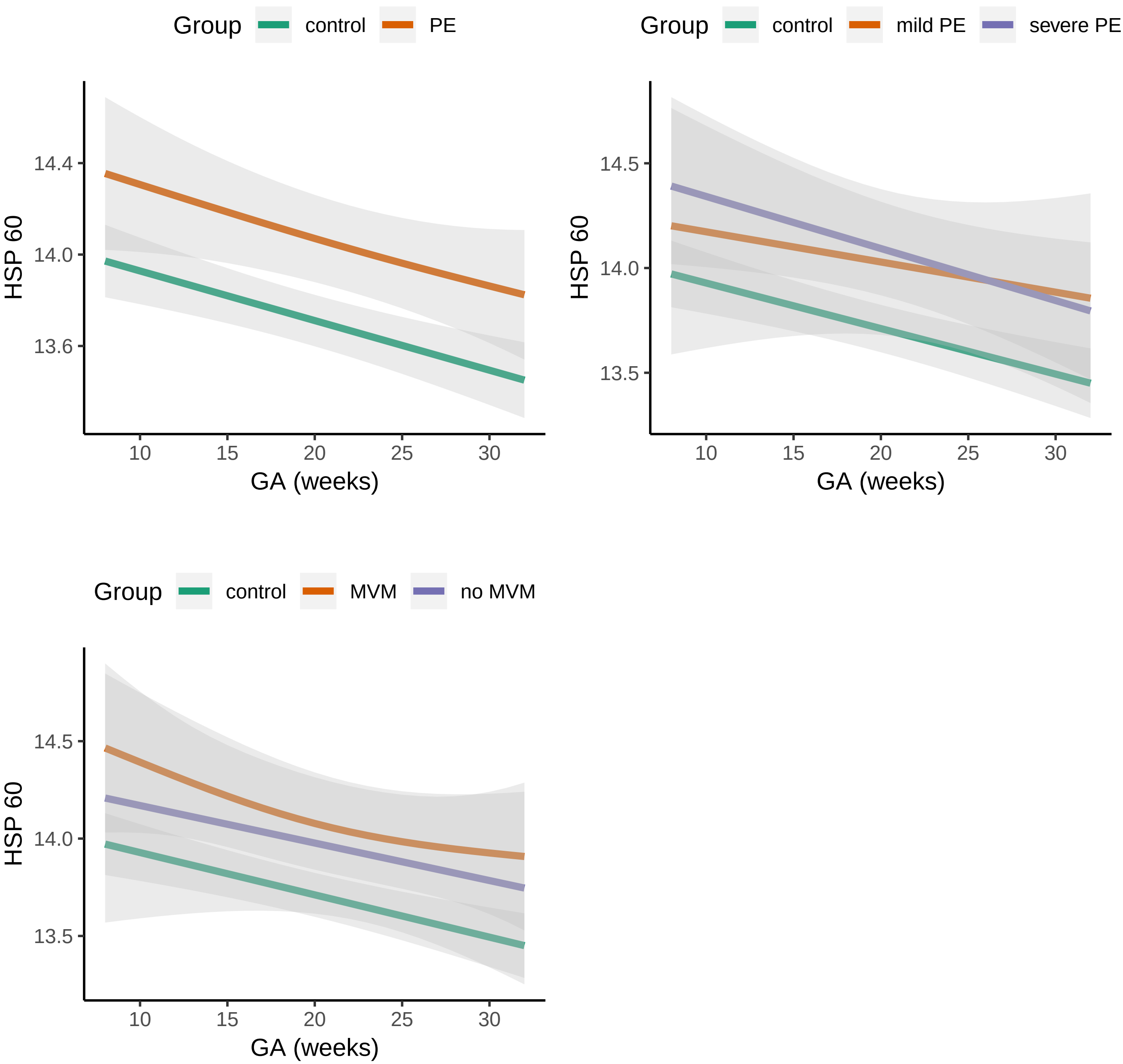
Group
control
PE
HSP 60
14.4
14.0
13.6
10
15
20
25
30
GA (weeks)
Group
control
mild PE
severe PE
HSP 60
14.5
14.0
13.5
10
15
20
25
30
GA (weeks)
Group
control
MVM
no MVM
HSP 60
14.5
14.0
13.5
10
15
20
25
30
GA (weeks)

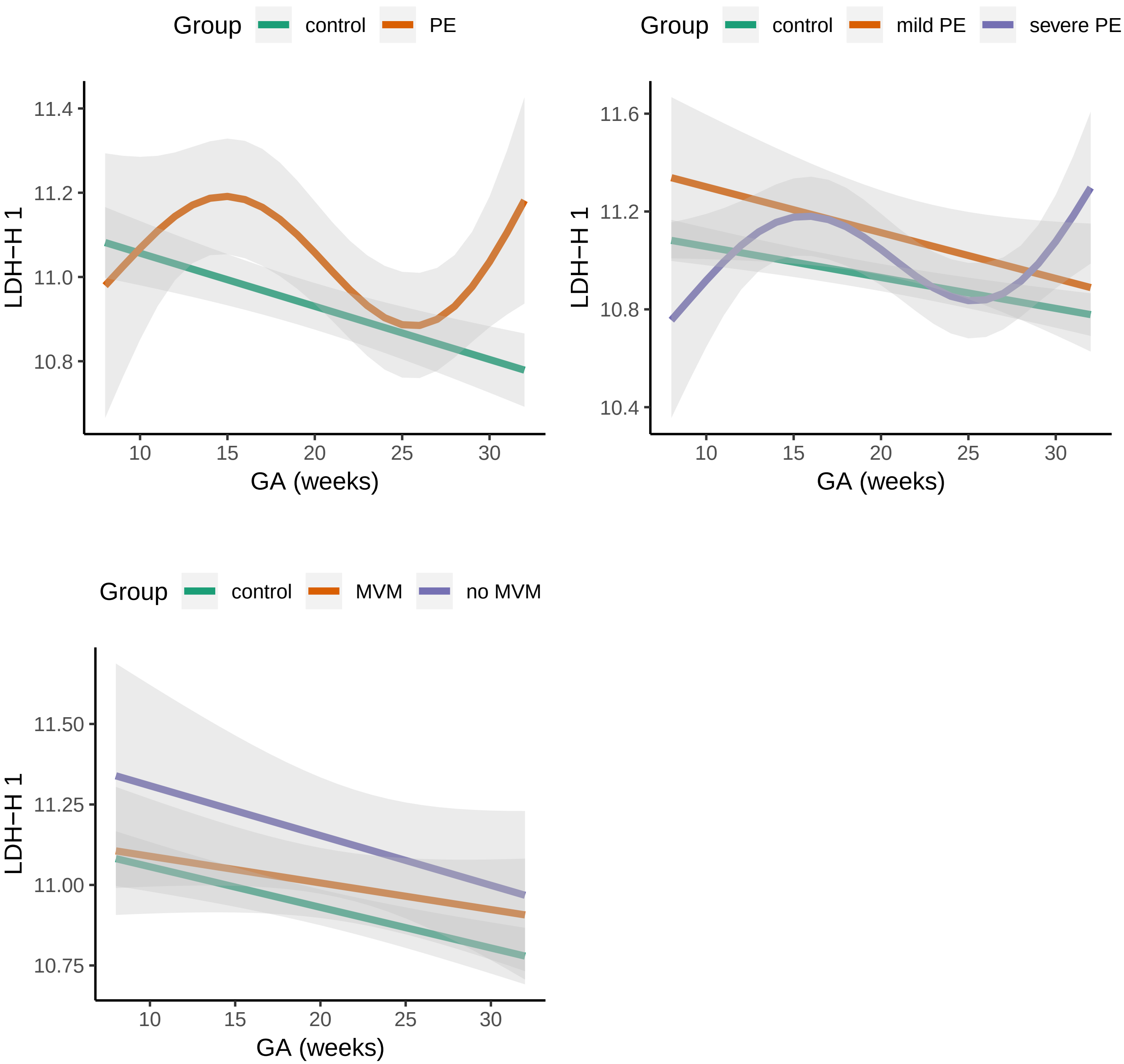
Group
control
PE
LDH−H 1
11.4
11.2
11.0
10.8
10
15
20
25
30
GA (weeks)
Group
control
mild PE
severe PE
LDH−H 1
11.6
11.2
10.8
10.4
10
15
20
25
30
GA (weeks)
Group
control
MVM
no MVM
LDH−H 1
11.50
11.25
11.00
10.75
10
15
20
25
30
GA (weeks)

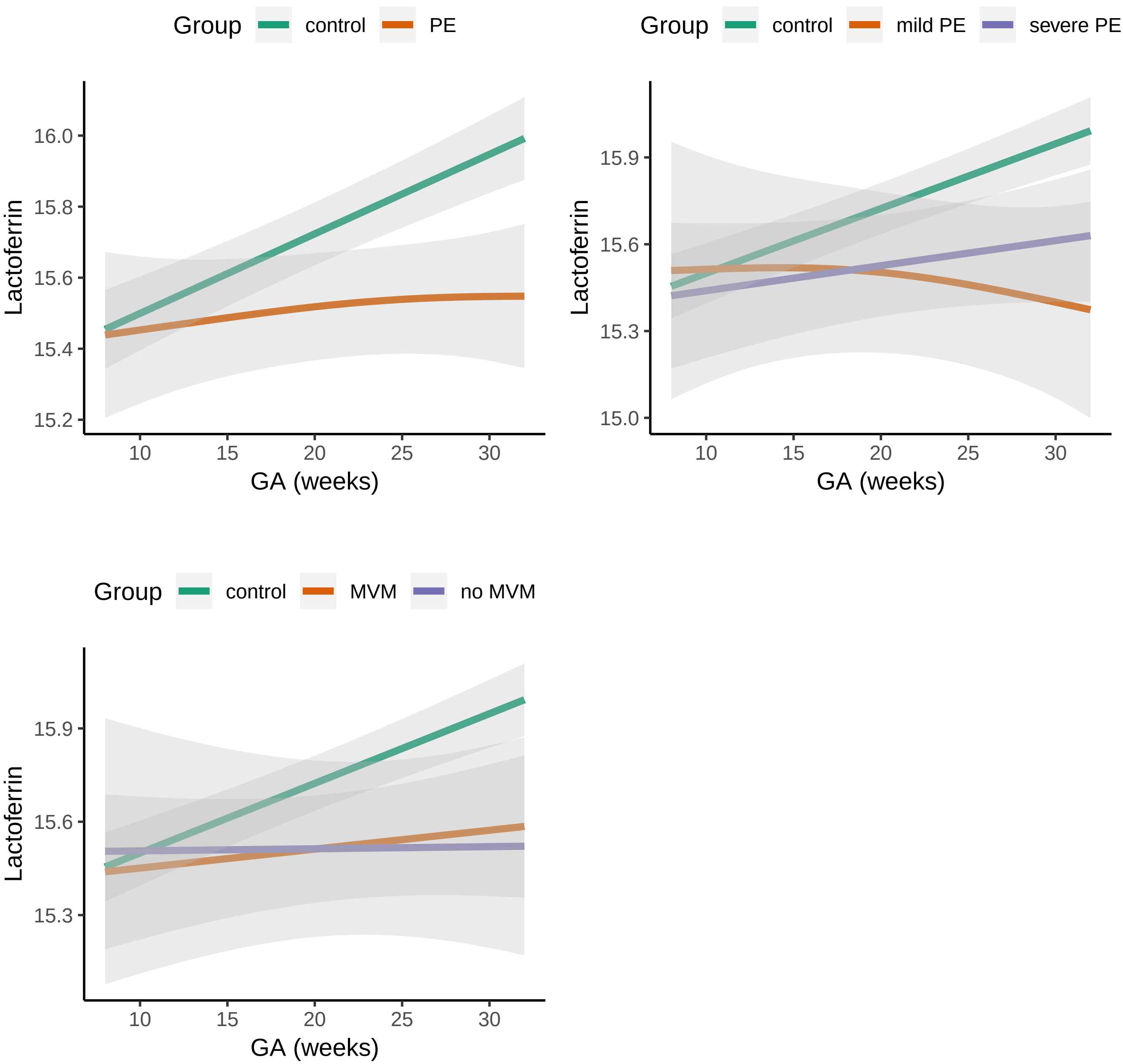

Group
control
PE
Lactoferrin
GA (weeks)
Group
control
mild PE
severe PE
Lactoferrin
GA (weeks)
Group
control
MVM
no MVM
Lactoferrin
GA (weeks)

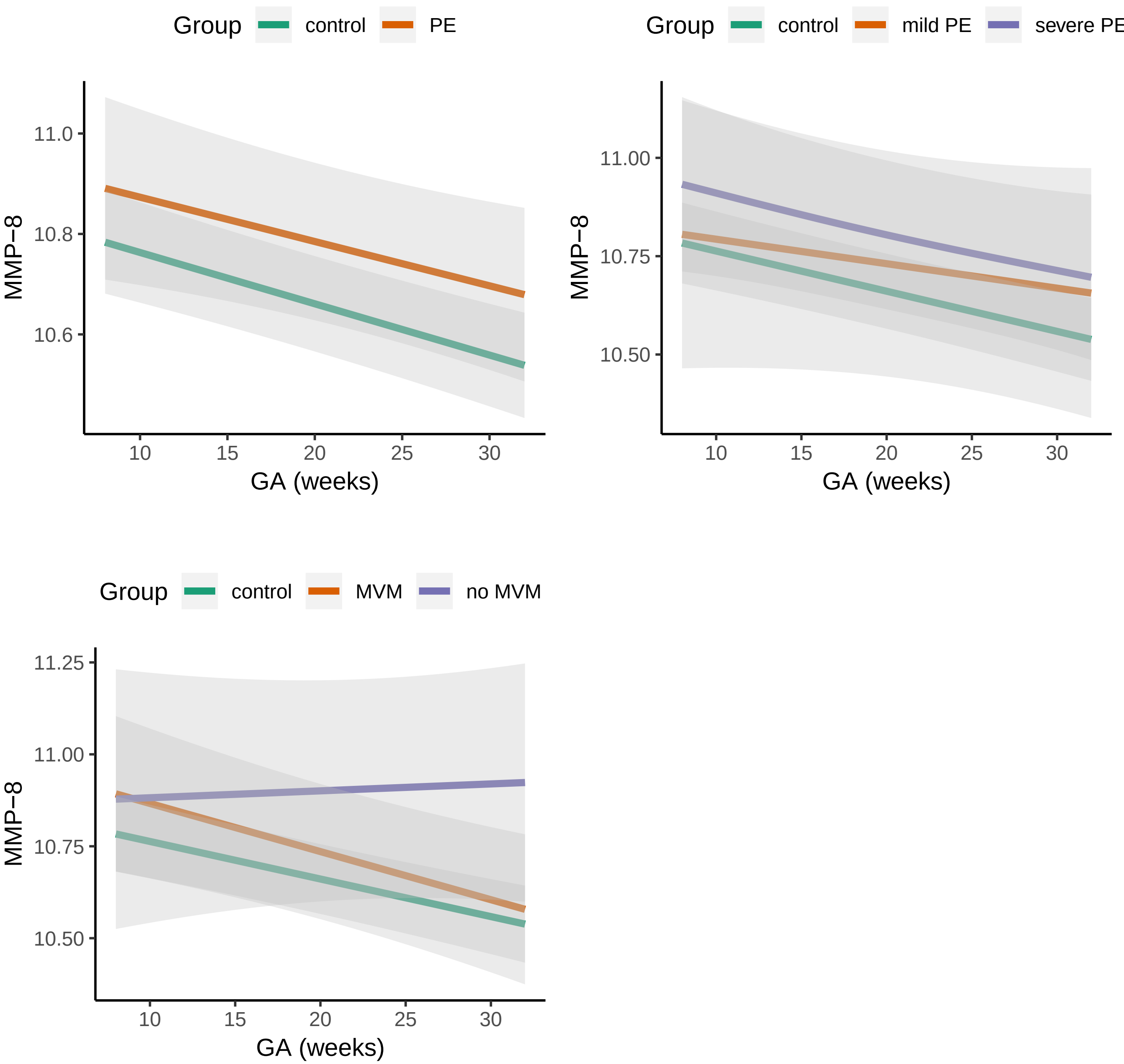
Group
control
PE
MMP−8
11.0
10.8
10.6
10
15
20
25
30
GA (weeks)
Group
control
mild PE
severe PE
MMP−8
11.00
10.75
10.50
10
15
20
25
30
GA (weeks)
Group
control
MVM
no MVM
MMP−8
11.25
11.00
10.75
10.50
10
15
20
25
30
GA (weeks)

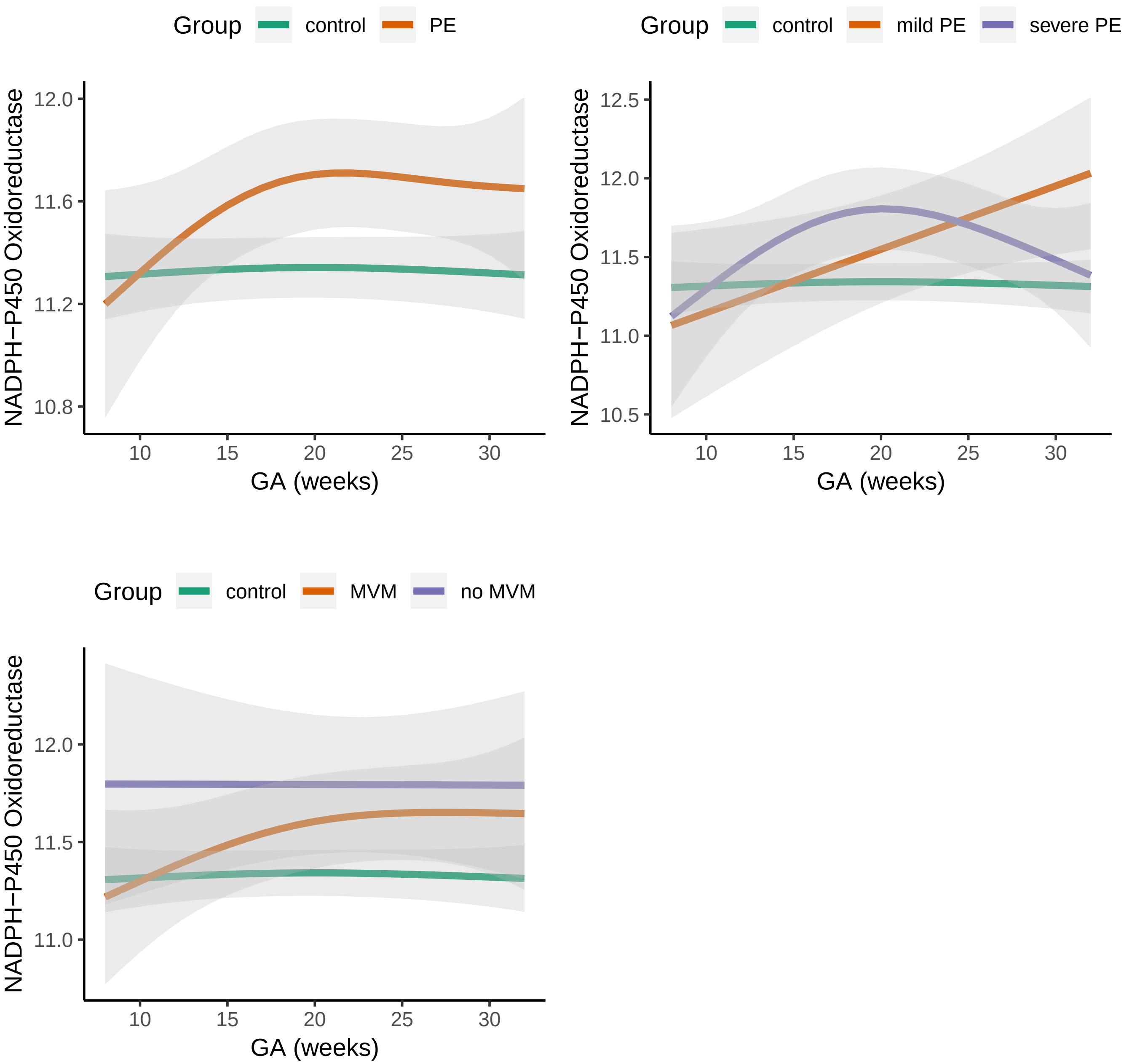

Group
control
PE
NADPH−P450 Oxidoreductase
12.0
11.6
11.2
10.8
10
15
20
25
30
GA (weeks)
Group
control
mild PE
severe PE
NADPH−P450 Oxidoreductase
12.5
12.0
11.5
11.0
10.5
10
15
20
25
30
GA (weeks)
Group
control
MVM
no MVM
NADPH−P450 Oxidoreductase
12.0
11.5
11.0
10
15
20
25
30
GA (weeks)

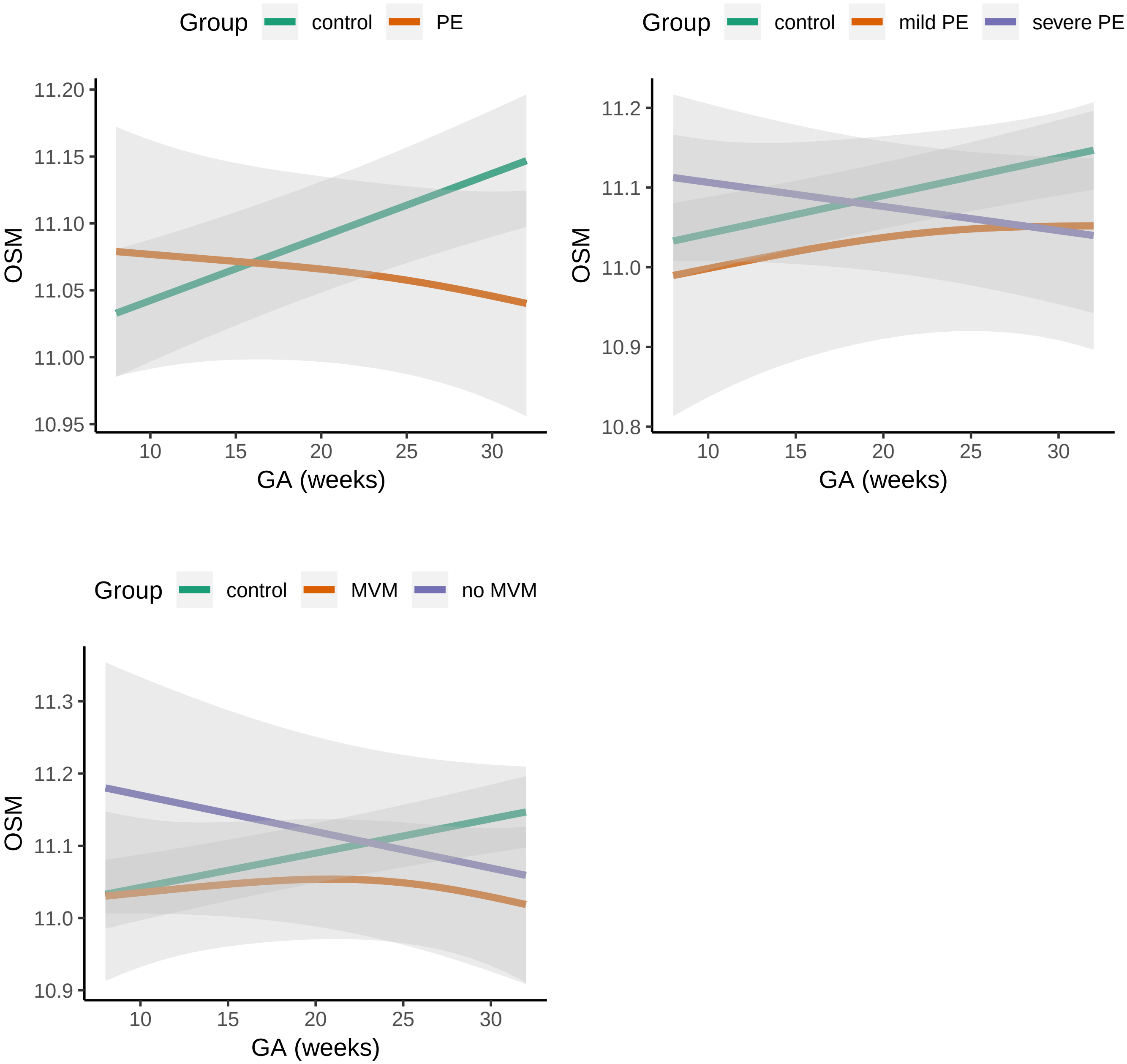
Group
control
PE
11.20
11.15
11.10
11.05
11.00
10.95
OSM
10
15
20
25
30
GA (weeks)
Group
control
mild PE
severe PE
11.2
11.1
11.0
10.9
10.8
OSM
10
15
20
25
30
GA (weeks)
Group
control
MVM
no MVM
11.3
11.2
11.1
11.0
10.9
OSM
10
15
20
25
30
GA (weeks)

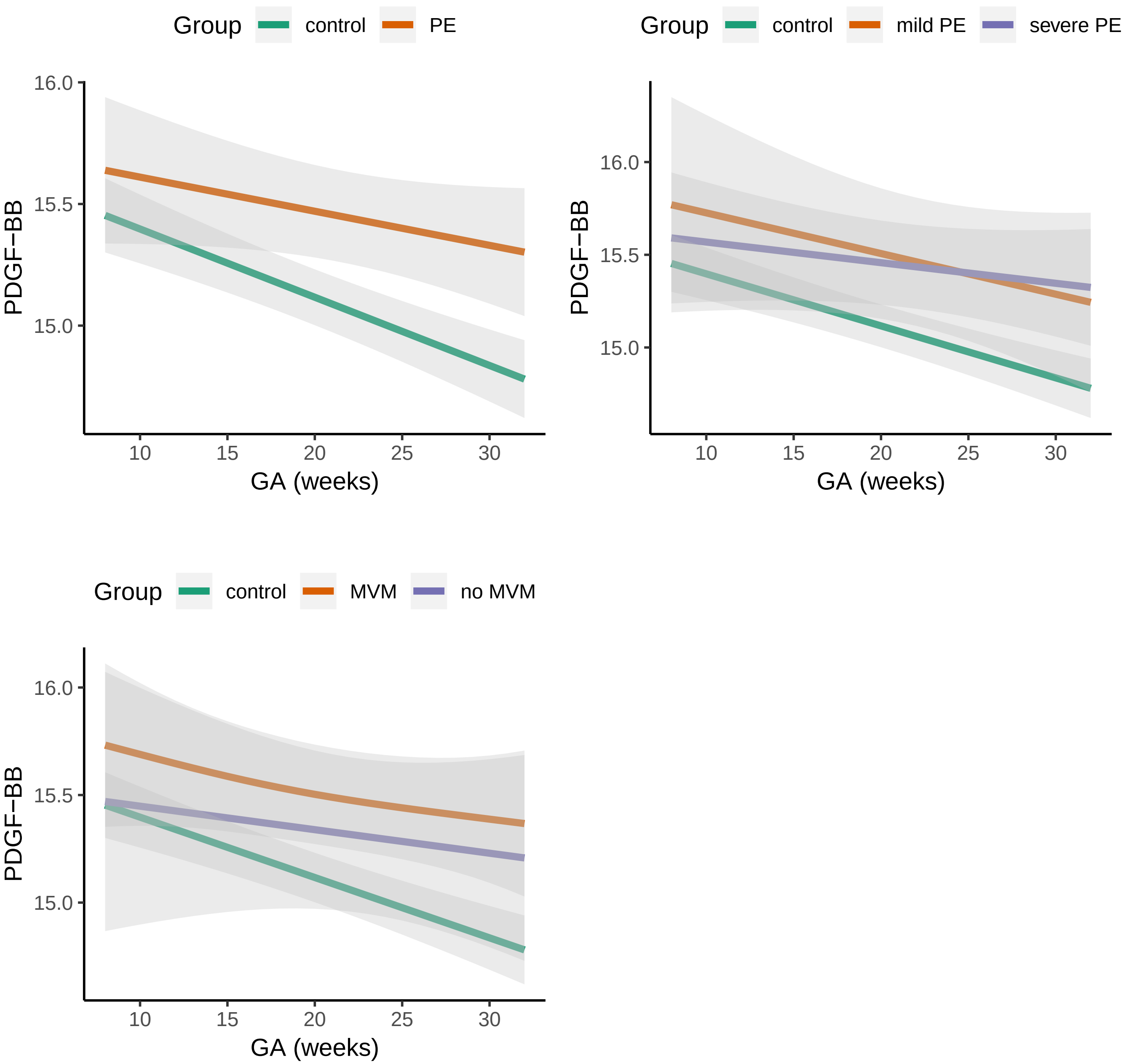

Group
control
PE
16.0
15.5
15.0
PDGF−BB
10
15
20
25
30
GA (weeks)
Group
control
mild PE
severe PE
16.0
15.5
15.0
PDGF−BB
10
15
20
25
30
GA (weeks)
Group
control
MVM
no MVM
16.0
15.5
15.0
PDGF−BB
10
15
20
25
30
GA (weeks)

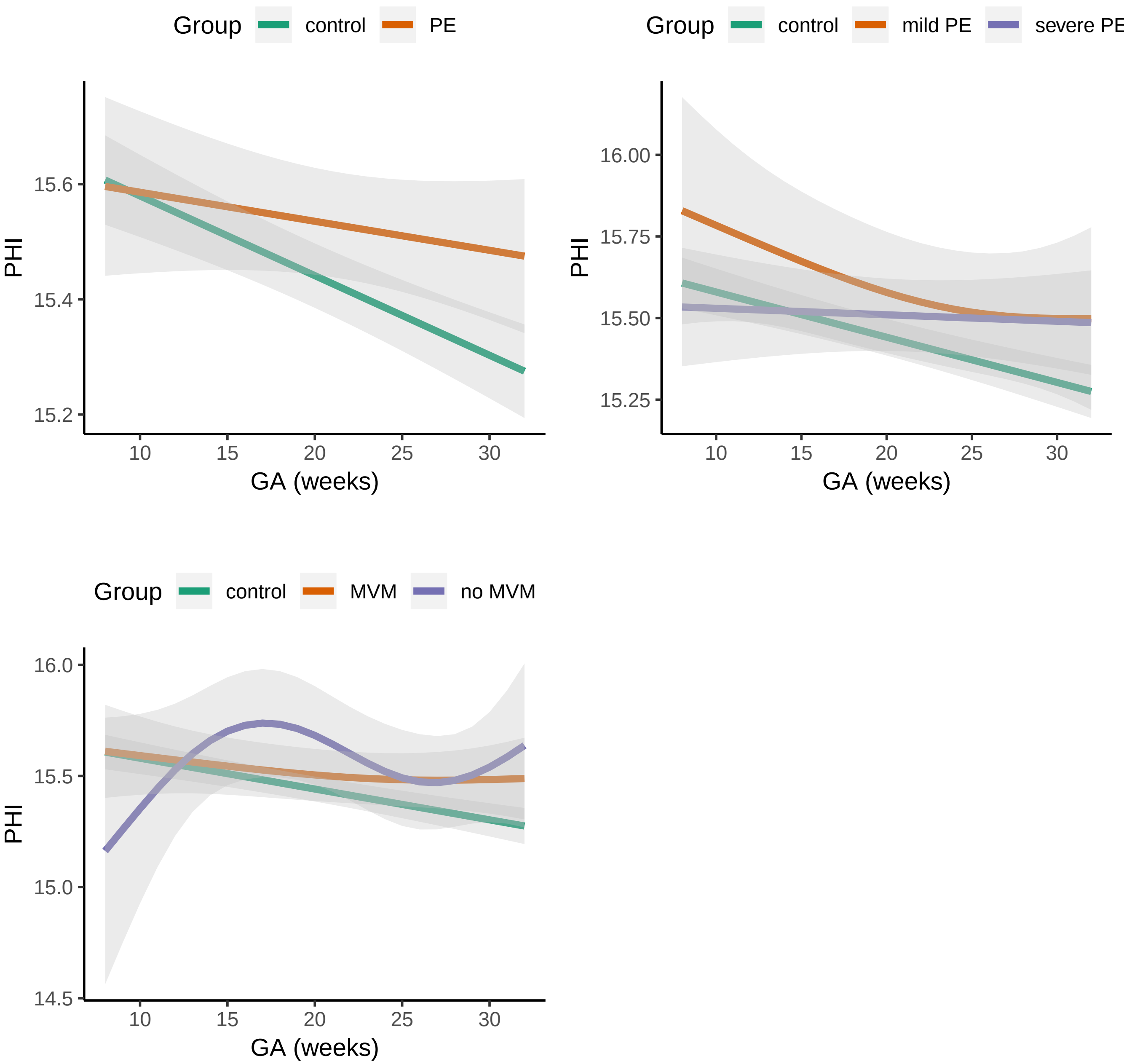
Group
control
PE
PHI
15.6
15.4
15.2
10
15
20
25
30
GA (weeks)
Group
control
mild PE
severe PE
PHI
16.00
15.75
15.50
15.25
10
15
20
25
30
GA (weeks)
Group
control
MVM
no MVM
PHI
16.0
15.5
15.0
14.5
10
15
20
25
30
GA (weeks)

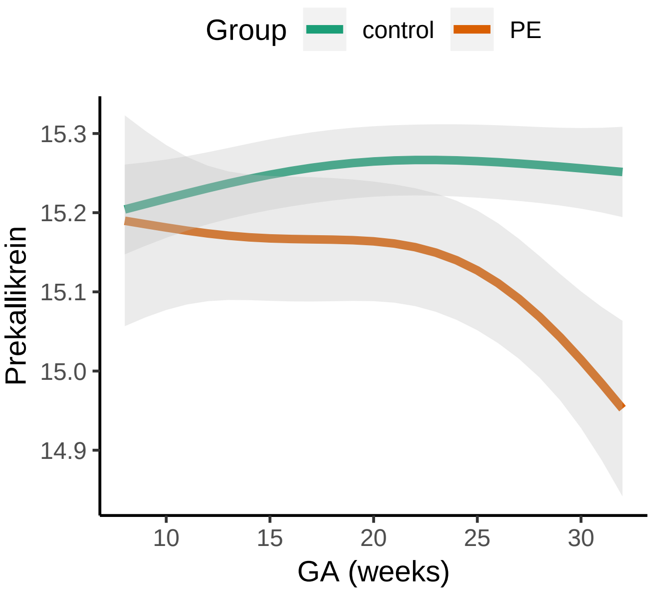
Group
control
PE
Prekallikrein
15.3
15.2
15.1
15.0
14.9
10
15
20
25
30
GA (weeks)

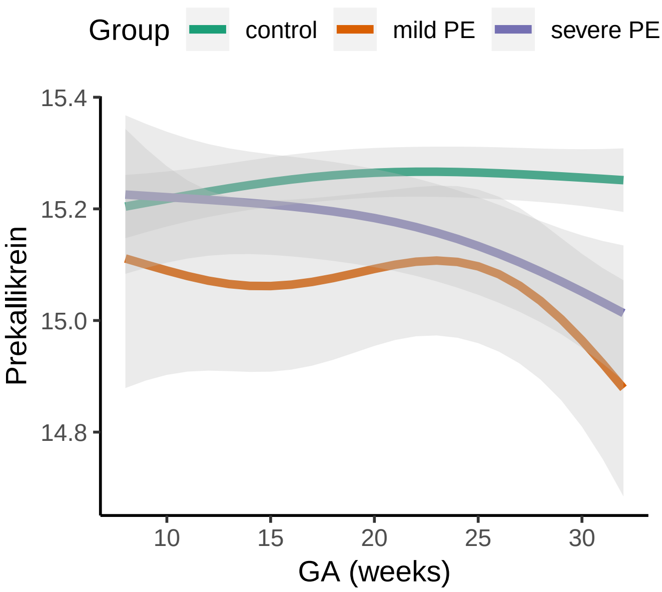
Group
control
mild PE
severe PE
Prekallikrein
15.4
15.2
15.0
14.8
10
15
20
25
30
GA (weeks)

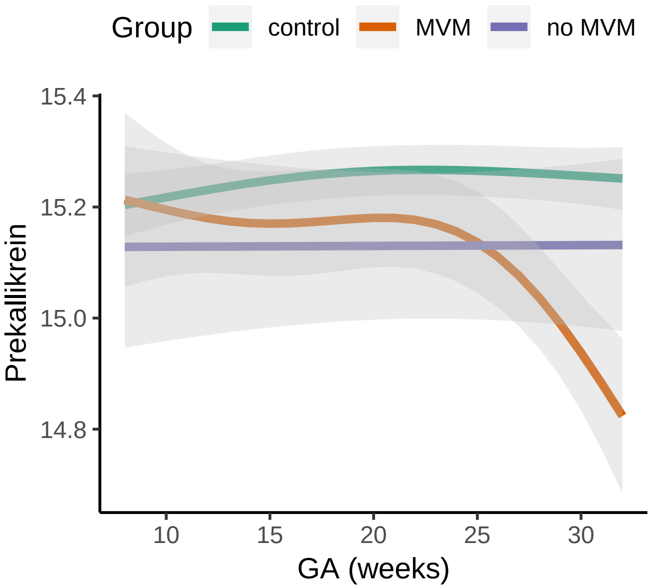
Group
control
MVM
no MVM
Prekallikrein
15.4
15.2
15.0
14.8
10
15
20
25
30
GA (weeks)

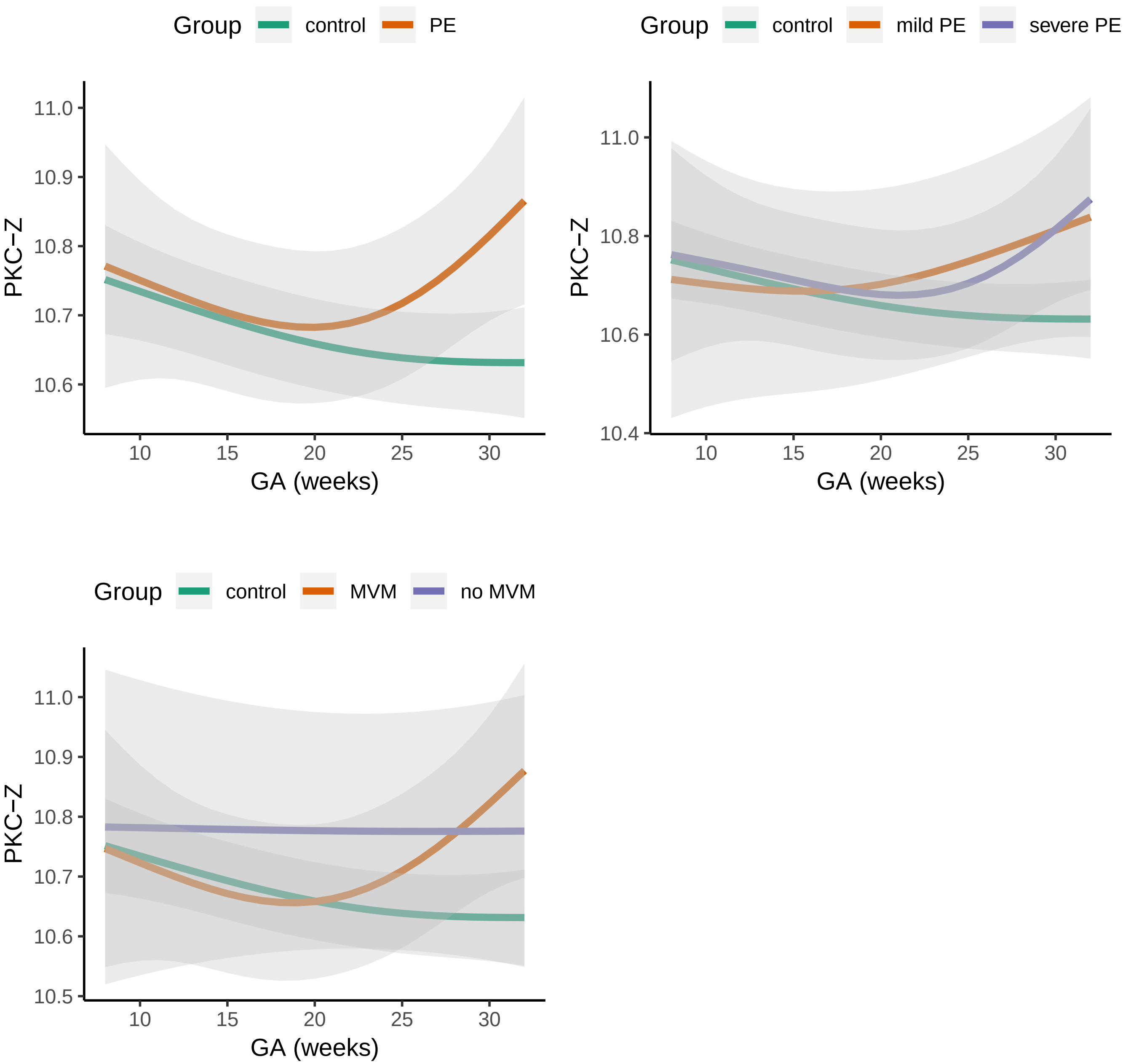

Group
control
PE
PKC−Z
11.0
10.9
10.8
10.7
10.6
10
15
20
25
30
GA (weeks)
Group
control
mild PE
severe PE
PKC−Z
11.0
10.8
10.6
10.4
10
15
20
25
30
GA (weeks)
Group
control
MVM
no MVM
PKC−Z
11.0
10.9
10.8
10.7
10.6
10.5
10
15
20
25
30
GA (weeks)

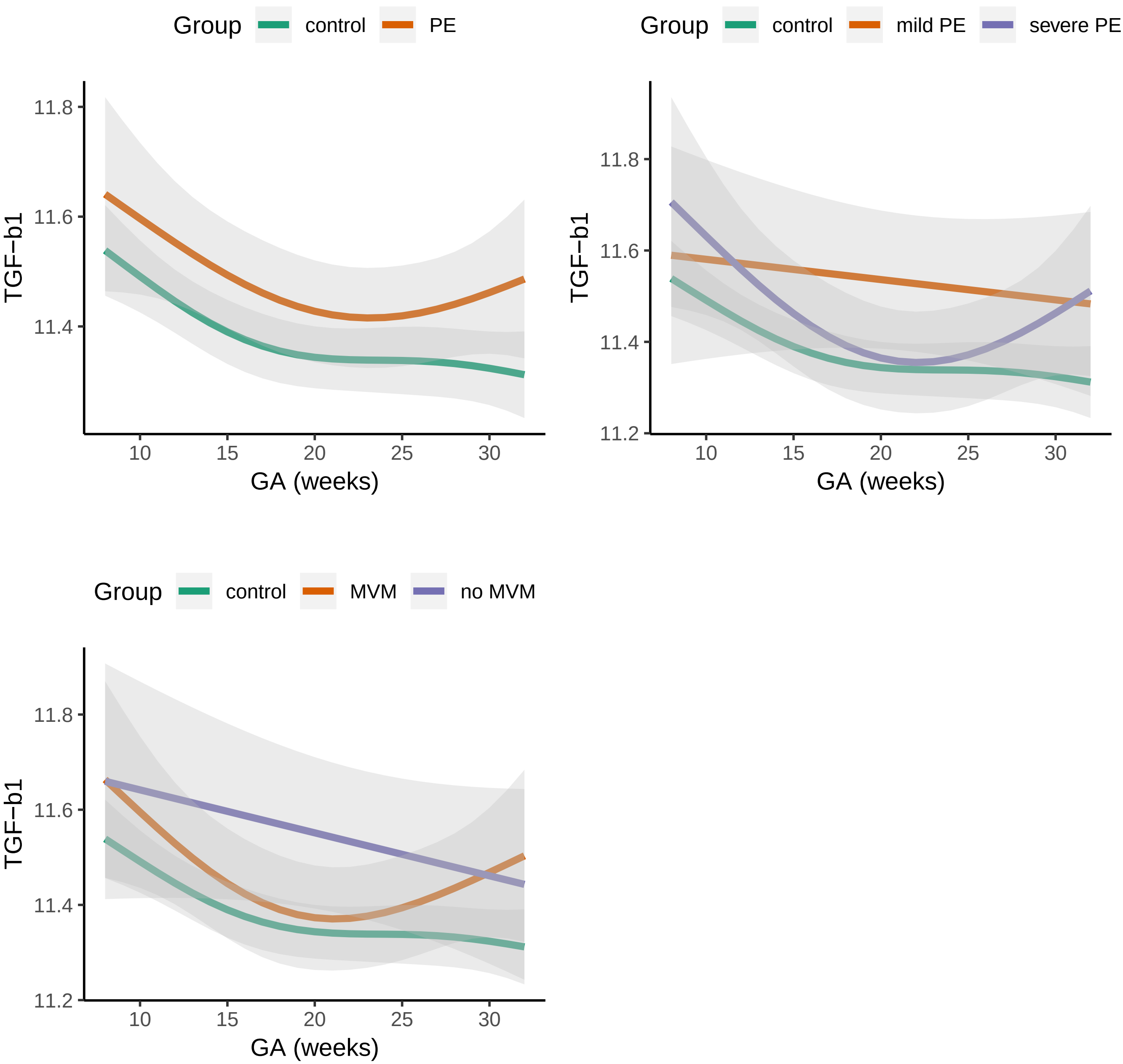
Group
control
PE
TGF−b1
11.8
11.6
11.4
10
15
20
25
30
GA (weeks)
Group
control
mild PE
severe PE
TGF−b1
11.8
11.6
11.4
11.2
10
15
20
25
30
GA (weeks)
Group
control
MVM
no MVM
TGF−b1
11.8
11.6
11.4
11.2
10
15
20
25
30
GA (weeks)

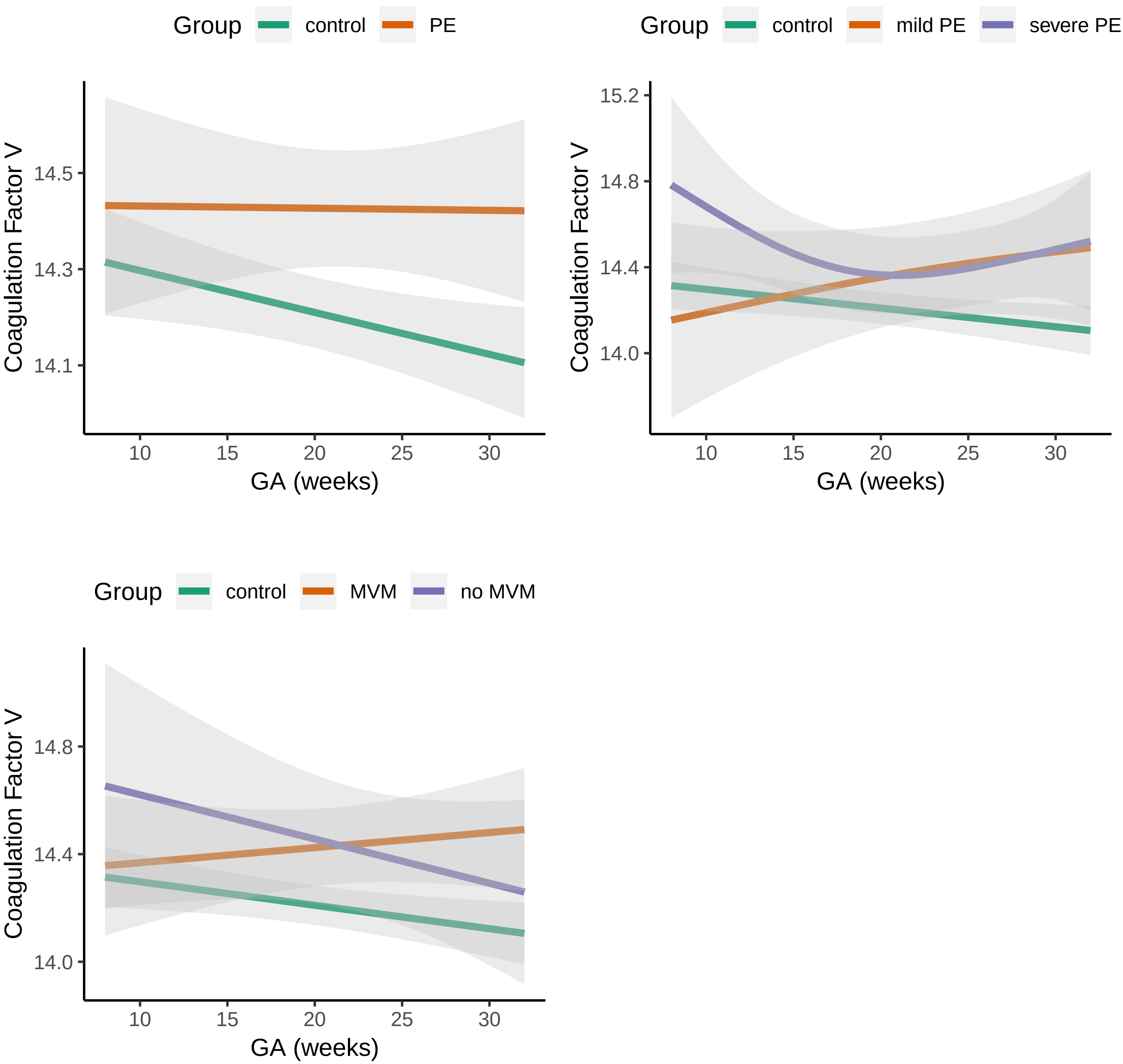

Group
control
PE
Coagulation Factor V
14.1
14.3
14.5
10
15
20
25
30
GA (weeks)
Group
control
mild PE
severe PE
Coagulation Factor V
14.0
14.4
14.8
15.2
10
15
20
25
30
GA (weeks)
Group
control
MVM
no MVM
Coagulation Factor V
14.0
14.4
14.8
10
15
20
25
30
GA (weeks)

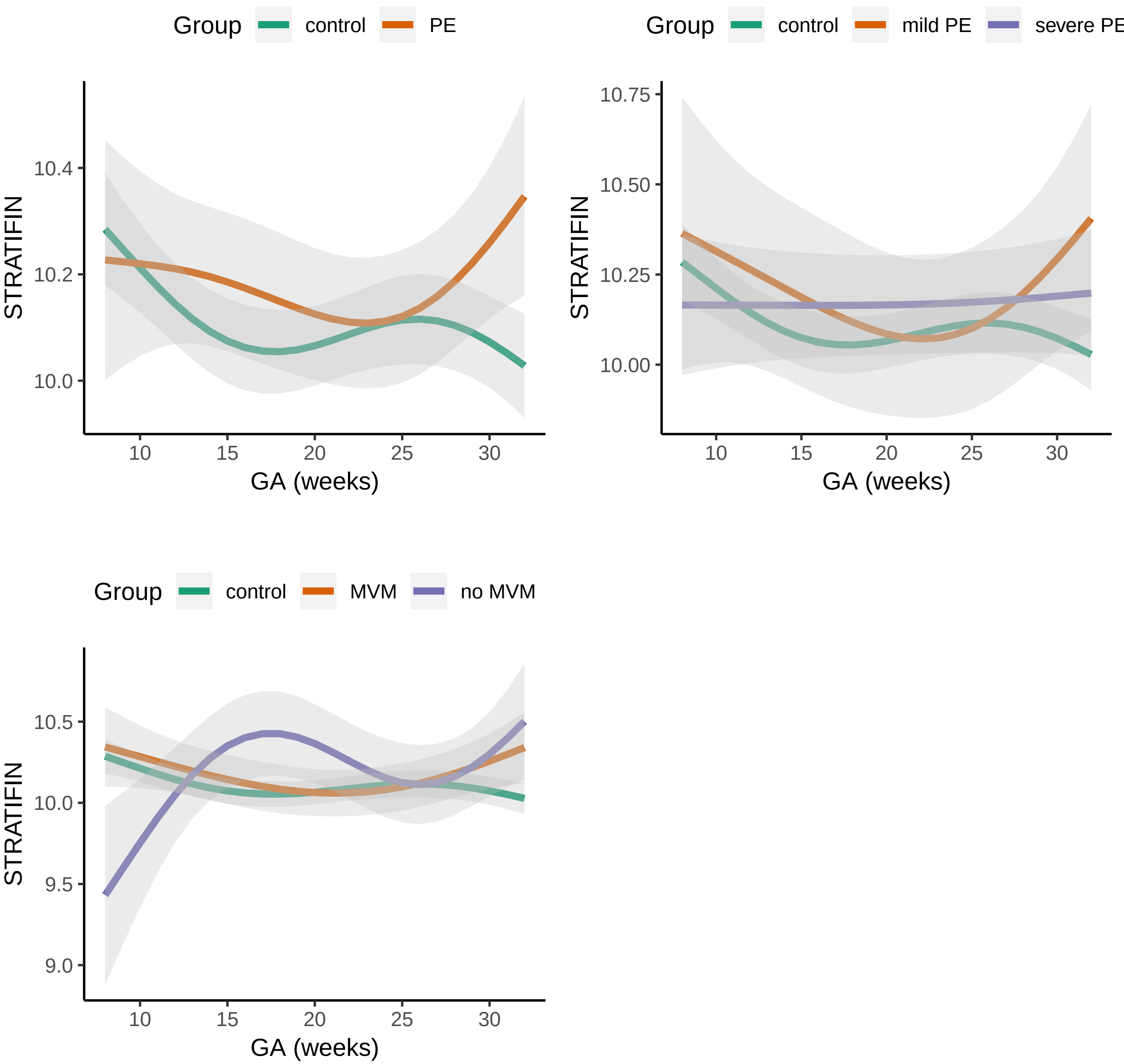
Group
control
PE
STRATIFIN
10.4
10.2
10.0
10
15
20
25
30
GA (weeks)
Group
control
mild PE
severe PE
STRATIFIN
10.75
10.50
10.25
10.00
10
15
20
25
30
GA (weeks)
Group
control
MVM
no MVM
STRATIFIN
10.5
10.0
9.5
9.0
10
15
20
25
30
GA (weeks)

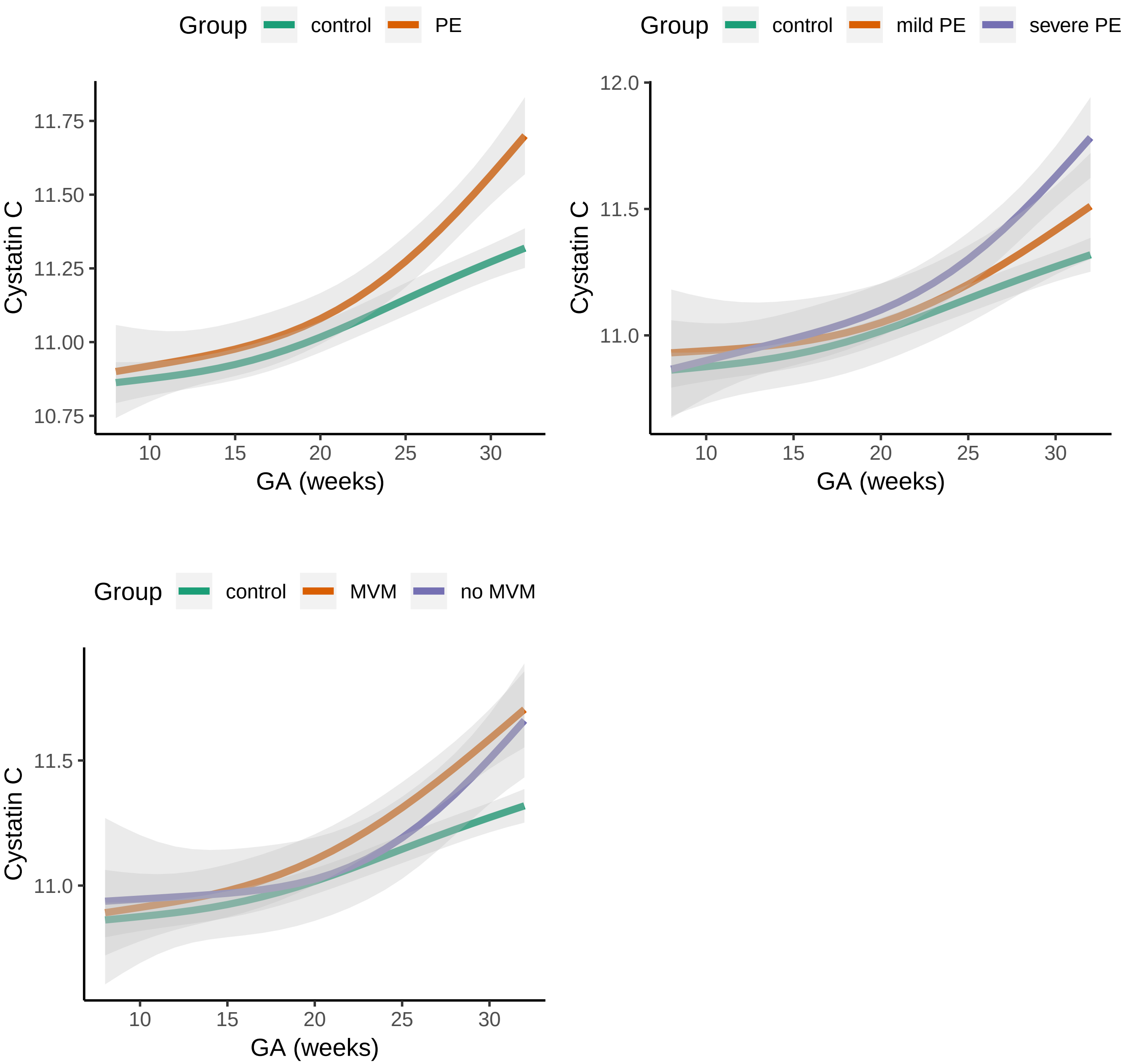
Group
control
PE
Cystatin C
11.75
11.50
11.25
11.00
10.75
10
15
20
25
30
GA (weeks)
Group
control
mild PE
severe PE
Cystatin C
12.0
11.5
11.0
10
15
20
25
30
GA (weeks)
Group
control
MVM
no MVM
Cystatin C
11.5
11.0
10
15
20
25
30
GA (weeks)

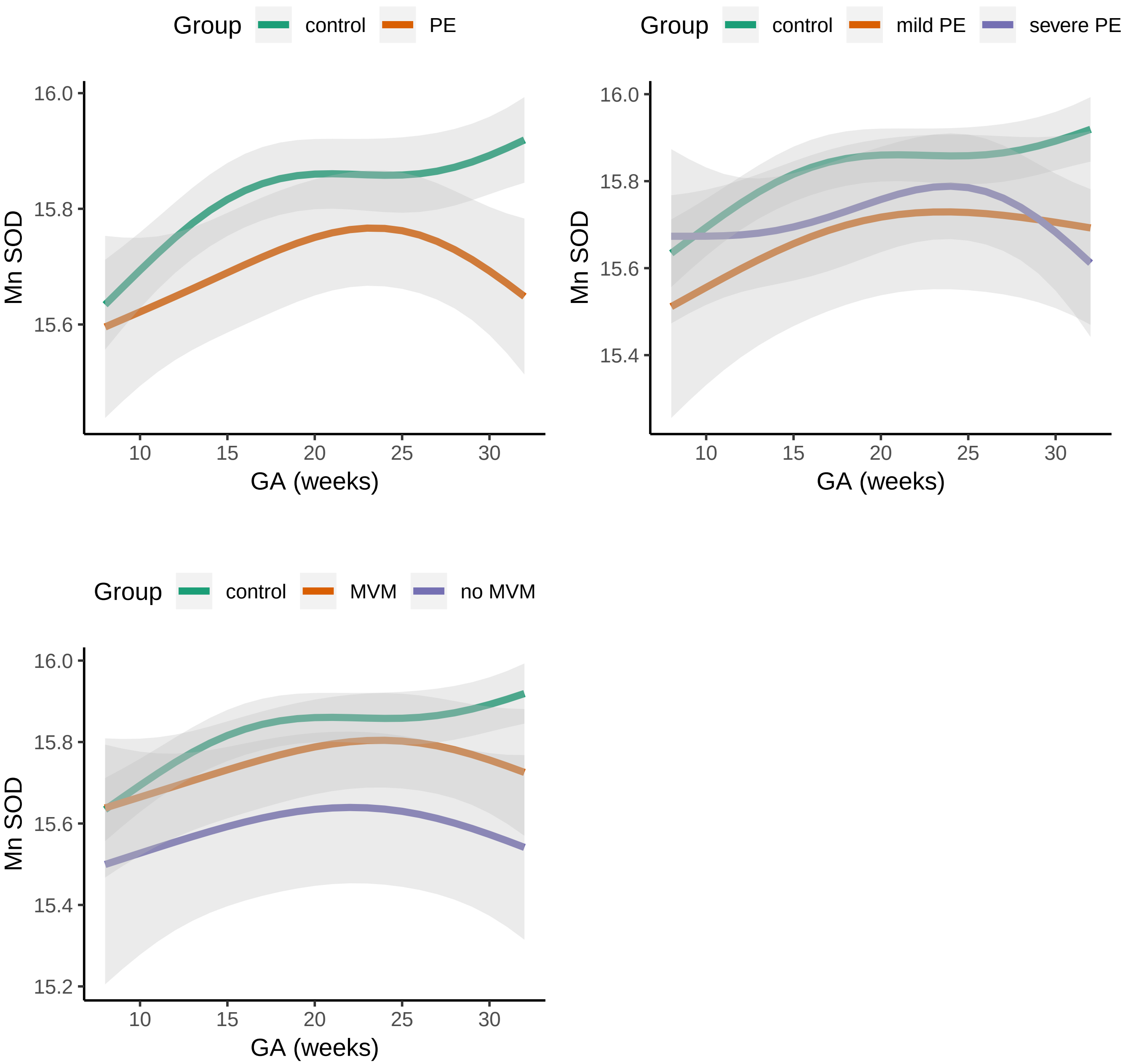

Group
control
PE
16.0
15.8
15.6
Mn SOD
10
15
20
25
30
GA (weeks)
Group
control
mild PE
severe PE
16.0
15.8
15.6
15.4
Mn SOD
10
15
20
25
30
GA (weeks)
Group
control
MVM
no MVM
16.0
15.8
15.6
15.4
15.2
Mn SOD
10
15
20
25
30
GA (weeks)

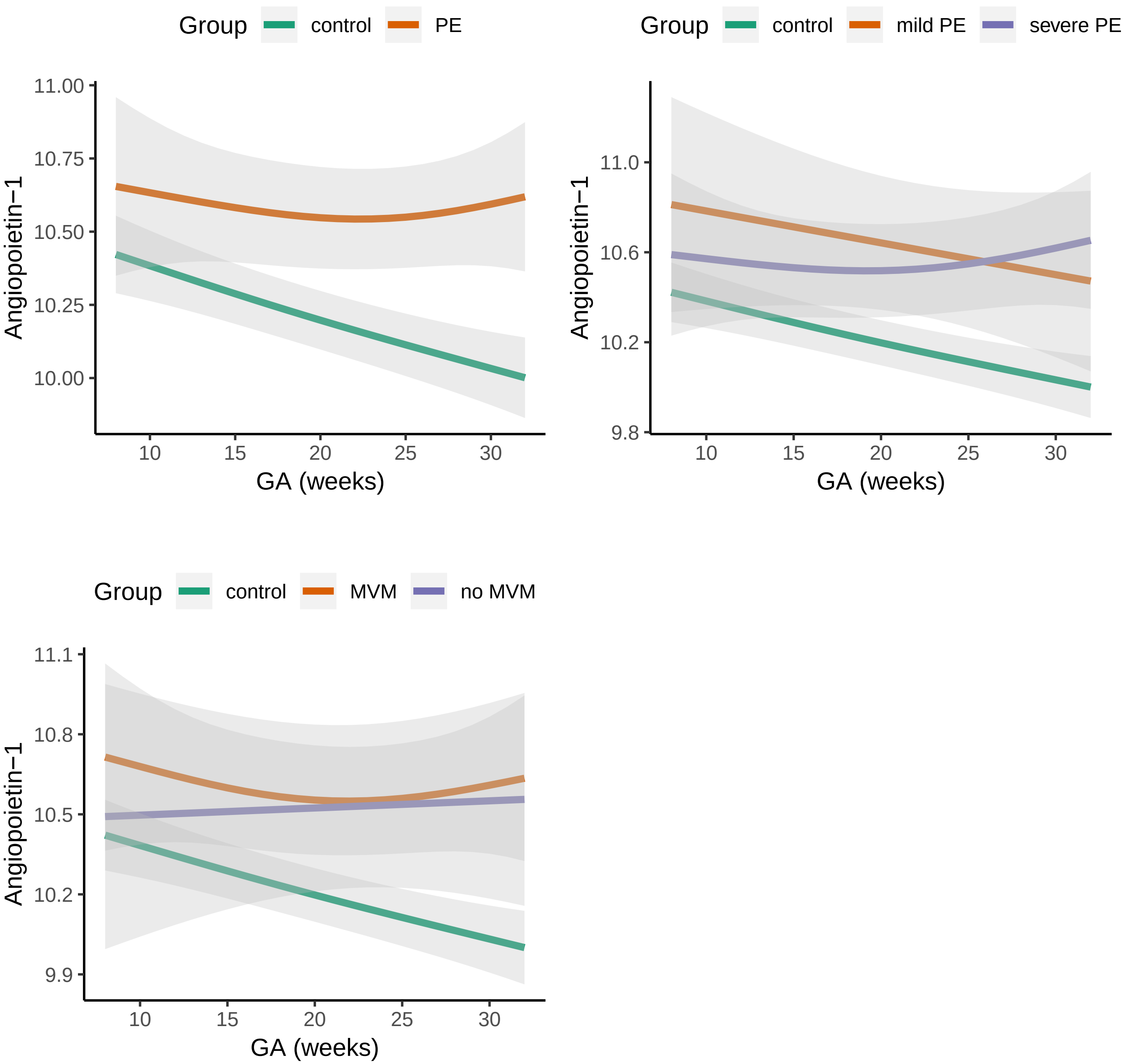

Group
control
PE
Angiopoietin−1
11.00
10.75
10.50
10.25
10.00
10
15
20
25
30
GA (weeks)
Group
control
mild PE
severe PE
Angiopoietin−1
11.0
10.6
10.2
9.8
10
15
20
25
30
GA (weeks)
Group
control
MVM
no MVM
Angiopoietin−1
11.1
10.8
10.5
10.2
9.9
10
15
20
25
30
GA (weeks)

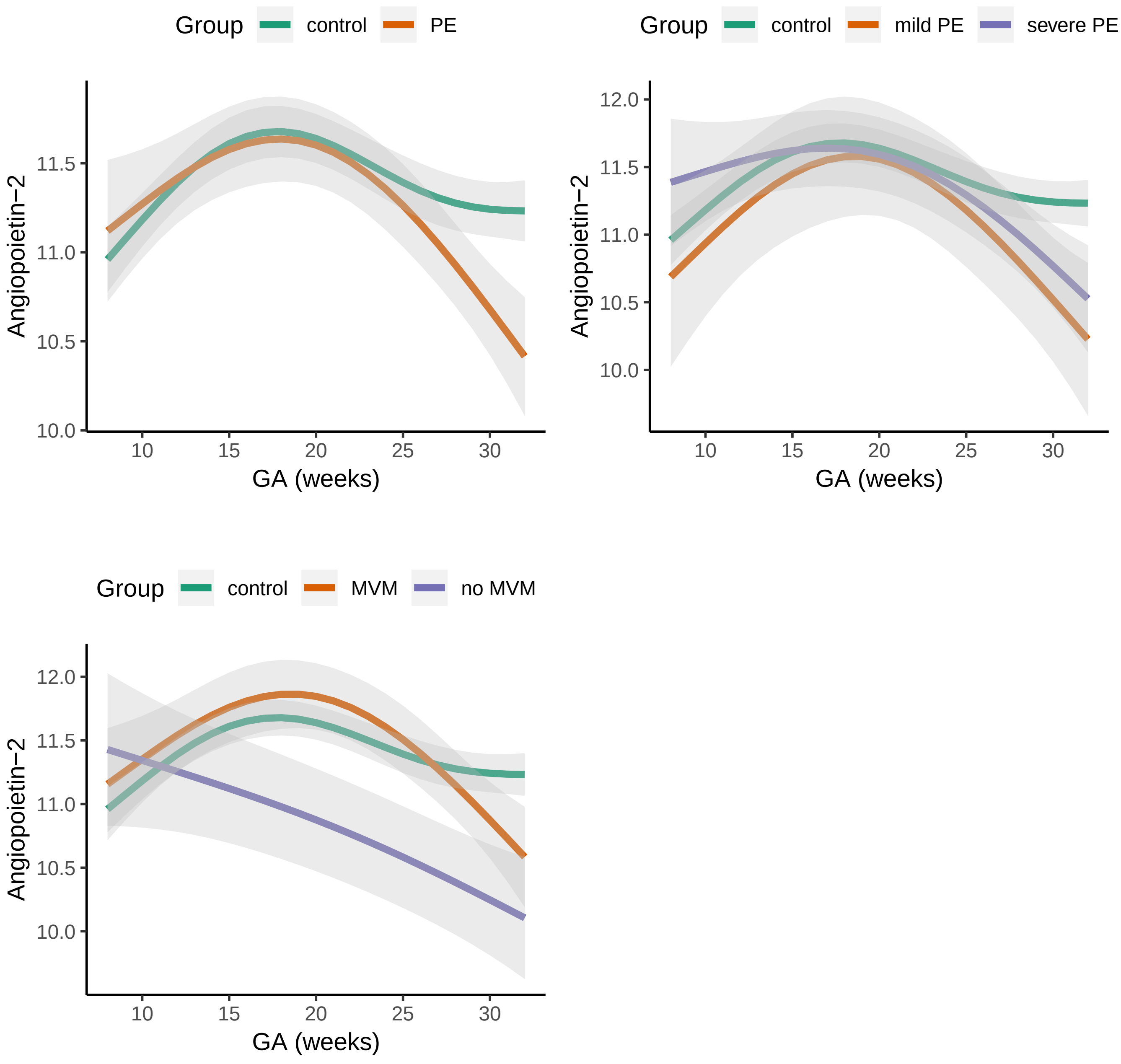

Group
control
PE
Angiopoietin−2
11.5
11.0
10.5
10.0
10
15
20
25
30
GA (weeks)
Group
control
mild PE
severe PE
Angiopoietin−2
12.0
11.5
11.0
10.5
10.0
10
15
20
25
30
GA (weeks)
Group
control
MVM
no MVM
Angiopoietin−2
12.0
11.5
11.0
10.5
10.0
10
15
20
25
30
GA (weeks)

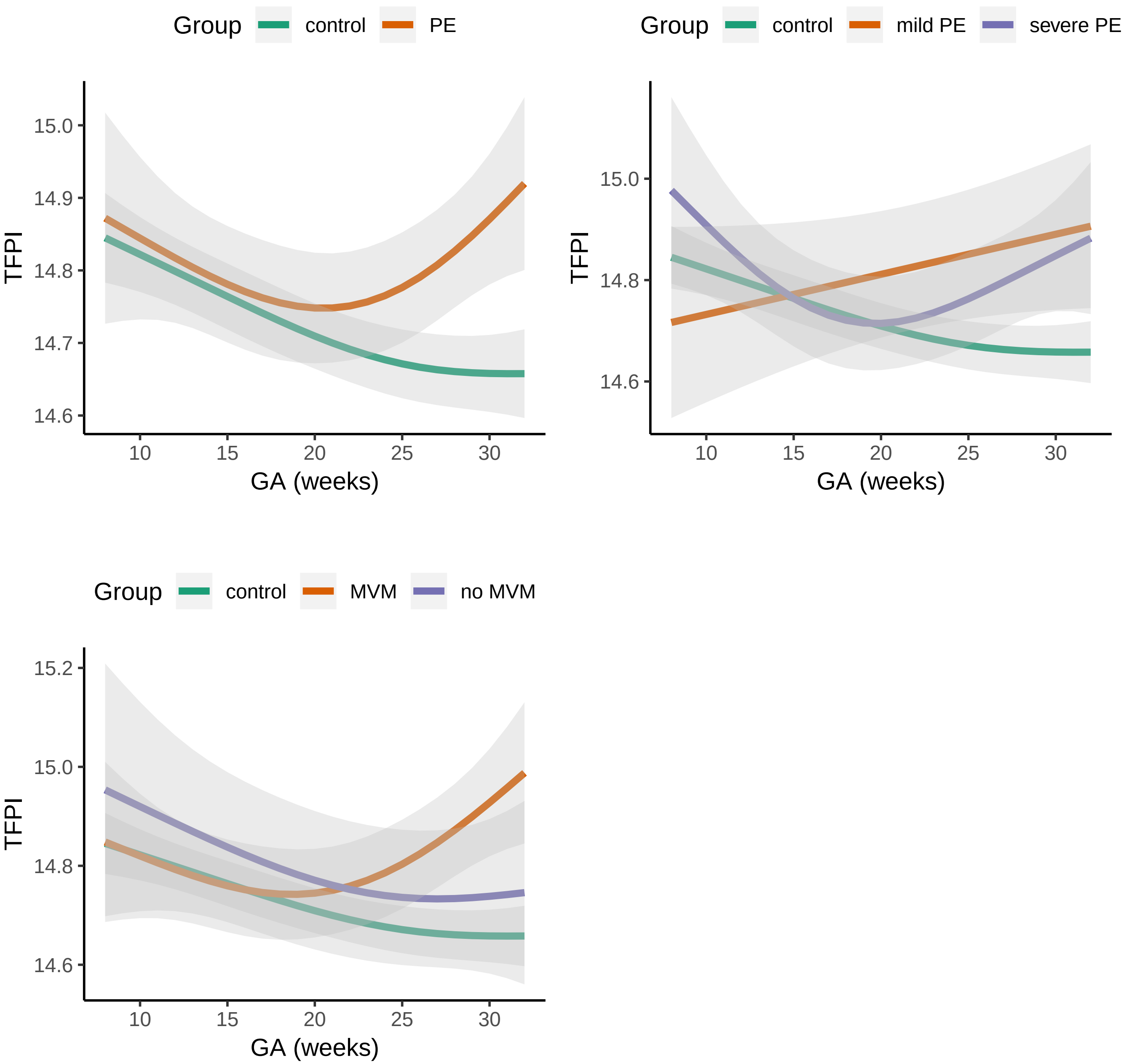

Group
control
PE
TFPI
15.0
14.9
14.8
14.7
14.6
10
15
20
25
30
GA (weeks)
Group
control
mild PE
severe PE
TFPI
15.0
14.8
14.6
10
15
20
25
30
GA (weeks)
Group
control
MVM
no MVM
TFPI
15.2
15.0
14.8
14.6
10
15
20
25
30
GA (weeks)

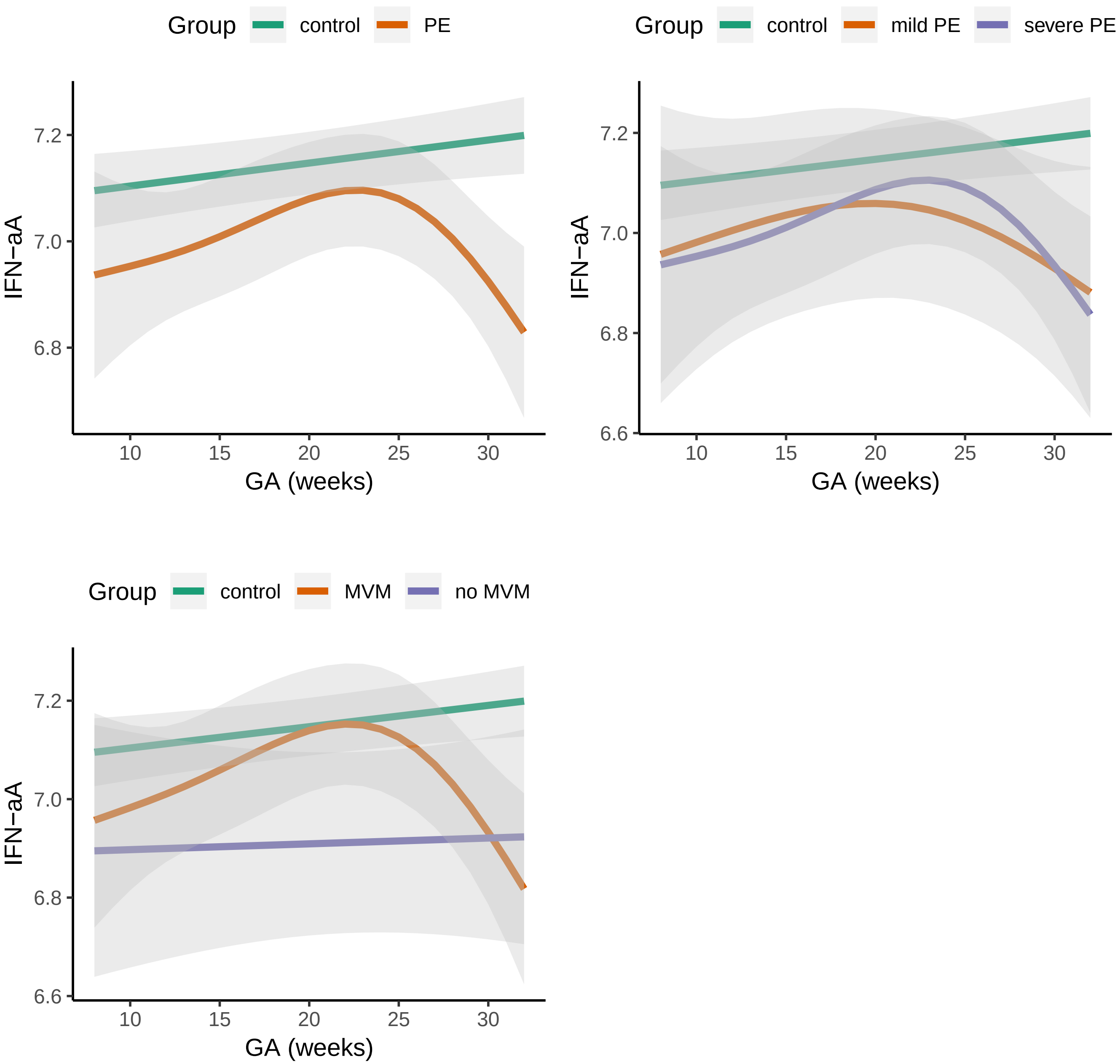
Group
control
PE
IFN−aA
7.2
7.0
6.8
10
15
20
25
30
GA (weeks)
Group
control
mild PE
severe PE
IFN−aA
7.2
7.0
6.8
6.6
10
15
20
25
30
GA (weeks)
Group
control
MVM
no MVM
IFN−aA
7.2
7.0
6.8
6.6
10
15
20
25
30
GA (weeks)

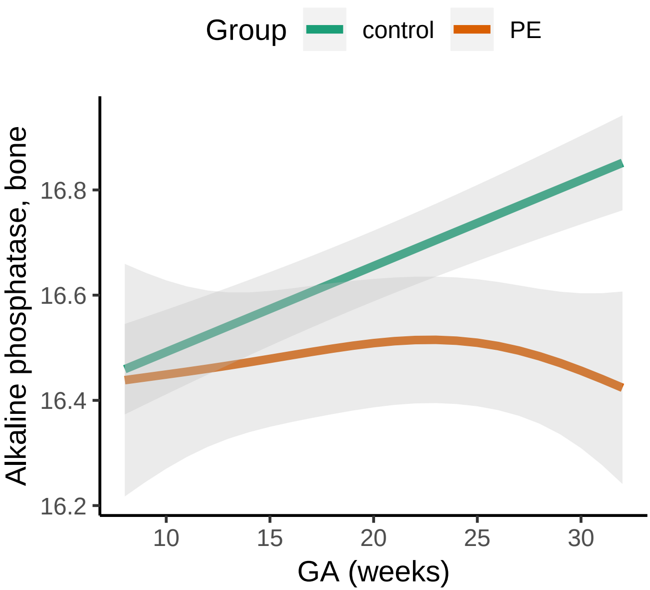

Group
control
PE
Alkaline phosphatase, bone
16.8
16.6
16.4
16.2
10
15
20
25
30
GA (weeks)

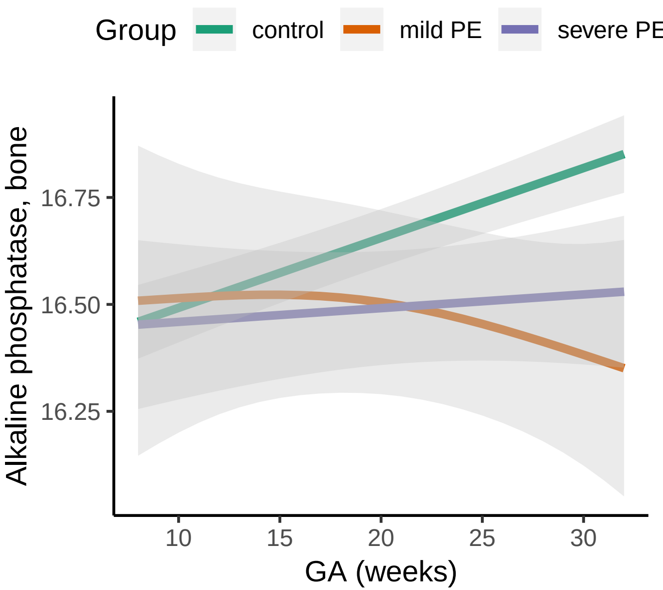

Group
control
mild PE
severe PE
Alkaline phosphatase, bone
16.75
16.50
16.25
10
15
20
25
30
GA (weeks)

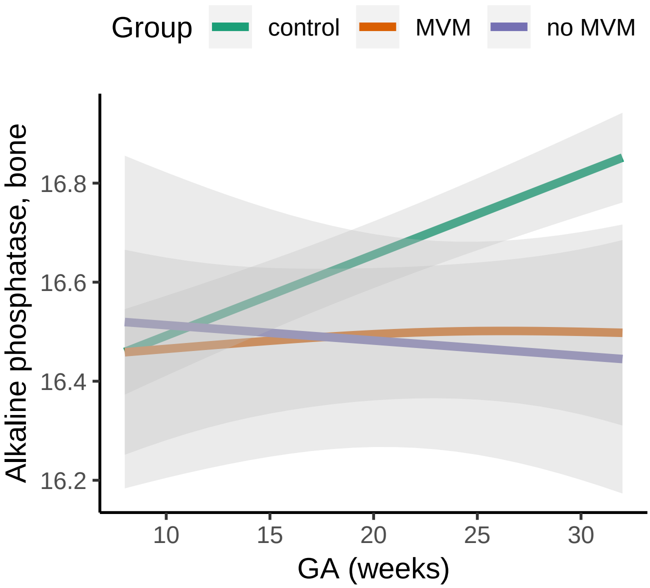

Group
control
MVM
no MVM
Alkaline phosphatase, bone
16.8
16.6
16.4
16.2
10
15
20
25
30
GA (weeks)

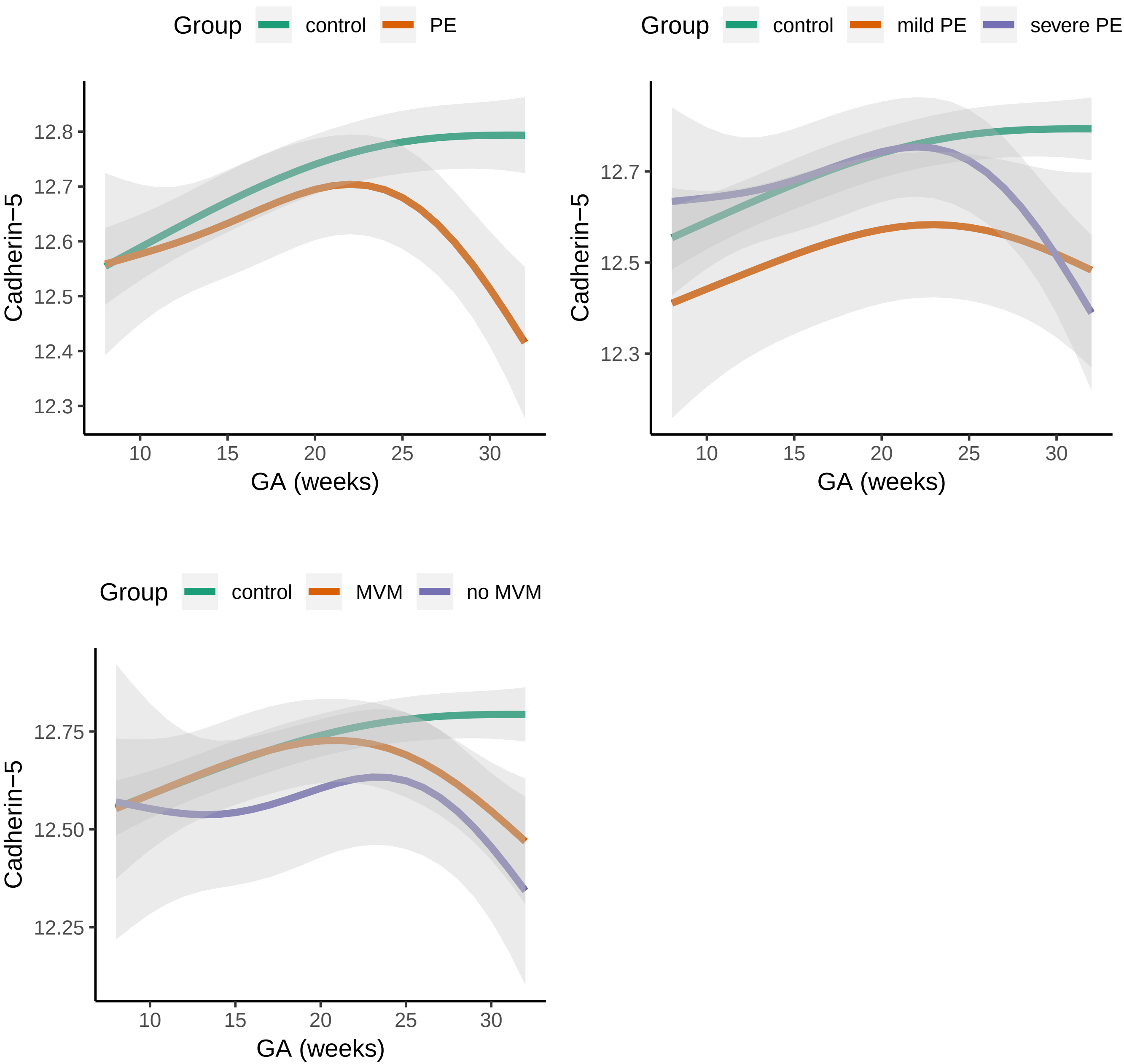
Group
control
PE
Cadherin−5
12.8
12.7
12.6
12.5
12.4
12.3
10
15
20
25
30
GA (weeks)
Group
control
mild PE
severe PE
Cadherin−5
12.7
12.5
12.3
10
15
20
25
30
GA (weeks)
Group
control
MVM
no MVM
Cadherin−5
12.75
12.50
12.25
10
15
20
25
30
GA (weeks)

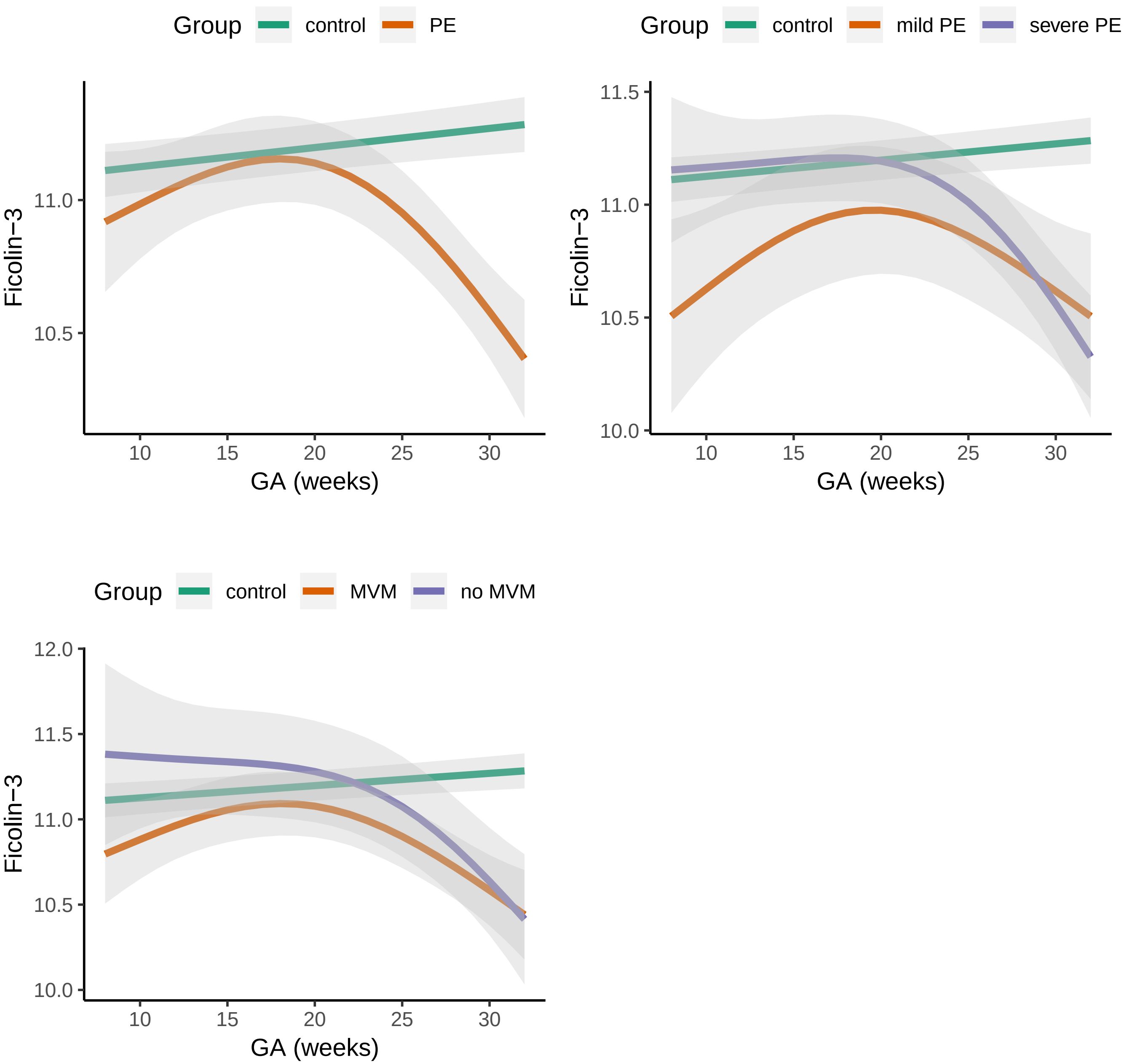
Group
control
PE
Ficolin−3
GA (weeks)
Group
control
mild PE
severe PE
Ficolin−3
GA (weeks)
Group
control
MVM
no MVM
Ficolin−3
GA (weeks)

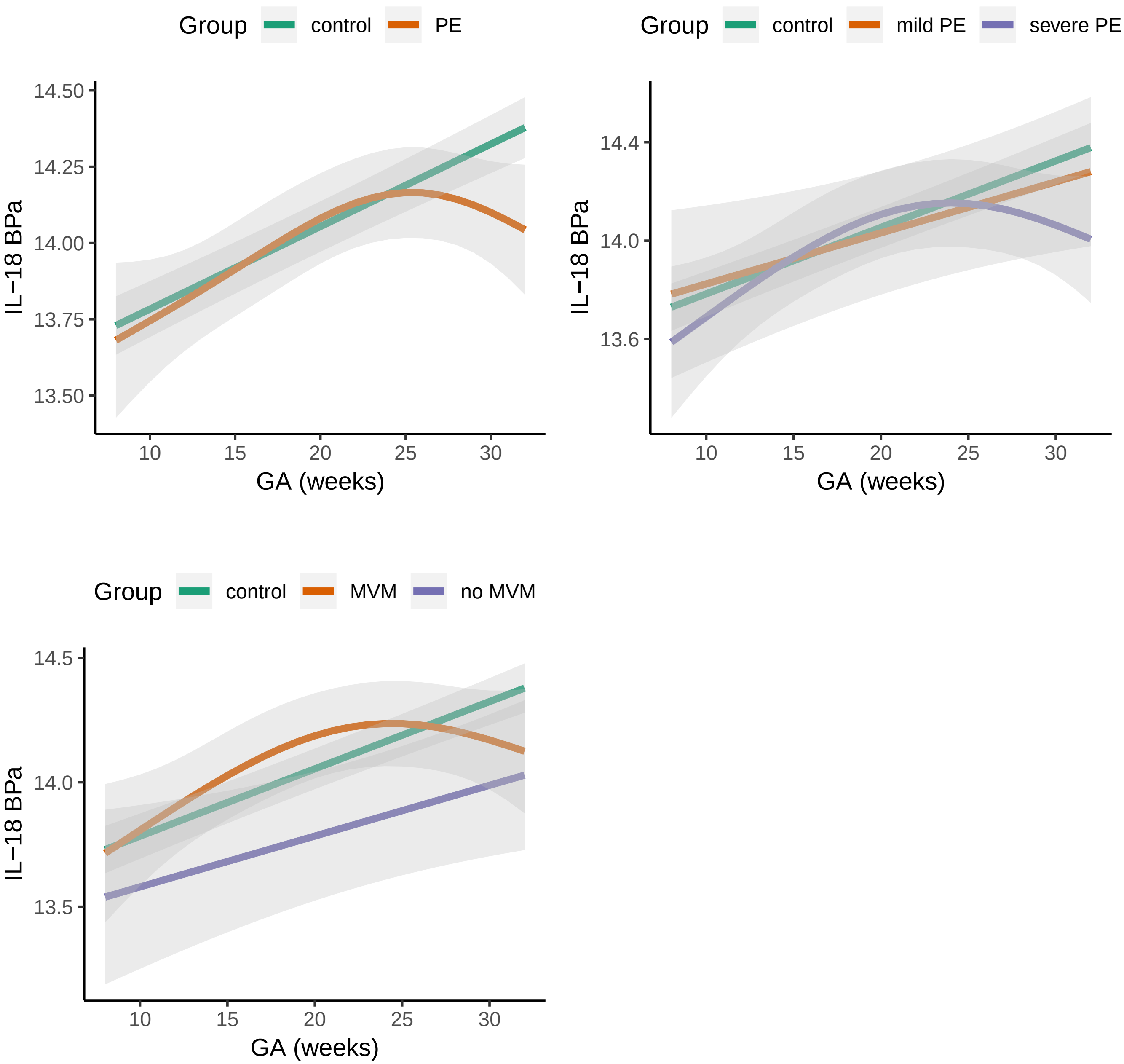

Group
control
PE
mild PE
severe PE
MVM
no MVM
IL−18 BPa
GA (weeks)
14.50
14.25
14.00
13.75
13.50
14.4
14.0
13.6
14.5
13.5
10
15
20
25
30

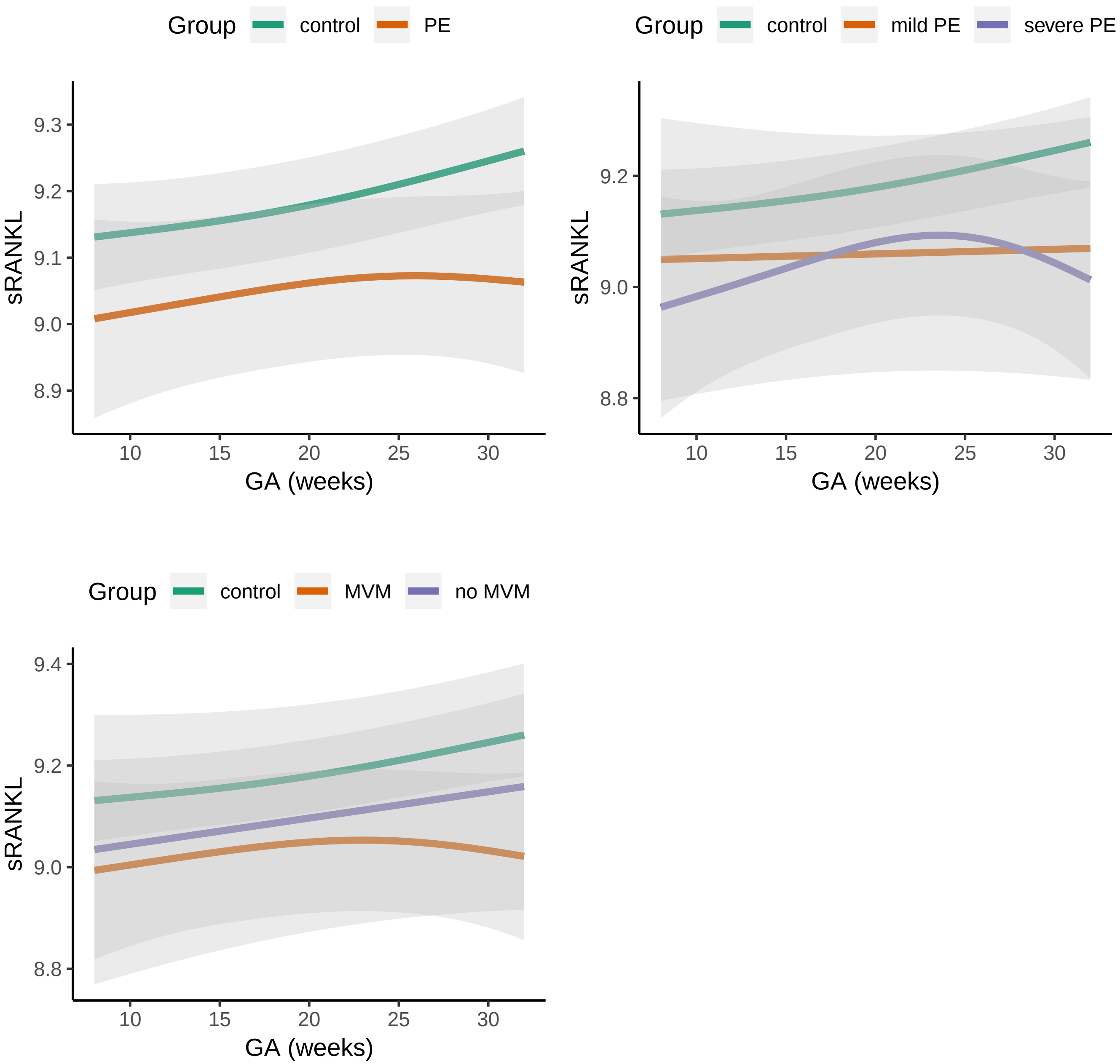

Group
control
PE
sRANKL
9.3
9.2
9.1
9.0
8.9
10
15
20
25
30
GA (weeks)
Group
control
mild PE
severe PE
sRANKL
9.2
9.0
8.8
10
15
20
25
30
GA (weeks)
Group
control
MVM
no MVM
sRANKL
9.4
9.2
9.0
8.8
10
15
20
25
30
GA (weeks)

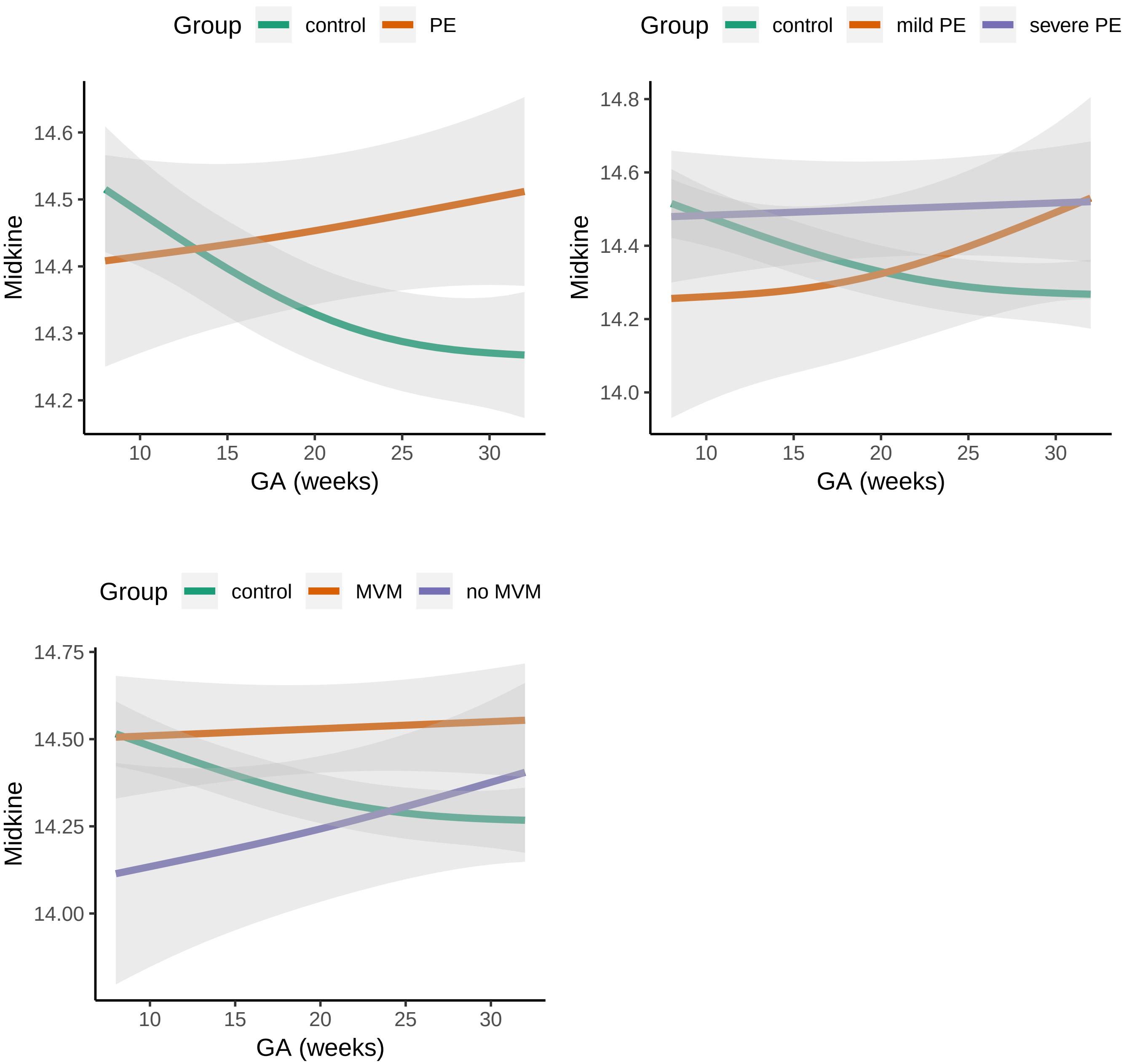
Group
control
PE
Midkine
14.6
14.5
14.4
14.3
14.2
10
15
20
25
30
GA (weeks)
Group
control
mild PE
severe PE
Midkine
14.8
14.6
14.4
14.2
14.0
10
15
20
25
30
GA (weeks)
Group
control
MVM
no MVM
Midkine
14.75
14.50
14.25
14.00
10
15
20
25
30
GA (weeks)

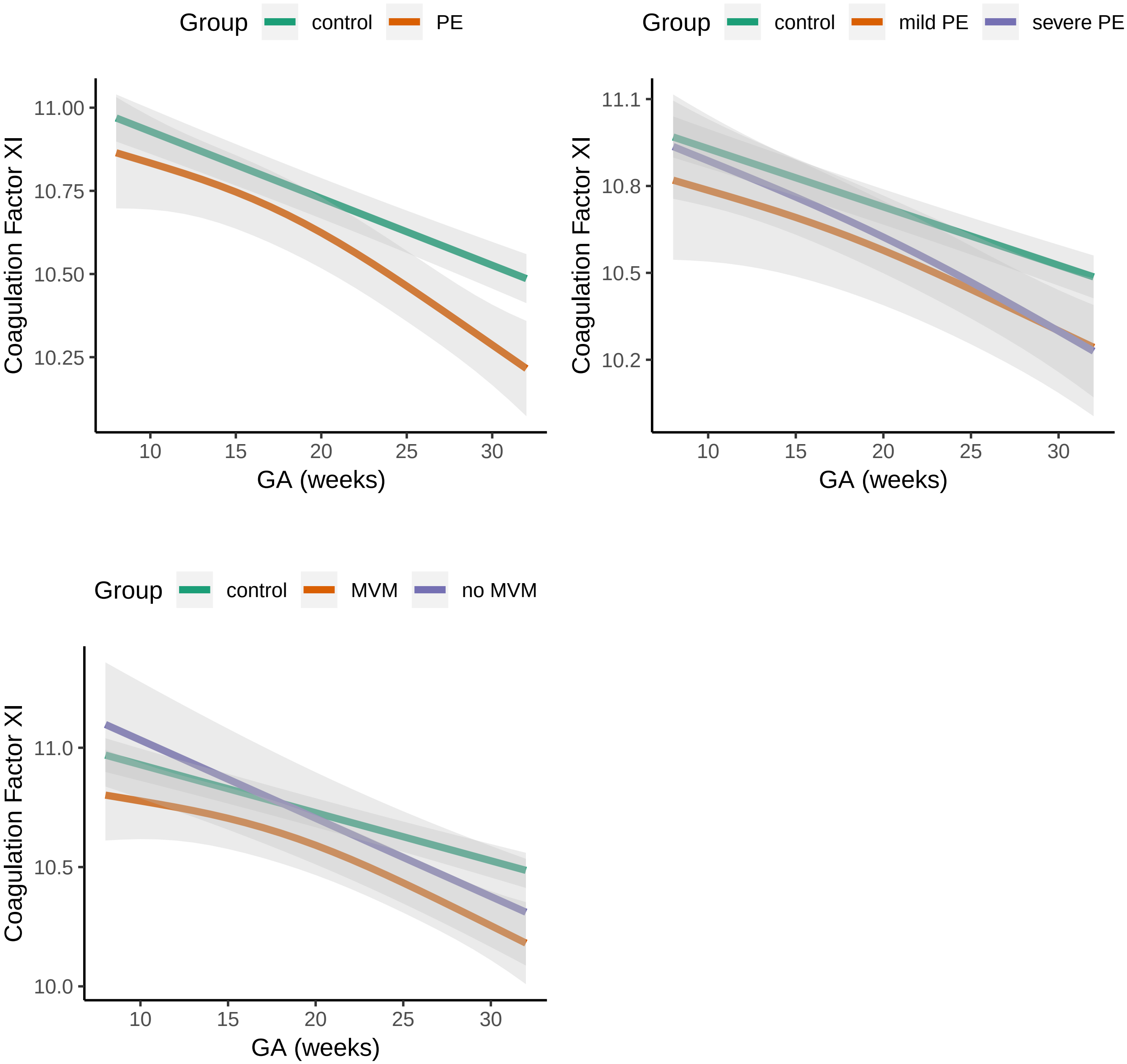
Group
control
PE
Coagulation Factor XI
11.00
10.75
10.50
10.25
10
15
20
25
30
GA (weeks)
Group
control
mild PE
severe PE
Coagulation Factor XI
11.1
10.8
10.5
10.2
10
15
20
25
30
GA (weeks)
Group
control
MVM
no MVM
Coagulation Factor XI
11.0
10.5
10.0
10
15
20
25
30
GA (weeks)

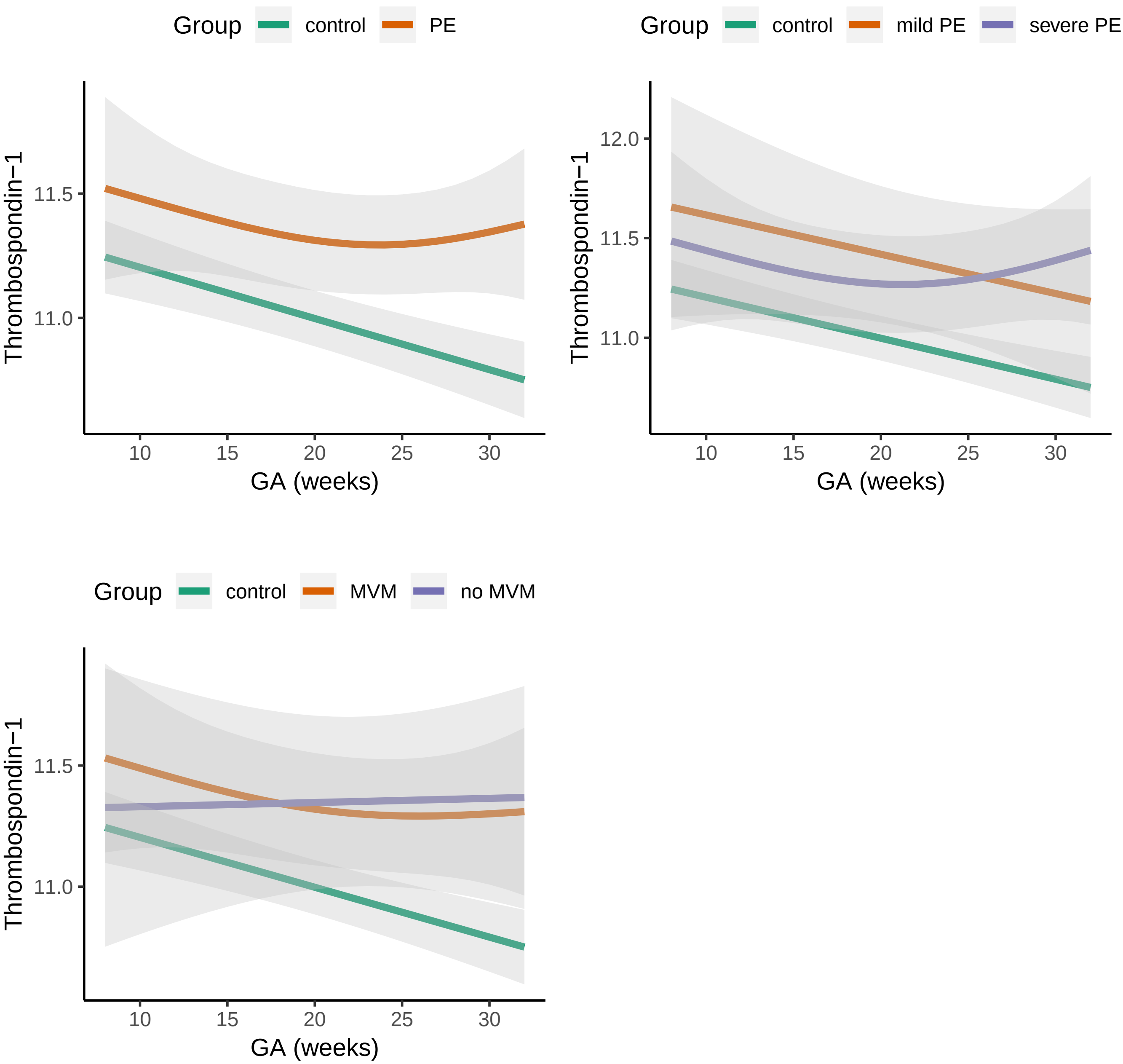

Group
control
PE
Thrombospondin−1
11.5
11.0
10
15
20
25
30
GA (weeks)
Group
control
mild PE
severe PE
Thrombospondin−1
12.0
11.5
11.0
10
15
20
25
30
GA (weeks)
Group
control
MVM
no MVM
Thrombospondin−1
11.5
11.0
10
15
20
25
30
GA (weeks)

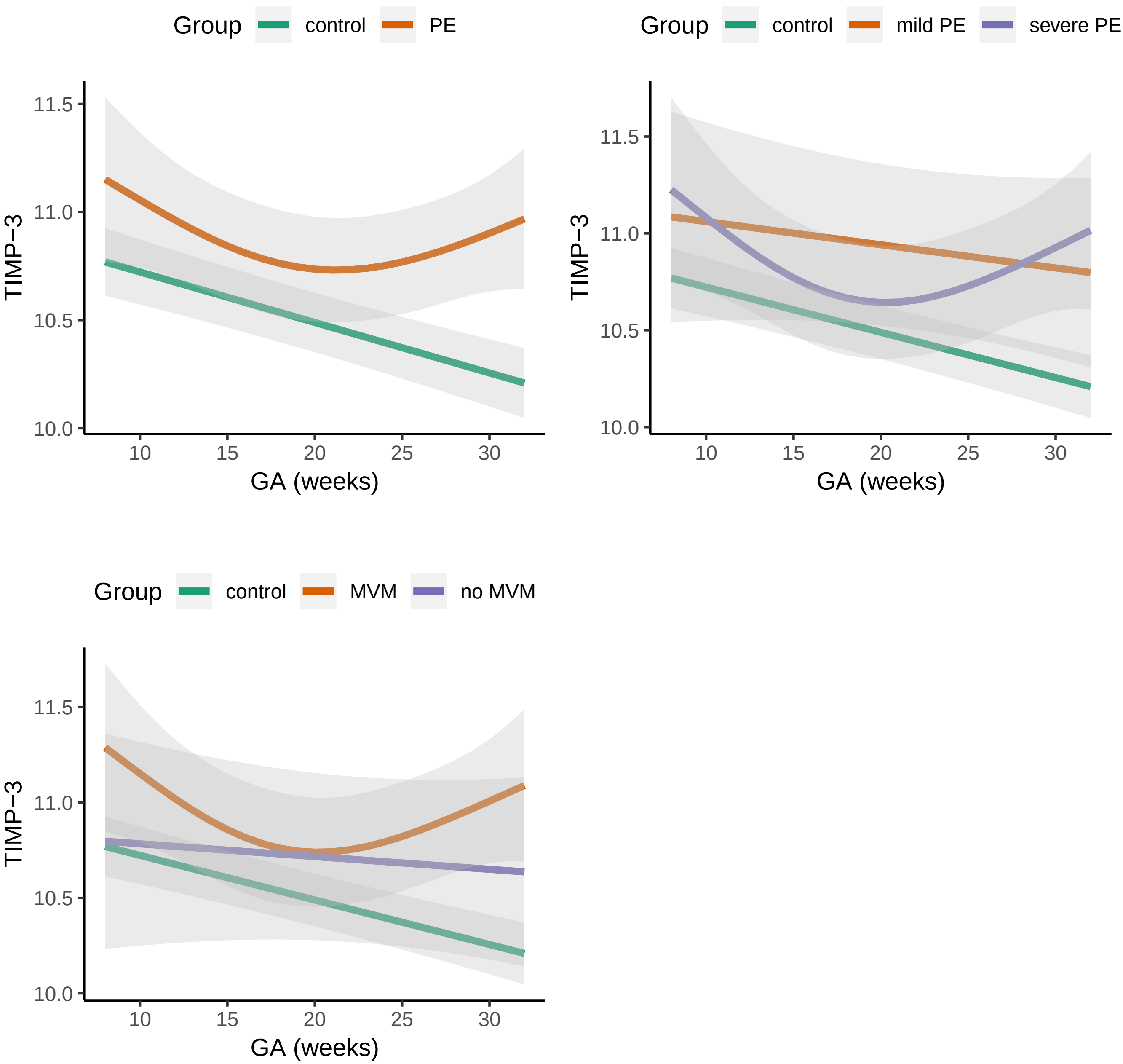
Group
control
PE
mild PE
severe PE
MVM
no MVM
TIMP−3
GA (weeks)
10.0
10.5
11.0
11.5
10
15
20
25
30

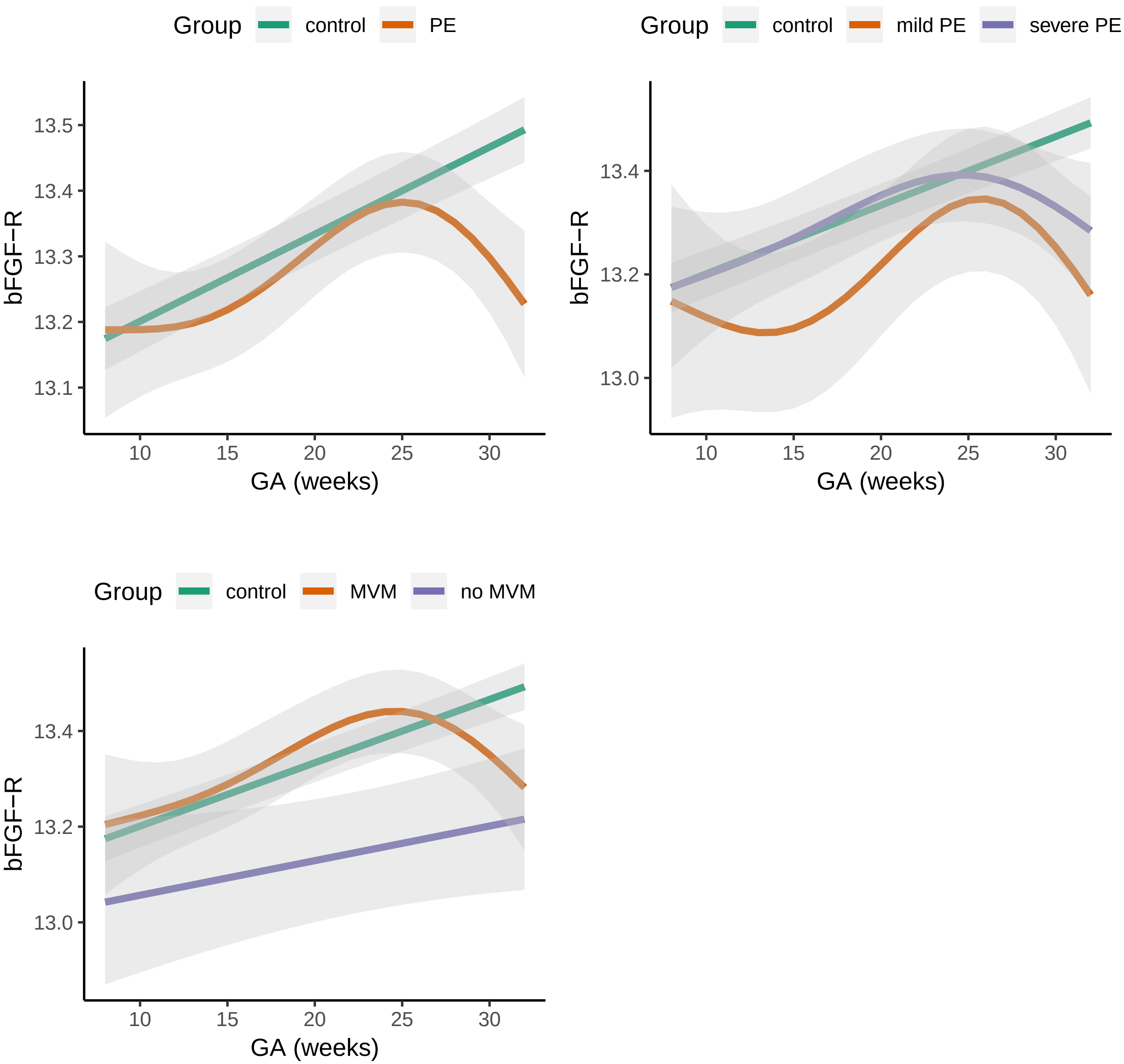
Group
control
PE
bFGF−R
13.5
13.4
13.3
13.2
13.1
10
15
20
25
30
GA (weeks)
Group
control
mild PE
severe PE
bFGF−R
13.4
13.2
13.0
10
15
20
25
30
GA (weeks)
Group
control
MVM
no MVM
bFGF−R
13.4
13.2
13.0
10
15
20
25
30
GA (weeks)

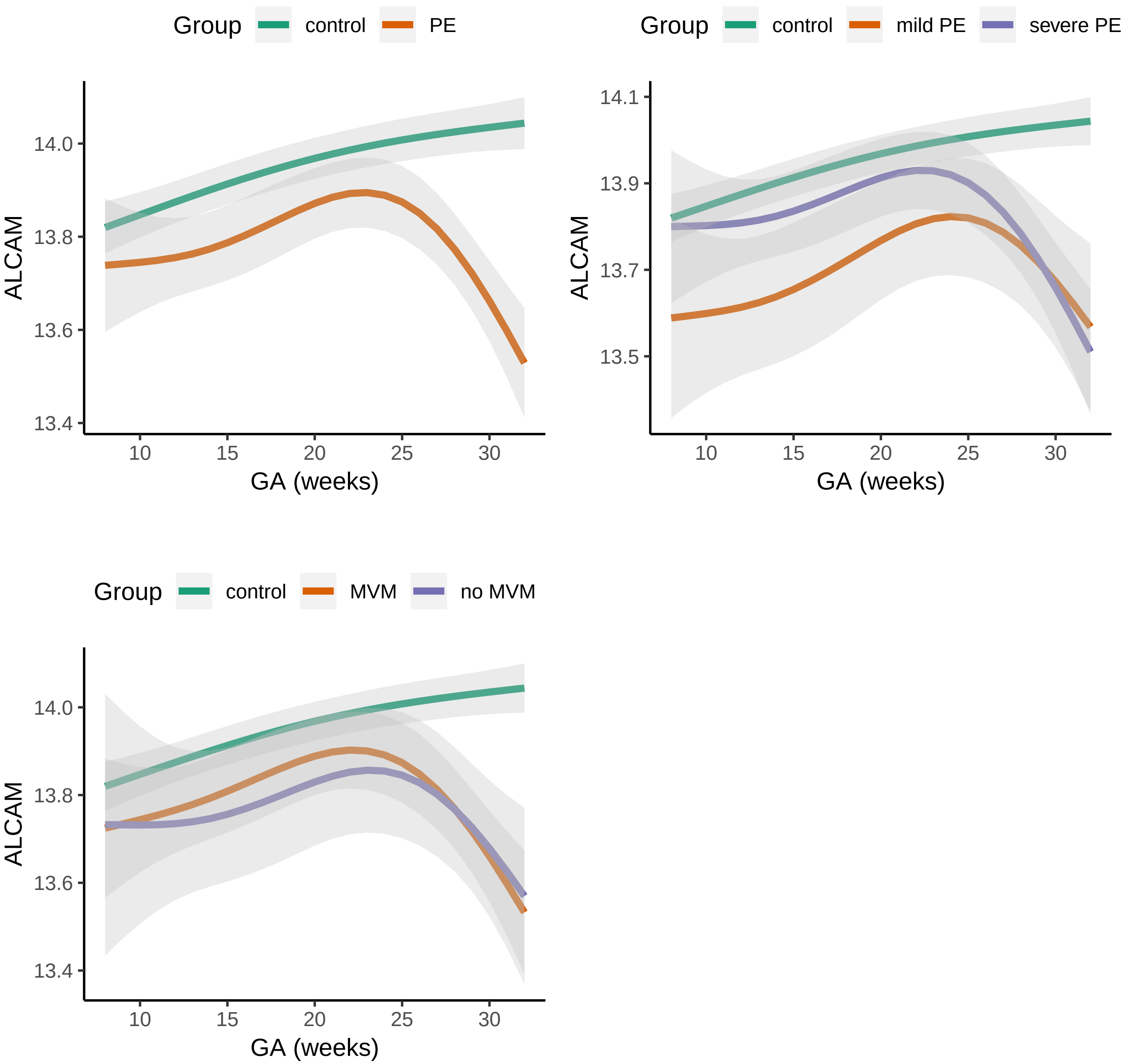

Group
control
PE
ALCAM
14.0
13.8
13.6
13.4
10
15
20
25
30
GA (weeks)
Group
control
mild PE
severe PE
ALCAM
14.1
13.9
13.7
13.5
10
15
20
25
30
GA (weeks)
Group
control
MVM
no MVM
ALCAM
14.0
13.8
13.6
13.4
10
15
20
25
30
GA (weeks)

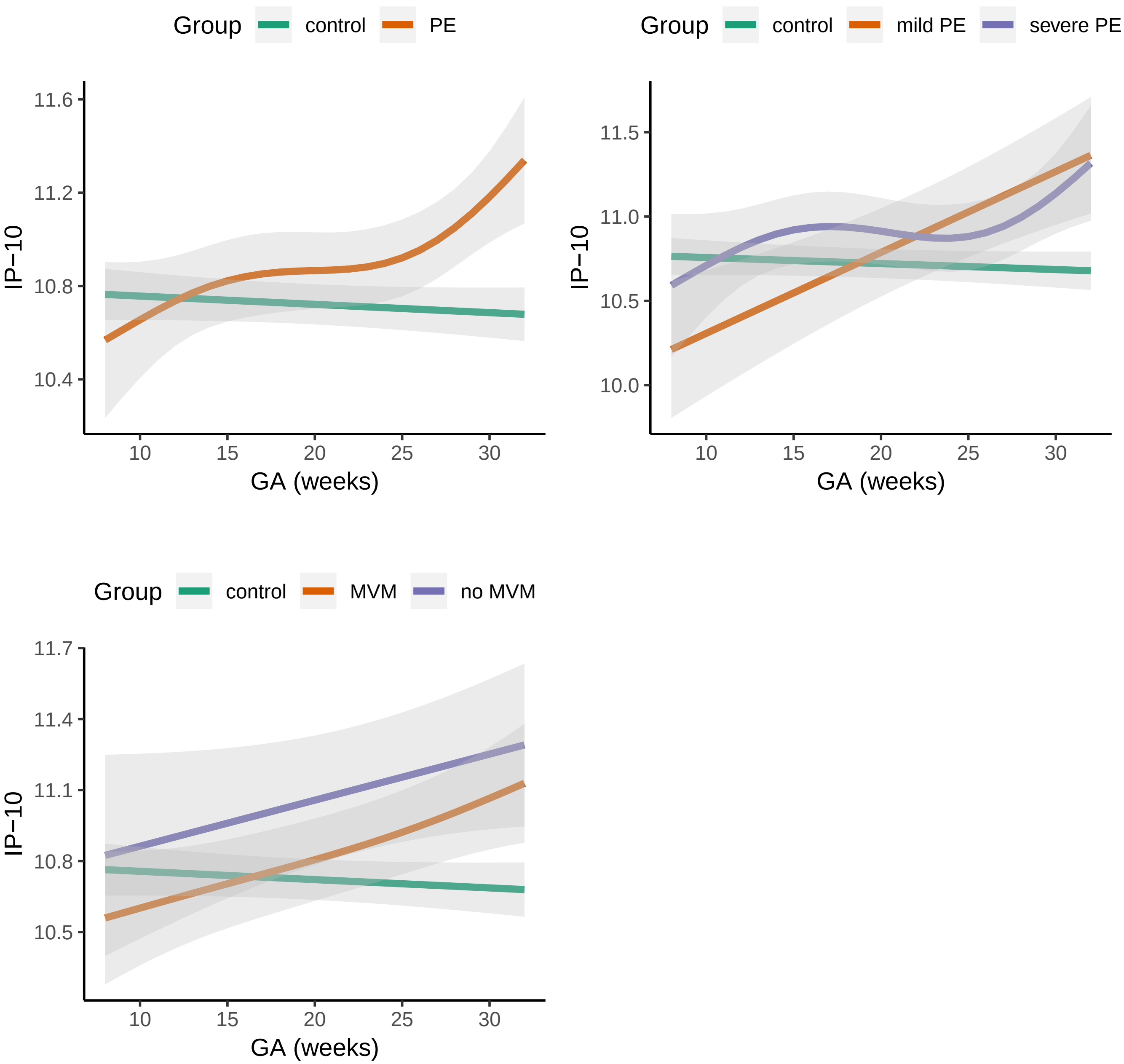
Group
control
PE
IP−10
11.6
11.2
10.8
10.4
10
15
20
25
30
GA (weeks)
Group
control
mild PE
severe PE
IP−10
11.5
11.0
10.5
10.0
10
15
20
25
30
GA (weeks)
Group
control
MVM
no MVM
IP−10
11.7
11.4
11.1
10.8
10.5
10
15
20
25
30
GA (weeks)

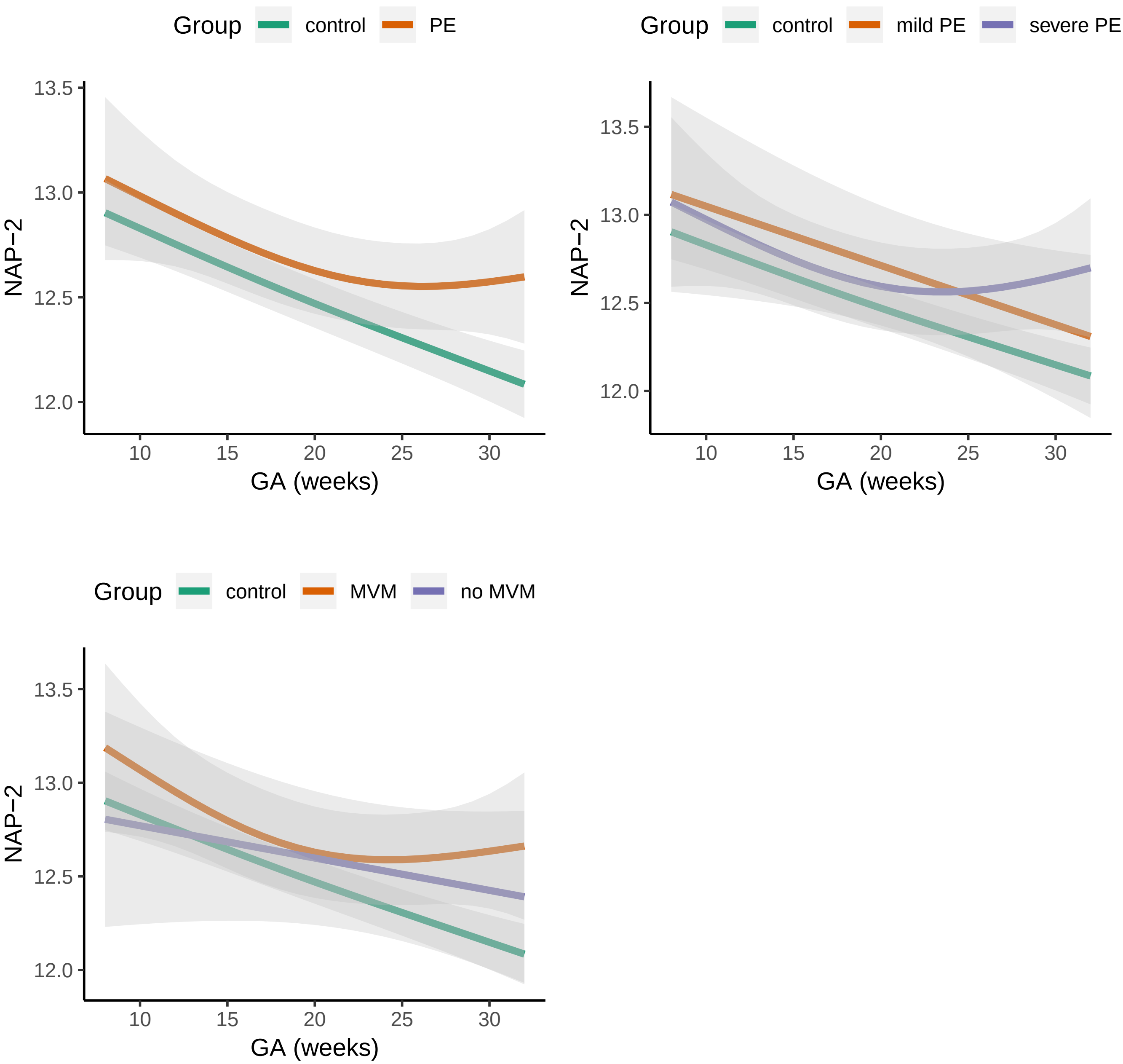

Group
control
PE
NAP−2
GA (weeks)
Group
control
mild PE
severe PE
NAP−2
GA (weeks)
Group
control
MVM
no MVM
NAP−2
GA (weeks)

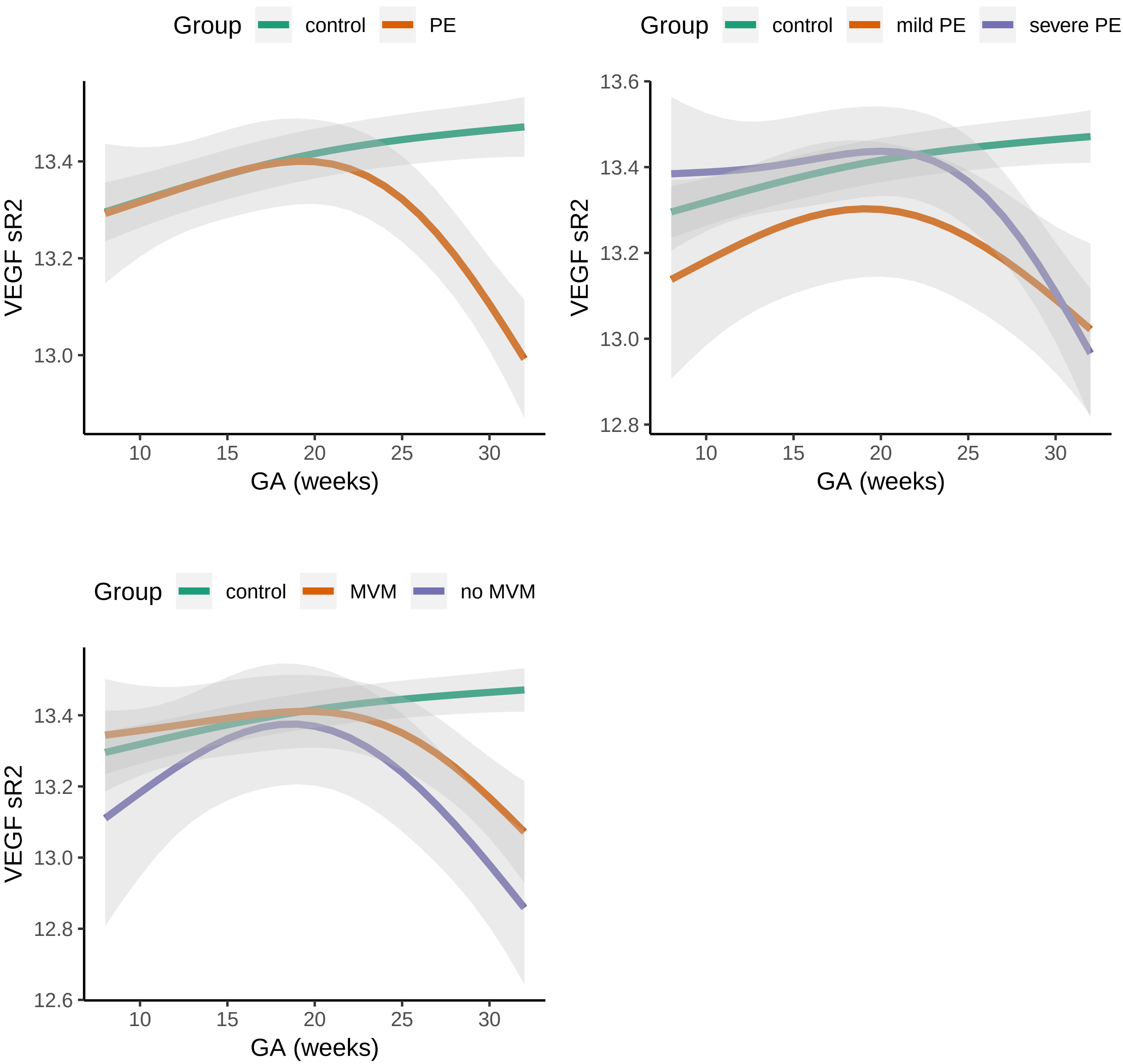

Group
control
PE
VEGF sR2
13.4
13.2
13.0
10
15
20
25
30
GA (weeks)
Group
control
mild PE
severe PE
VEGF sR2
13.6
13.4
13.2
13.0
12.8
10
15
20
25
30
GA (weeks)
Group
control
MVM
no MVM
VEGF sR2
13.4
13.2
13.0
12.8
12.6
10
15
20
25
30
GA (weeks)

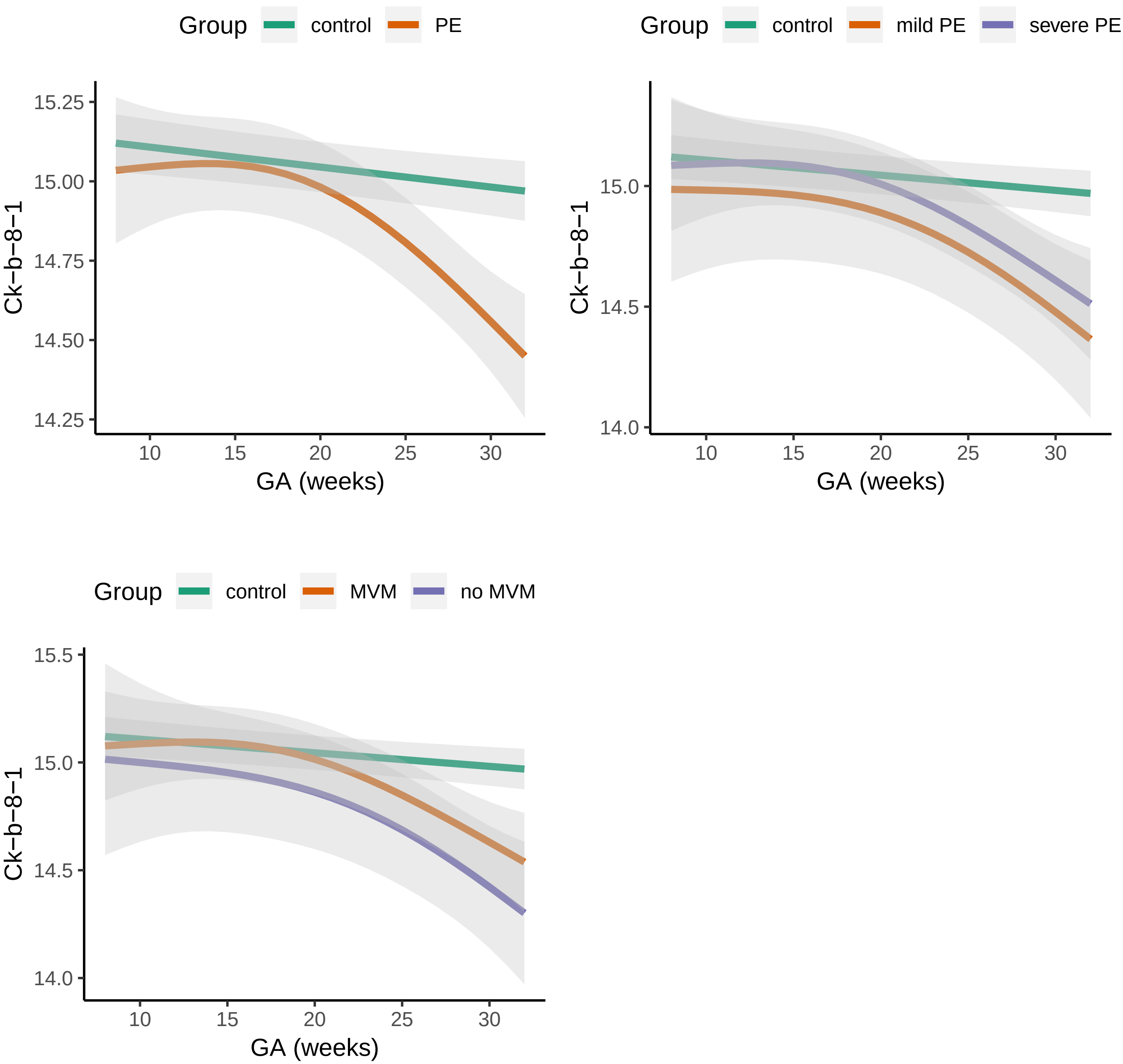

Group
control
PE
15.25
15.00
14.75
14.50
14.25
Ck−b−8−1
10
15
20
25
30
GA (weeks)
Group
control
mild PE
severe PE
15.0
14.5
14.0
Ck−b−8−1
10
15
20
25
30
GA (weeks)
Group
control
MVM
no MVM
15.5
15.0
14.5
14.0
Ck−b−8−1
10
15
20
25
30
GA (weeks)

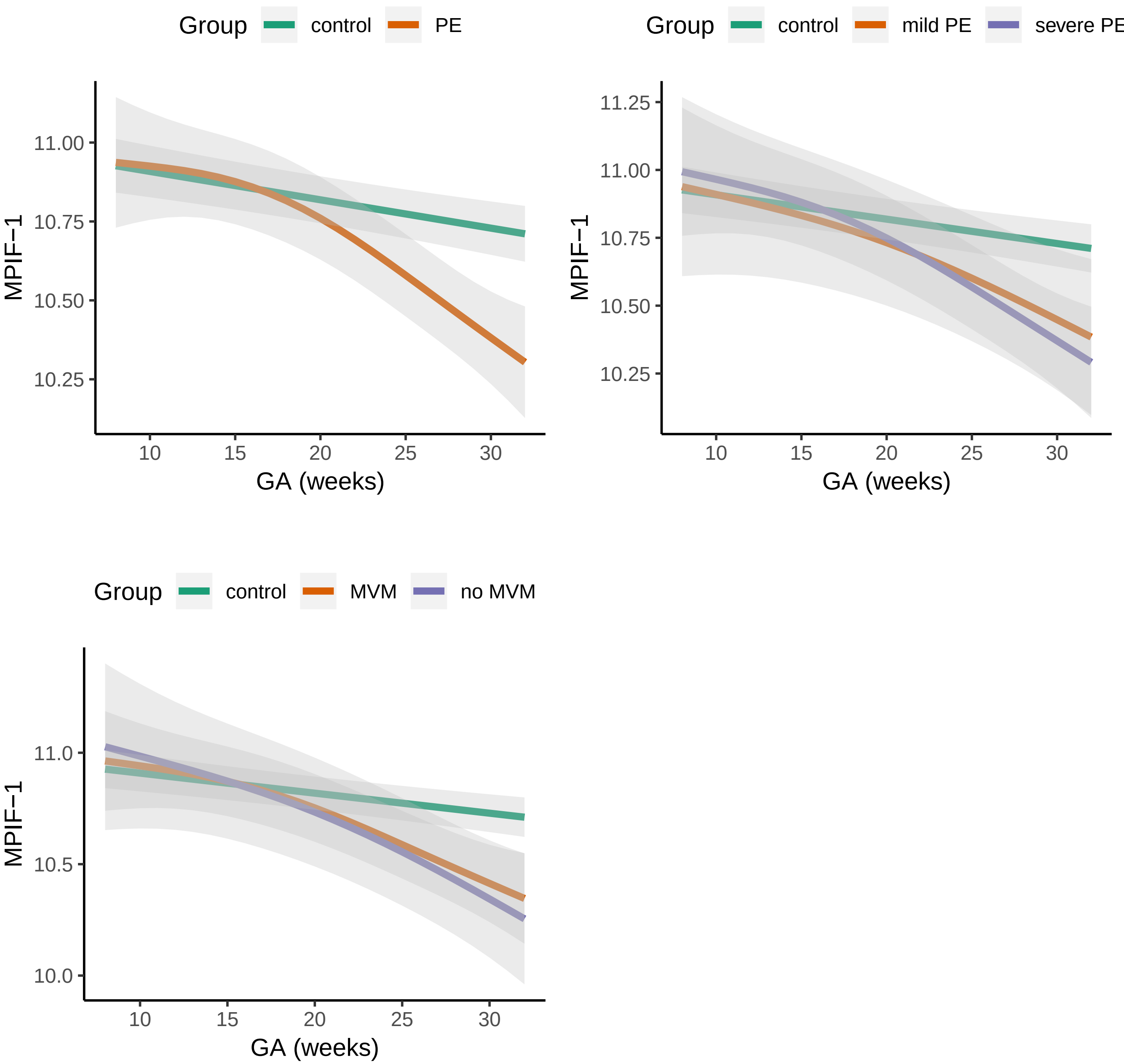

Group
control
PE
MPIF−1
11.00
10.75
10.50
10.25
10
15
20
25
30
GA (weeks)
Group
control
mild PE
severe PE
MPIF−1
11.25
11.00
10.75
10.50
10.25
10
15
20
25
30
GA (weeks)
Group
control
MVM
no MVM
MPIF−1
11.0
10.5
10.0
10
15
20
25
30
GA (weeks)

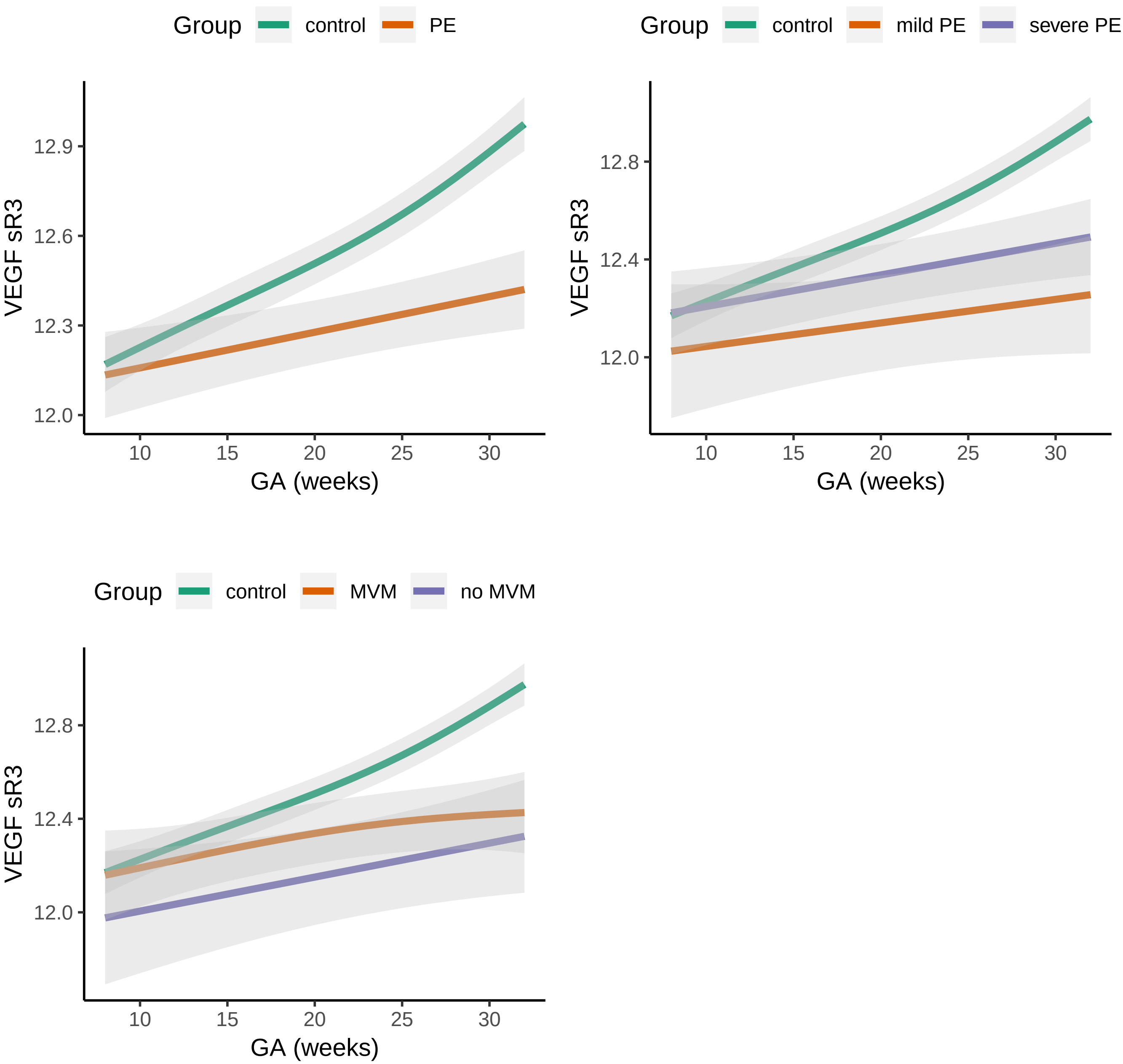

Group
control
PE
VEGF sR3
12.9
12.6
12.3
12.0
10
15
20
25
30
GA (weeks)
Group
control
mild PE
severe PE
VEGF sR3
12.8
12.4
12.0
10
15
20
25
30
GA (weeks)
Group
control
MVM
no MVM
VEGF sR3
12.8
12.4
12.0
10
15
20
25
30
GA (weeks)

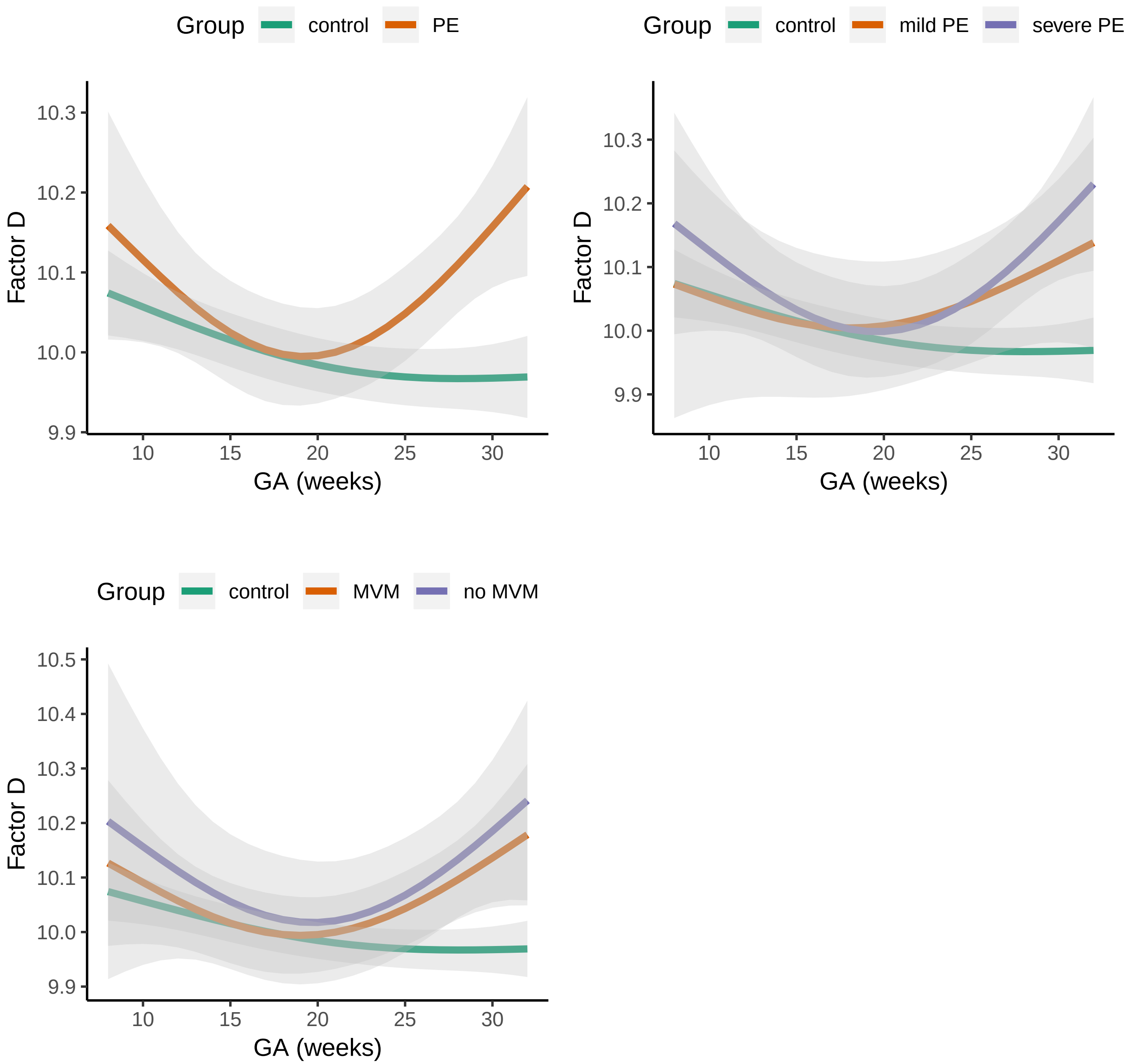

Group
control
PE
Factor D
10.3
10.2
10.1
10.0
9.9
10
15
20
25
30
GA (weeks)
Group
control
mild PE
severe PE
Factor D
10.3
10.2
10.1
10.0
9.9
10
15
20
25
30
GA (weeks)
Group
control
MVM
no MVM
Factor D
10.5
10.4
10.3
10.2
10.1
10.0
9.9
10
15
20
25
30
GA (weeks)

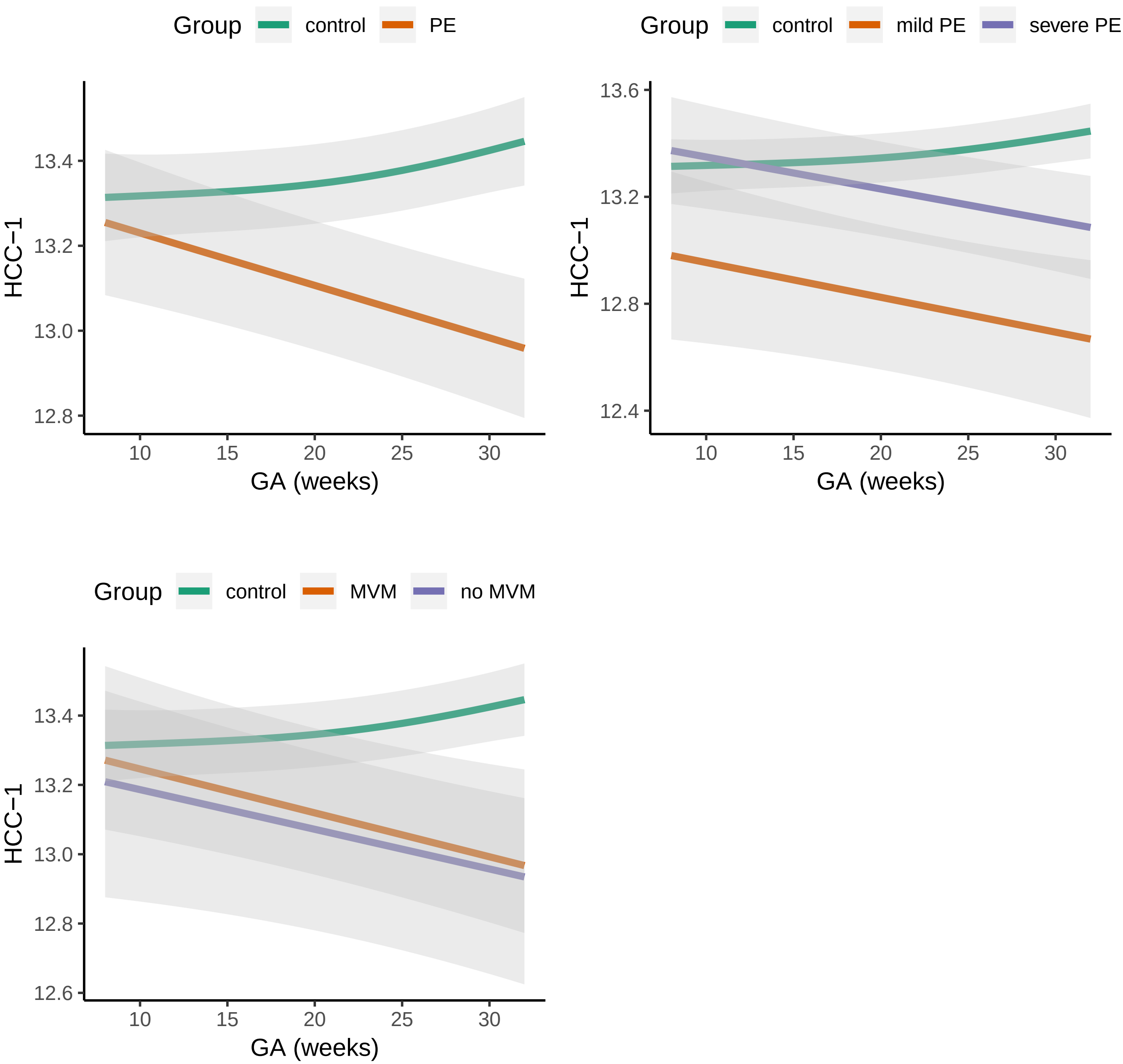

Group
control
PE
HCC−1
13.4
13.2
13.0
12.8
10
15
20
25
30
GA (weeks)
Group
control
mild PE
severe PE
HCC−1
13.6
13.2
12.8
12.4
10
15
20
25
30
GA (weeks)
Group
control
MVM
no MVM
HCC−1
13.4
13.2
13.0
12.8
12.6
10
15
20
25
30
GA (weeks)

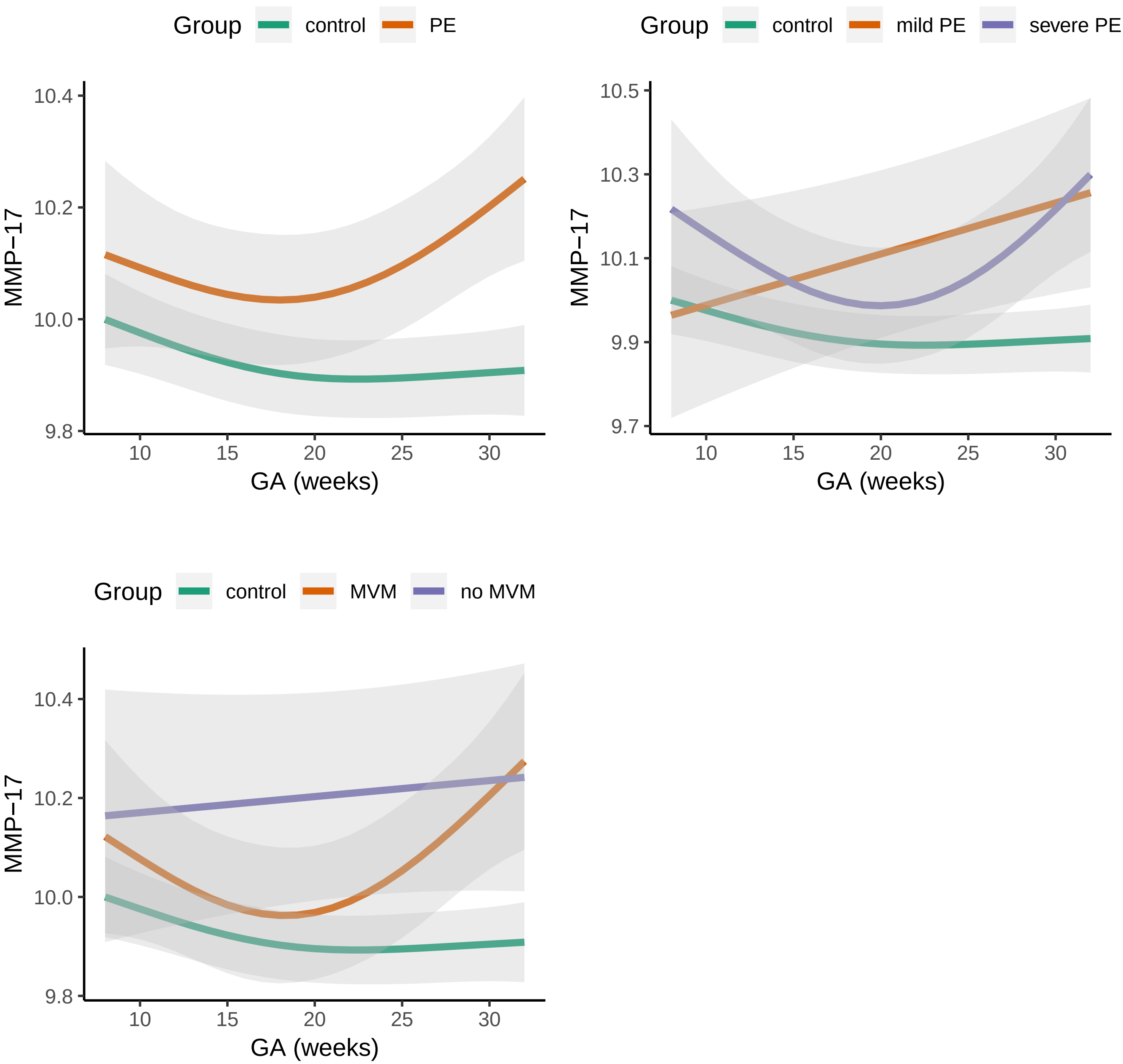

Group
control
PE
MMP−17
10.4
10.2
10.0
9.8
10
15
20
25
30
GA (weeks)
Group
control
mild PE
severe PE
MMP−17
10.5
10.3
10.1
9.9
9.7
10
15
20
25
30
GA (weeks)
Group
control
MVM
no MVM
MMP−17
10.4
10.2
10.0
9.8
10
15
20
25
30
GA (weeks)

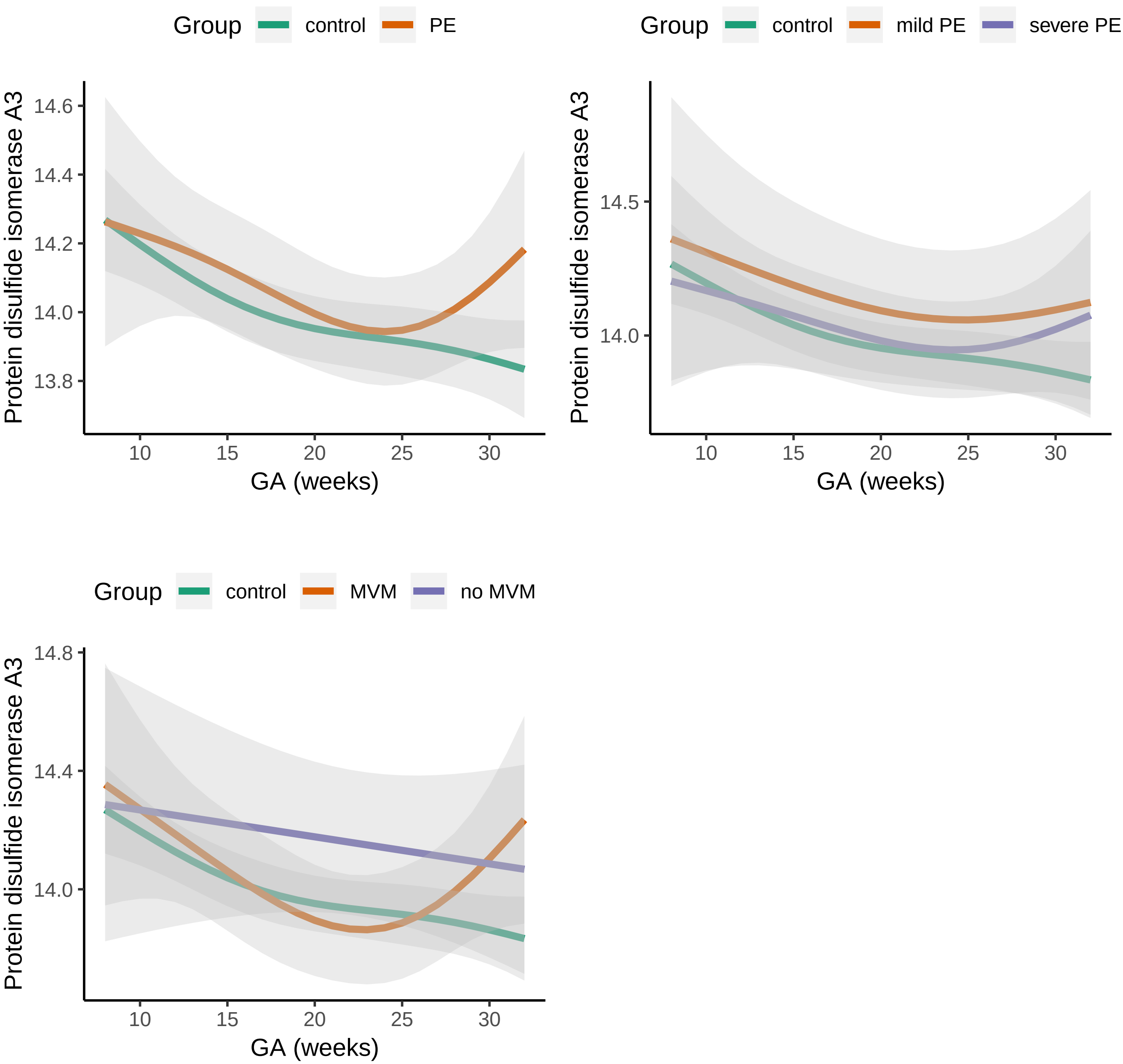
Group
control
PE
Protein disulfide isomerase A3
14.6
14.4
14.2
14.0
13.8
10
15
20
25
30
GA (weeks)
Group
control
mild PE
severe PE
Protein disulfide isomerase A3
14.5
14.0
10
15
20
25
30
GA (weeks)
Group
control
MVM
no MVM
Protein disulfide isomerase A3
14.8
14.4
14.0
10
15
20
25
30
GA (weeks)

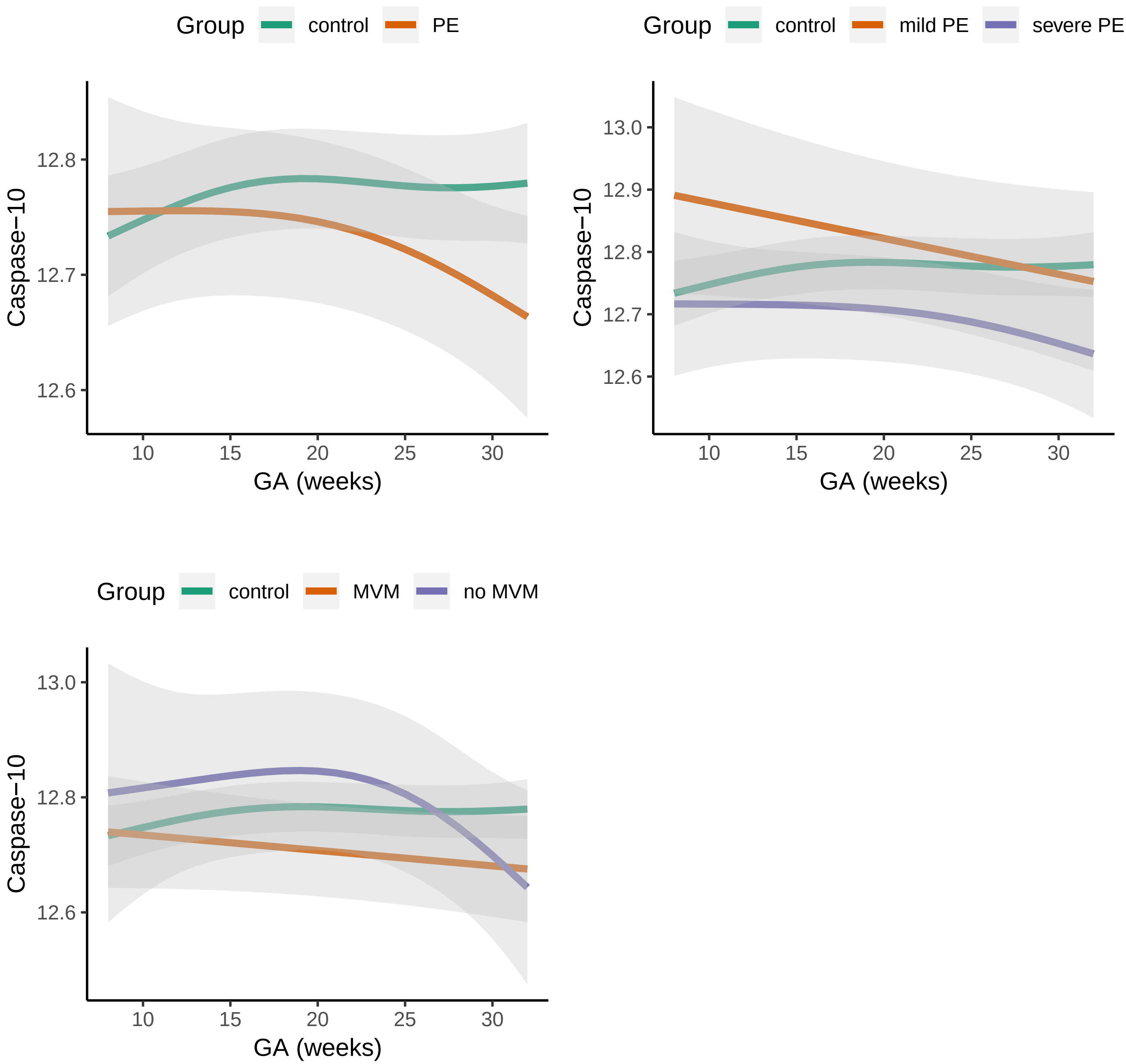

Group
control
PE
Caspase−10
12.8
12.7
12.6
10
15
20
25
30
GA (weeks)
Group
control
mild PE
severe PE
Caspase−10
13.0
12.9
12.8
12.7
12.6
10
15
20
25
30
GA (weeks)
Group
control
MVM
no MVM
Caspase−10
13.0
12.8
12.6
10
15
20
25
30
GA (weeks)

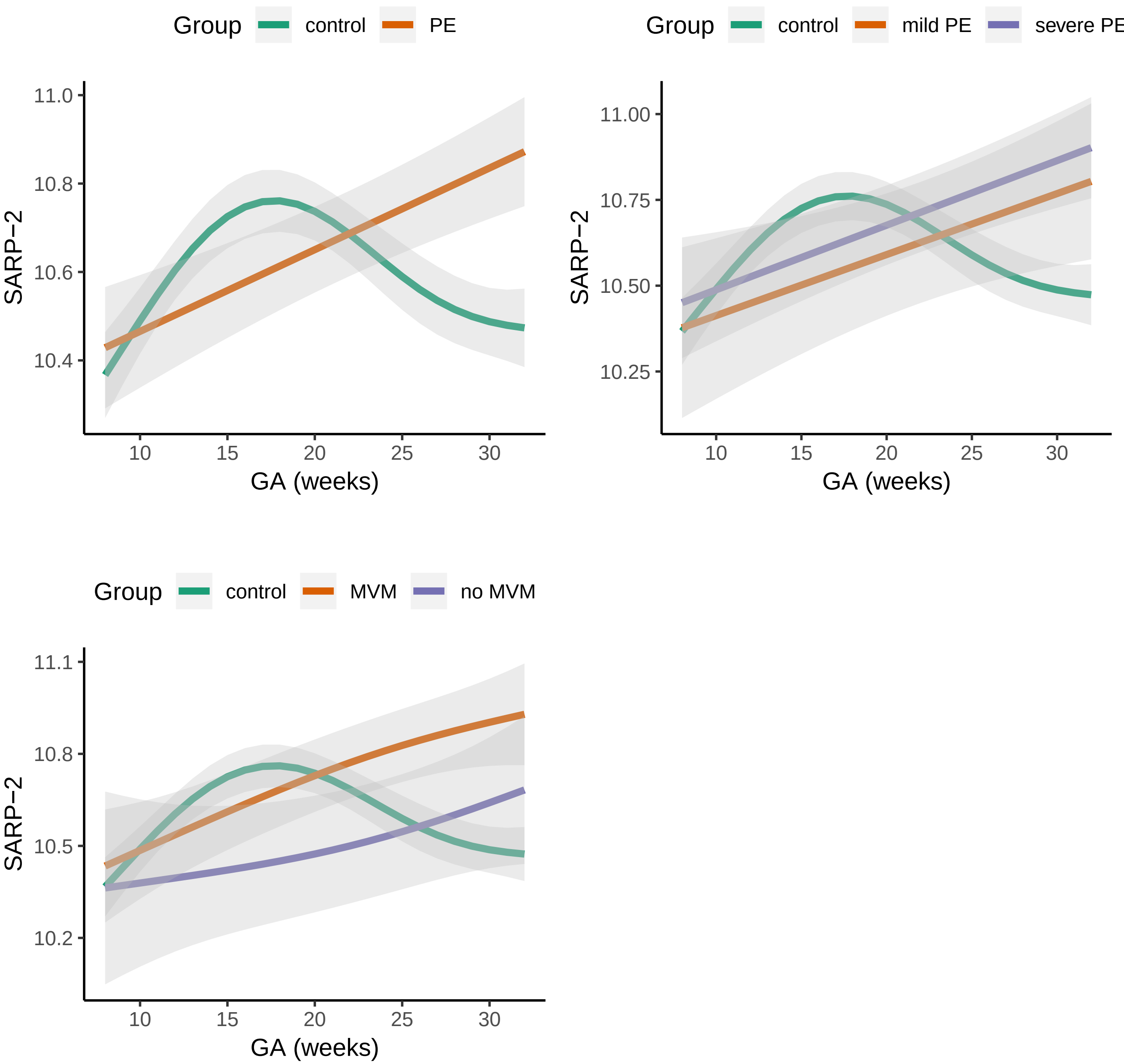
Group
control
PE
SARP−2
11.0
10.8
10.6
10.4
10
15
20
25
30
GA (weeks)
Group
control
mild PE
severe PE
SARP−2
11.00
10.75
10.50
10.25
10
15
20
25
30
GA (weeks)
Group
control
MVM
no MVM
SARP−2
11.1
10.8
10.5
10.2
10
15
20
25
30
GA (weeks)

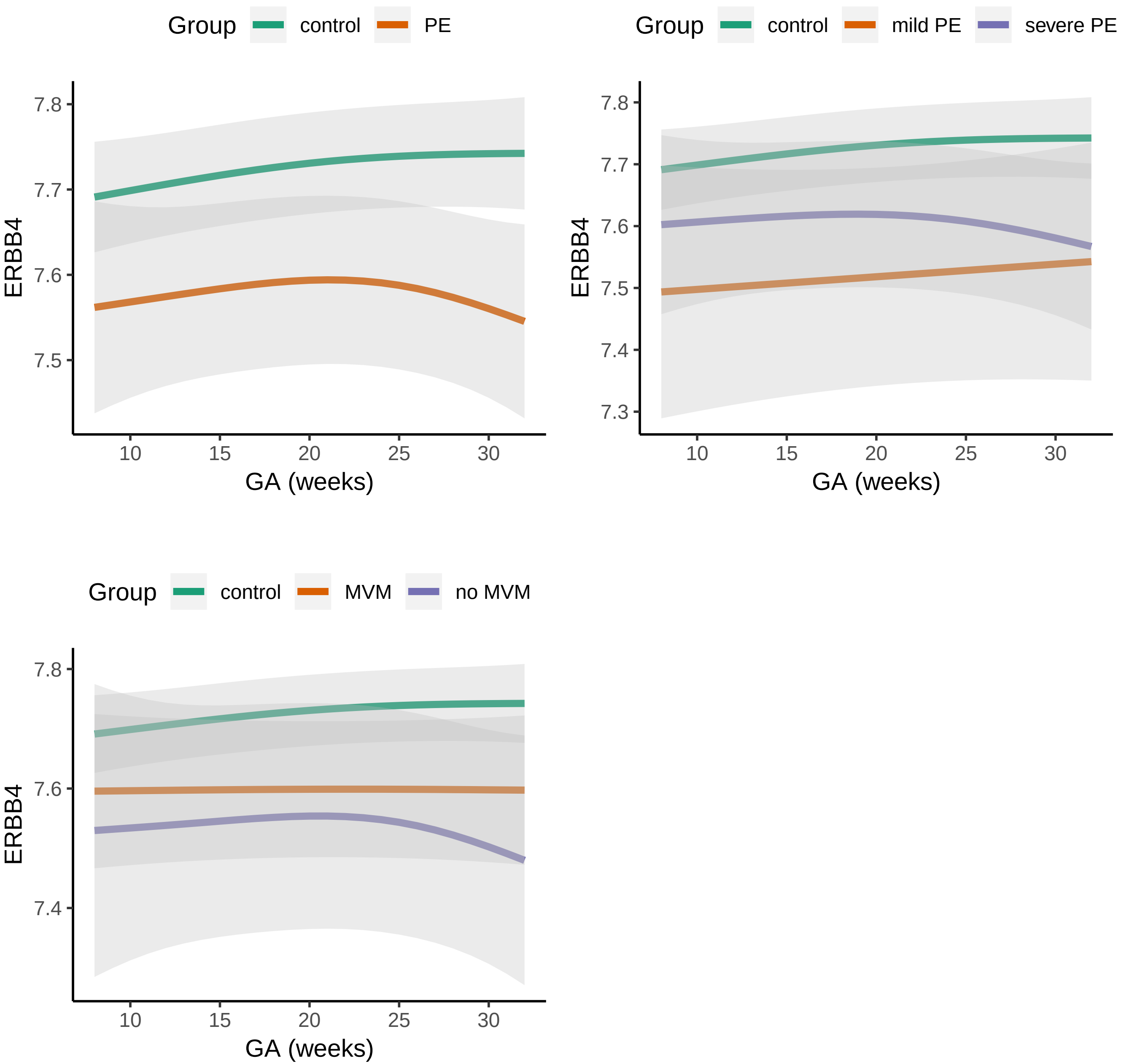

Group
control
PE
ERBB4
7.8
7.7
7.6
7.5
10
15
20
25
30
GA (weeks)
Group
control
mild PE
severe PE
ERBB4
7.8
7.7
7.6
7.5
7.4
7.3
10
15
20
25
30
GA (weeks)
Group
control
MVM
no MVM
ERBB4
7.8
7.6
7.4
10
15
20
25
30
GA (weeks)

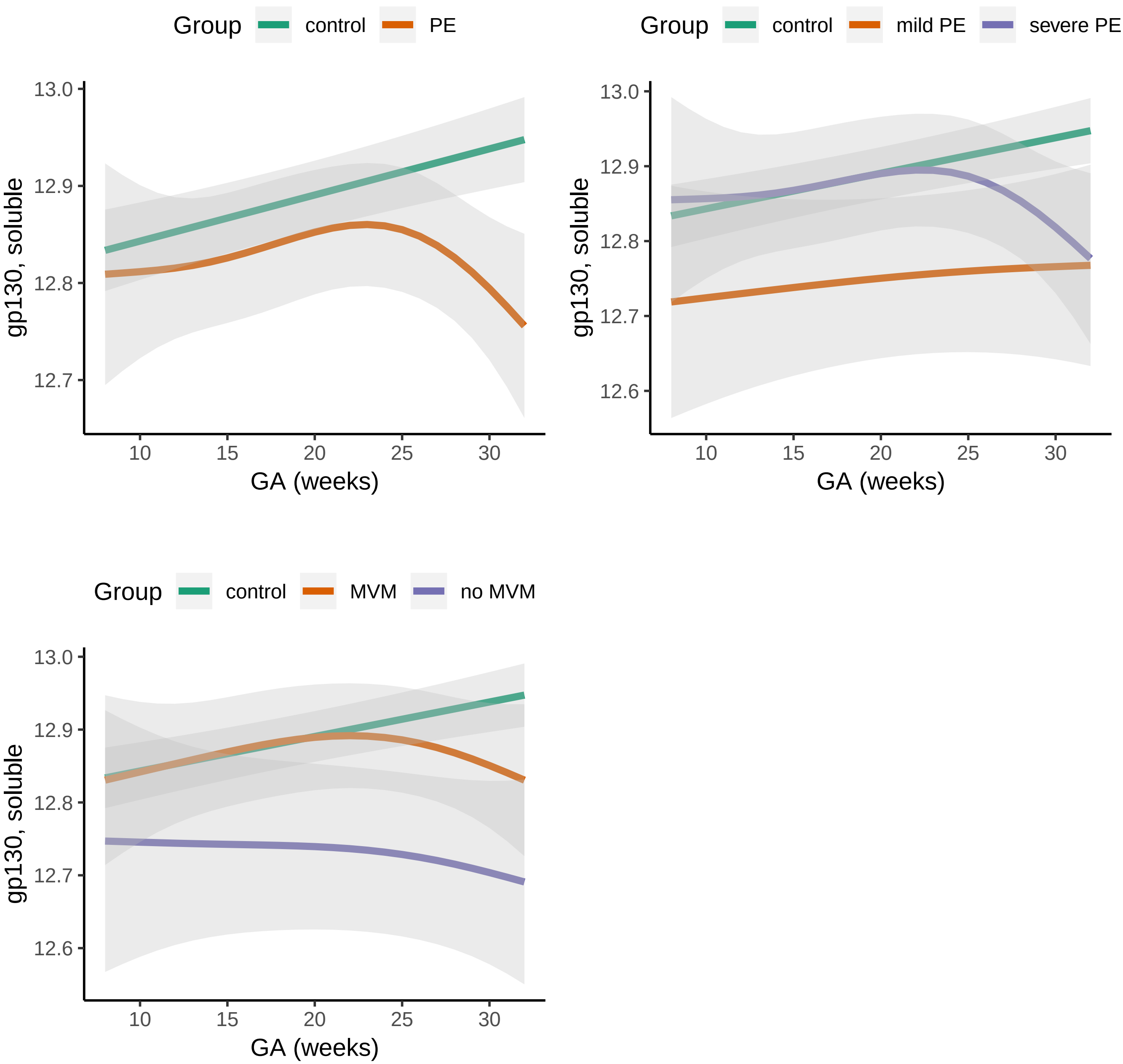

Group
control
PE
13.0
12.9
12.8
12.7
gp130, soluble
10
15
20
25
30
GA (weeks)
Group
control
mild PE
severe PE
13.0
12.9
12.8
12.7
12.6
gp130, soluble
10
15
20
25
30
GA (weeks)
Group
control
MVM
no MVM
13.0
12.9
12.8
12.7
12.6
gp130, soluble
10
15
20
25
30
GA (weeks)

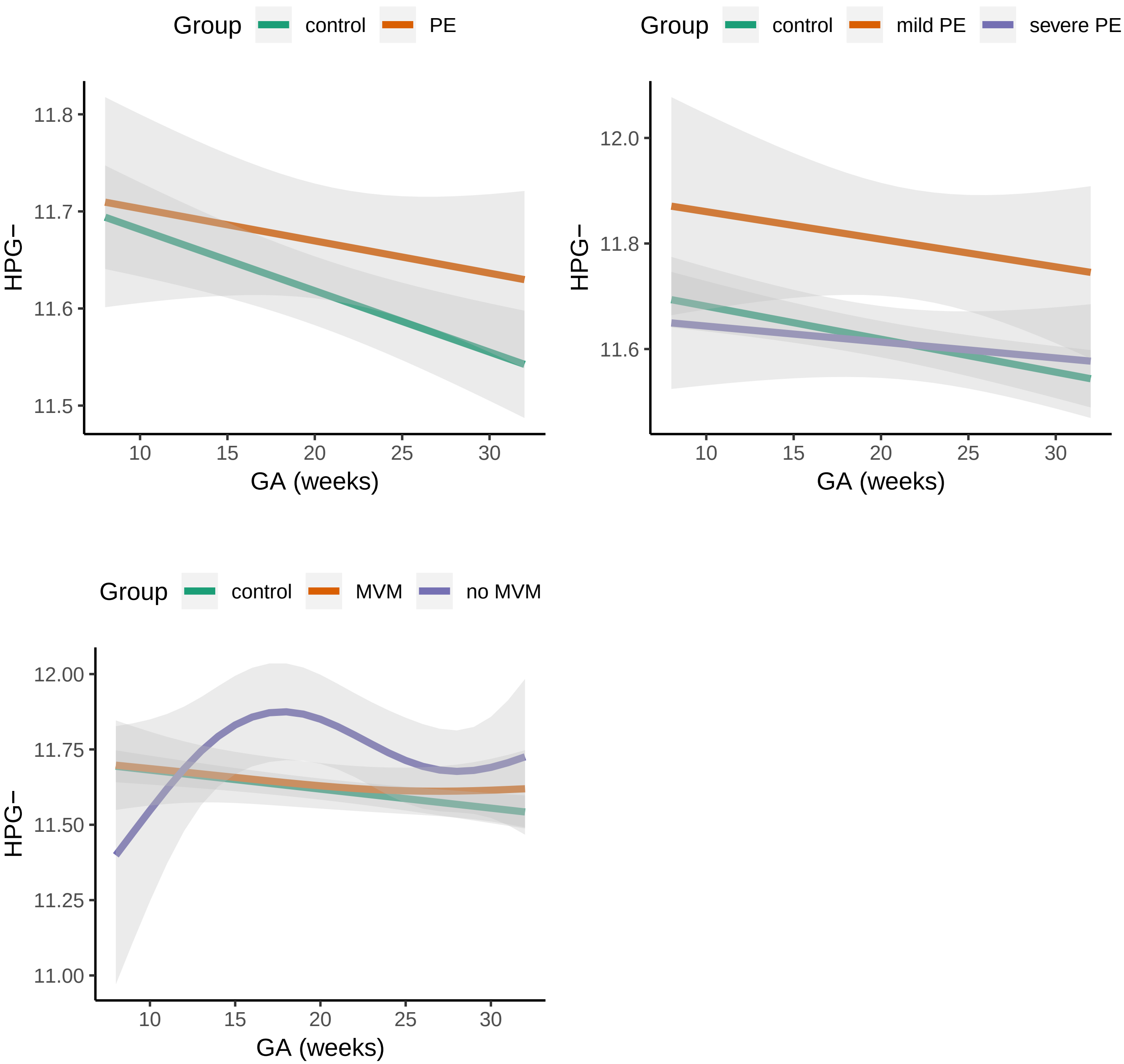

Group
control
PE
11.8
11.7
11.6
11.5
HPG−
10
15
20
25
30
GA (weeks)
Group
control
mild PE
severe PE
12.0
11.8
11.6
HPG−
10
15
20
25
30
GA (weeks)
Group
control
MVM
no MVM
12.00
11.75
11.50
11.25
11.00
HPG−
10
15
20
25
30
GA (weeks)

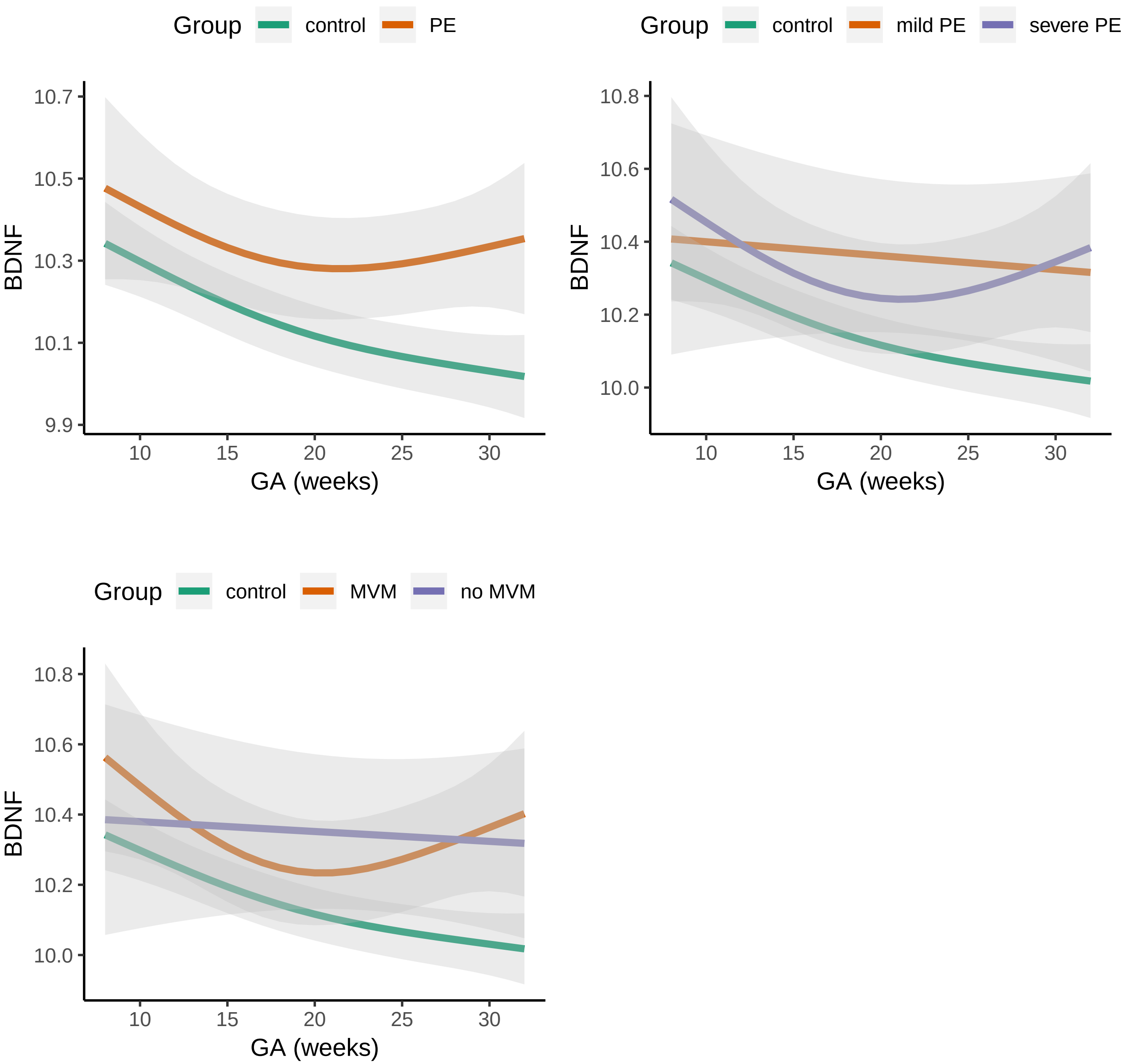
Group
control
PE
10.7
10.5
10.3
10.1
9.9
BDNF
10
15
20
25
30
GA (weeks)
Group
control
mild PE
severe PE
10.8
10.6
10.4
10.2
10.0
BDNF
10
15
20
25
30
GA (weeks)
Group
control
MVM
no MVM
10.8
10.6
10.4
10.2
10.0
BDNF
10
15
20
25
30
GA (weeks)

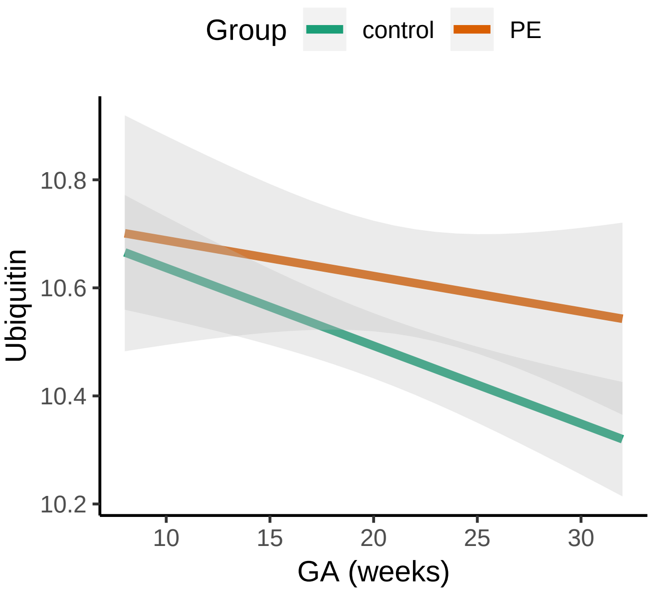
Group
control
PE
10.8
10.6
10.4
10.2
Ubiquitin
10
15
20
25
30
GA (weeks)

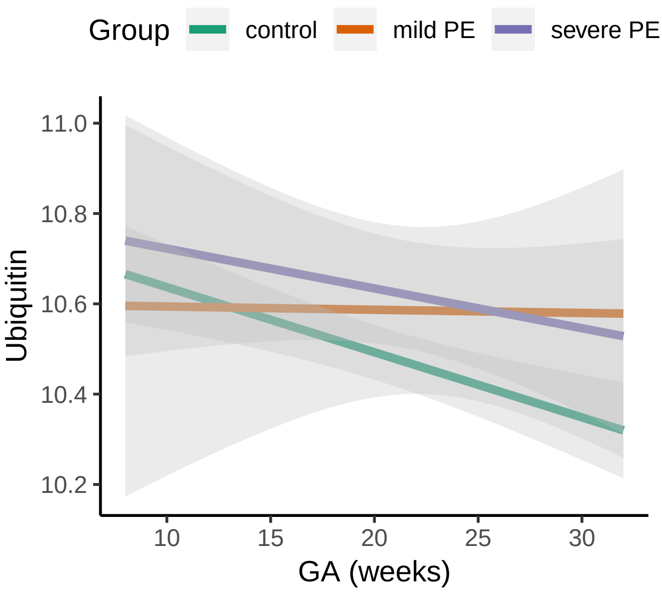
Group
control
mild PE
severe PE
11.0
10.8
10.6
10.4
10.2
Ubiquitin
10
15
20
25
30
GA (weeks)

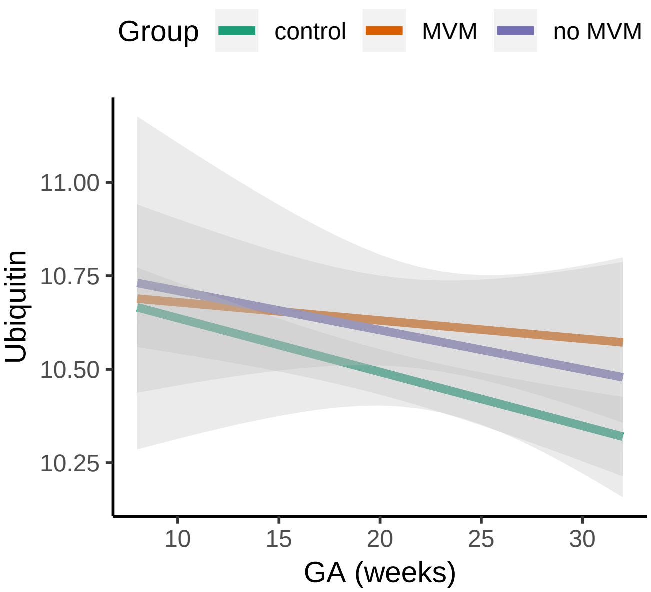
Group
control
MVM
no MVM
11.00
10.75
10.50
10.25
Ubiquitin
10
15
20
25
30
GA (weeks)

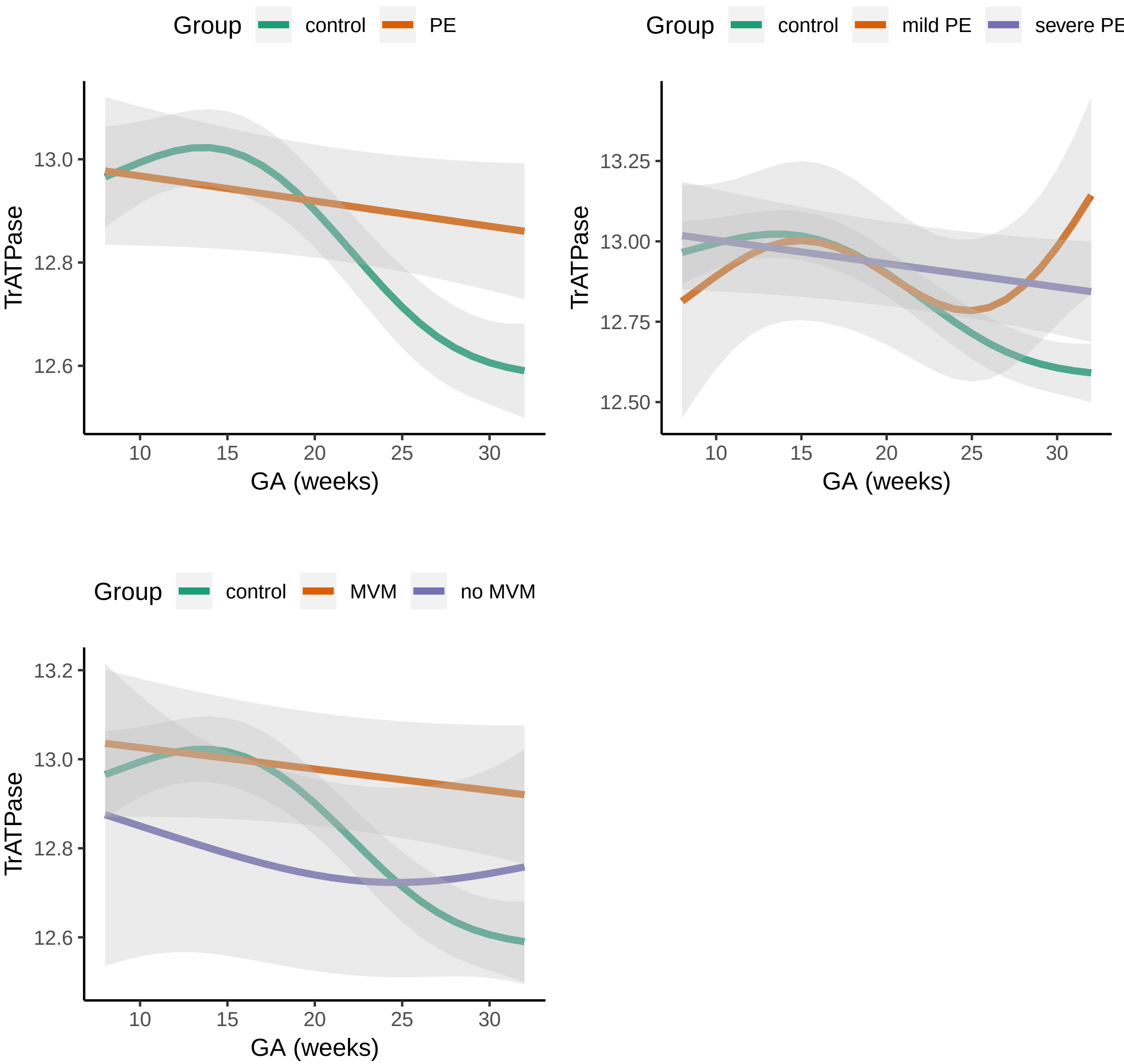

Group
control
PE
TrATPase
13.0
12.8
12.6
10
15
20
25
30
GA (weeks)
Group
control
mild PE
severe PE
TrATPase
13.25
13.00
12.75
12.50
10
15
20
25
30
GA (weeks)
Group
control
MVM
no MVM
TrATPase
13.2
13.0
12.8
12.6
10
15
20
25
30
GA (weeks)

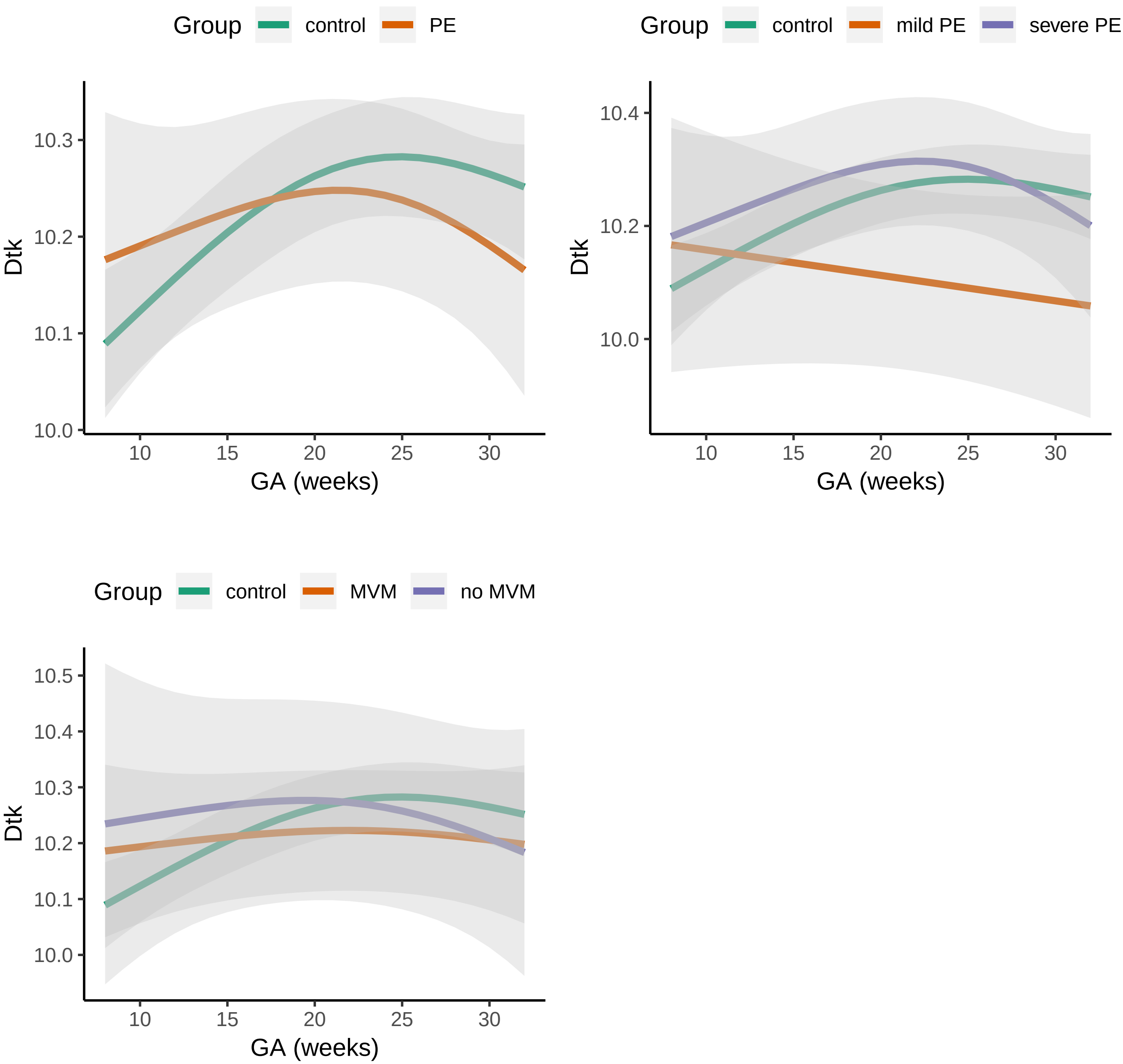

Group
control
PE
10.3
10.2
10.1
10.0
Dtk
10
15
20
25
30
GA (weeks)
Group
control
mild PE
severe PE
10.4
10.2
10.0
Dtk
10
15
20
25
30
GA (weeks)
Group
control
MVM
no MVM
10.5
10.4
10.3
10.2
10.1
10.0
Dtk
10
15
20
25
30
GA (weeks)

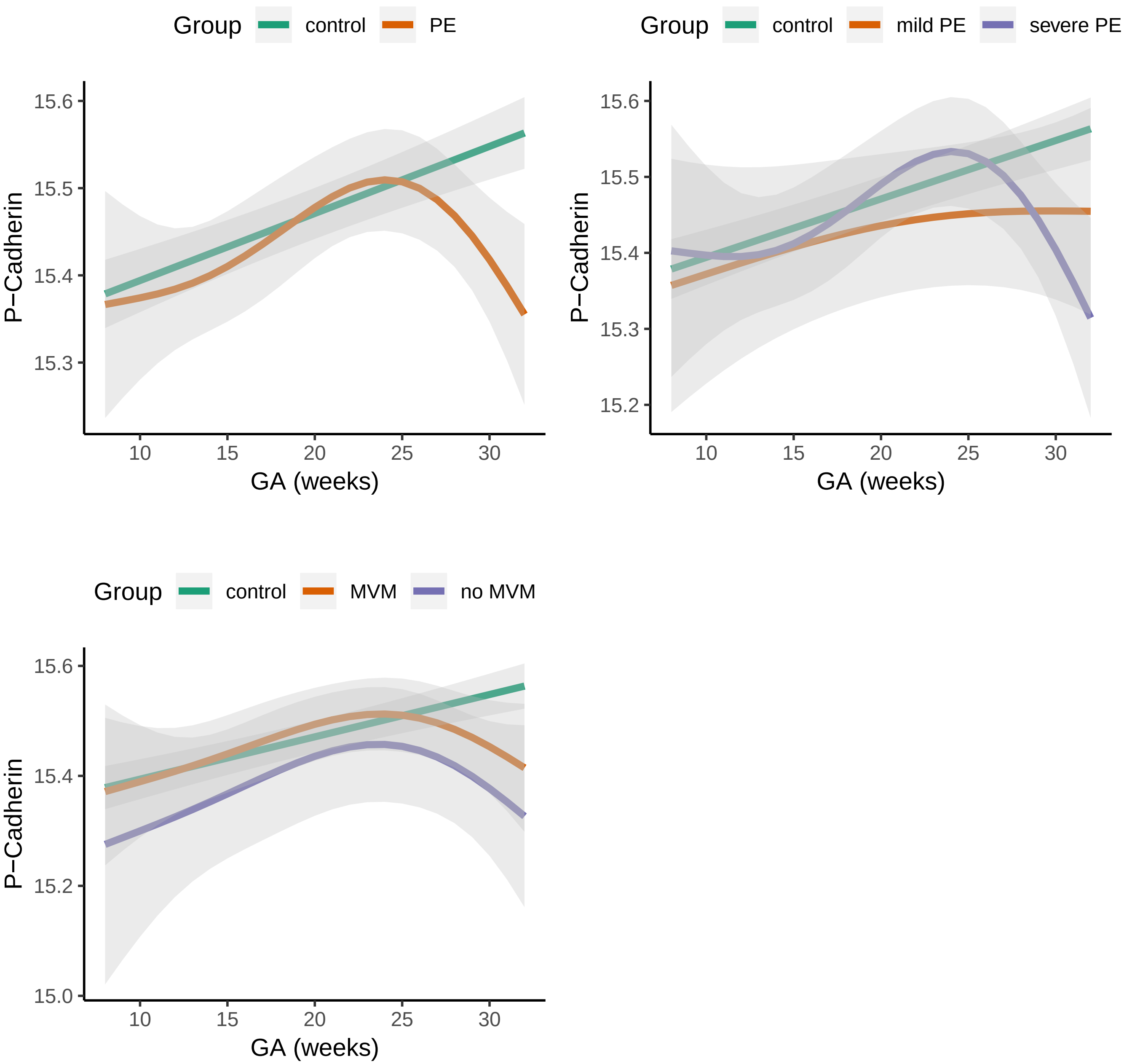
Group
control
PE
P−Cadherin
GA (weeks)
Group
control
mild PE
severe PE
P−Cadherin
GA (weeks)
Group
control
MVM
no MVM
P−Cadherin
GA (weeks)

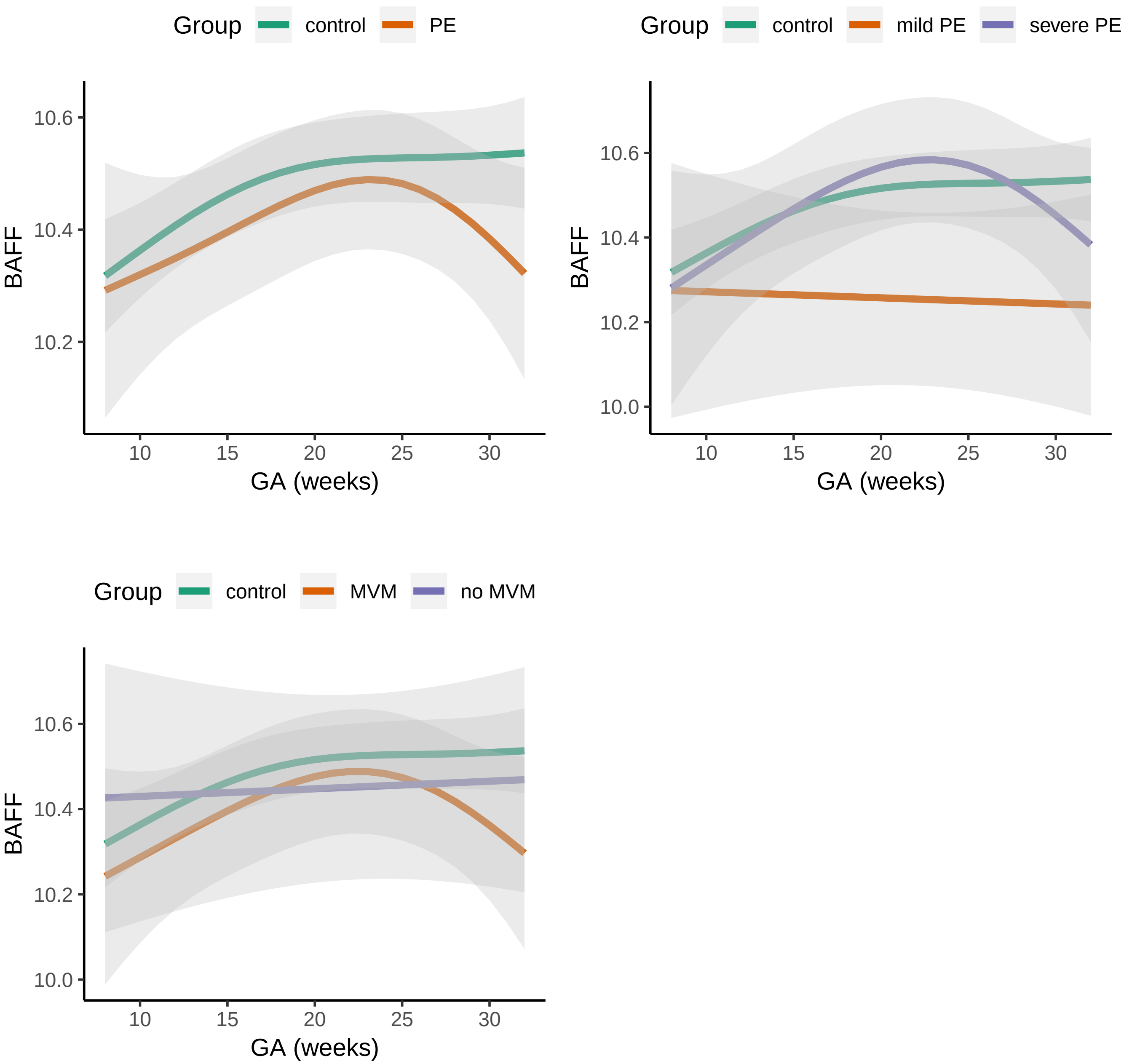

Group
control
PE
10.6
10.4
10.2
BAFF
10
15
20
25
30
GA (weeks)
Group
control
mild PE
severe PE
10.6
10.4
10.2
10.0
BAFF
10
15
20
25
30
GA (weeks)
Group
control
MVM
no MVM
10.6
10.4
10.2
10.0
BAFF
10
15
20
25
30
GA (weeks)

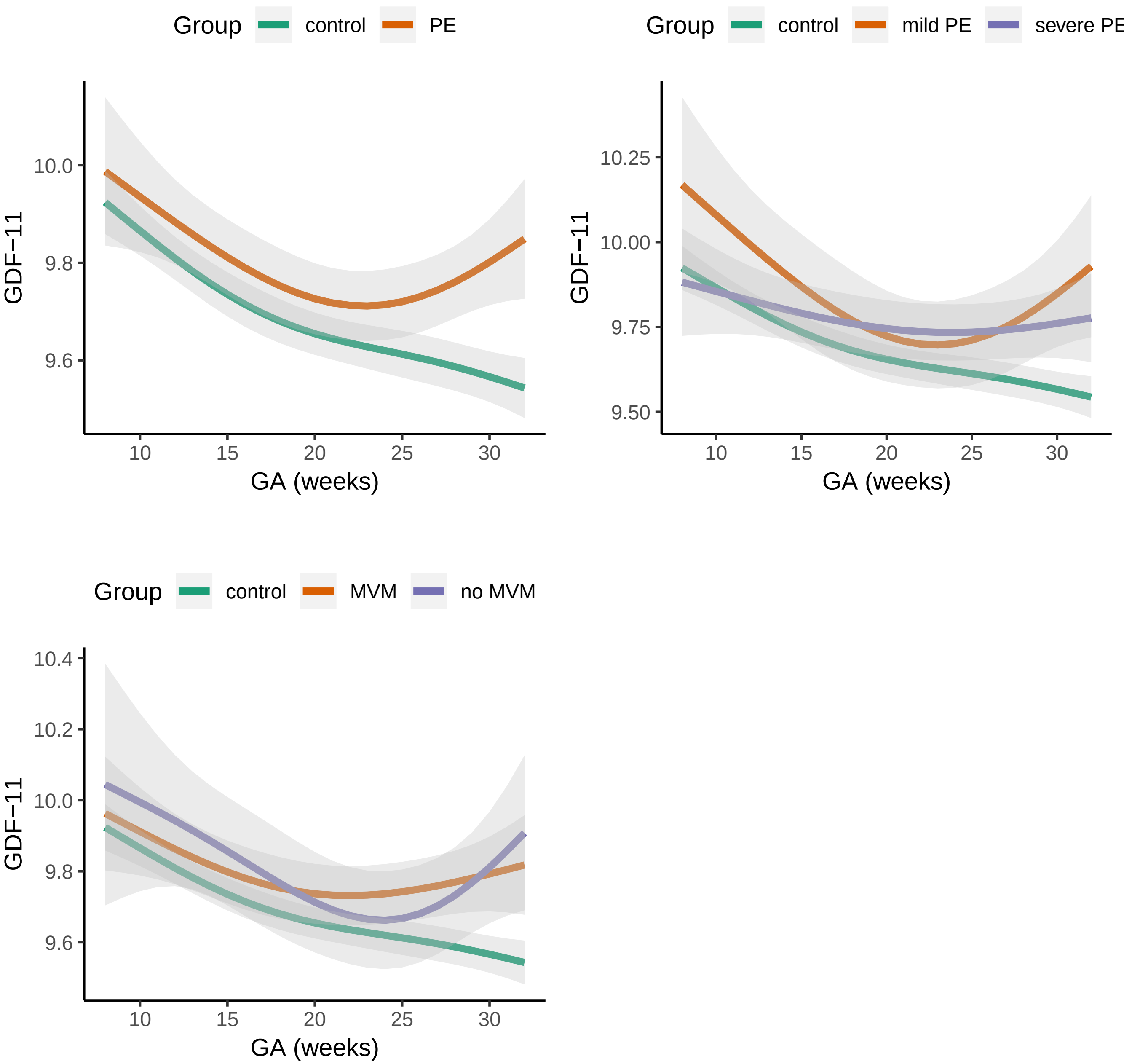
Group
control
PE
GDF−11
10.0
9.8
9.6
10
15
20
25
30
GA (weeks)
Group
control
mild PE
severe PE
GDF−11
10.25
10.00
9.75
9.50
10
15
20
25
30
GA (weeks)
Group
control
MVM
no MVM
GDF−11
10.4
10.2
10.0
9.8
9.6
10
15
20
25
30
GA (weeks)

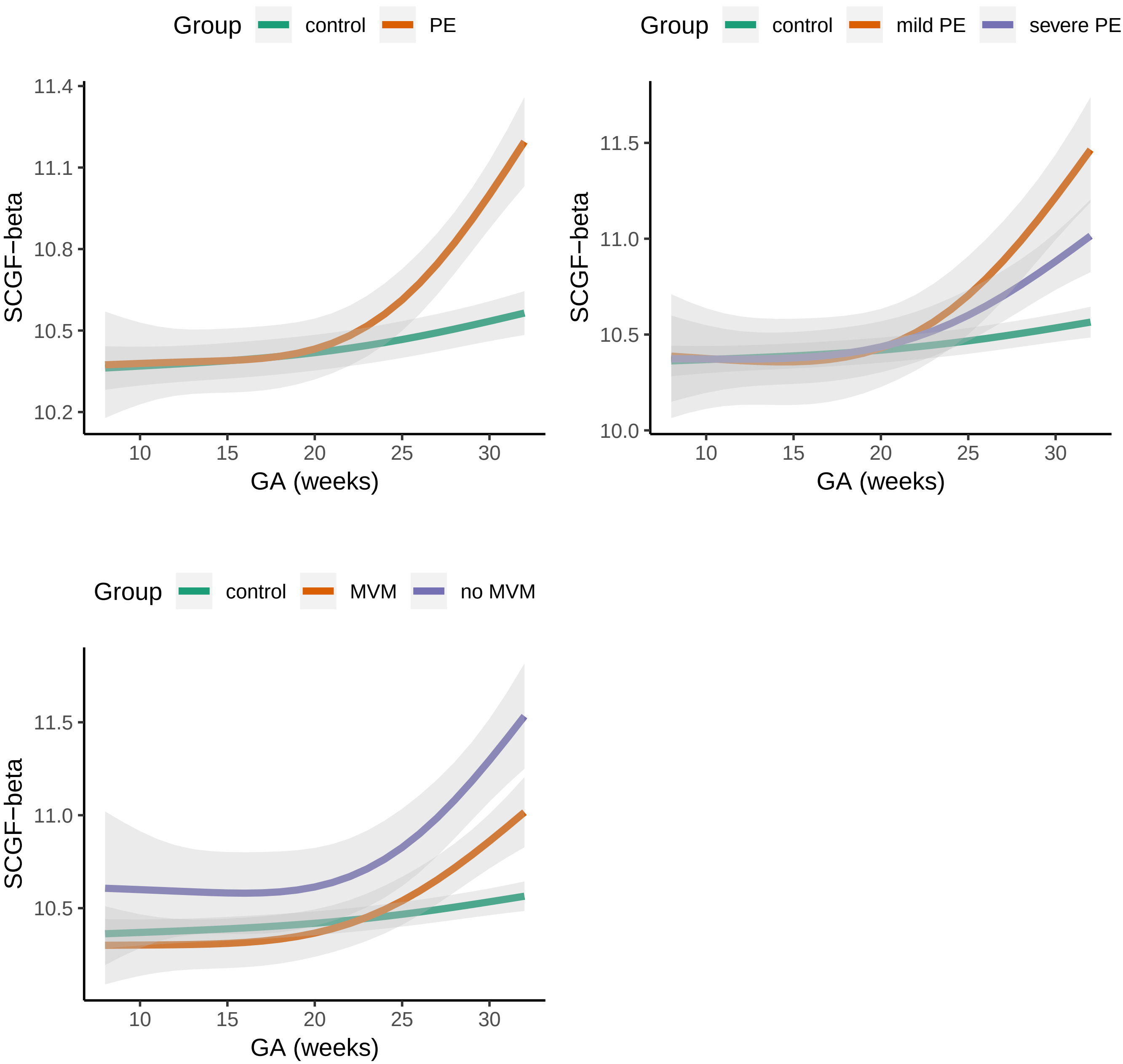
Group
control
PE
SCGF−beta
11.4
11.1
10.8
10.5
10.2
10
15
20
25
30
GA (weeks)
Group
control
mild PE
severe PE
SCGF−beta
11.5
11.0
10.5
10.0
10
15
20
25
30
GA (weeks)
Group
control
MVM
no MVM
SCGF−beta
11.5
11.0
10.5
10
15
20
25
30
GA (weeks)

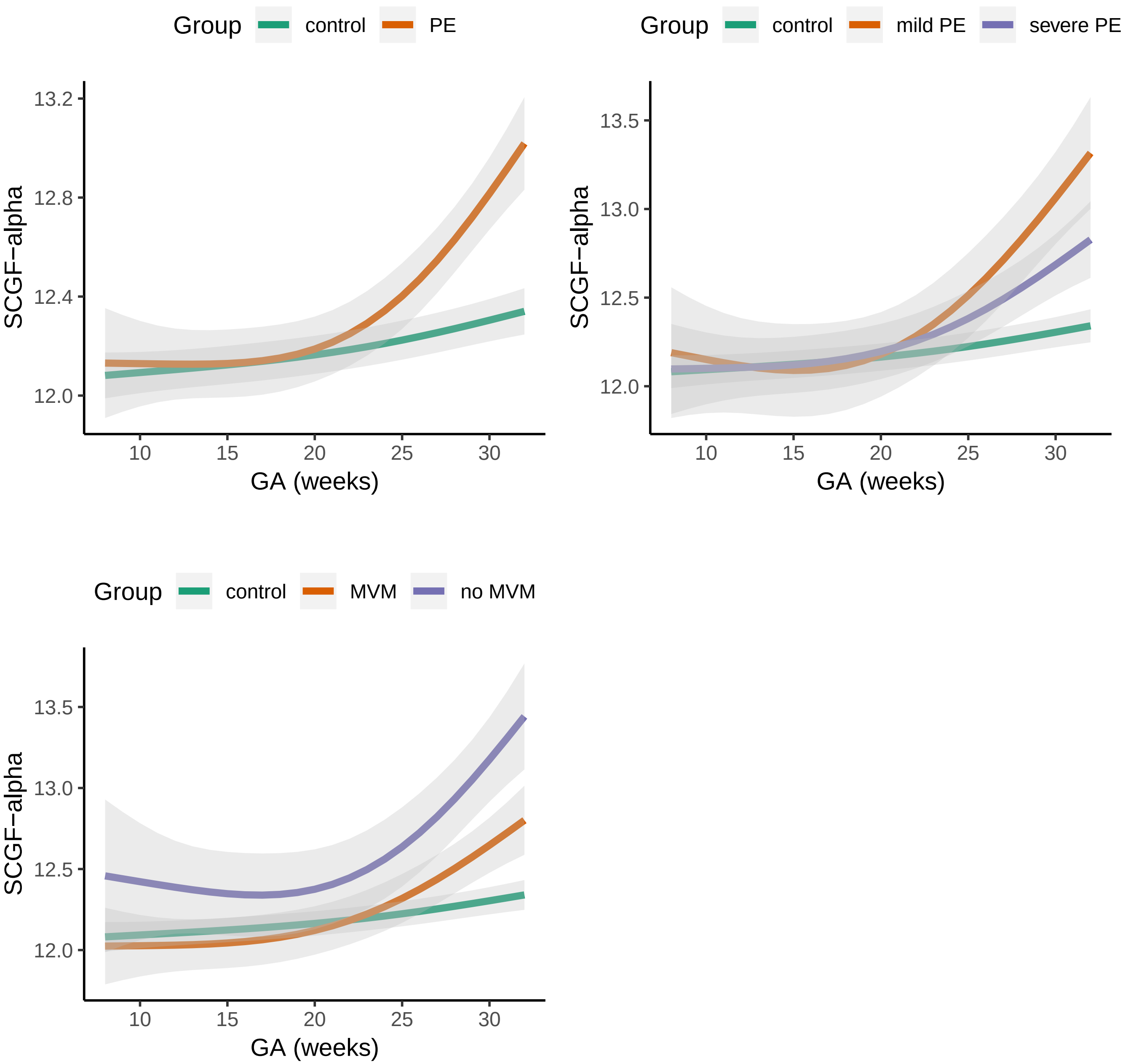

Group
control
PE
13.2
12.8
12.4
12.0
SCGF−alpha
10
15
20
25
30
GA (weeks)
Group
control
mild PE
severe PE
13.5
13.0
12.5
12.0
SCGF−alpha
10
15
20
25
30
GA (weeks)
Group
control
MVM
no MVM
13.5
13.0
12.5
12.0
SCGF−alpha
10
15
20
25
30
GA (weeks)

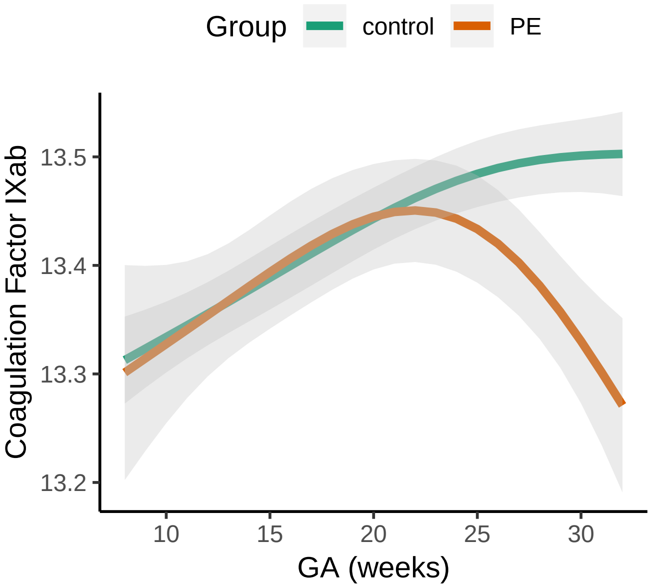

Group
control
PE
Coagulation Factor IXab
13.2
13.3
13.4
13.5
10
15
20
25
30
GA (weeks)

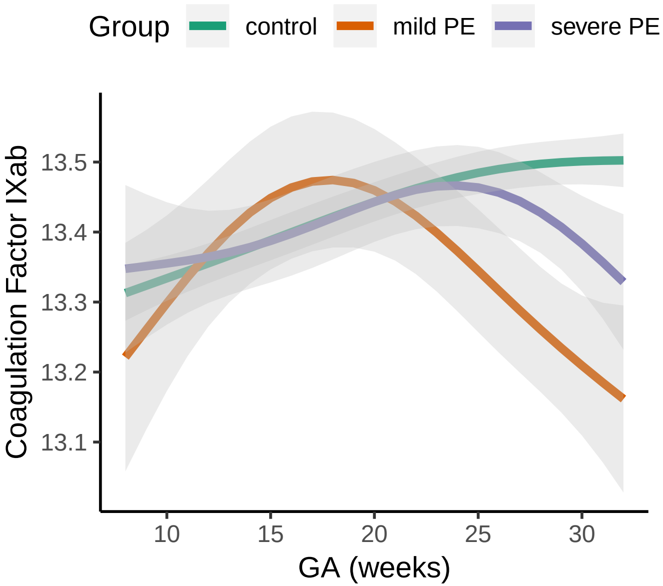

Group
control
mild PE
severe PE
Coagulation Factor IXab
13.1
13.2
13.3
13.4
13.5
10
15
20
25
30
GA (weeks)

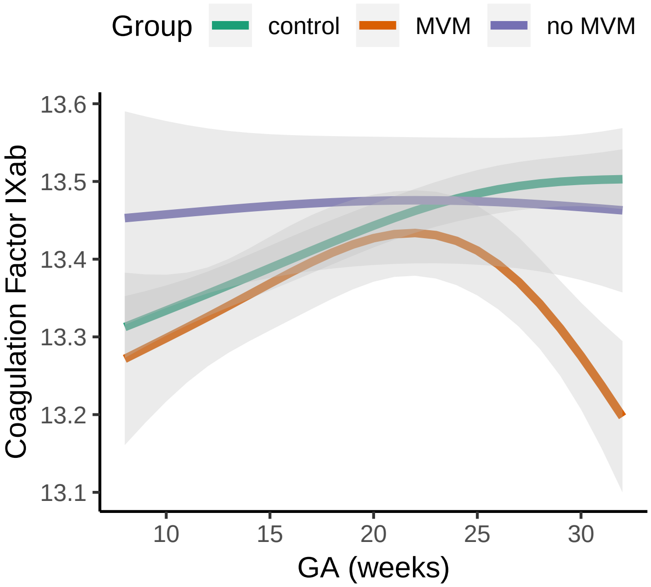

Group
control
MVM
no MVM
Coagulation Factor IXab
13.1
13.2
13.3
13.4
13.5
13.6
10
15
20
25
30
GA (weeks)

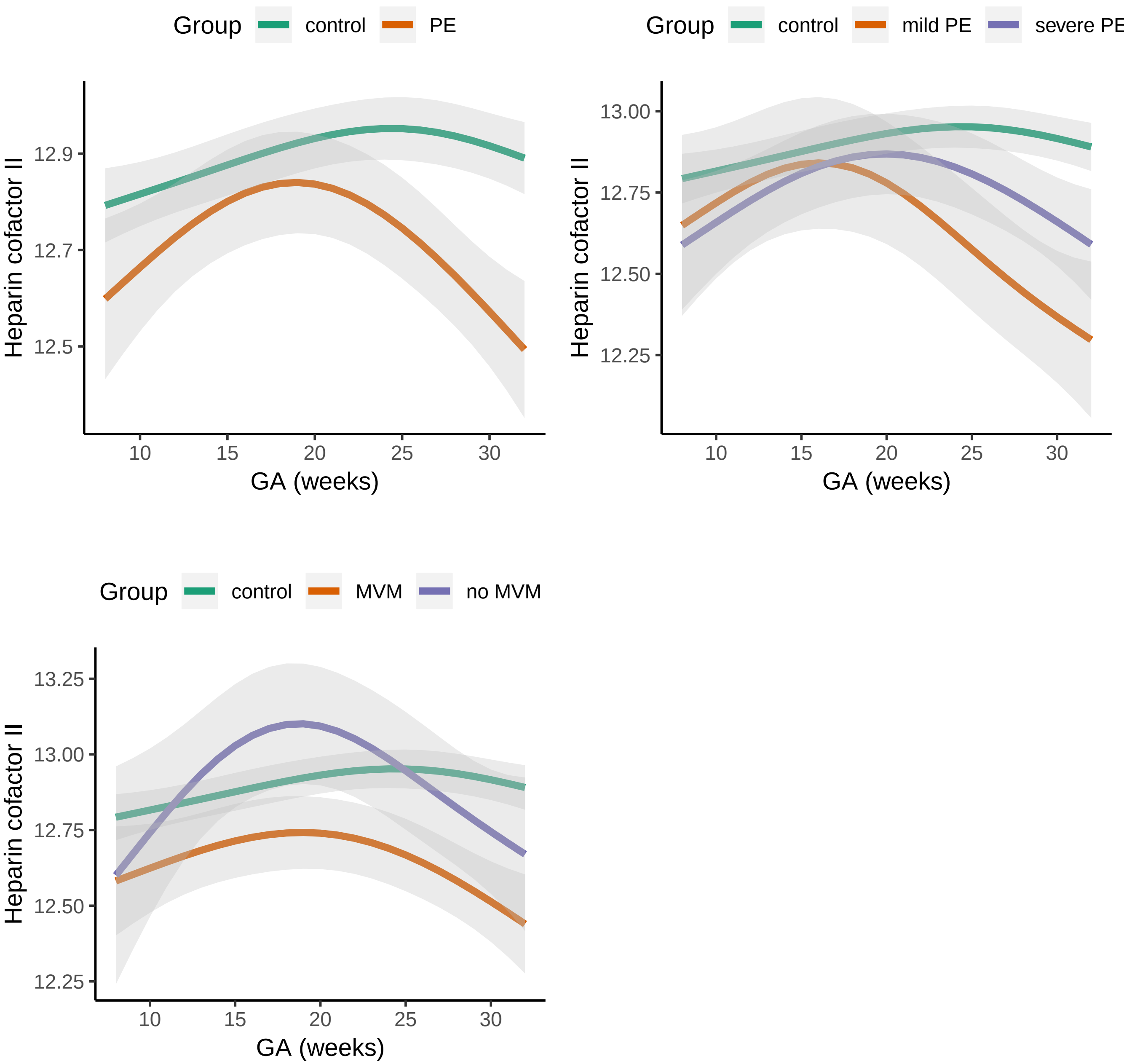
Group
control
PE
Heparin cofactor II
12.9
12.7
12.5
10
15
20
25
30
GA (weeks)
Group
control
mild PE
severe PE
Heparin cofactor II
13.00
12.75
12.50
12.25
10
15
20
25
30
GA (weeks)
Group
control
MVM
no MVM
Heparin cofactor II
13.25
13.00
12.75
12.50
12.25
10
15
20
25
30
GA (weeks)

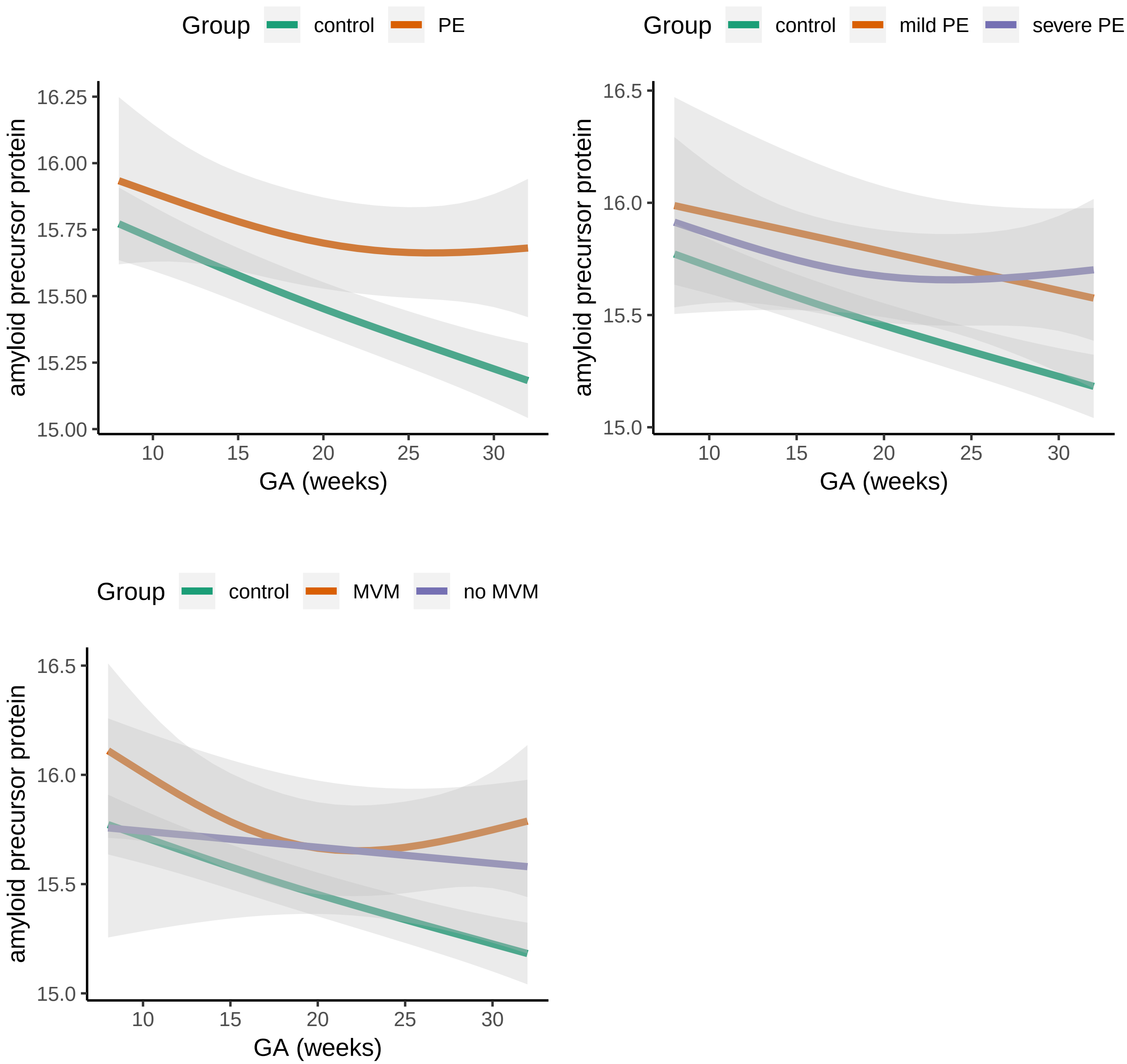
Group
control
PE
amyloid precursor protein
16.25
16.00
15.75
15.50
15.25
15.00
10
15
20
25
30
GA (weeks)
Group
control
mild PE
severe PE
amyloid precursor protein
16.5
16.0
15.5
15.0
10
15
20
25
30
GA (weeks)
Group
control
MVM
no MVM
amyloid precursor protein
16.5
16.0
15.5
15.0
10
15
20
25
30
GA (weeks)

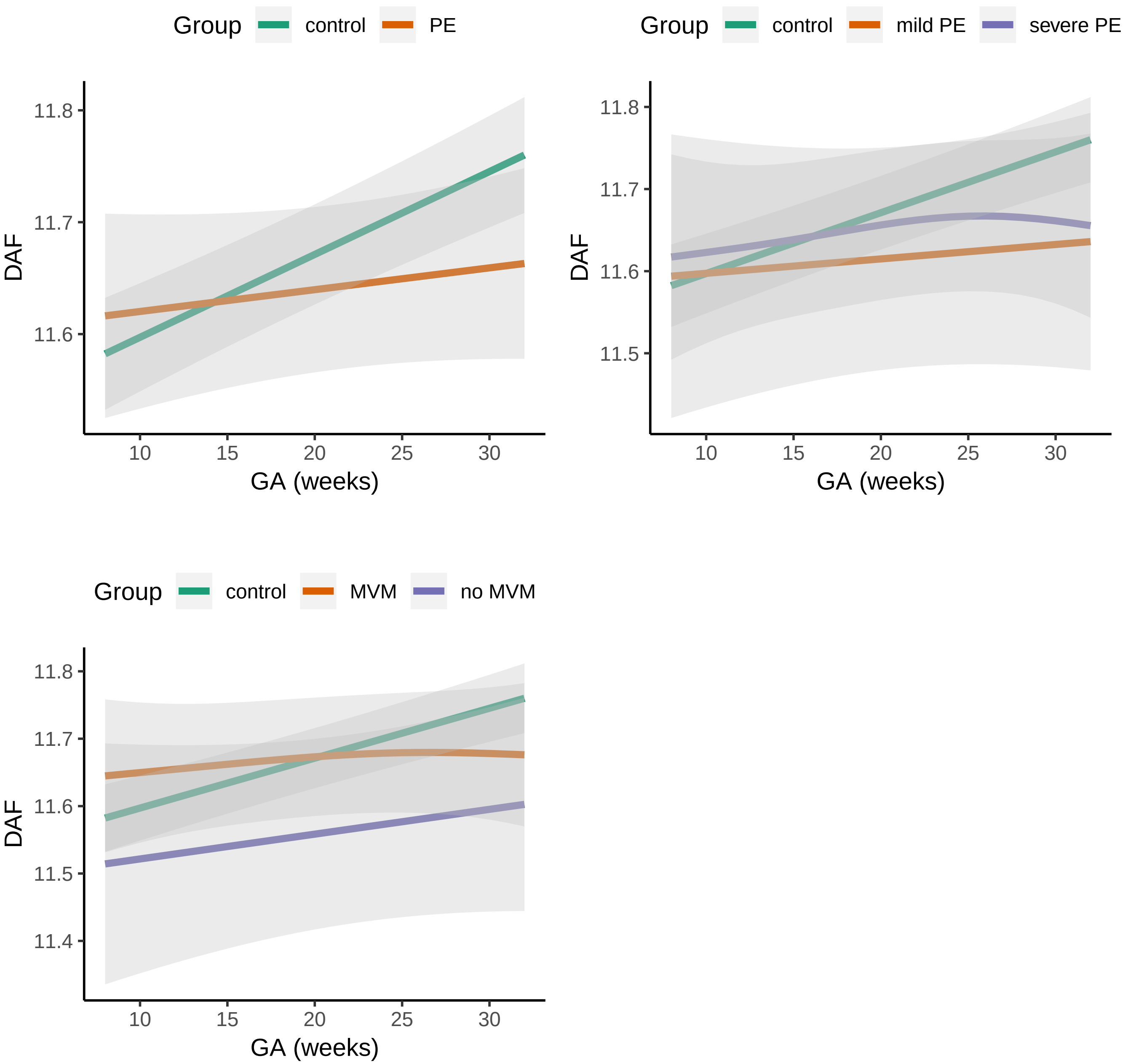

Group
control
PE
DAF
11.8
11.7
11.6
10
15
20
25
30
GA (weeks)
Group
control
mild PE
severe PE
DAF
11.8
11.7
11.6
11.5
10
15
20
25
30
GA (weeks)
Group
control
MVM
no MVM
DAF
11.8
11.7
11.6
11.5
11.4
10
15
20
25
30
GA (weeks)

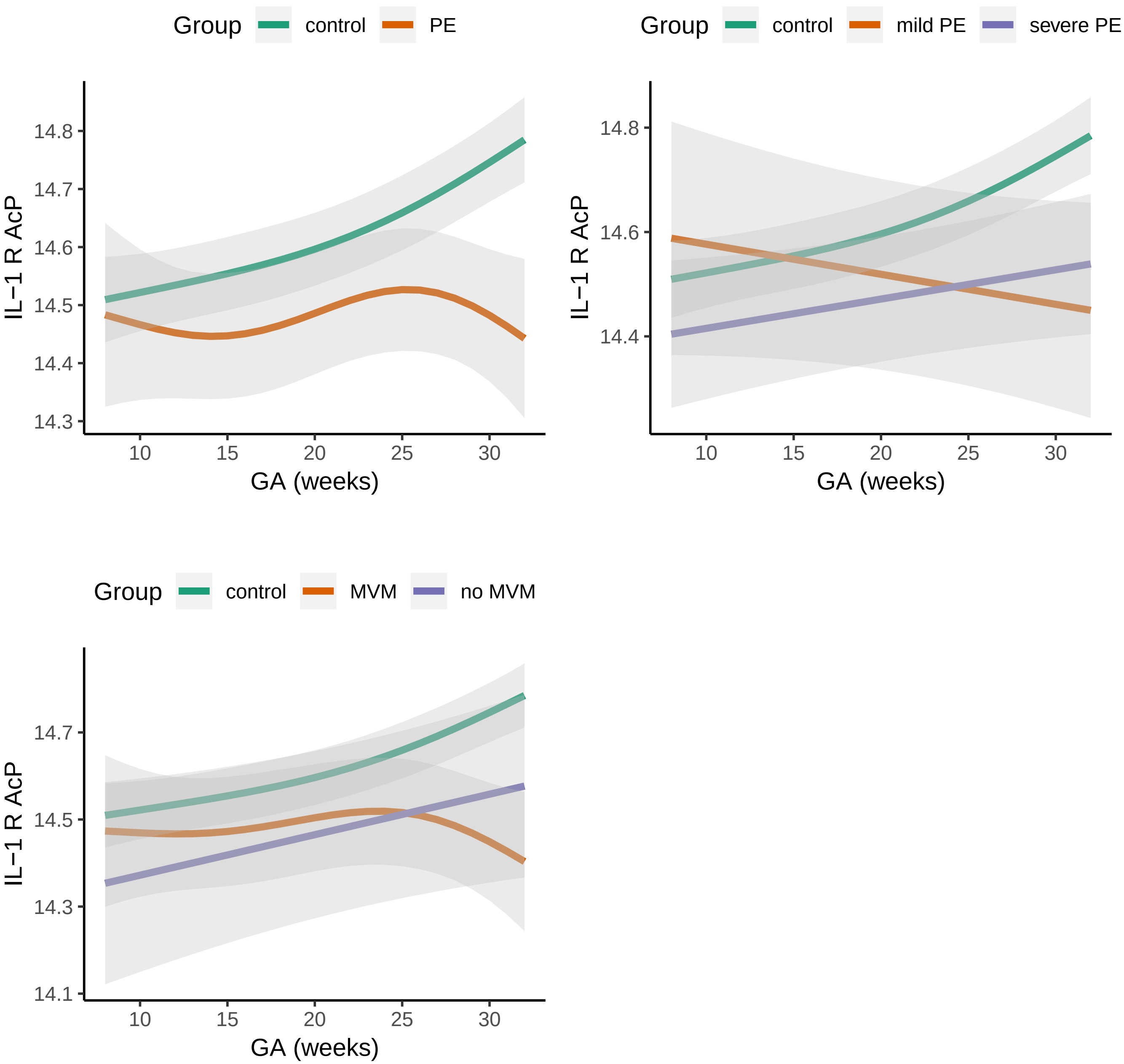

Group
control
PE
IL−1 R AcP
14.3
14.4
14.5
14.6
14.7
14.8
10
15
20
25
30
GA (weeks)
Group
control
mild PE
severe PE
IL−1 R AcP
14.4
14.6
14.8
10
15
20
25
30
GA (weeks)
Group
control
MVM
no MVM
IL−1 R AcP
14.1
14.3
14.5
14.7
10
15
20
25
30
GA (weeks)

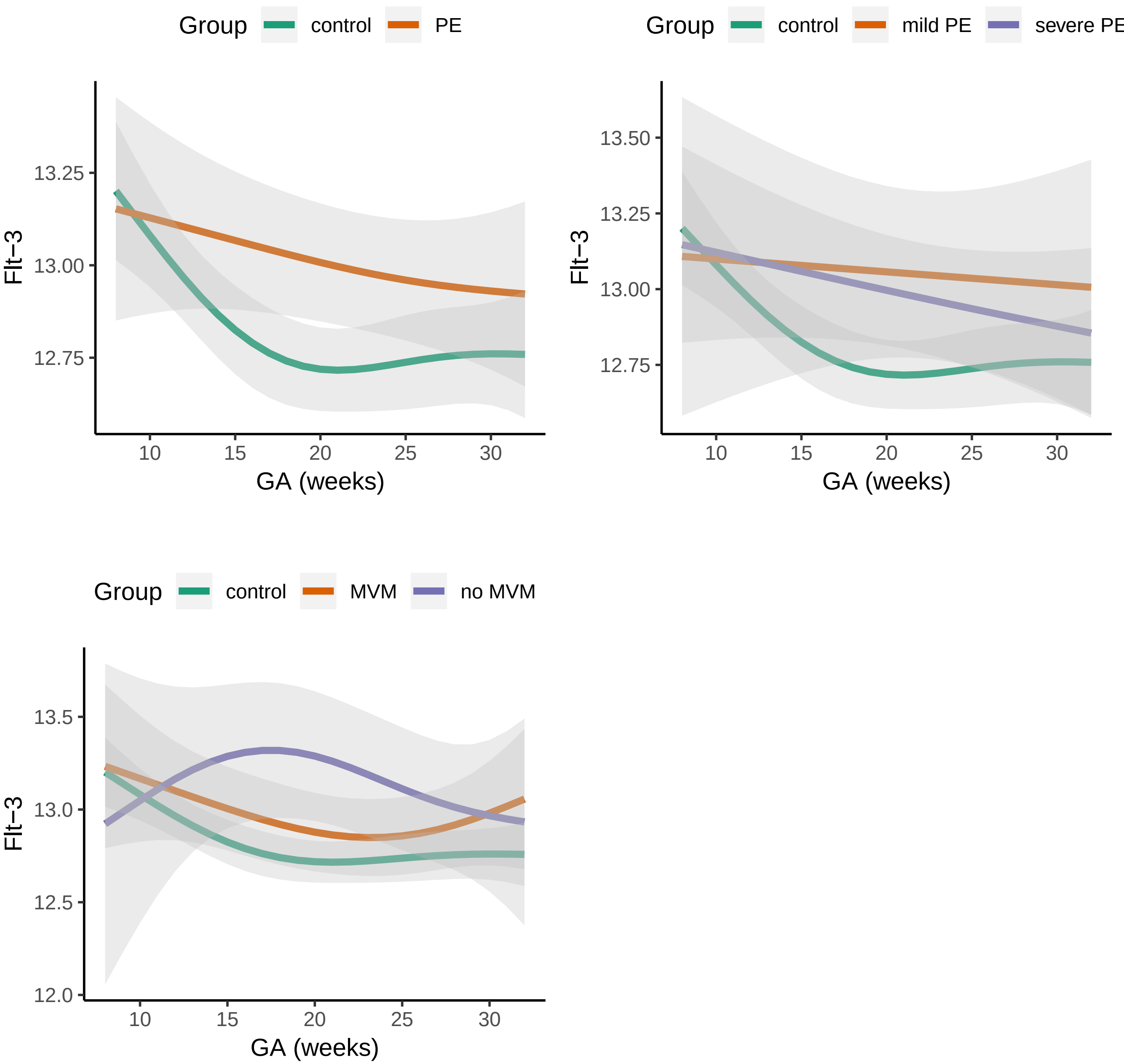

Group
control
PE
Flt−3
13.25
13.00
12.75
10
15
20
25
30
GA (weeks)
Group
control
mild PE
severe PE
Flt−3
13.50
13.25
13.00
12.75
10
15
20
25
30
GA (weeks)
Group
control
MVM
no MVM
Flt−3
13.5
13.0
12.5
12.0
10
15
20
25
30
GA (weeks)

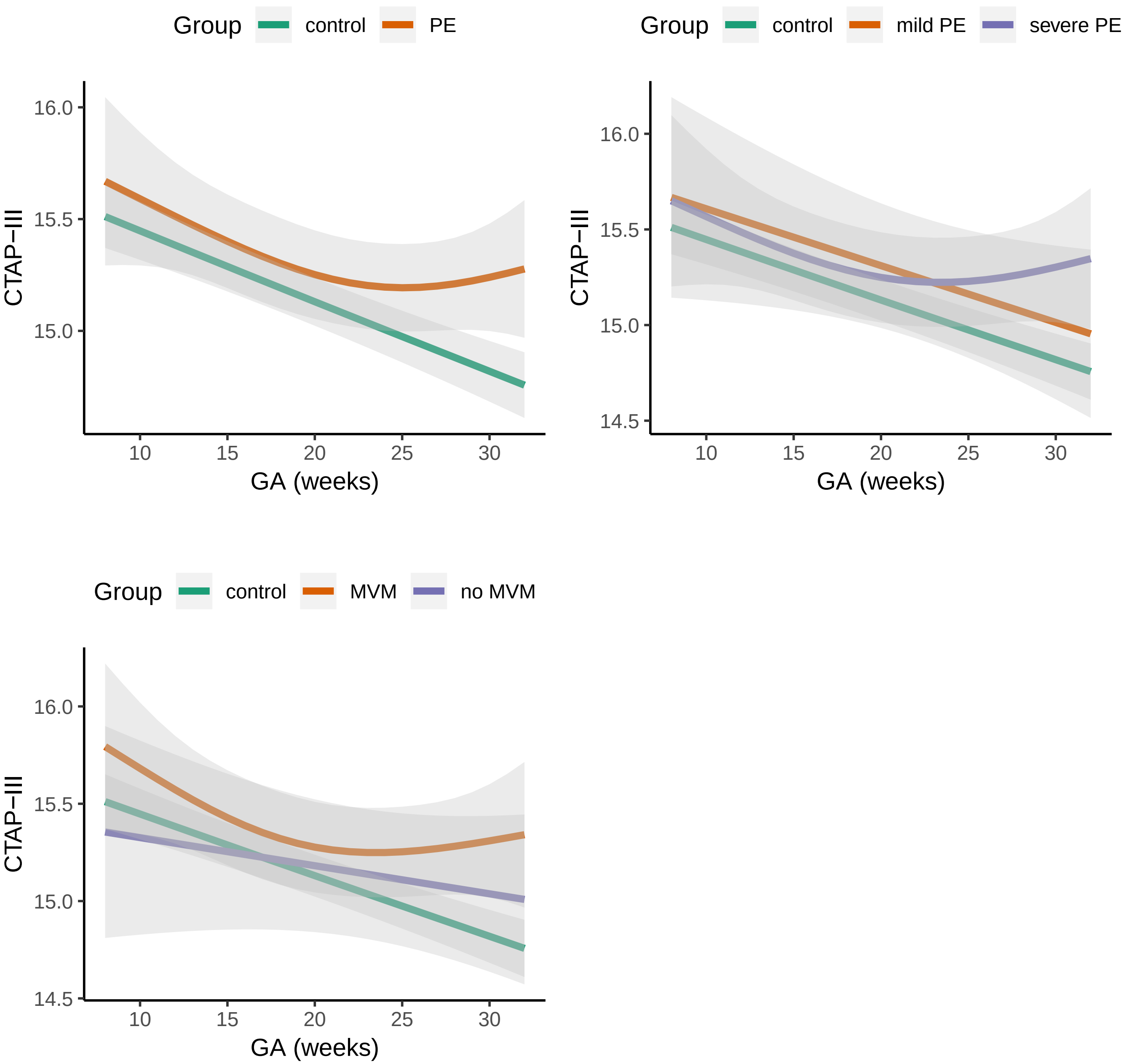
Group
control
PE
16.0
15.5
15.0
CTAP−III
10
15
20
25
30
GA (weeks)
Group
control
mild PE
severe PE
16.0
15.5
15.0
14.5
CTAP−III
10
15
20
25
30
GA (weeks)
Group
control
MVM
no MVM
16.0
15.5
15.0
14.5
CTAP−III
10
15
20
25
30
GA (weeks)

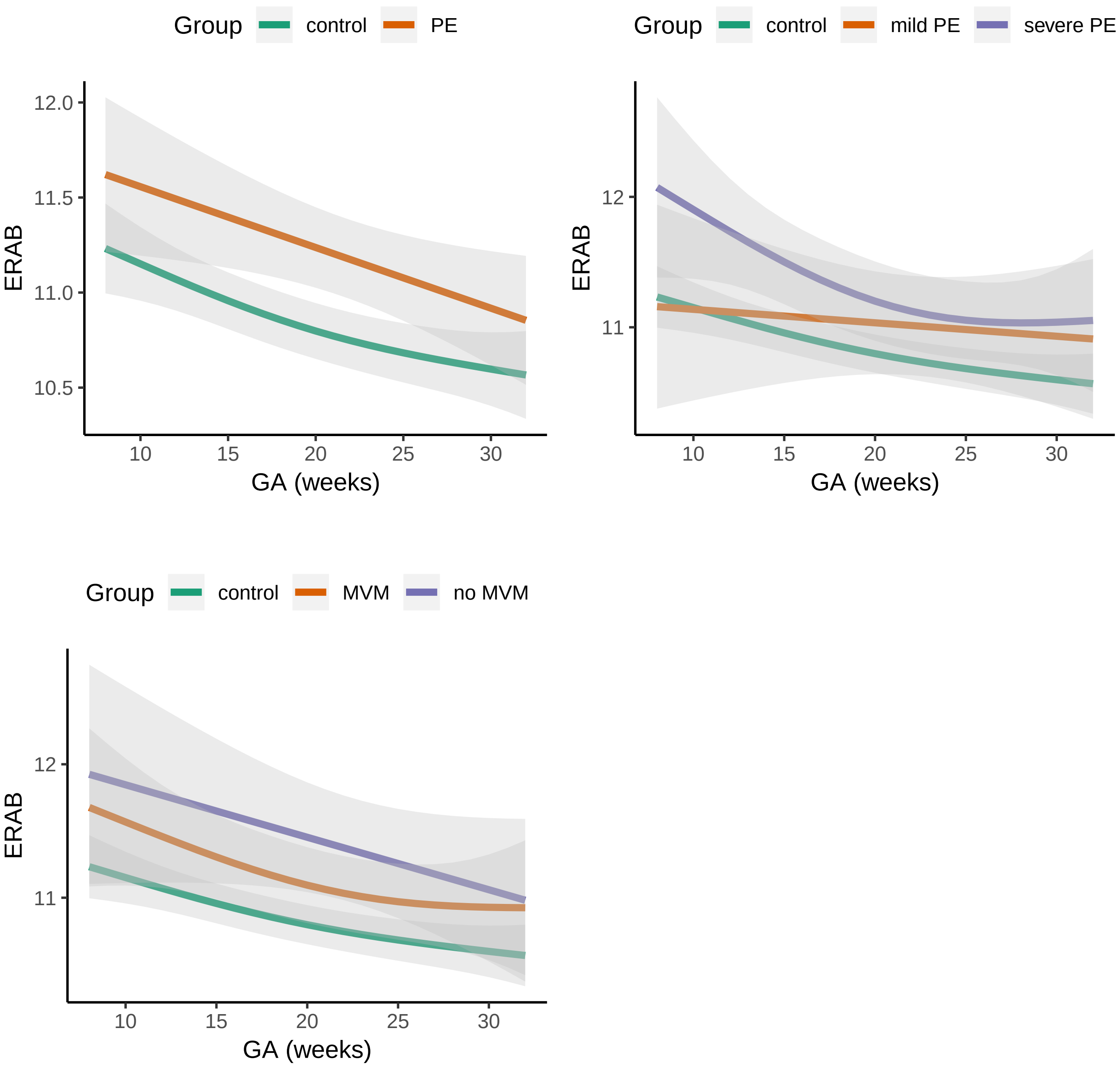

Group
control
PE
12.0
11.5
11.0
10.5
ERAB
10
15
20
25
30
GA (weeks)
Group
control
mild PE
severe PE
12
11
ERAB
10
15
20
25
30
GA (weeks)
Group
control
MVM
no MVM
12
11
ERAB
10
15
20
25
30
GA (weeks)

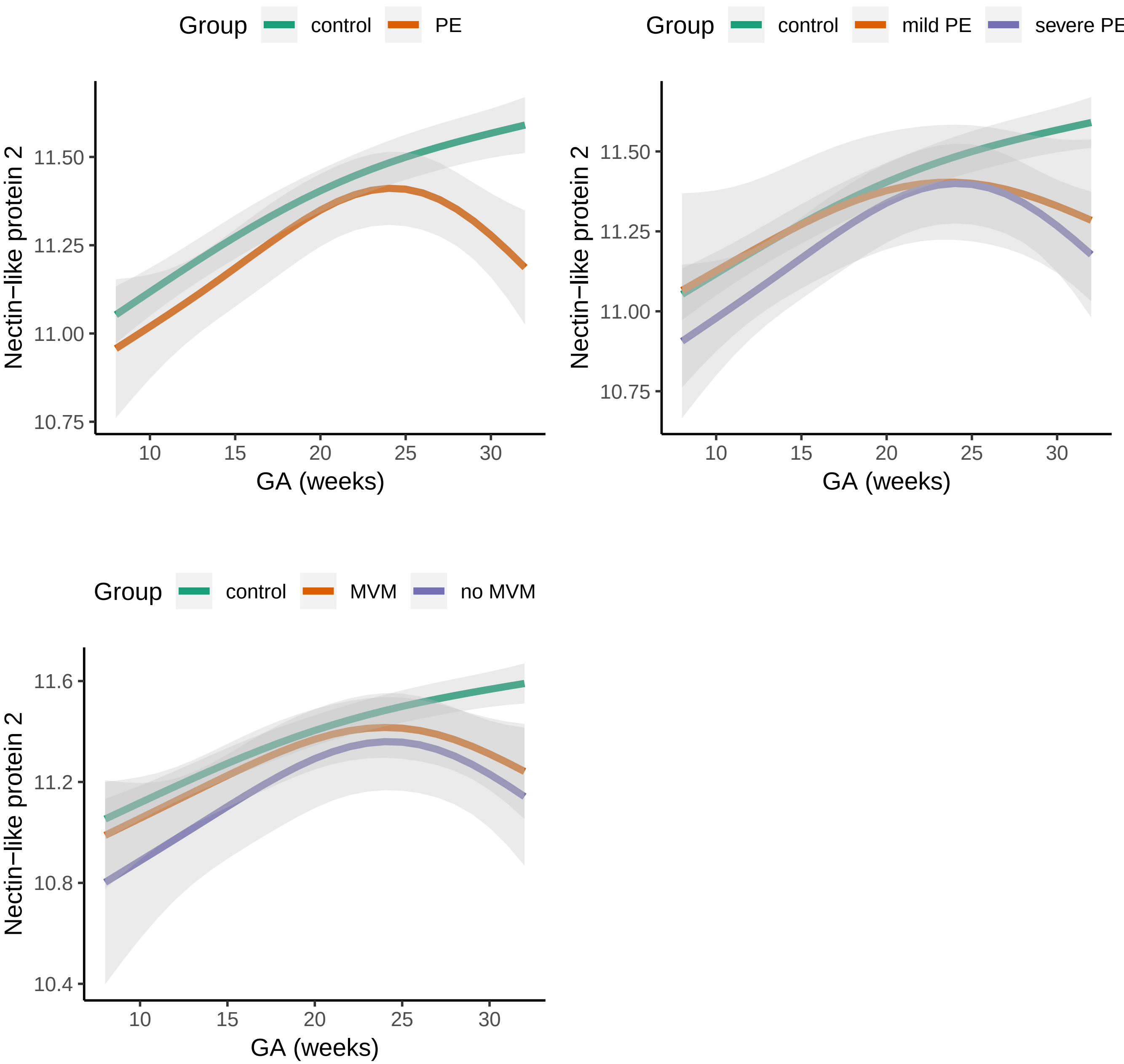
Group
control
PE
Nectin−like protein 2
11.50
11.25
11.00
10.75
10
15
20
25
30
GA (weeks)
Group
control
mild PE
severe PE
Nectin−like protein 2
11.50
11.25
11.00
10.75
10
15
20
25
30
GA (weeks)
Group
control
MVM
no MVM
Nectin−like protein 2
11.6
11.2
10.8
10.4
10
15
20
25
30
GA (weeks)

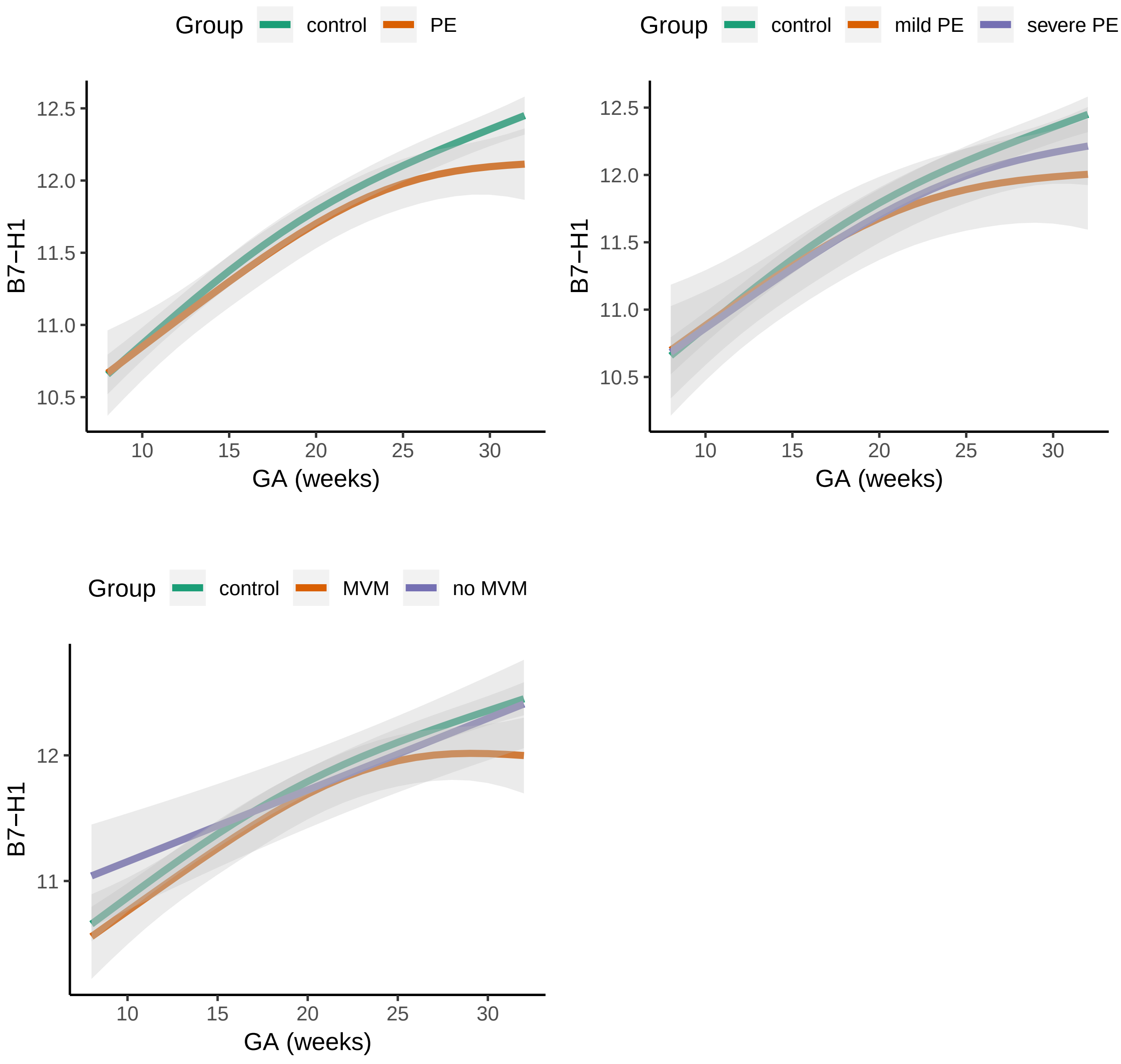
Group
control
PE
12.5
12.0
11.5
11.0
10.5
B7−H1
10
15
20
25
30
GA (weeks)
Group
control
mild PE
severe PE
12.5
12.0
11.5
11.0
10.5
B7−H1
10
15
20
25
30
GA (weeks)
Group
control
MVM
no MVM
12
11
B7−H1
10
15
20
25
30
GA (weeks)

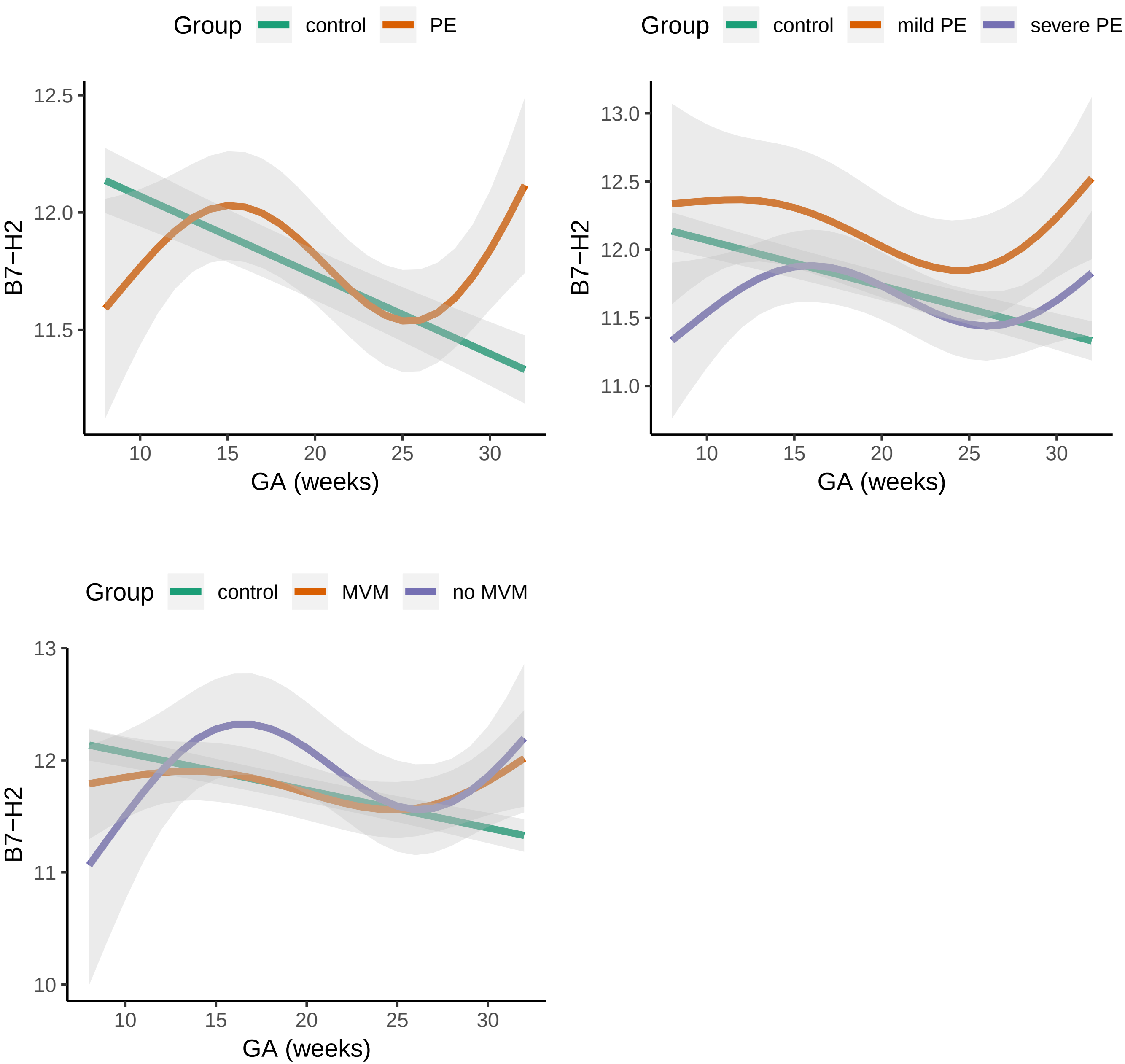
Group
control
PE
B7−H2
12.5
12.0
11.5
10
15
20
25
30
GA (weeks)
Group
control
mild PE
severe PE
B7−H2
13.0
12.5
12.0
11.5
11.0
10
15
20
25
30
GA (weeks)
Group
control
MVM
no MVM
B7−H2
13
12
11
10
10
15
20
25
30
GA (weeks)

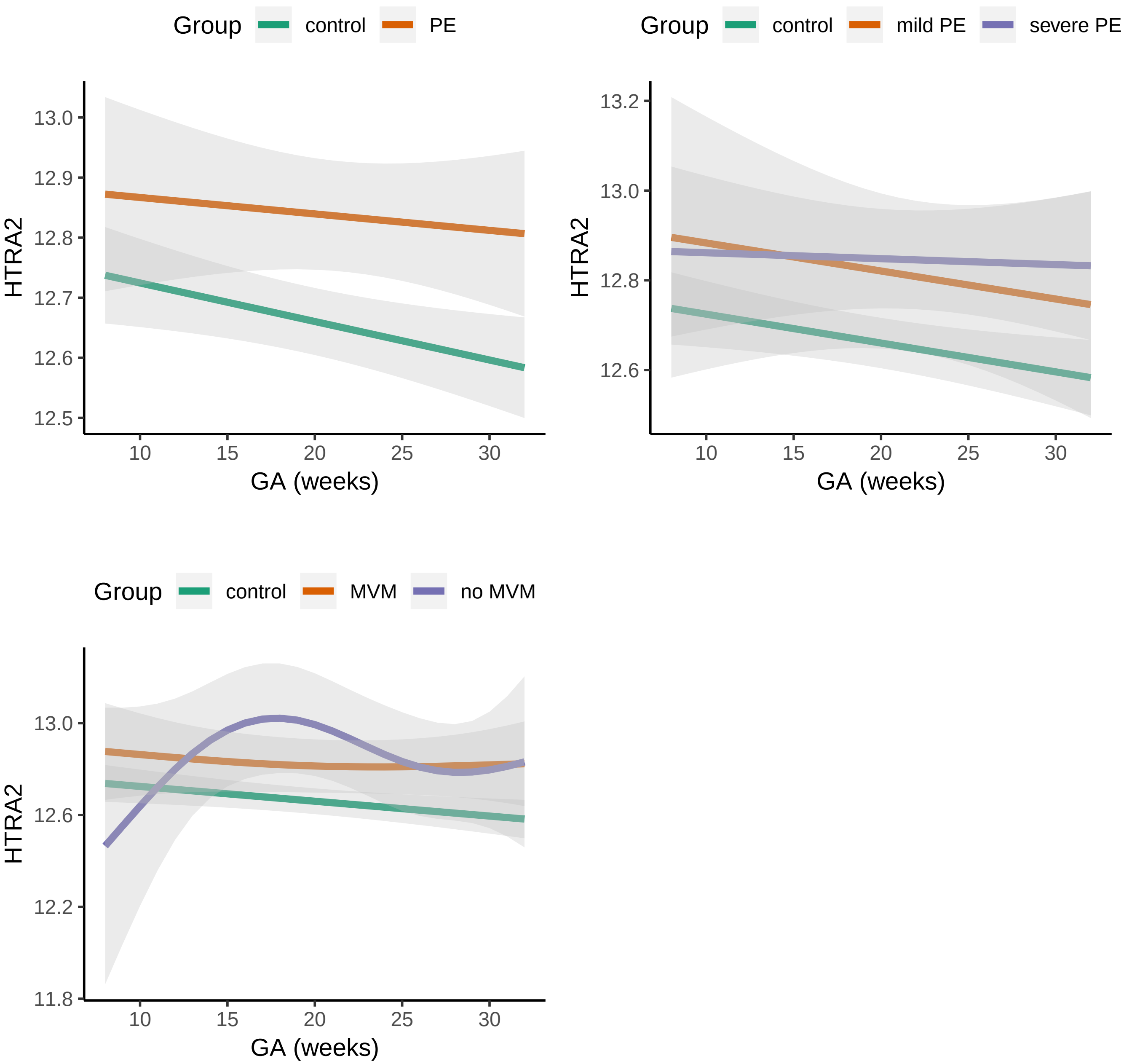

Group
control
PE
HTRA2
GA (weeks)
13.0
12.9
12.8
12.7
12.6
12.5
10
15
20
25
30
Group
control
mild PE
severe PE
HTRA2
GA (weeks)
13.2
13.0
12.8
12.6
Group
control
MVM
no MVM
HTRA2
GA (weeks)
13.0
12.6
12.2
11.8

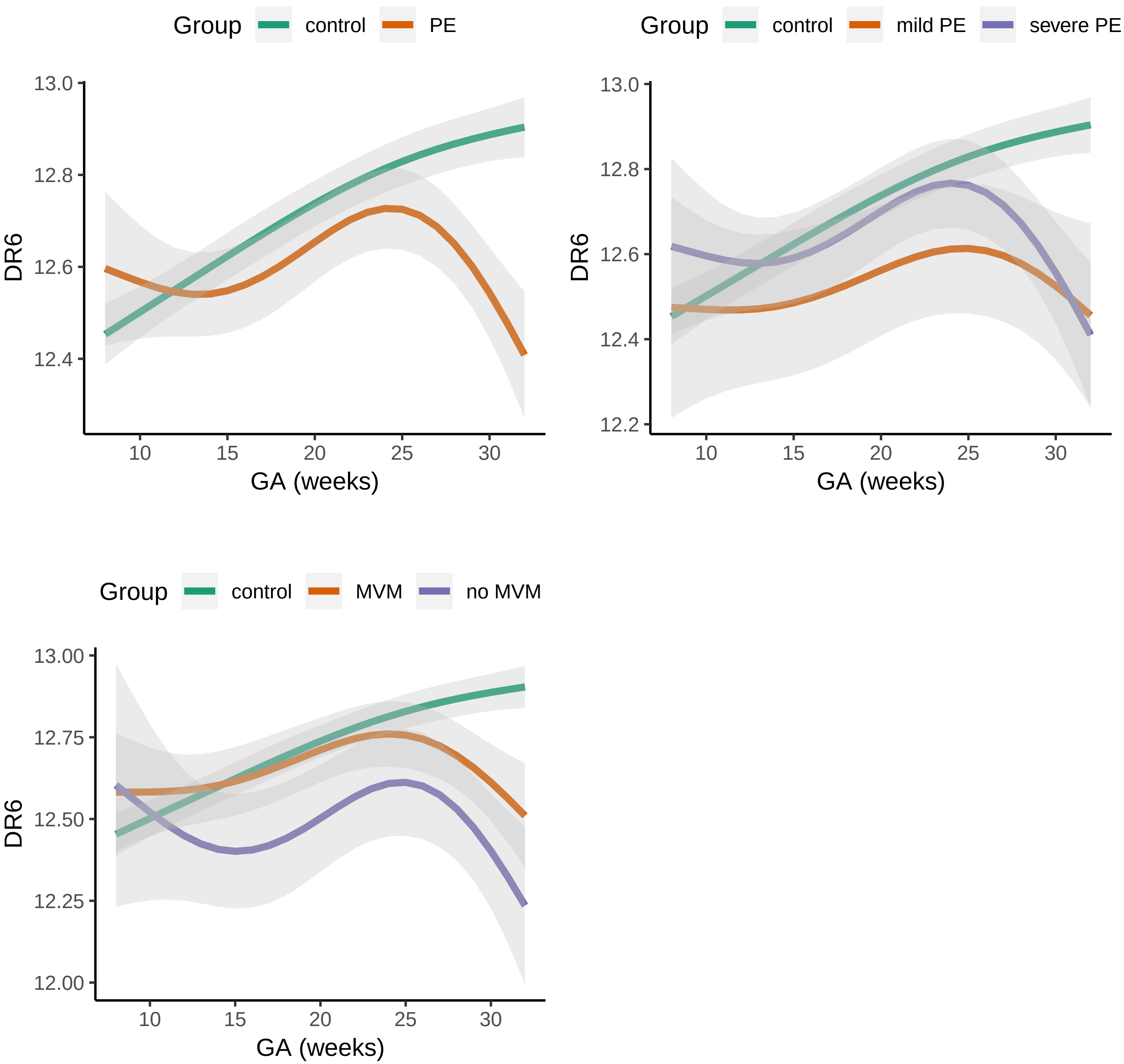
Group
control
PE
13.0
12.8
12.6
12.4
DR6
10
15
20
25
30
GA (weeks)
Group
control
mild PE
severe PE
13.0
12.8
12.6
12.4
12.2
DR6
10
15
20
25
30
GA (weeks)
Group
control
MVM
no MVM
13.00
12.75
12.50
12.25
12.00
DR6
10
15
20
25
30
GA (weeks)

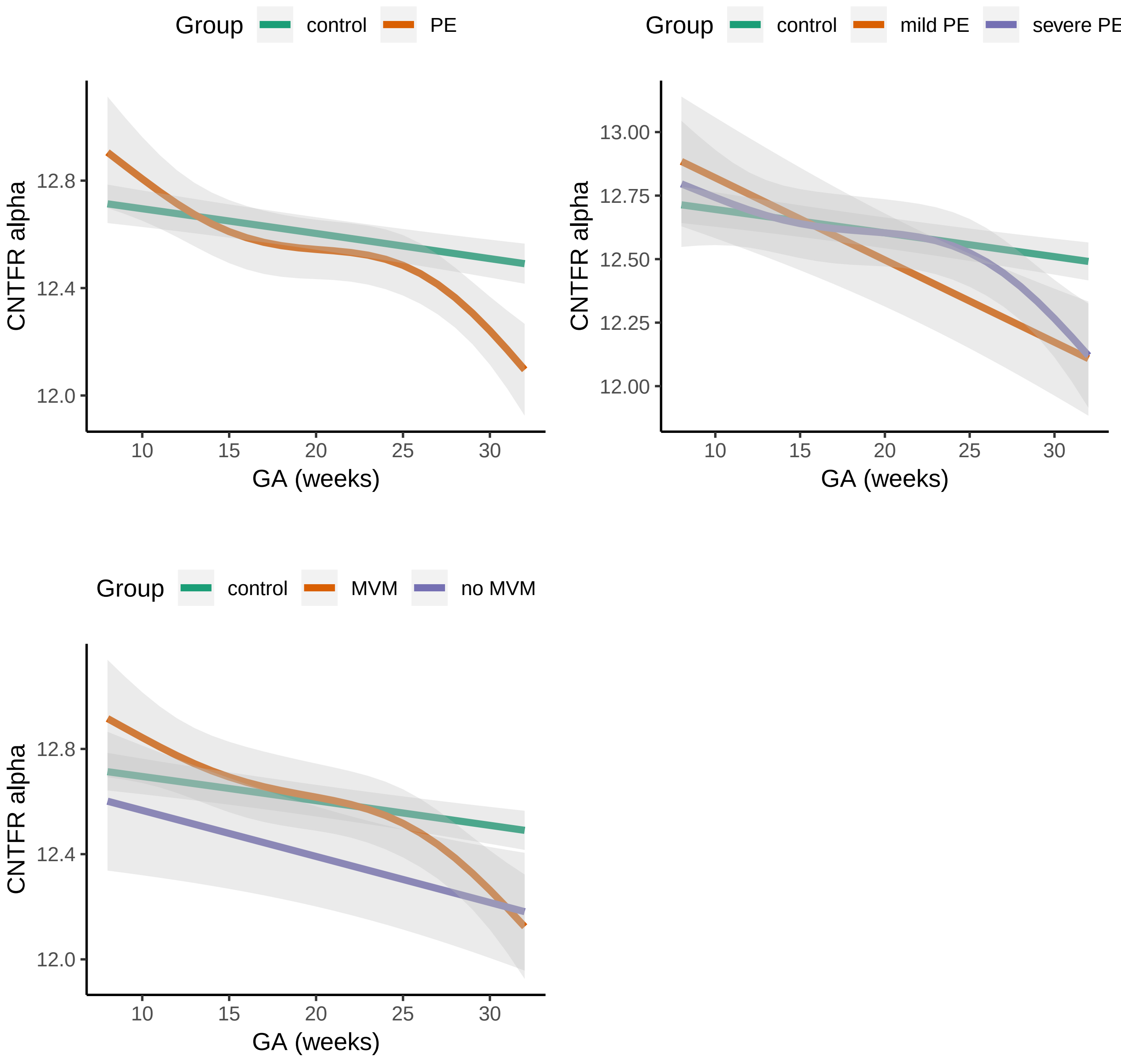

Group
control
PE
CNTFR alpha
12.0
12.4
12.8
10
15
20
25
30
GA (weeks)
Group
control
mild PE
severe PE
CNTFR alpha
12.00
12.25
12.50
12.75
13.00
10
15
20
25
30
GA (weeks)
Group
control
MVM
no MVM
CNTFR alpha
12.0
12.4
12.8
10
15
20
25
30
GA (weeks)

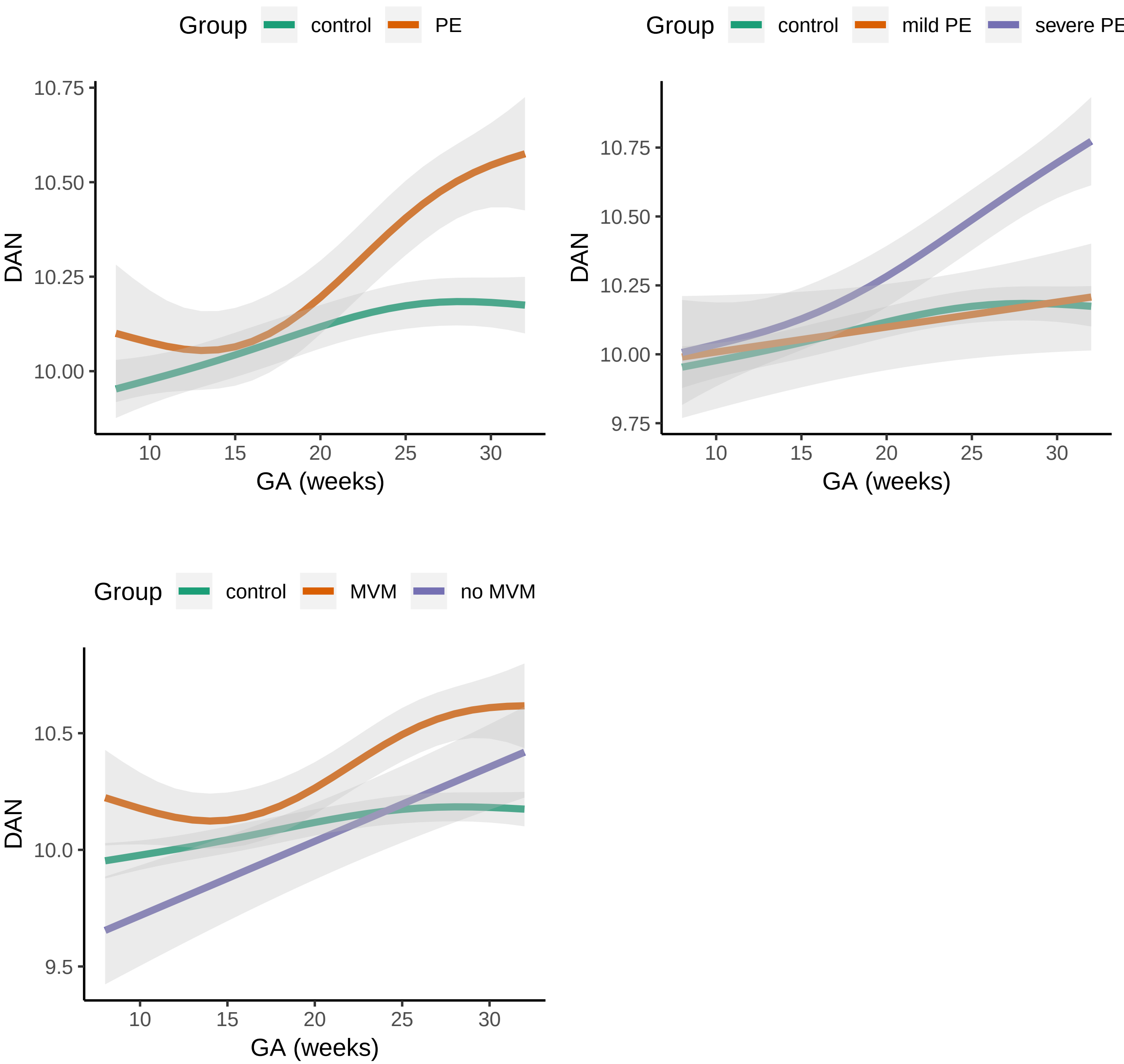
Group
control
PE
10.75
10.50
10.25
10.00
DAN
10
15
20
25
30
GA (weeks)
Group
control
mild PE
severe PE
10.75
10.50
10.25
10.00
9.75
DAN
10
15
20
25
30
GA (weeks)
Group
control
MVM
no MVM
10.5
10.0
9.5
DAN
10
15
20
25
30
GA (weeks)

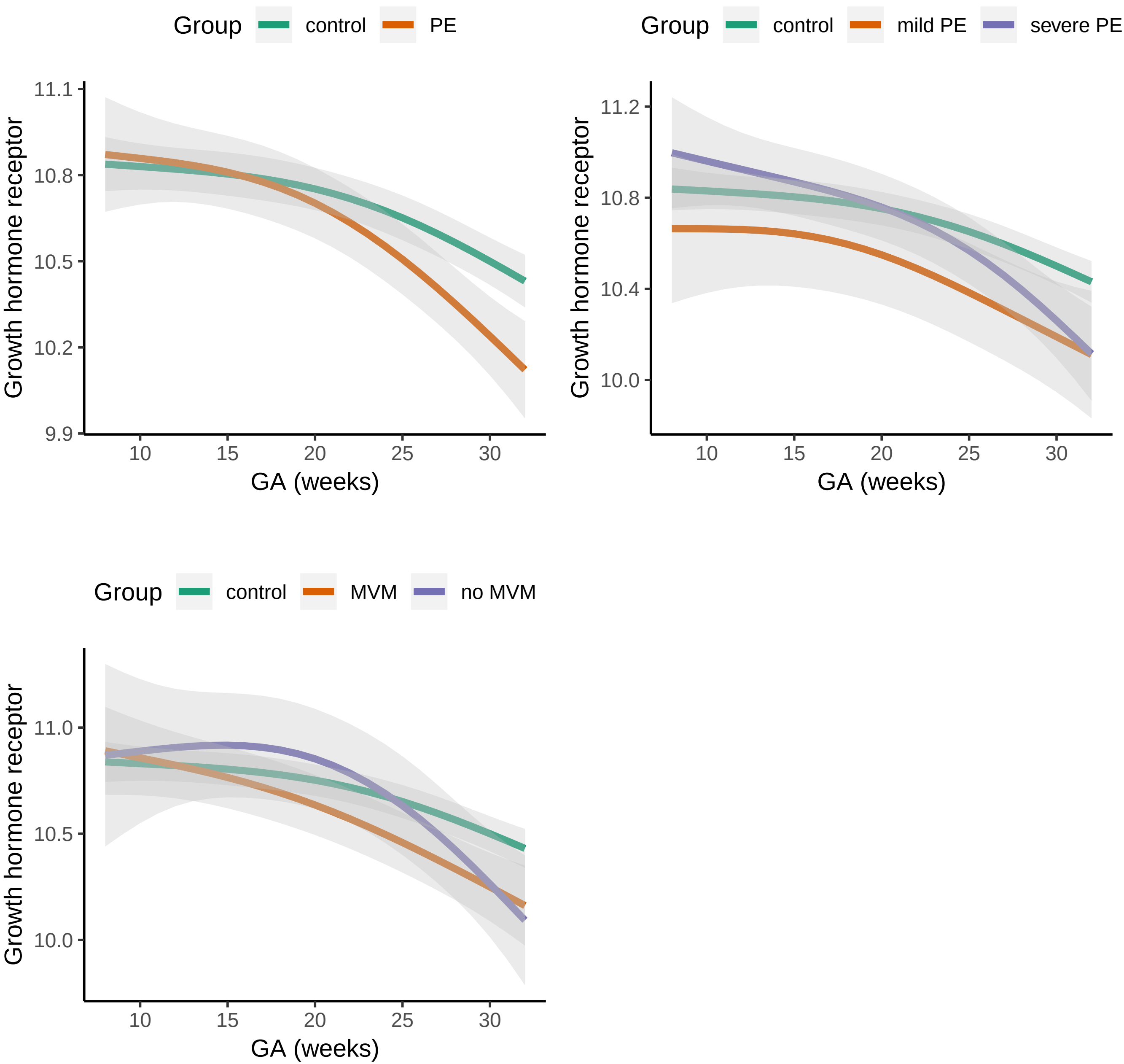
Group
control
PE
Growth hormone receptor
11.1
10.8
10.5
10.2
9.9
10
15
20
25
30
GA (weeks)
Group
control
mild PE
severe PE
Growth hormone receptor
11.2
10.8
10.4
10.0
10
15
20
25
30
GA (weeks)
Group
control
MVM
no MVM
Growth hormone receptor
11.0
10.5
10.0
10
15
20
25
30
GA (weeks)

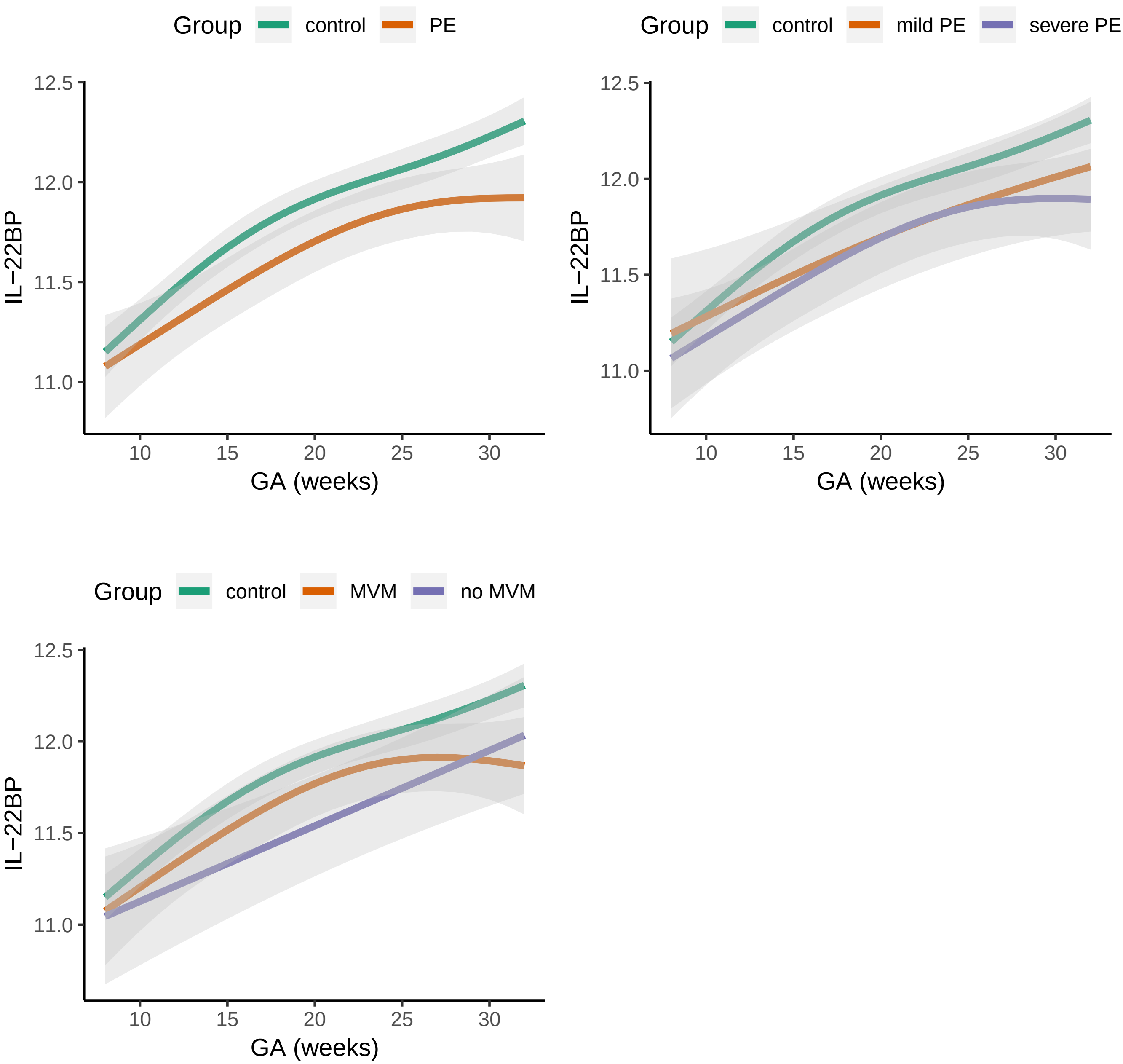

Group
control
PE
12.5
12.0
11.5
11.0
IL−22BP
10
15
20
25
30
GA (weeks)
Group
control
mild PE
severe PE
12.5
12.0
11.5
11.0
IL−22BP
10
15
20
25
30
GA (weeks)
Group
control
MVM
no MVM
12.5
12.0
11.5
11.0
IL−22BP
10
15
20
25
30
GA (weeks)

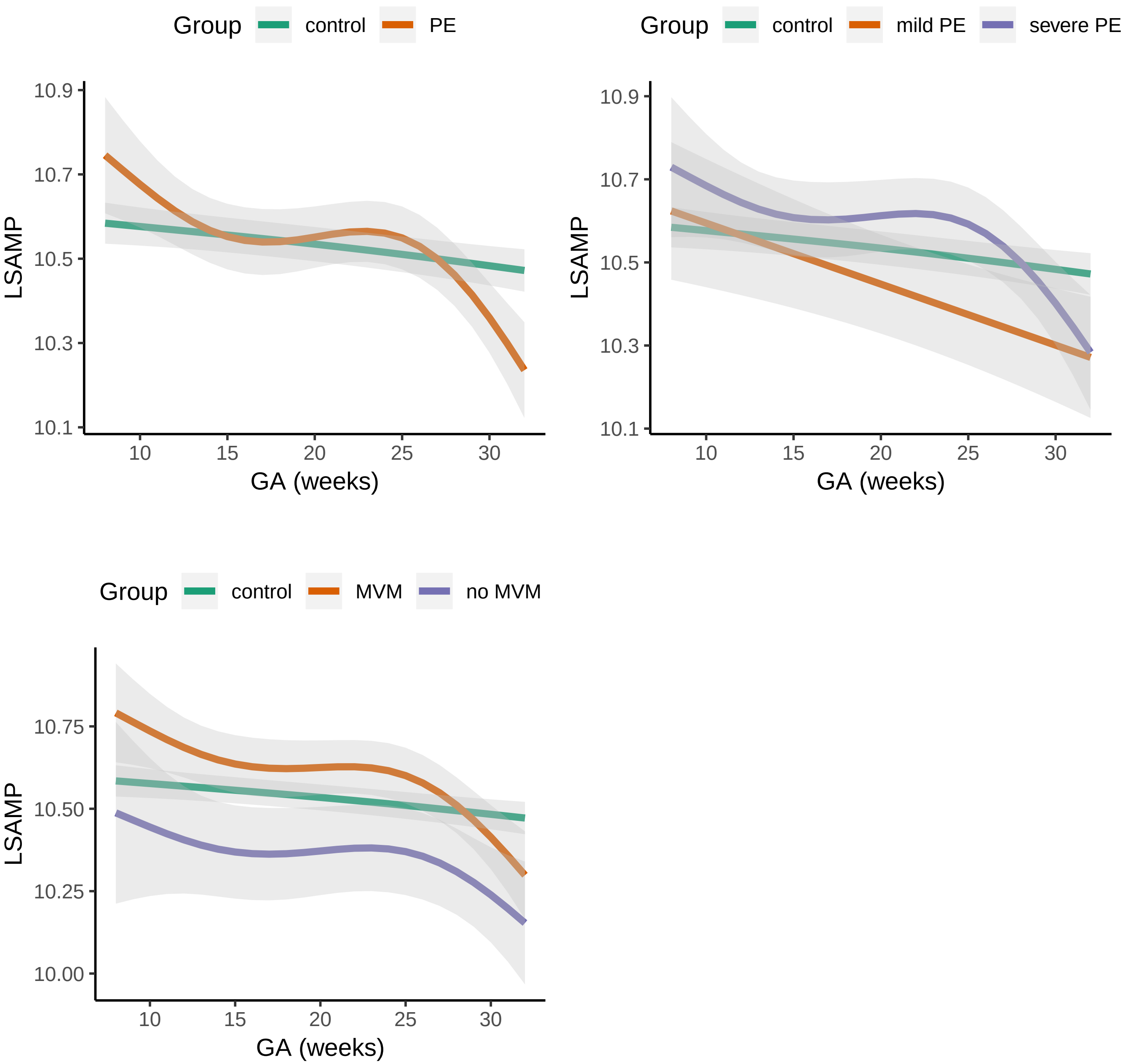

Group
control
PE
LSAMP
10.9
10.7
10.5
10.3
10.1
10
15
20
25
30
GA (weeks)
Group
control
mild PE
severe PE
LSAMP
10.9
10.7
10.5
10.3
10.1
10
15
20
25
30
GA (weeks)
Group
control
MVM
no MVM
LSAMP
10.75
10.50
10.25
10.00
10
15
20
25
30
GA (weeks)

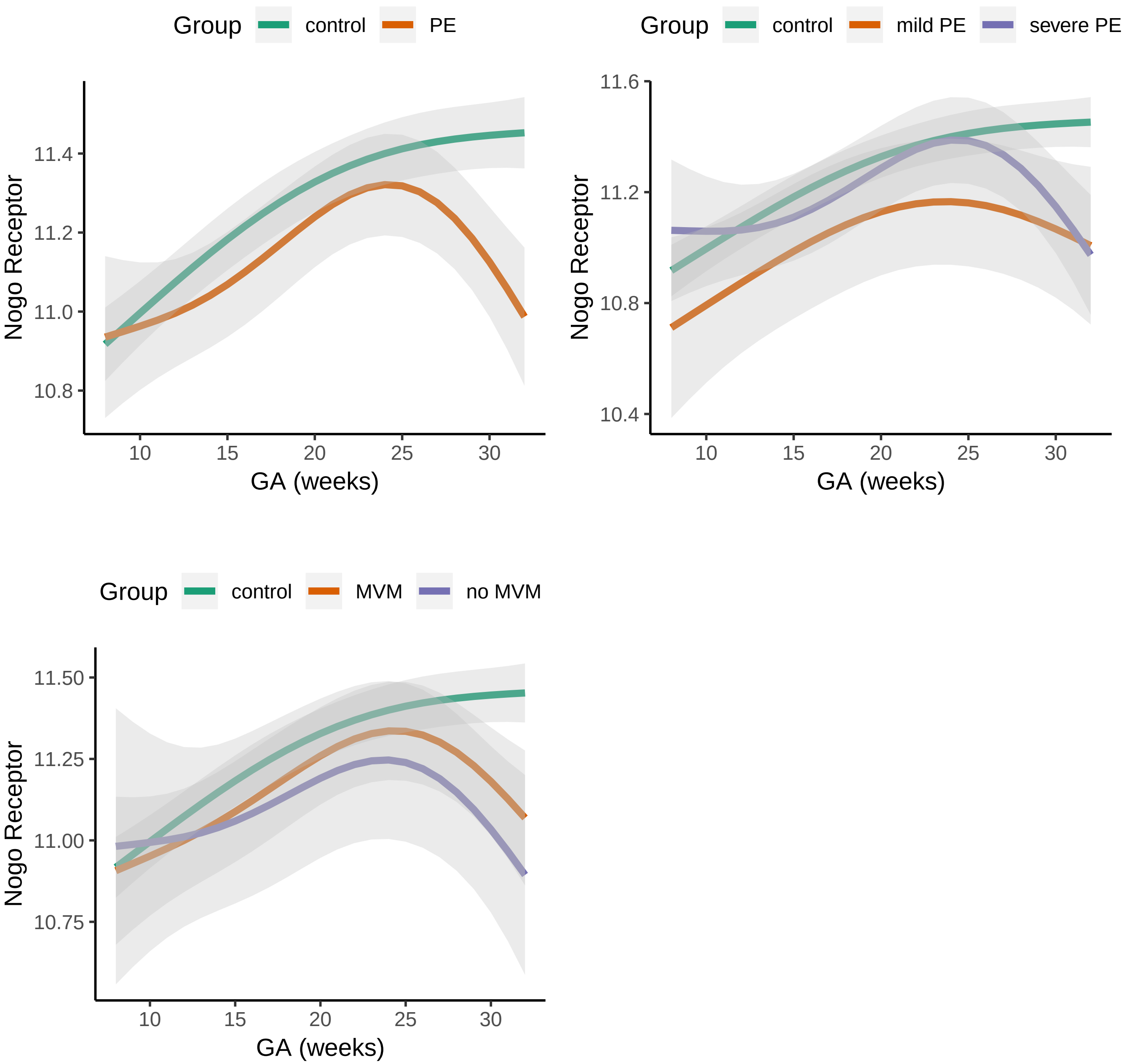

Group control PE
Nogo Receptor
11.4
11.2
11.0
10.8
10 15 20 25 30
GA (weeks)
Group control mild PE severe PE
Nogo Receptor
11.6
11.2
10.8
10.4
10 15 20 25 30
GA (weeks)
Group control MVM no MVM
Nogo Receptor
11.50
11.25
11.00
10.75
10 15 20 25 30
GA (weeks)

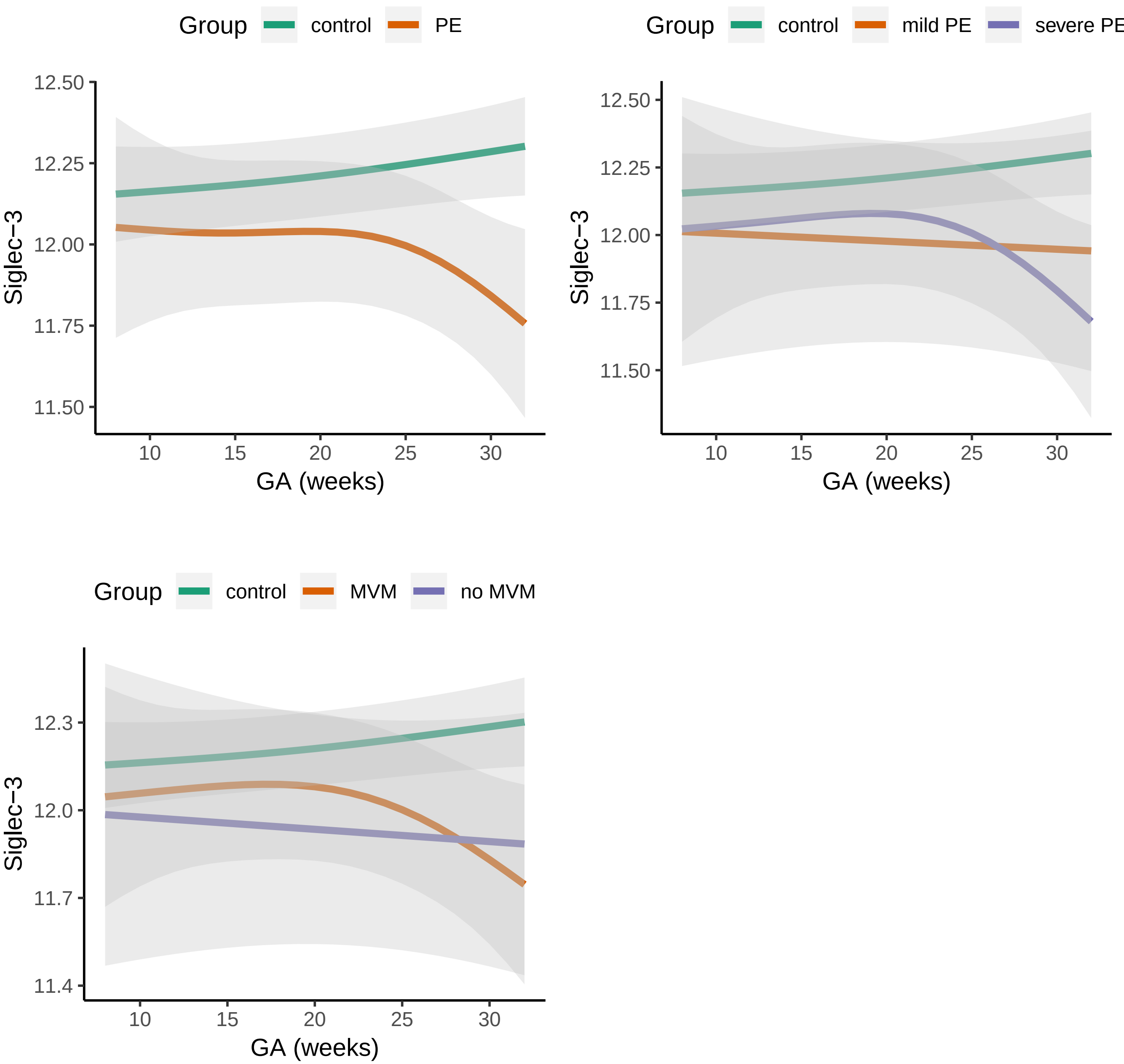
Group
control
PE
12.50
12.25
12.00
11.75
11.50
Siglec−3
10
15
20
25
30
GA (weeks)
Group
control
mild PE
severe PE
12.50
12.25
12.00
11.75
11.50
Siglec−3
10
15
20
25
30
GA (weeks)
Group
control
MVM
no MVM
12.3
12.0
11.7
11.4
Siglec−3
10
15
20
25
30
GA (weeks)

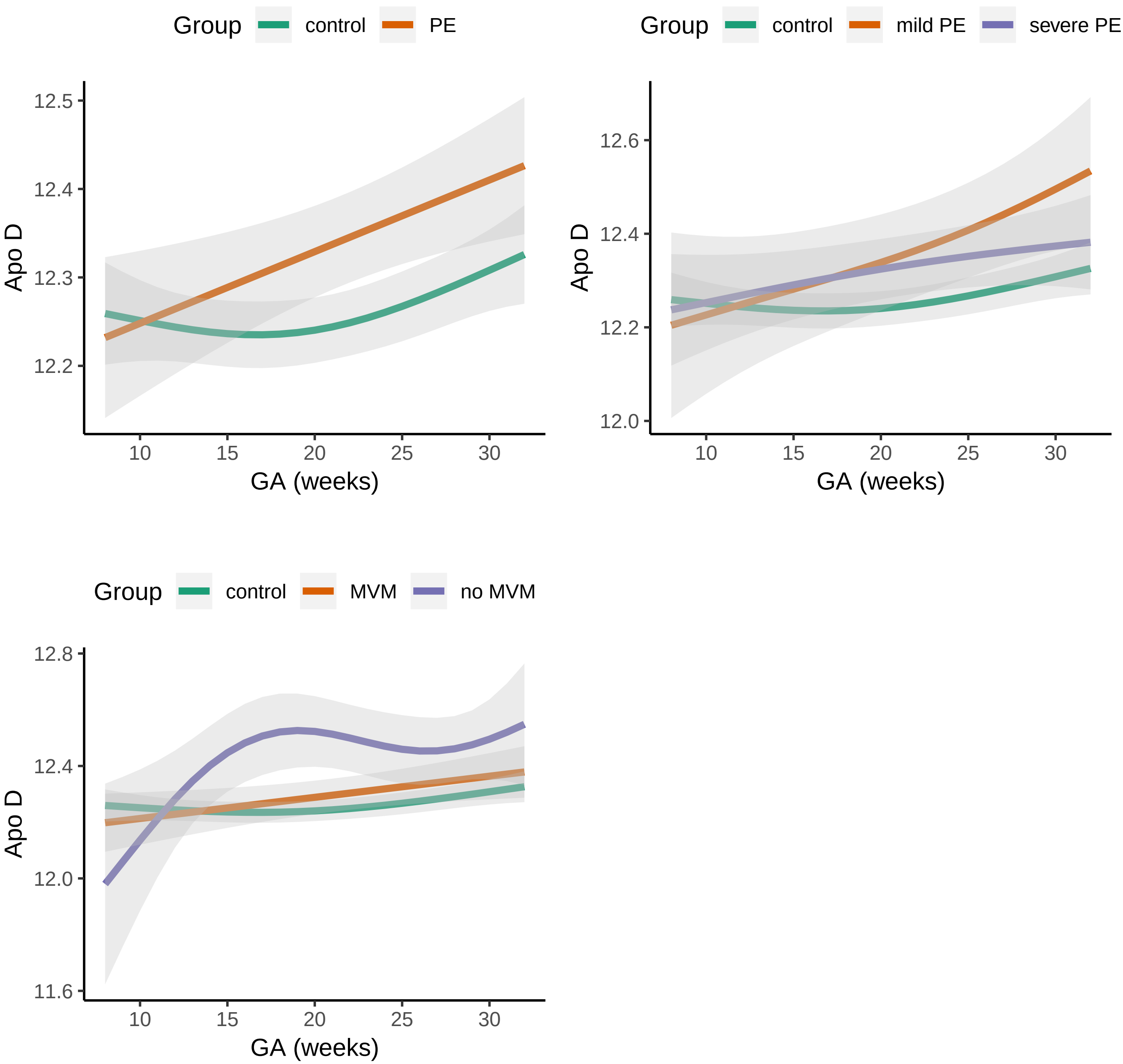

Group
control
PE
Apo D
12.5
12.4
12.3
12.2
10
15
20
25
30
GA (weeks)
Group
control
mild PE
severe PE
Apo D
12.6
12.4
12.2
12.0
10
15
20
25
30
GA (weeks)
Group
control
MVM
no MVM
Apo D
12.8
12.4
12.0
11.6
10
15
20
25
30
GA (weeks)

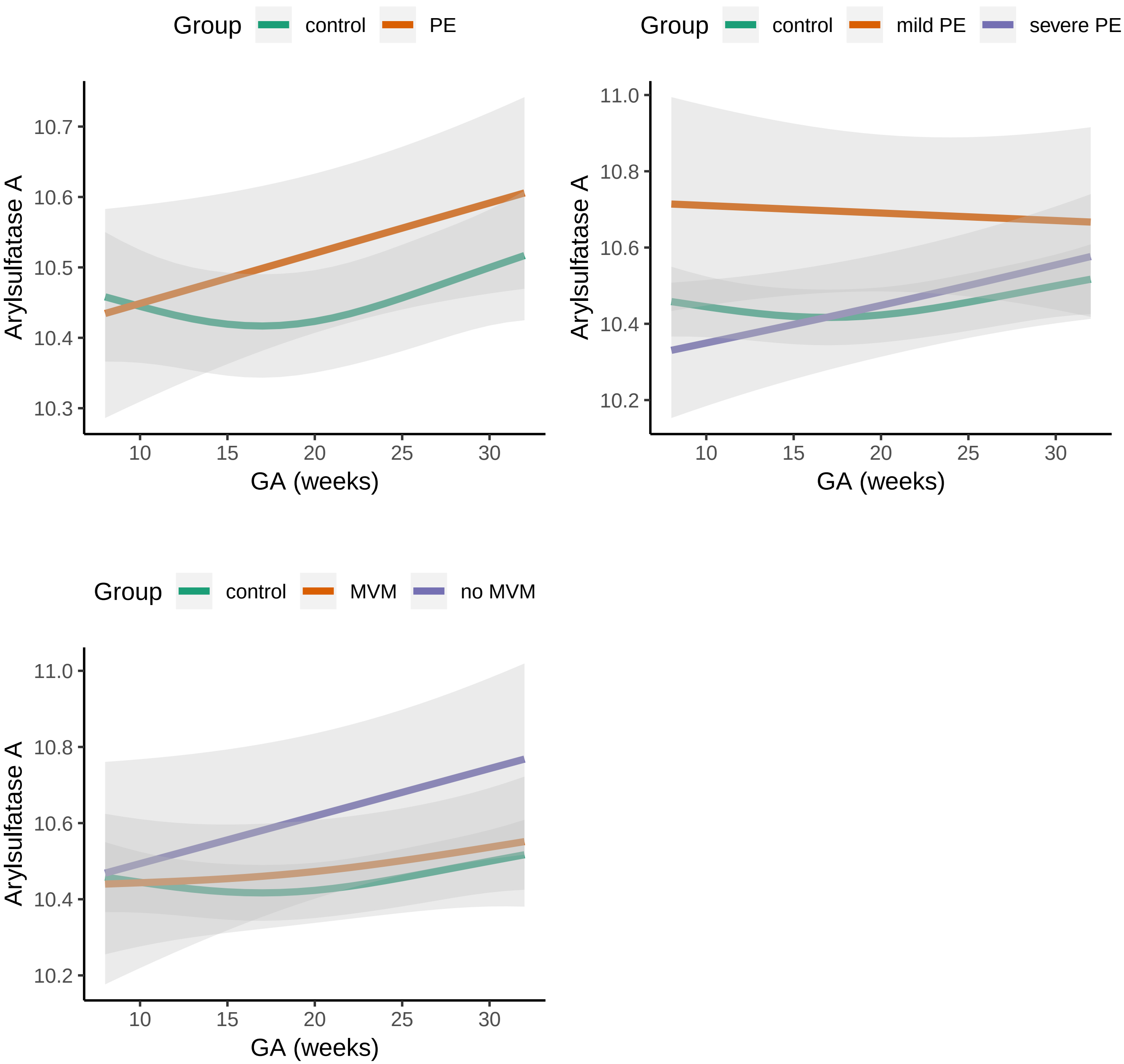

Group
control
PE
Arylsulfatase A
10.7
10.6
10.5
10.4
10.3
10
15
20
25
30
GA (weeks)
Group
control
mild PE
severe PE
Arylsulfatase A
11.0
10.8
10.6
10.4
10.2
10
15
20
25
30
GA (weeks)
Group
control
MVM
no MVM
Arylsulfatase A
11.0
10.8
10.6
10.4
10.2
10
15
20
25
30
GA (weeks)

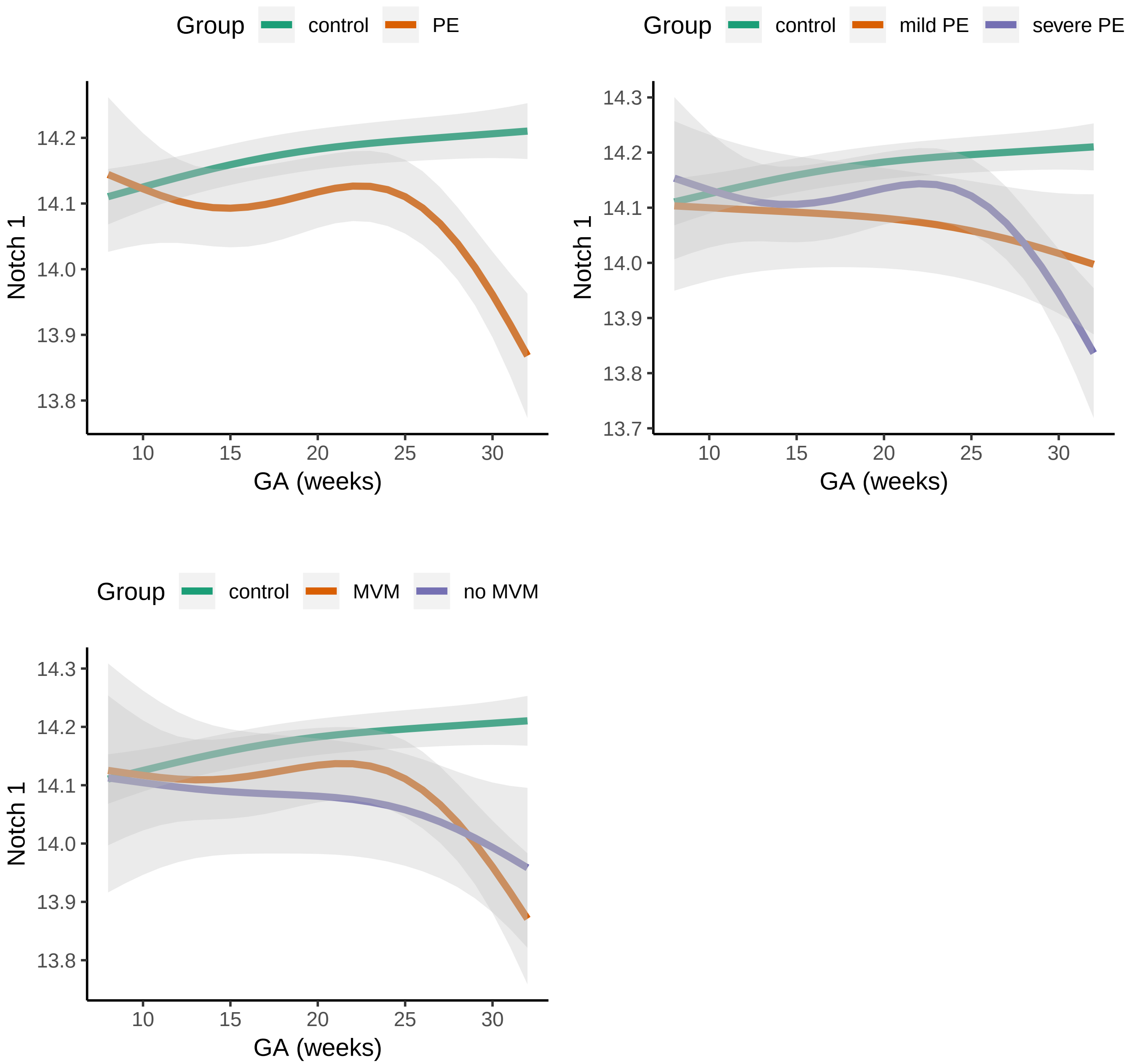

Group
control
PE
14.2
14.1
14.0
13.9
13.8
Notch 1
10
15
20
25
30
GA (weeks)
Group
control
mild PE
severe PE
14.3
14.2
14.1
14.0
13.9
13.8
13.7
Notch 1
10
15
20
25
30
GA (weeks)
Group
control
MVM
no MVM
14.3
14.2
14.1
14.0
13.9
13.8
Notch 1
10
15
20
25
30
GA (weeks)

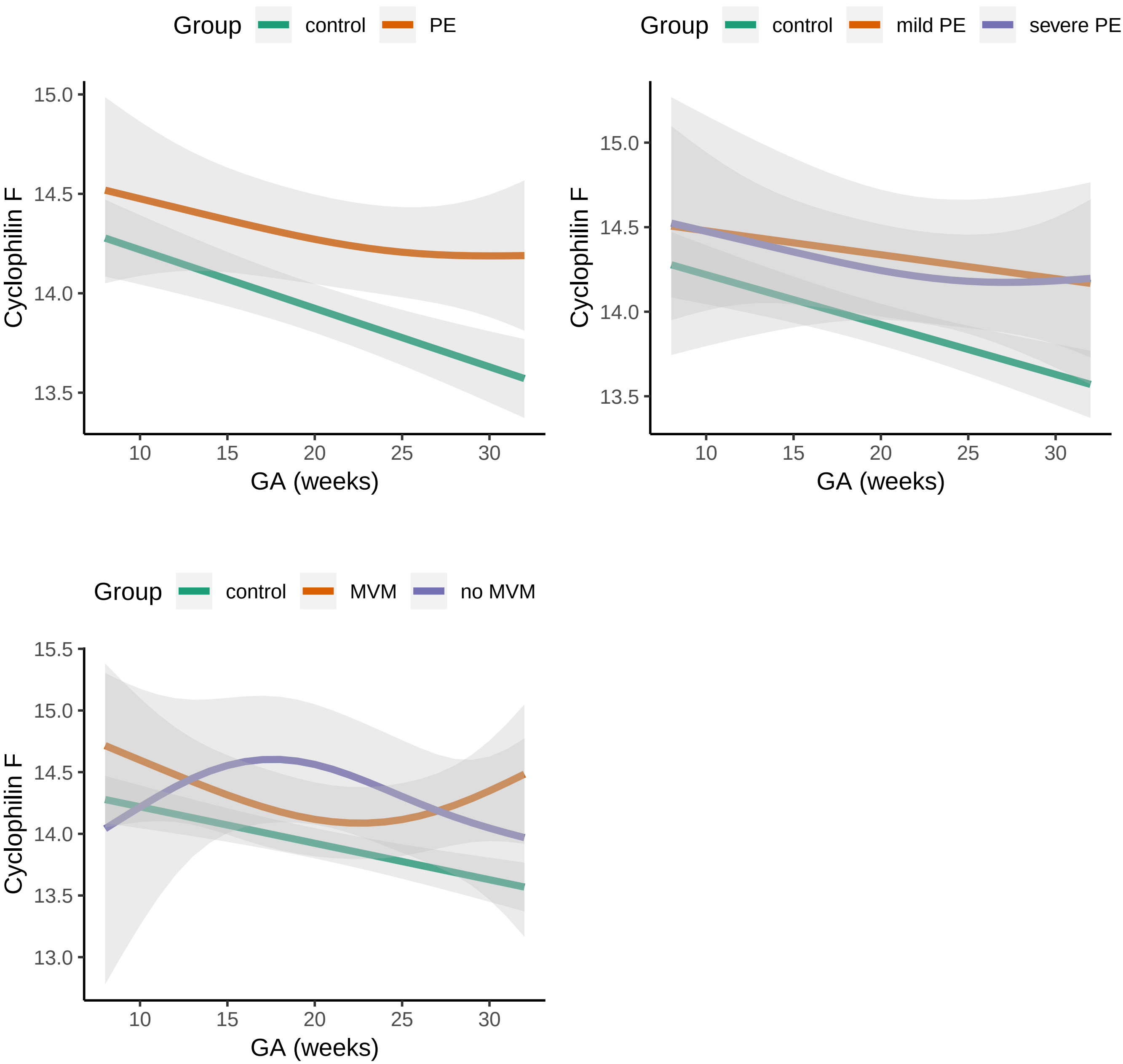
Group
control
PE
Cyclophilin F
15.0
14.5
14.0
13.5
10
15
20
25
30
GA (weeks)
Group
control
mild PE
severe PE
Cyclophilin F
15.0
14.5
14.0
13.5
10
15
20
25
30
GA (weeks)
Group
control
MVM
no MVM
Cyclophilin F
15.5
15.0
14.5
14.0
13.5
13.0
10
15
20
25
30
GA (weeks)

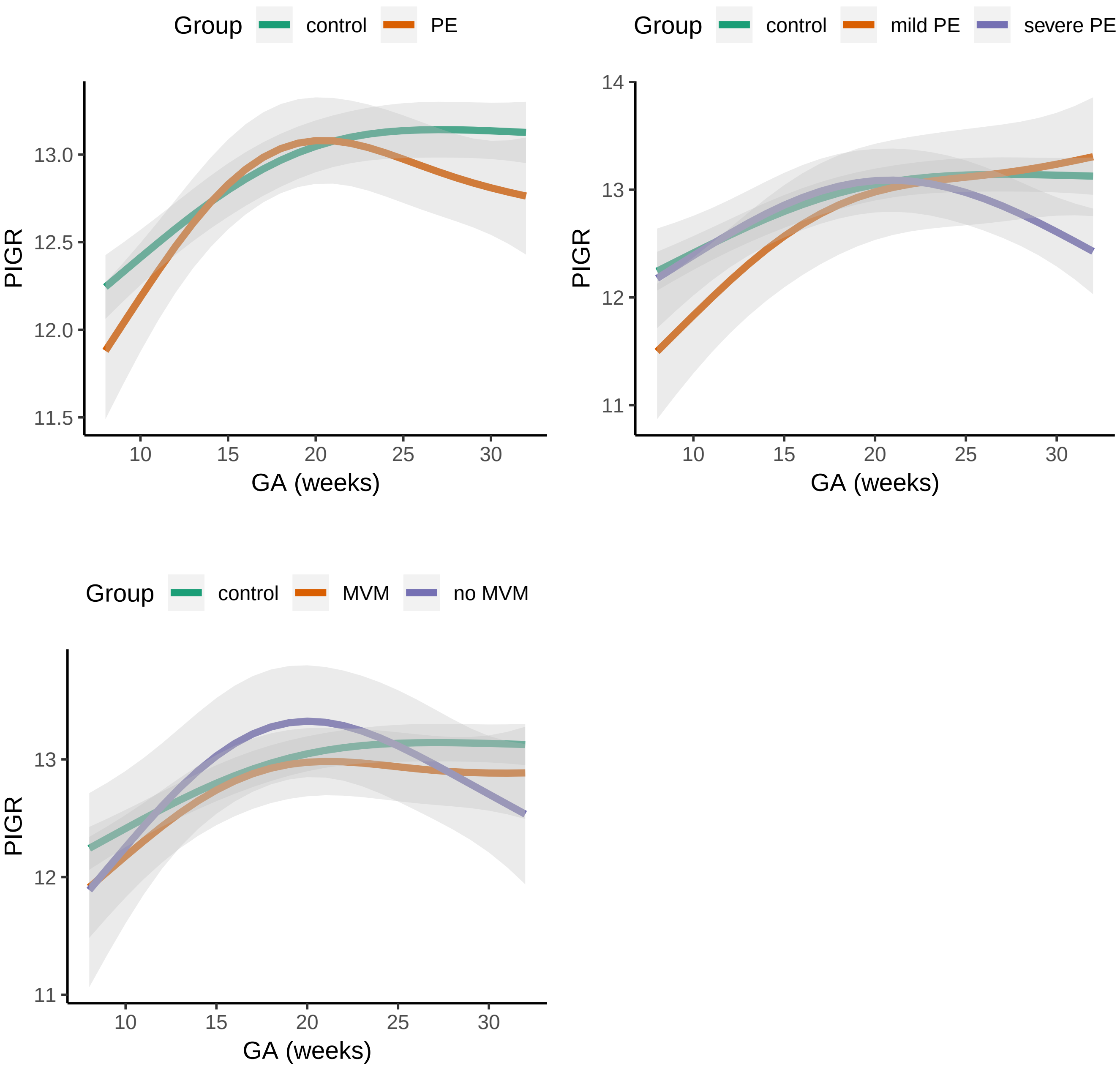
Group
control
PE
PIGR
13.0
12.5
12.0
11.5
10
15
20
25
30
GA (weeks)
Group
control
mild PE
severe PE
PIGR
14
13
12
11
10
15
20
25
30
GA (weeks)
Group
control
MVM
no MVM
PIGR
13
12
11
10
15
20
25
30
GA (weeks)

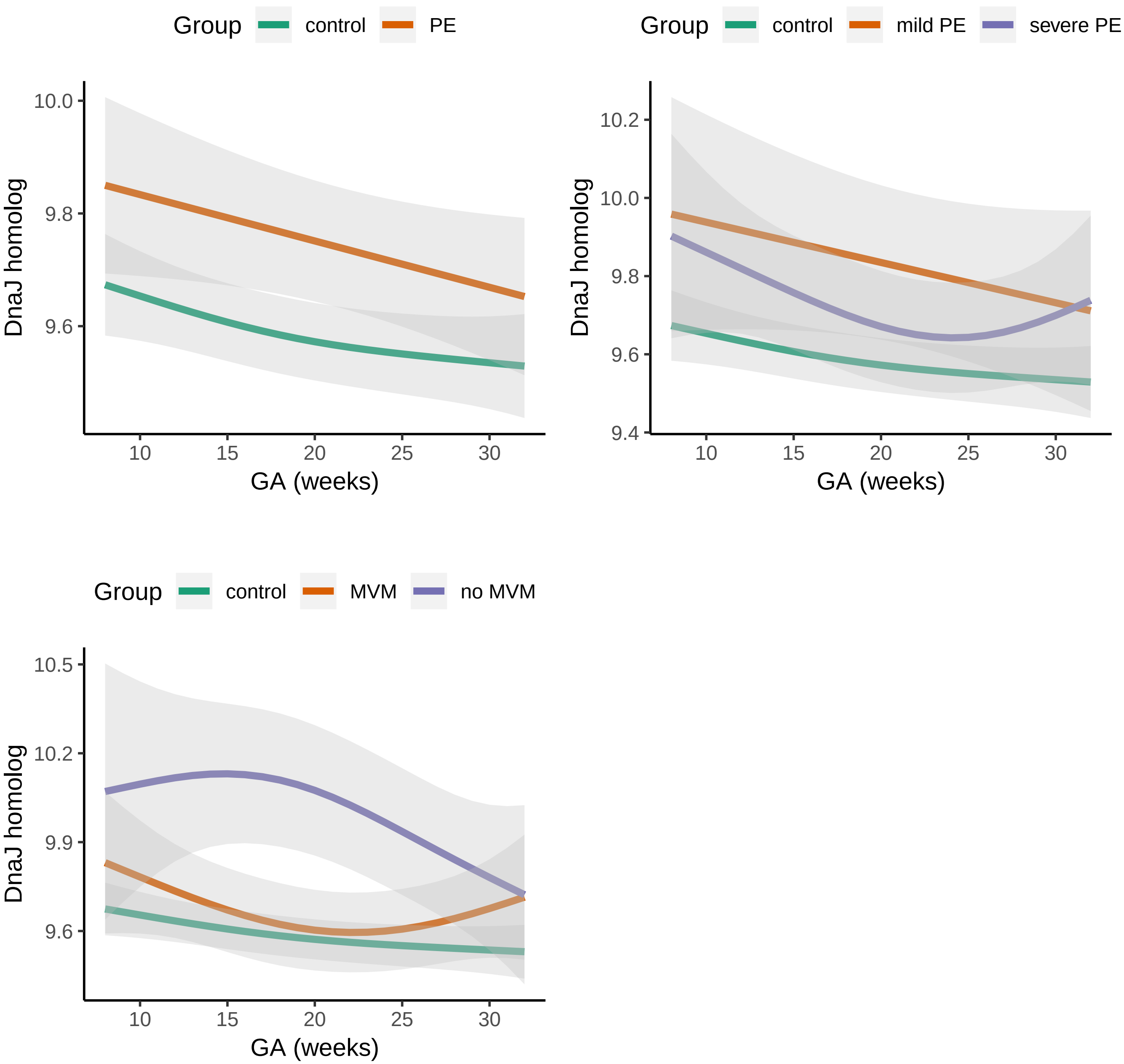

Group
control
PE
DnaJ homolog
10.0
9.8
9.6
10
15
20
25
30
GA (weeks)
Group
control
mild PE
severe PE
DnaJ homolog
10.2
10.0
9.8
9.6
9.4
10
15
20
25
30
GA (weeks)
Group
control
MVM
no MVM
DnaJ homolog
10.5
10.2
9.9
9.6
10
15
20
25
30
GA (weeks)

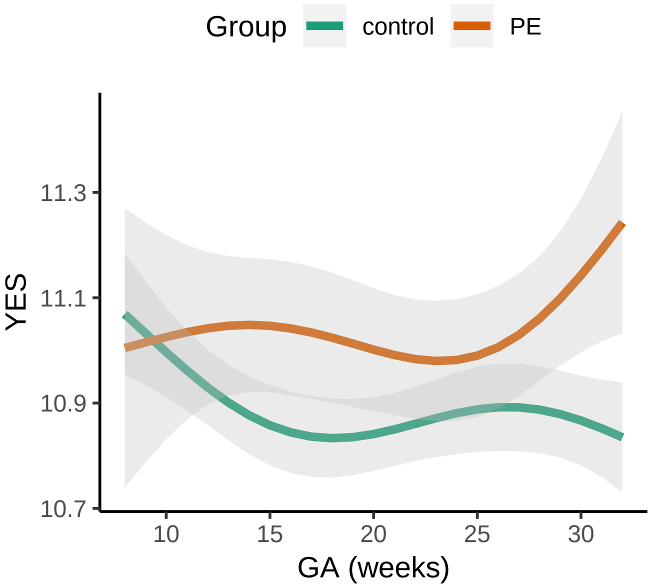
Group
control
PE
11.3
11.1
10.9
10.7
YES
10
15
20
25
30
GA (weeks)

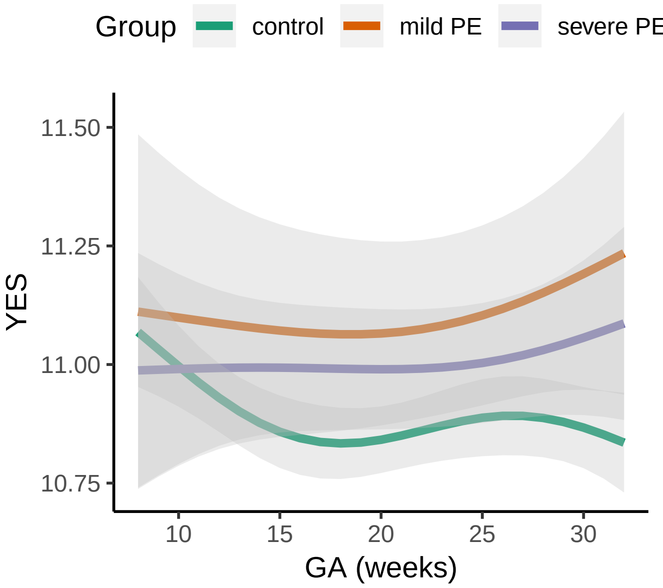
Group
control
mild PE
severe PE
11.50
11.25
11.00
10.75
YES
10
15
20
25
30
GA (weeks)

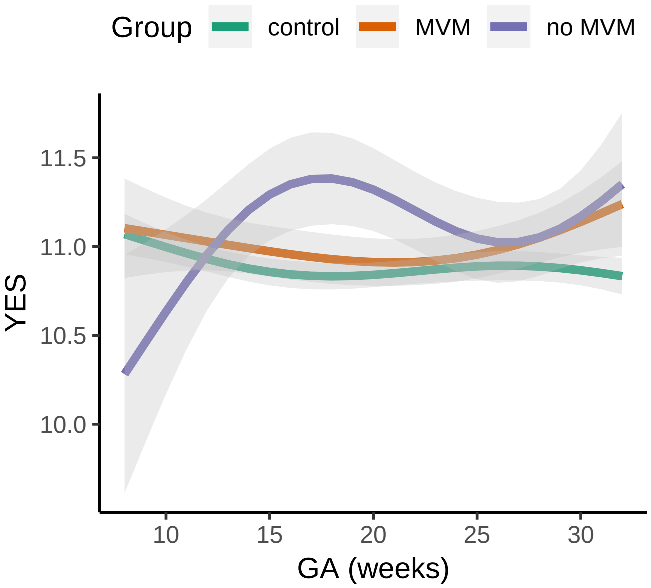
Group
control
MVM
no MVM
11.5
11.0
10.5
10.0
YES
10
15
20
25
30
GA (weeks)

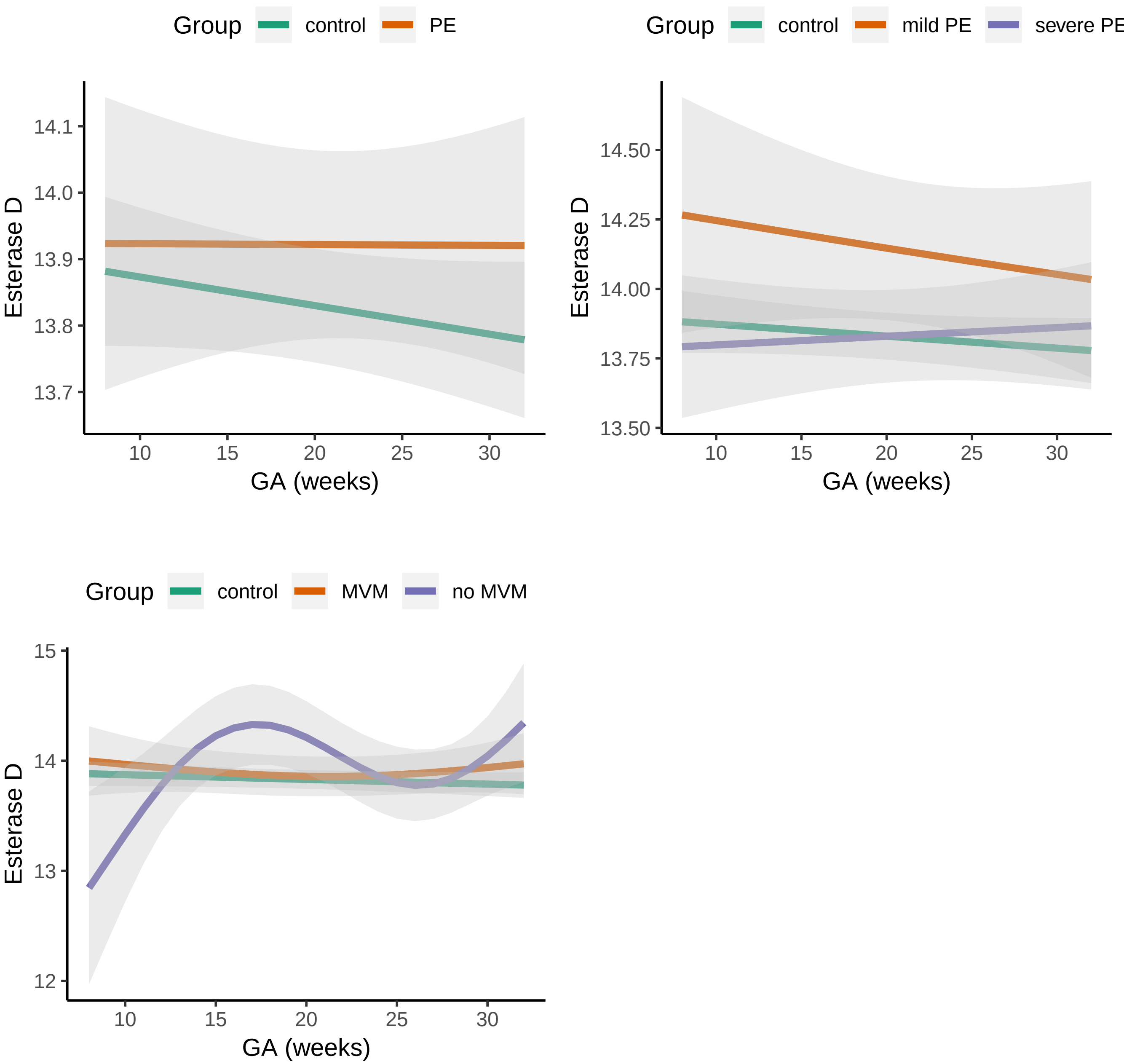
Group
control
PE
Esterase D
14.1
14.0
13.9
13.8
13.7
10
15
20
25
30
GA (weeks)
Group
control
mild PE
severe PE
Esterase D
14.50
14.25
14.00
13.75
13.50
10
15
20
25
30
GA (weeks)
Group
control
MVM
no MVM
Esterase D
15
14
13
12
10
15
20
25
30
GA (weeks)

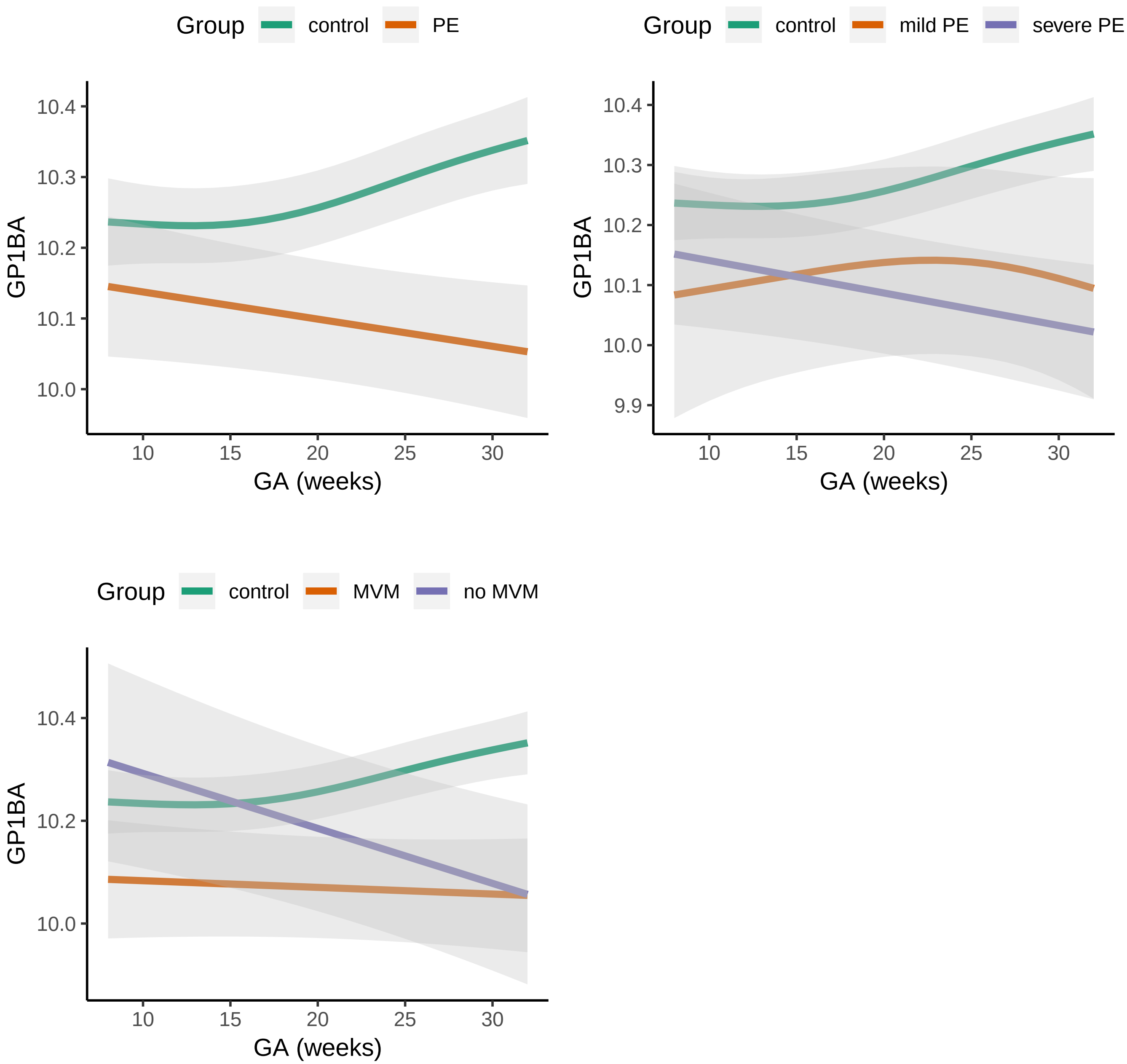
Group
control
PE
10.4
10.3
10.2
10.1
10.0
GP1BA
10
15
20
25
30
GA (weeks)
Group
control
mild PE
severe PE
10.4
10.3
10.2
10.1
10.0
9.9
GP1BA
10
15
20
25
30
GA (weeks)
Group
control
MVM
no MVM
10.4
10.2
10.0
GP1BA
10
15
20
25
30
GA (weeks)

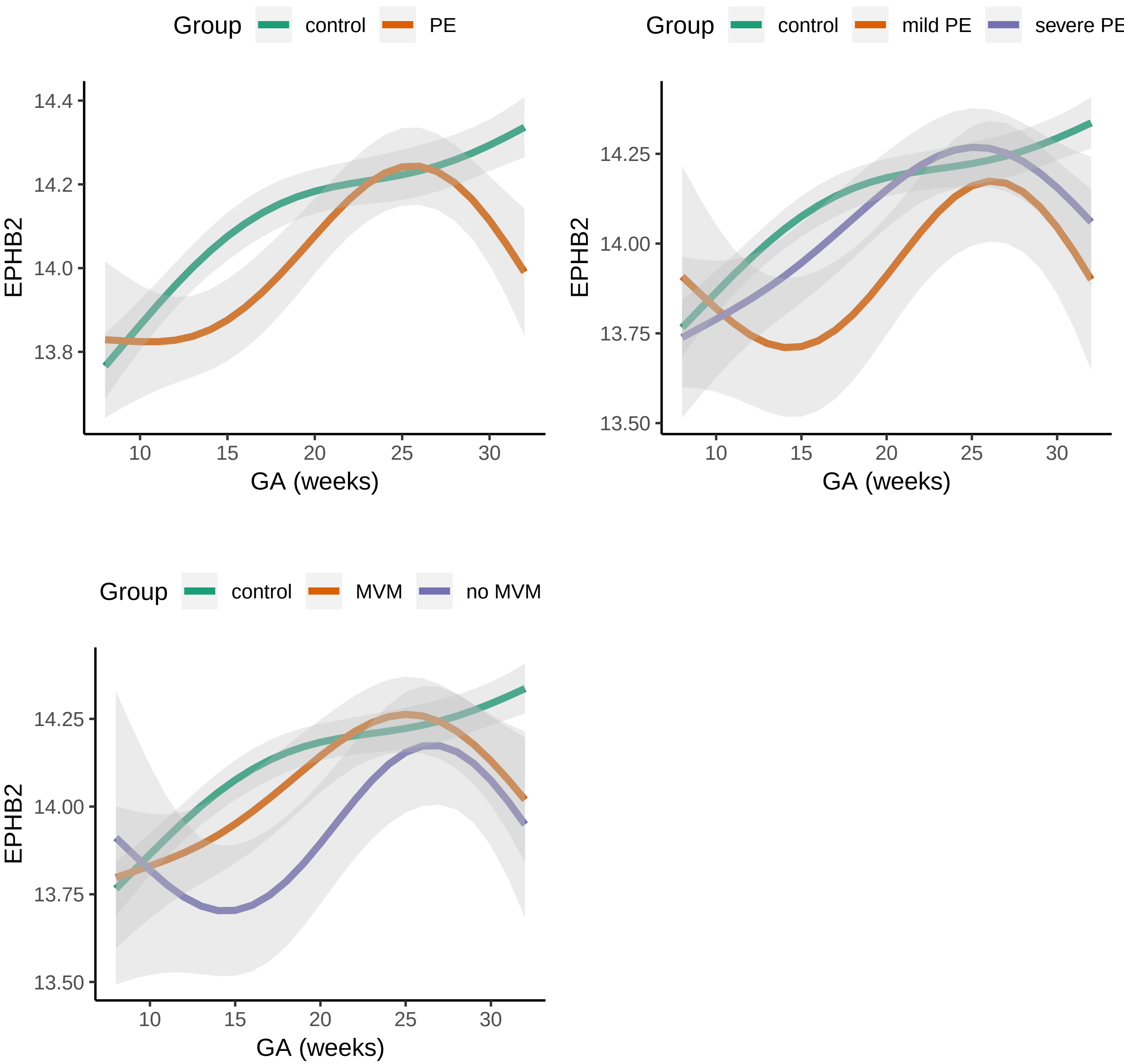
Group
control
PE
EPHB2
14.4
14.2
14.0
13.8
10
15
20
25
30
GA (weeks)
Group
control
mild PE
severe PE
EPHB2
14.25
14.00
13.75
13.50
10
15
20
25
30
GA (weeks)
Group
control
MVM
no MVM
EPHB2
14.25
14.00
13.75
13.50
10
15
20
25
30
GA (weeks)

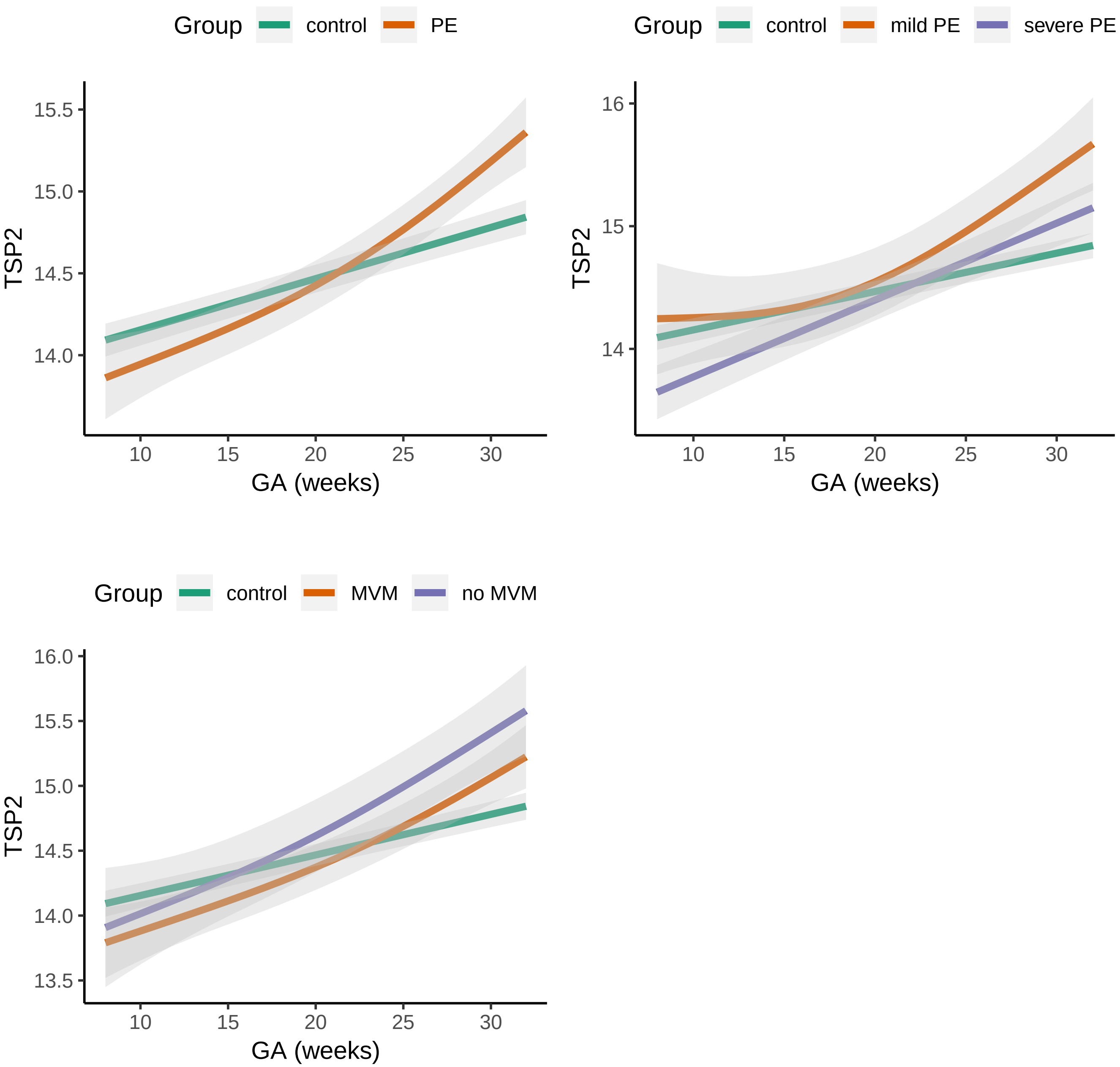
Group
control
PE
TSP2
15.5
15.0
14.5
14.0
10
15
20
25
30
GA (weeks)
Group
control
mild PE
severe PE
TSP2
16
15
14
10
15
20
25
30
GA (weeks)
Group
control
MVM
no MVM
TSP2
16.0
15.5
15.0
14.5
14.0
13.5
10
15
20
25
30
GA (weeks)

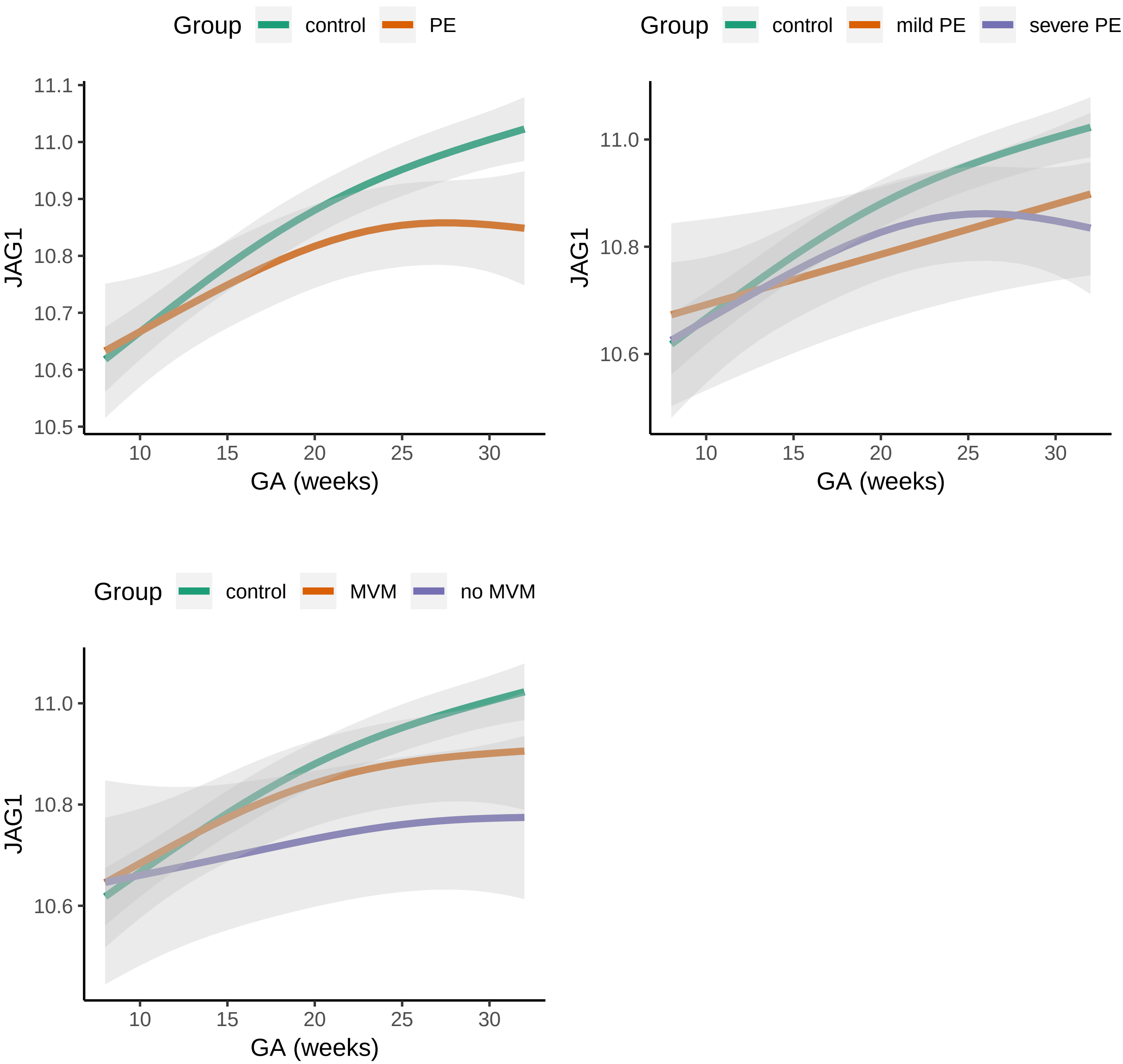
Group
control
PE
JAG1
11.1
11.0
10.9
10.8
10.7
10.6
10.5
10
15
20
25
30
GA (weeks)
Group
control
mild PE
severe PE
JAG1
11.0
10.8
10.6
10
15
20
25
30
GA (weeks)
Group
control
MVM
no MVM
JAG1
11.0
10.8
10.6
10
15
20
25
30
GA (weeks)

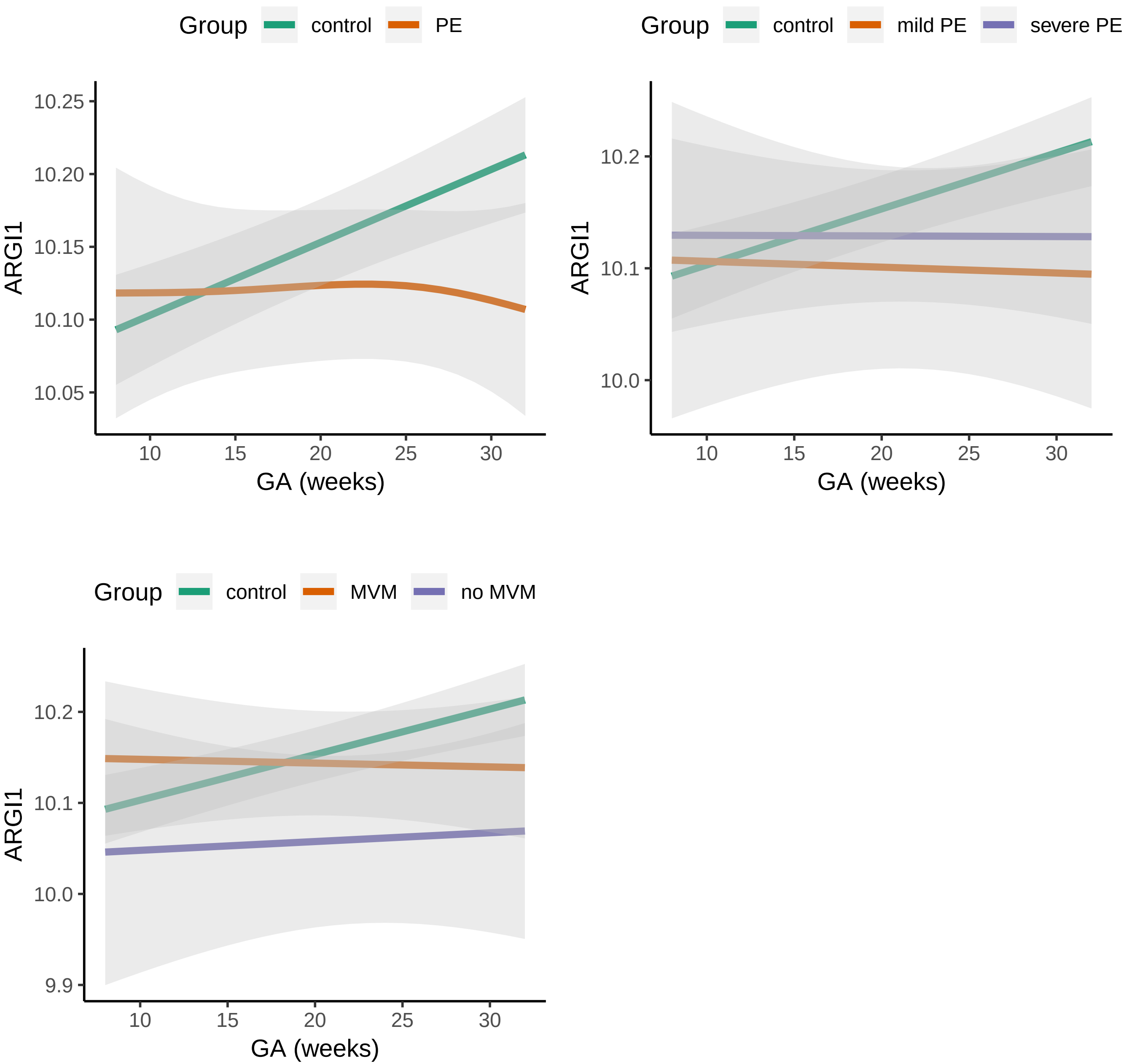
Group
control
PE
ARGI1
10.25
10.20
10.15
10.10
10.05
10
15
20
25
30
GA (weeks)
Group
control
mild PE
severe PE
ARGI1
10.2
10.1
10.0
10
15
20
25
30
GA (weeks)
Group
control
MVM
no MVM
ARGI1
10.2
10.1
10.0
9.9
10
15
20
25
30
GA (weeks)

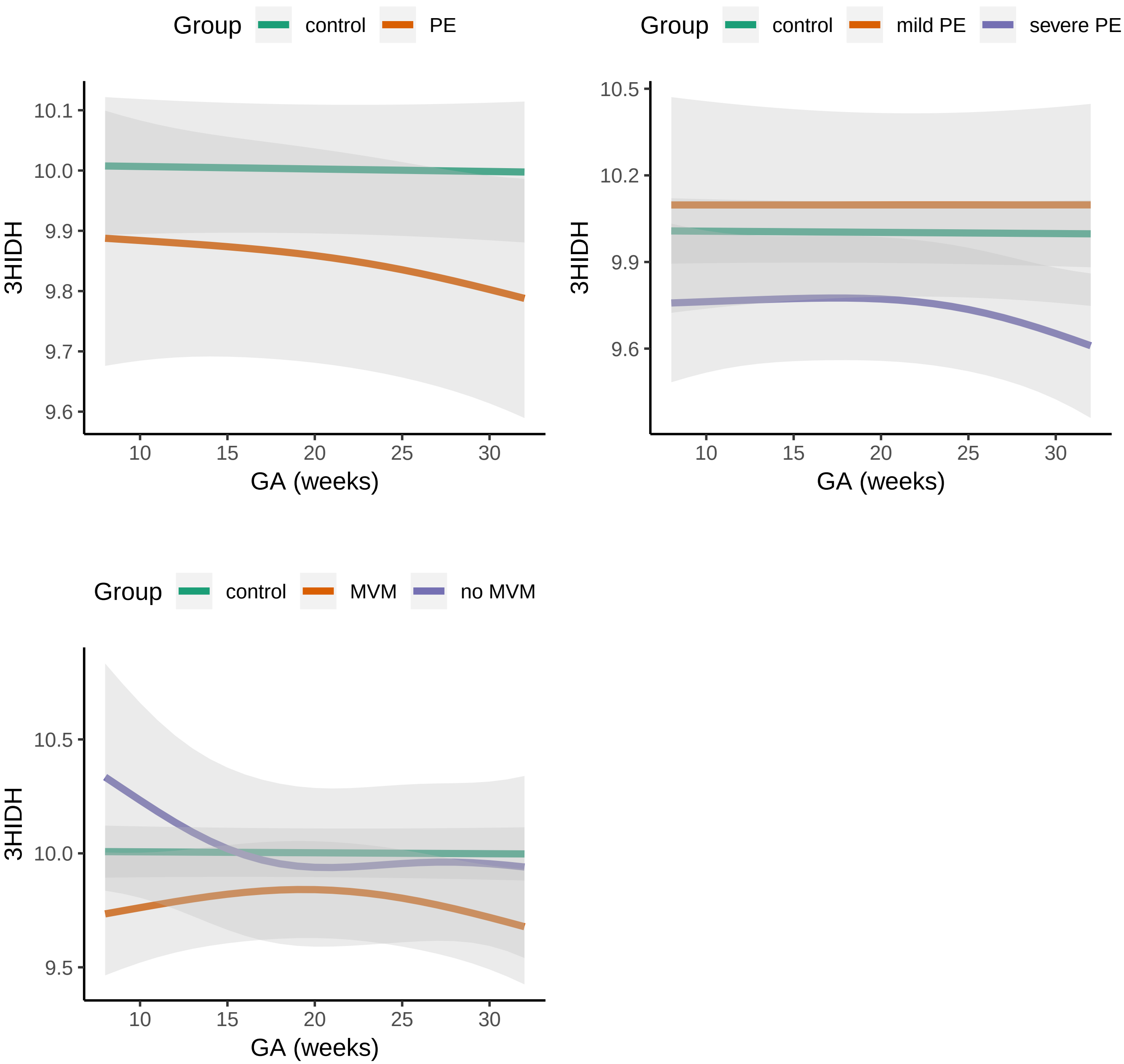
Group
control
PE
10.1
10.0
9.9
9.8
9.7
9.6
3HIDH
10
15
20
25
30
GA (weeks)
Group
control
mild PE
severe PE
10.5
10.2
9.9
9.6
3HIDH
10
15
20
25
30
GA (weeks)
Group
control
MVM
no MVM
10.5
10.0
9.5
3HIDH
10
15
20
25
30
GA (weeks)

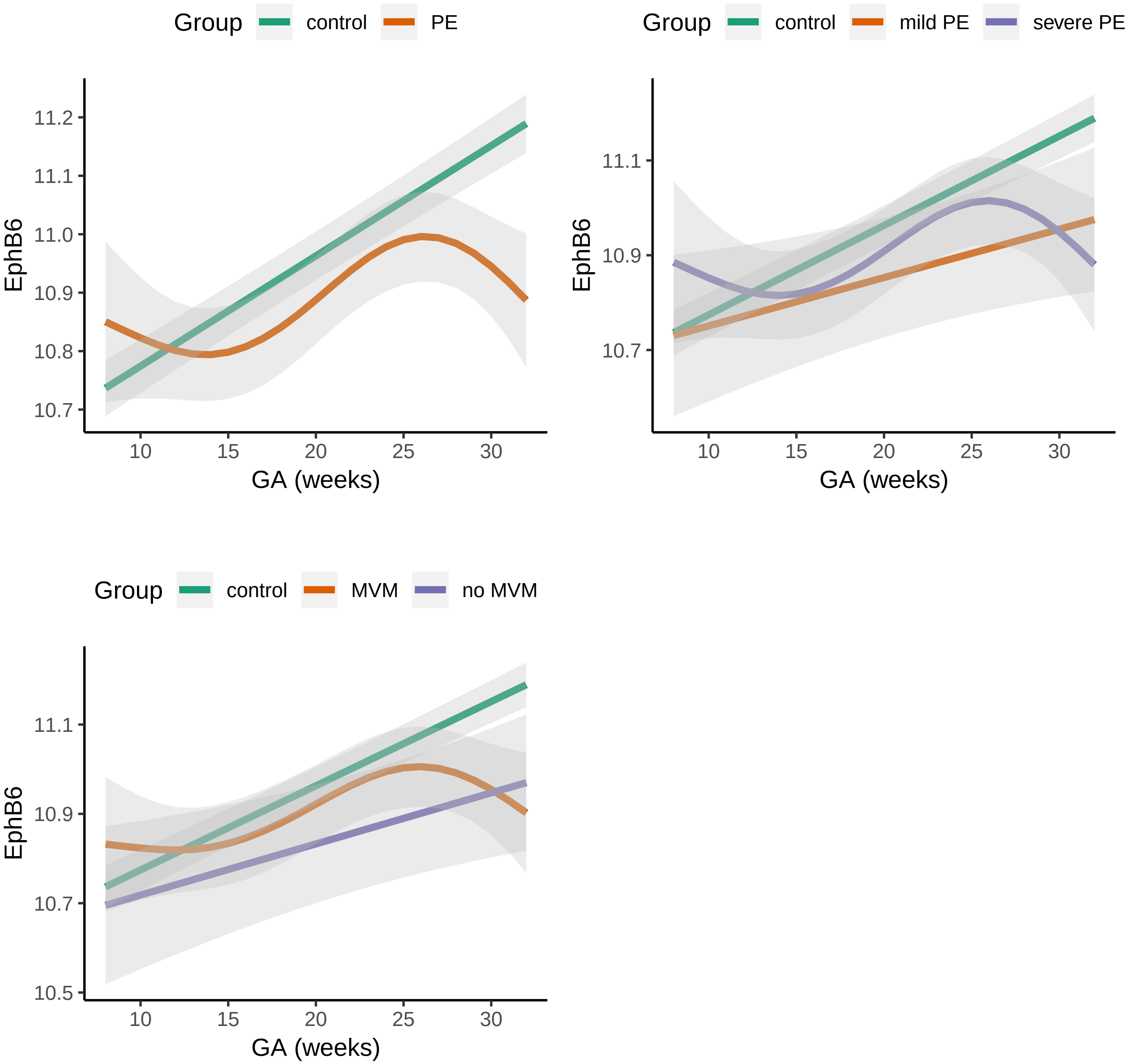

Group
control
PE
EphB6
11.2
11.1
11.0
10.9
10.8
10.7
10
15
20
25
30
GA (weeks)
Group
control
mild PE
severe PE
EphB6
11.1
10.9
10.7
10
15
20
25
30
GA (weeks)
Group
control
MVM
no MVM
EphB6
11.1
10.9
10.7
10.5
10
15
20
25
30
GA (weeks)

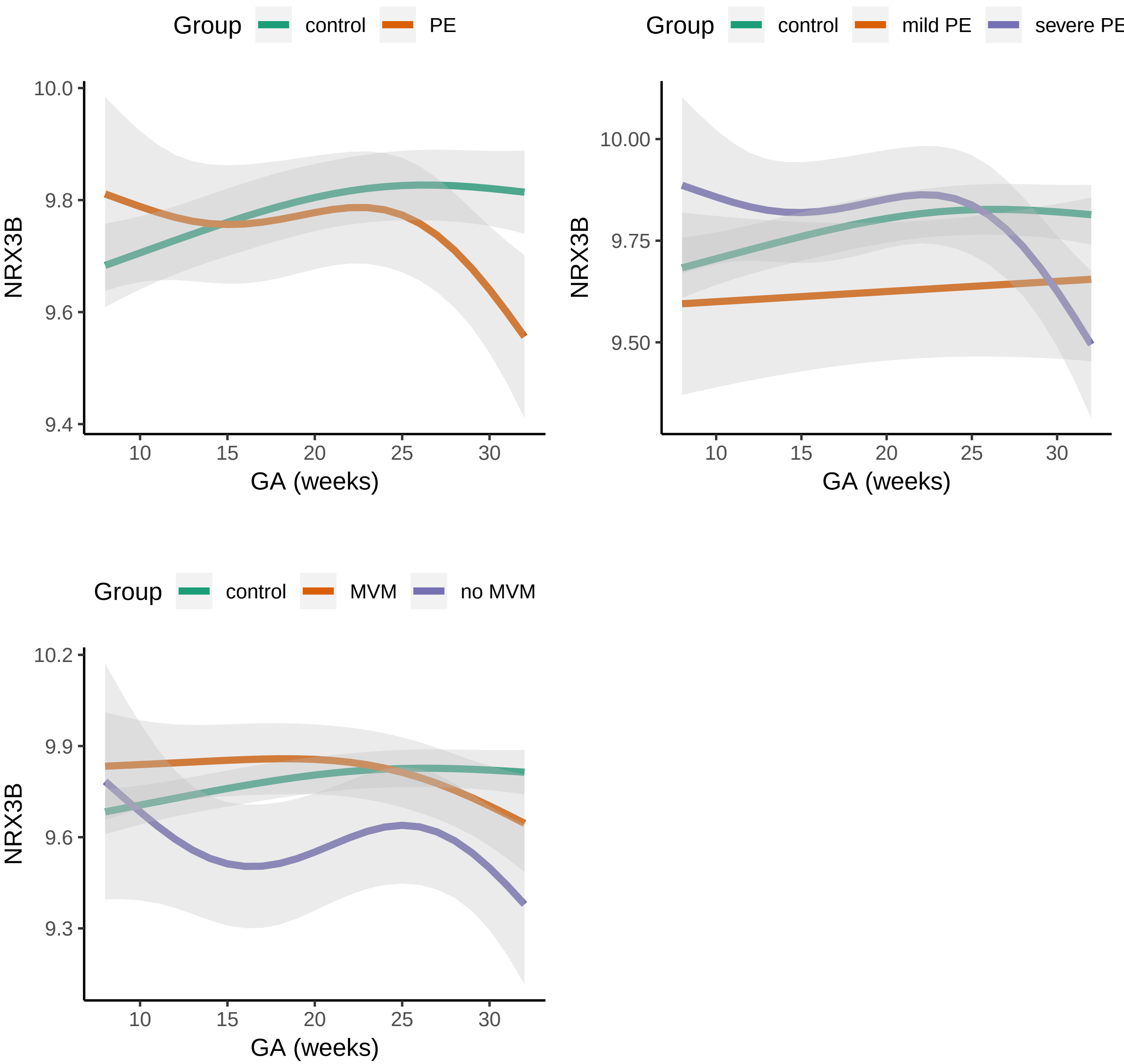

Group
control
PE
10.0
9.8
9.6
9.4
NRX3B
10
15
20
25
30
GA (weeks)
Group
control
mild PE
severe PE
10.00
9.75
9.50
NRX3B
10
15
20
25
30
GA (weeks)
Group
control
MVM
no MVM
10.2
9.9
9.6
9.3
NRX3B
10
15
20
25
30
GA (weeks)

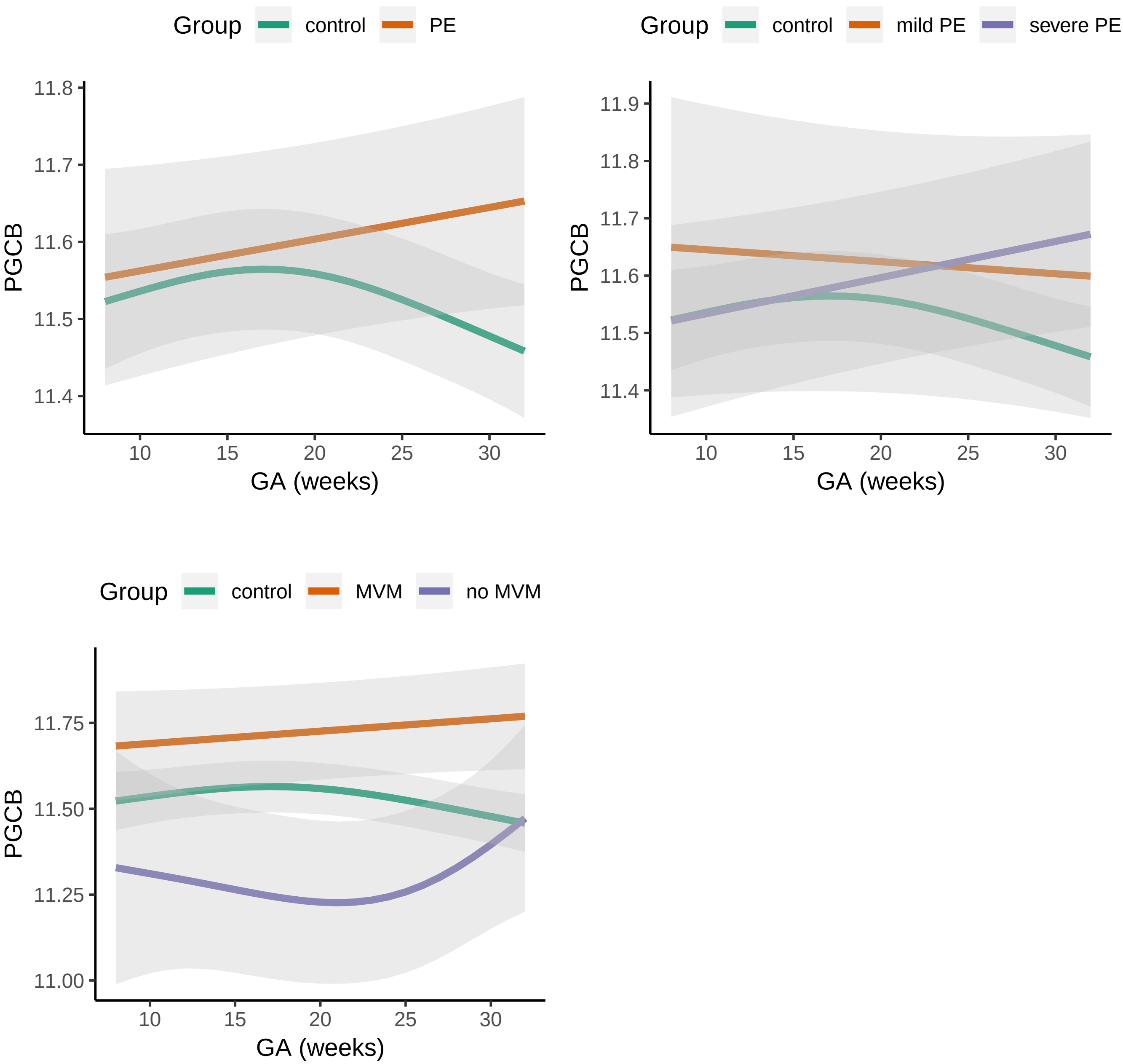

Group
control
PE
PGCB
11.8
11.7
11.6
11.5
11.4
10
15
20
25
30
GA (weeks)
Group
control
mild PE
severe PE
PGCB
11.9
11.8
11.7
11.6
11.5
11.4
10
15
20
25
30
GA (weeks)
Group
control
MVM
no MVM
PGCB
11.75
11.50
11.25
11.00
10
15
20
25
30
GA (weeks)

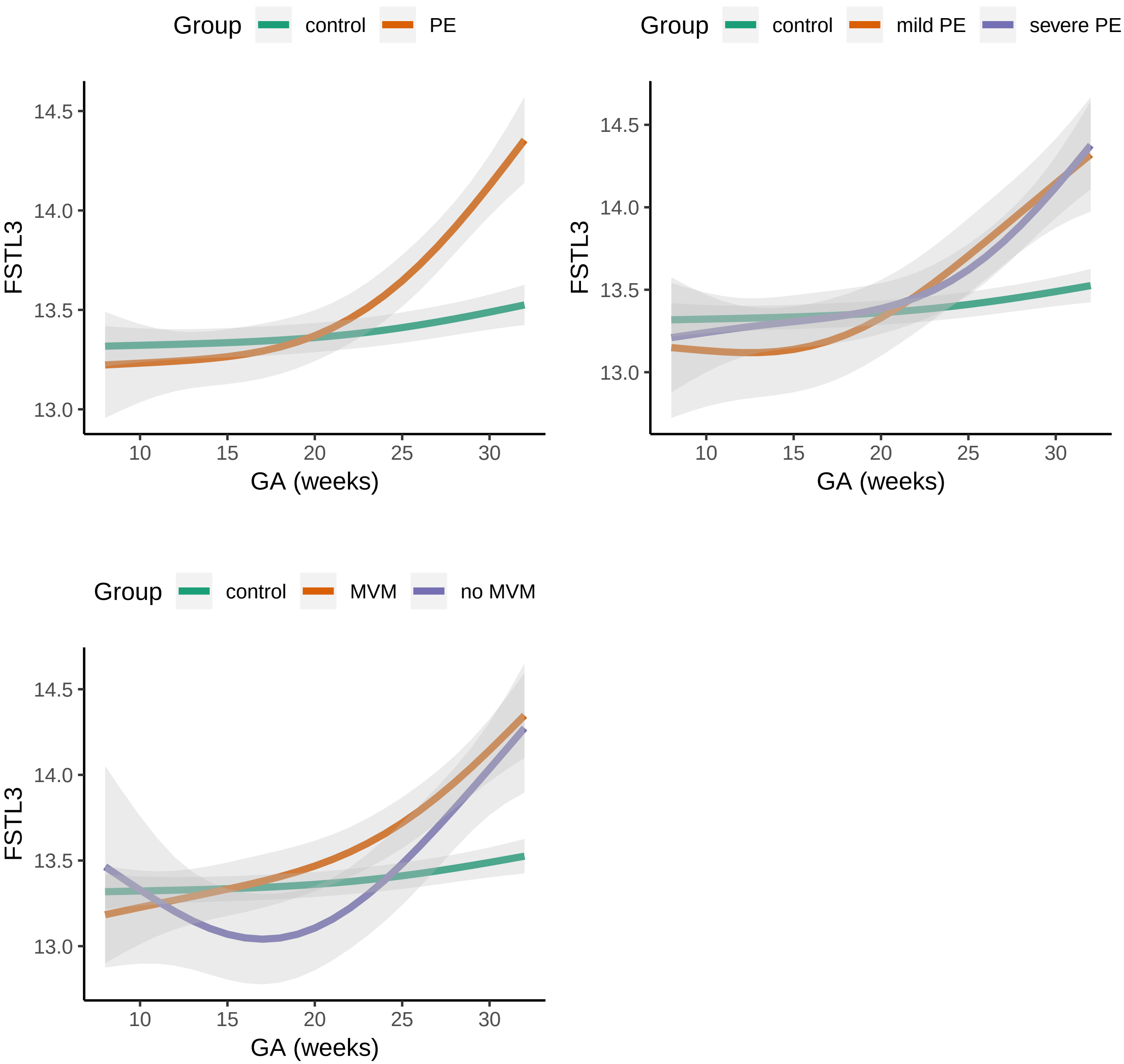
Group
control
PE
FSTL3
GA (weeks)
Group
control
mild PE
severe PE
FSTL3
GA (weeks)
Group
control
MVM
no MVM
FSTL3
GA (weeks)

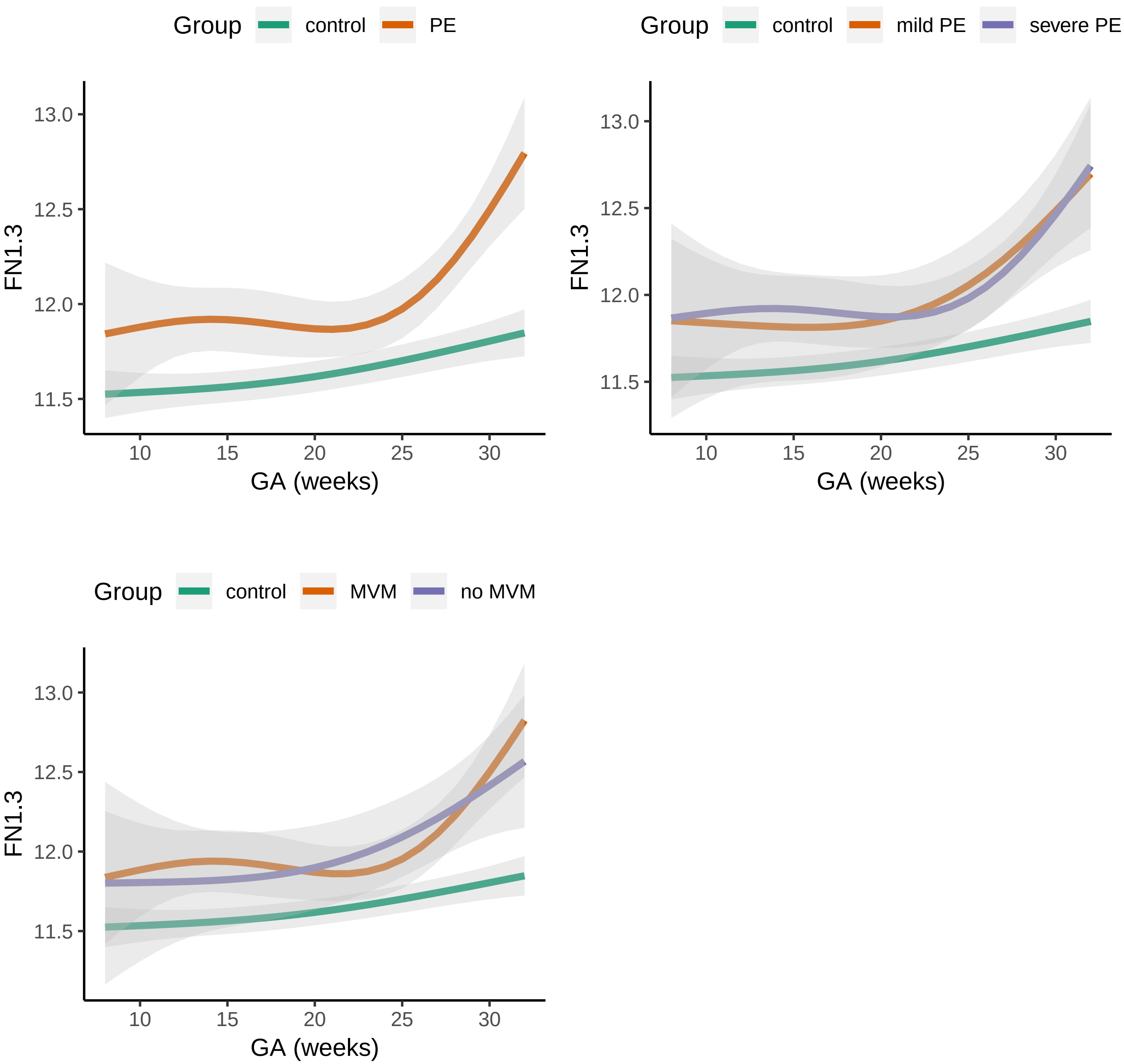
Group
control
PE
13.0
12.5
12.0
11.5
FN1.3
10
15
20
25
30
GA (weeks)
Group
control
mild PE
severe PE
13.0
12.5
12.0
11.5
FN1.3
10
15
20
25
30
GA (weeks)
Group
control
MVM
no MVM
13.0
12.5
12.0
11.5
FN1.3
10
15
20
25
30
GA (weeks)

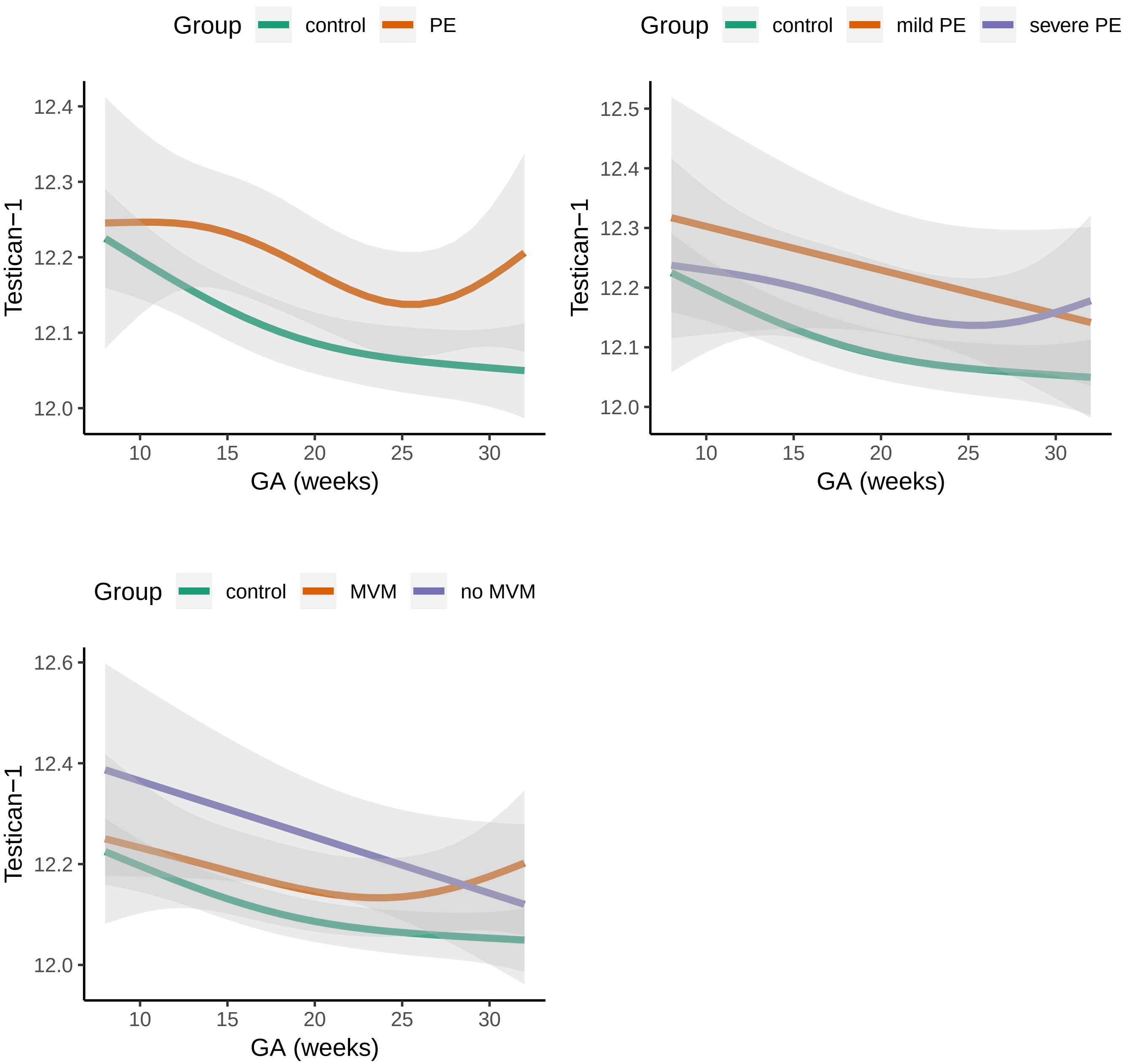
Group
control
PE
Testican−1
12.4
12.3
12.2
12.1
12.0
10
15
20
25
30
GA (weeks)
Group
control
mild PE
severe PE
Testican−1
12.5
12.4
12.3
12.2
12.1
12.0
10
15
20
25
30
GA (weeks)
Group
control
MVM
no MVM
Testican−1
12.6
12.4
12.2
12.0
10
15
20
25
30
GA (weeks)

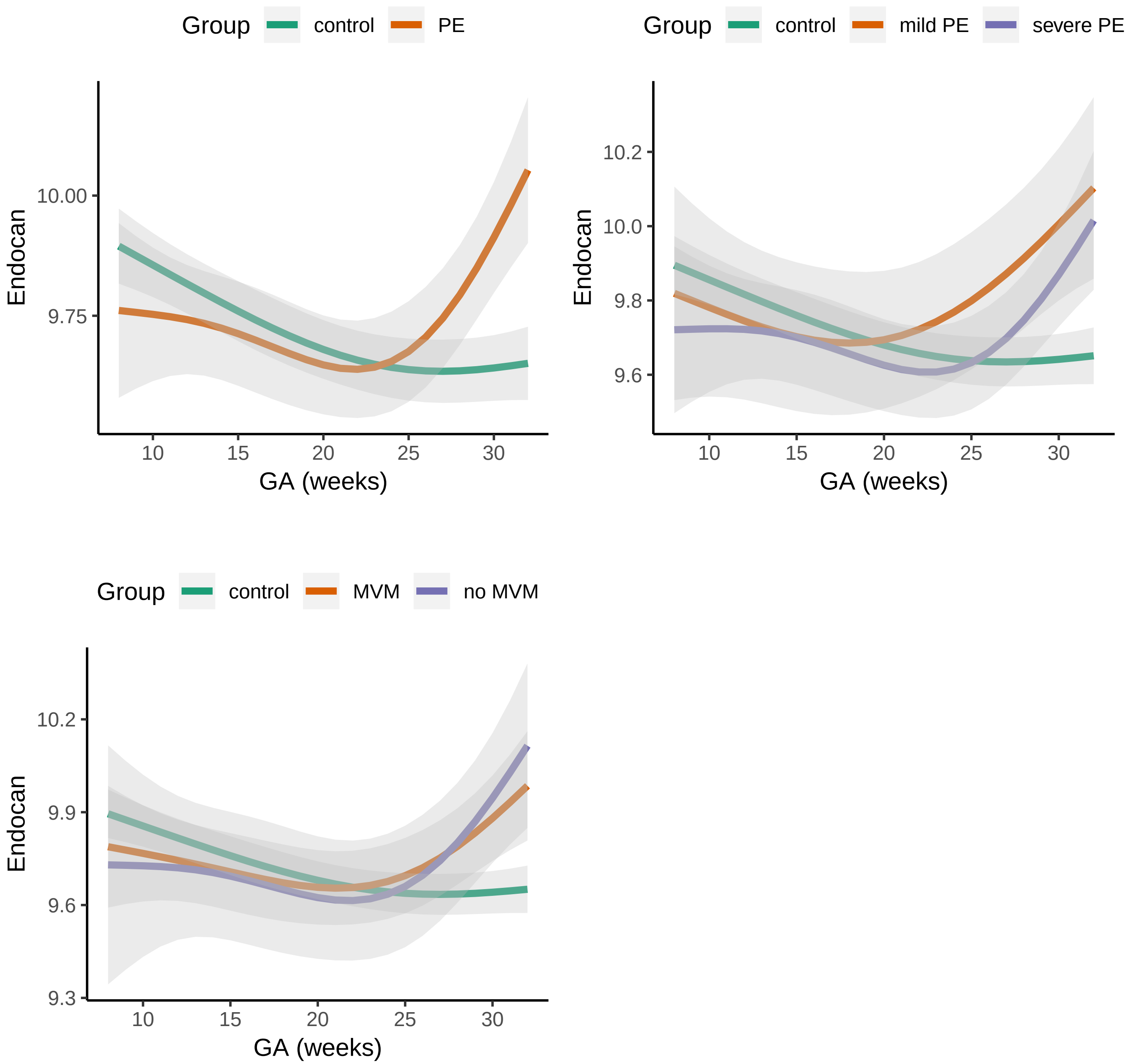
Group
control
PE
Endocan
10.00
9.75
10
15
20
25
30
GA (weeks)
Group
control
mild PE
severe PE
Endocan
10.2
10.0
9.8
9.6
10
15
20
25
30
GA (weeks)
Group
control
MVM
no MVM
Endocan
10.2
9.9
9.6
9.3
10
15
20
25
30
GA (weeks)

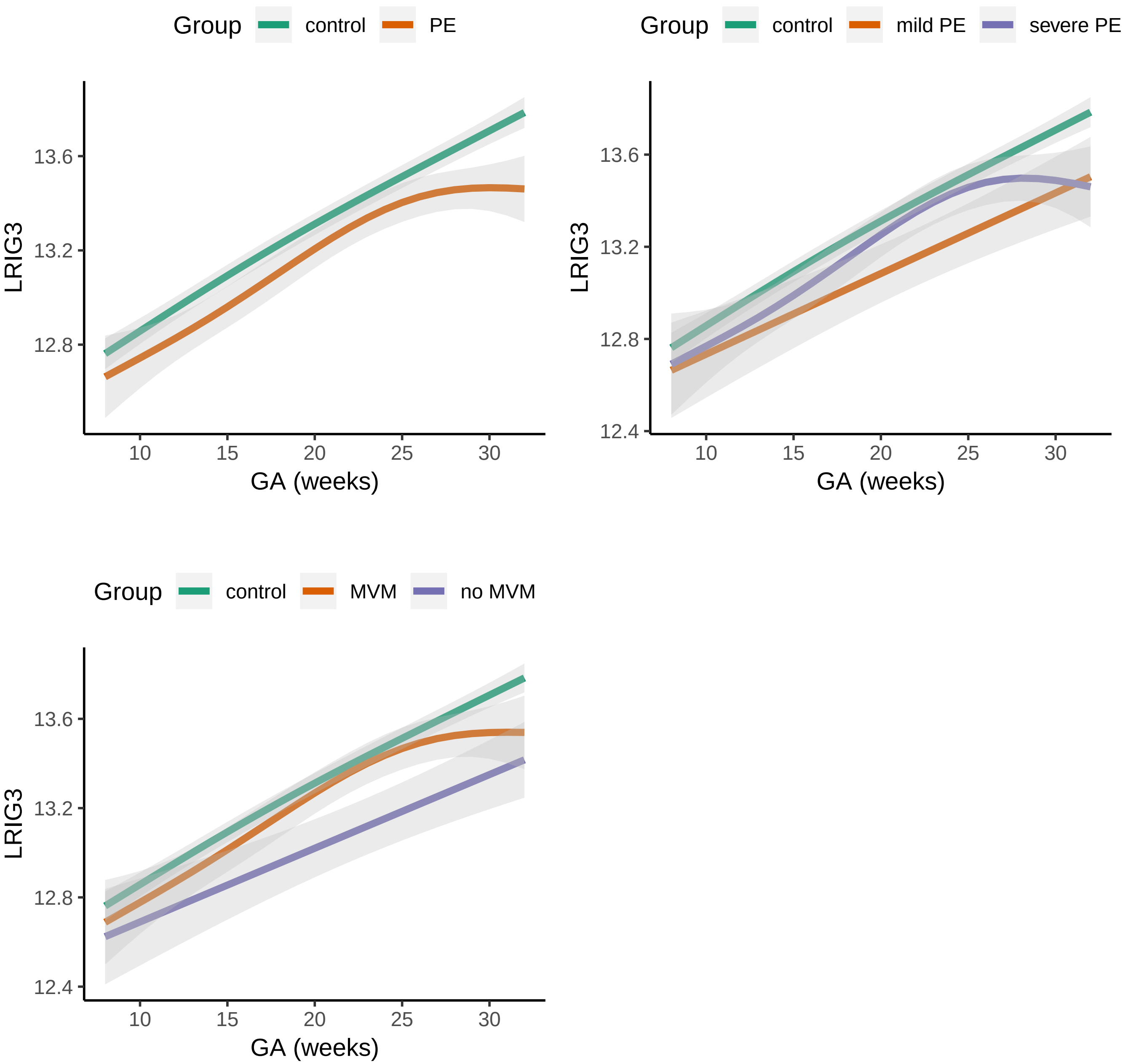
Group
control
PE
LRIG3
13.6
13.2
12.8
10
15
20
25
30
GA (weeks)
Group
control
mild PE
severe PE
LRIG3
13.6
13.2
12.8
12.4
10
15
20
25
30
GA (weeks)
Group
control
MVM
no MVM
LRIG3
13.6
13.2
12.8
12.4
10
15
20
25
30
GA (weeks)

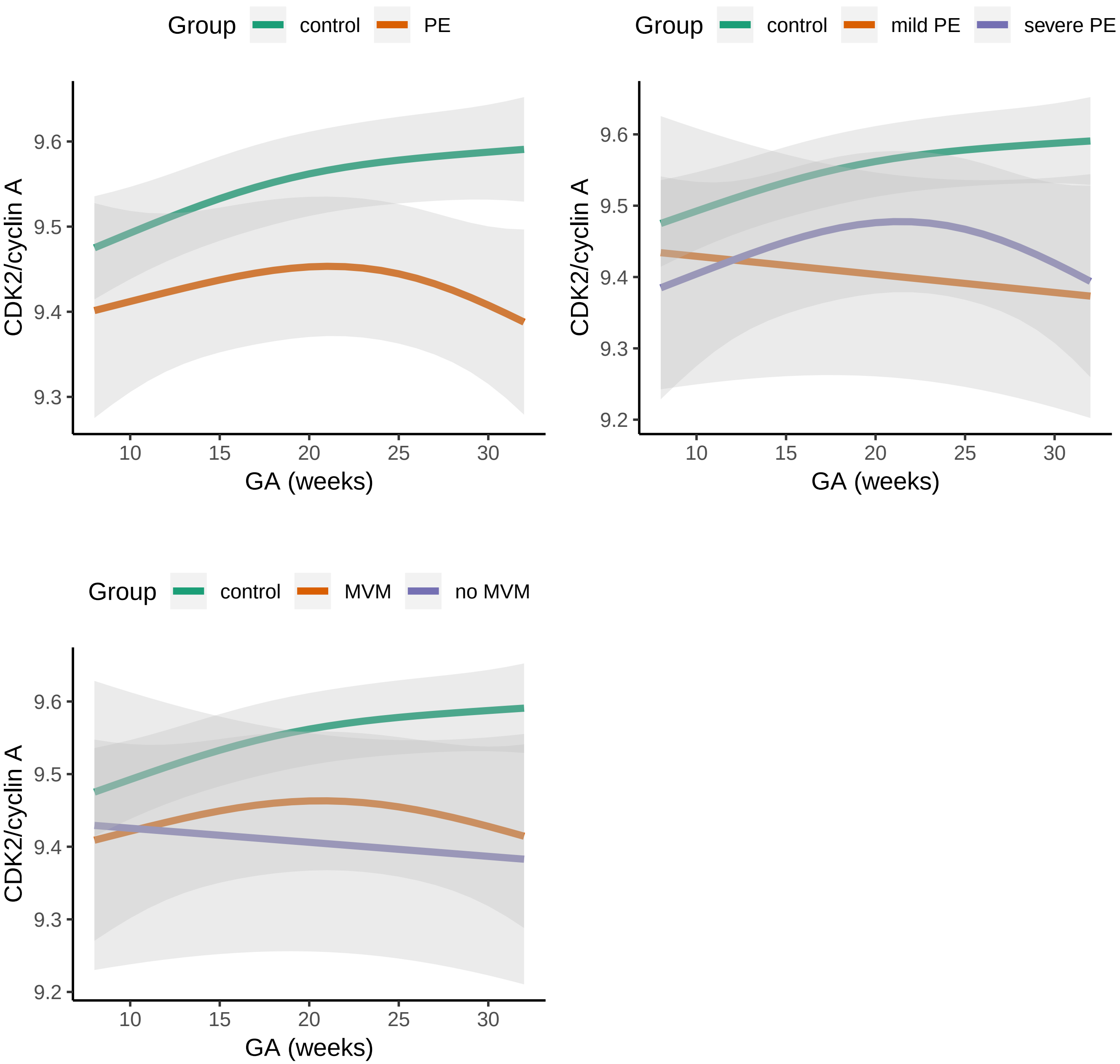

Group
control
PE
CDK2/cyclin A
9.3
9.4
9.5
9.6
10
15
20
25
30
GA (weeks)
Group
control
mild PE
severe PE
CDK2/cyclin A
9.2
9.3
9.4
9.5
9.6
10
15
20
25
30
GA (weeks)
Group
control
MVM
no MVM
CDK2/cyclin A
9.2
9.3
9.4
9.5
9.6
10
15
20
25
30
GA (weeks)

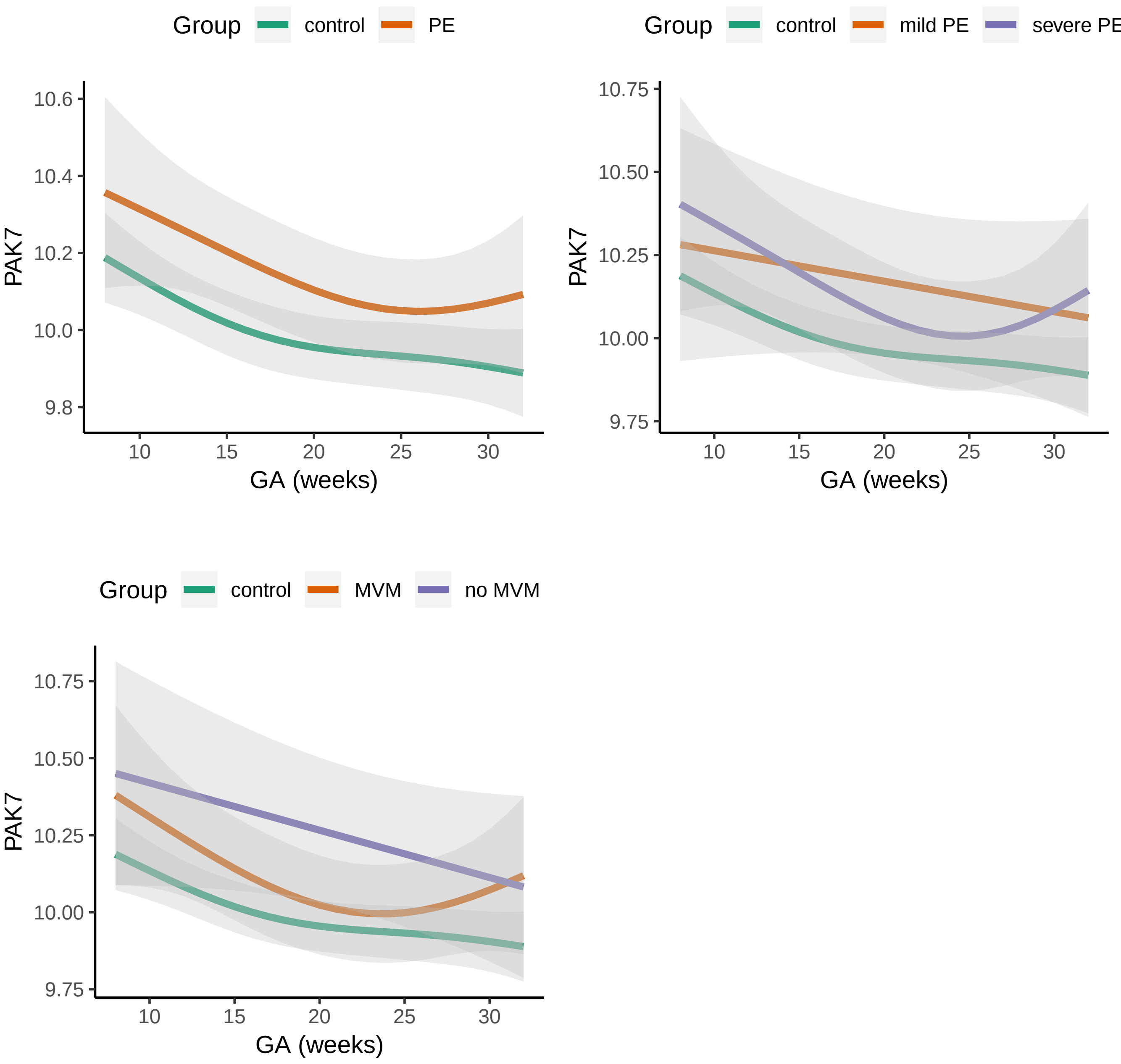

Group
control
PE
10.6
10.4
10.2
10.0
9.8
PAK7
10
15
20
25
30
GA (weeks)
Group
control
mild PE
severe PE
10.75
10.50
10.25
10.00
9.75
PAK7
10
15
20
25
30
GA (weeks)
Group
control
MVM
no MVM
10.75
10.50
10.25
10.00
9.75
PAK7
10
15
20
25
30
GA (weeks)

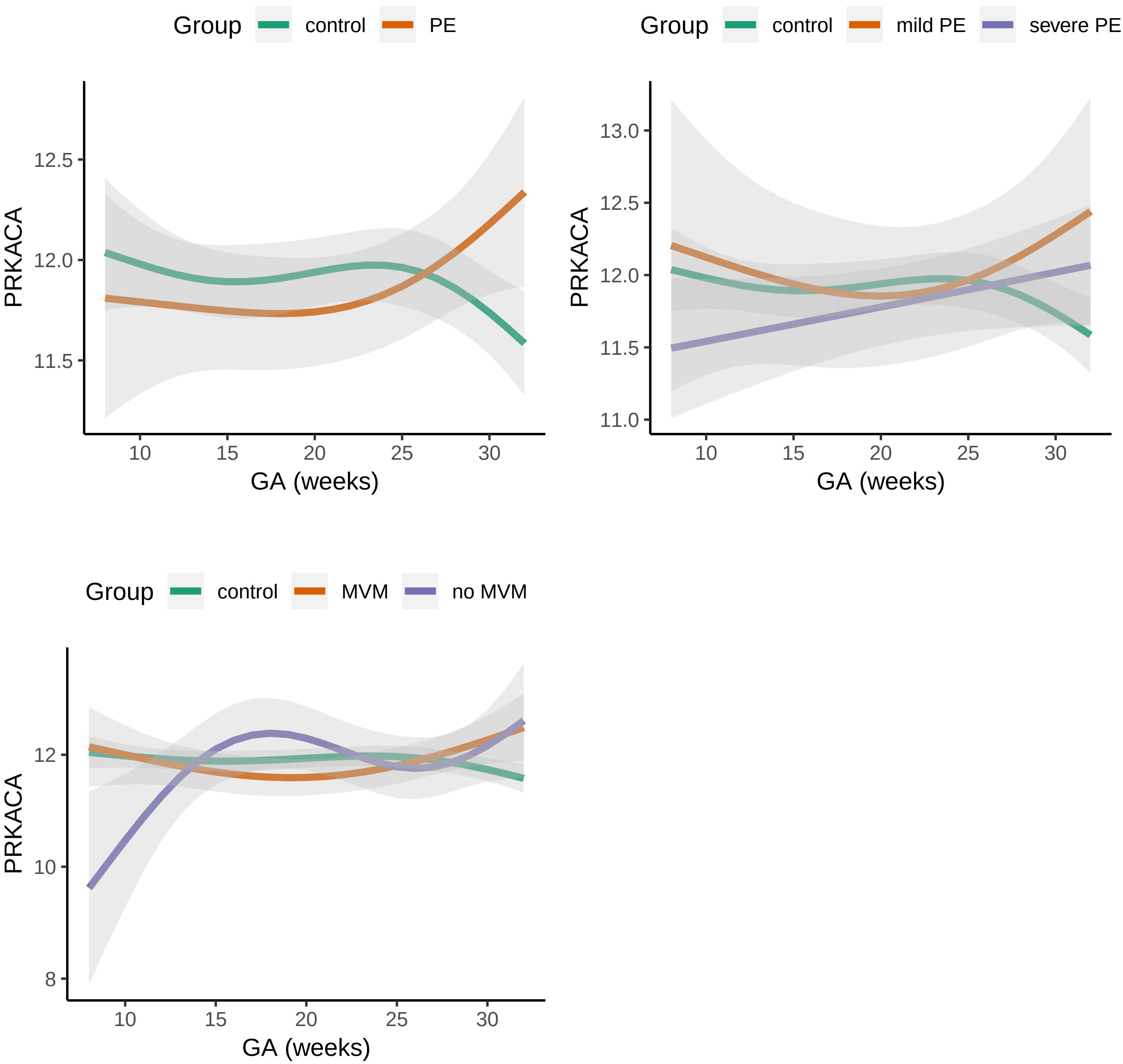
Group
control
PE
PRKACA
12.5
12.0
11.5
10
15
20
25
30
GA (weeks)
Group
control
mild PE
severe PE
PRKACA
13.0
12.5
12.0
11.5
11.0
10
15
20
25
30
GA (weeks)
Group
control
MVM
no MVM
PRKACA
12
10
8
10
15
20
25
30
GA (weeks)

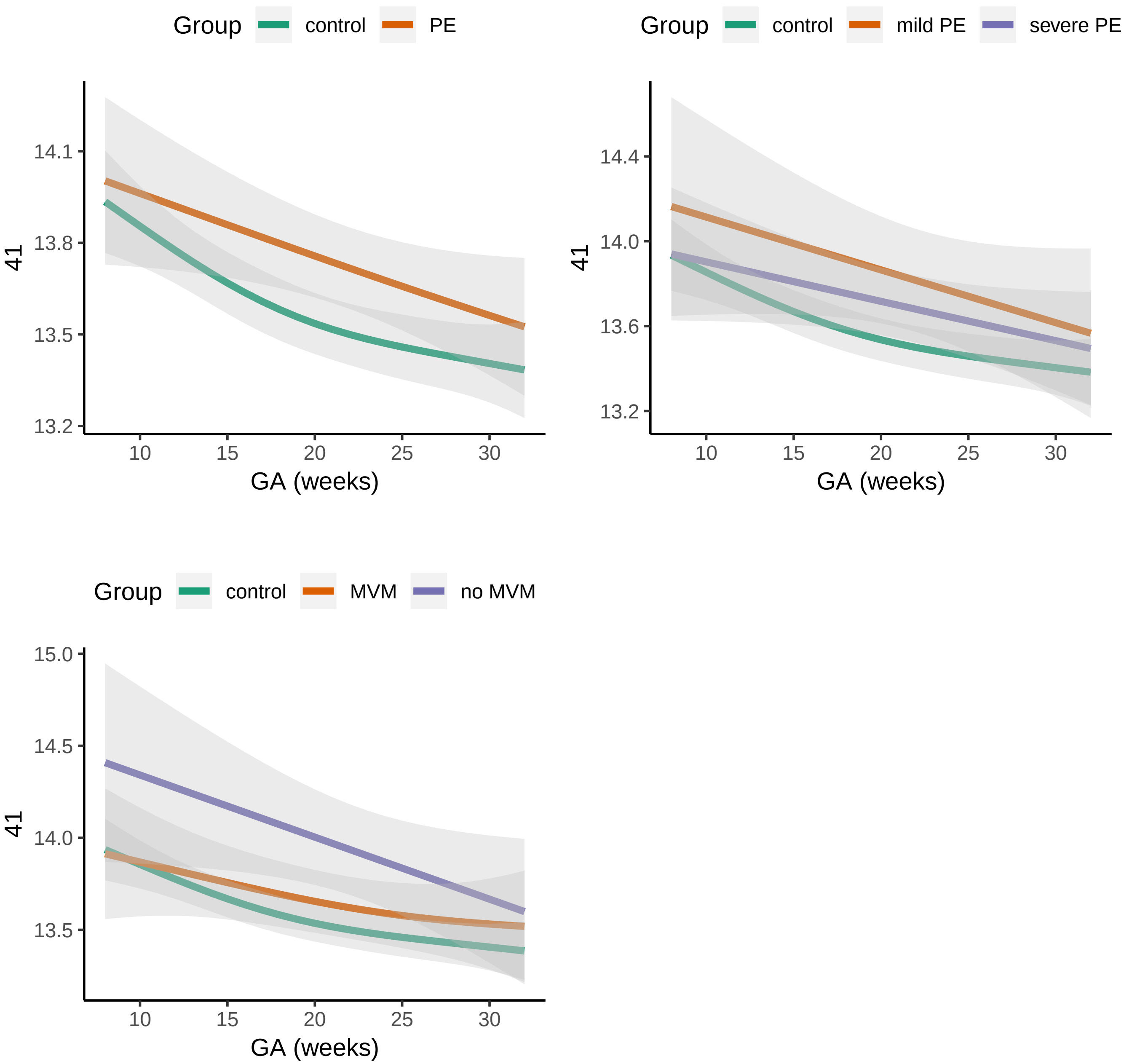

Group control PE
41
14.1
13.8
13.5
13.2
10 15 20 25 30
GA (weeks)
Group control mild PE severe PE
41
14.4
14.0
13.6
13.2
10 15 20 25 30
GA (weeks)
Group control MVM no MVM
41
15.0
14.5
14.0
13.5
10 15 20 25 30
GA (weeks)

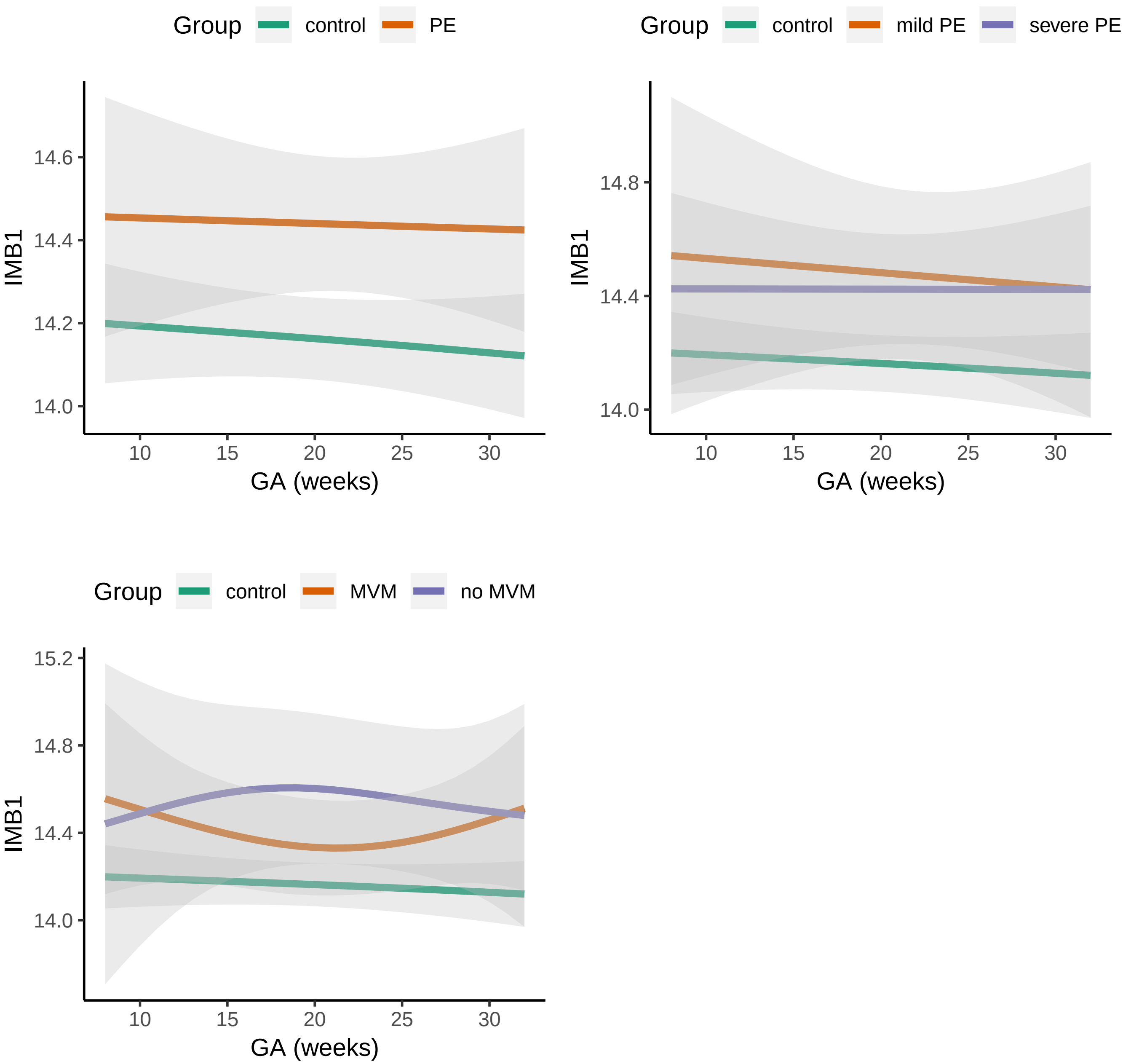
Group
control
PE
IMB1
14.6
14.4
14.2
14.0
10
15
20
25
30
GA (weeks)
Group
control
mild PE
severe PE
IMB1
14.8
14.4
14.0
10
15
20
25
30
GA (weeks)
Group
control
MVM
no MVM
IMB1
15.2
14.8
14.4
14.0
10
15
20
25
30
GA (weeks)

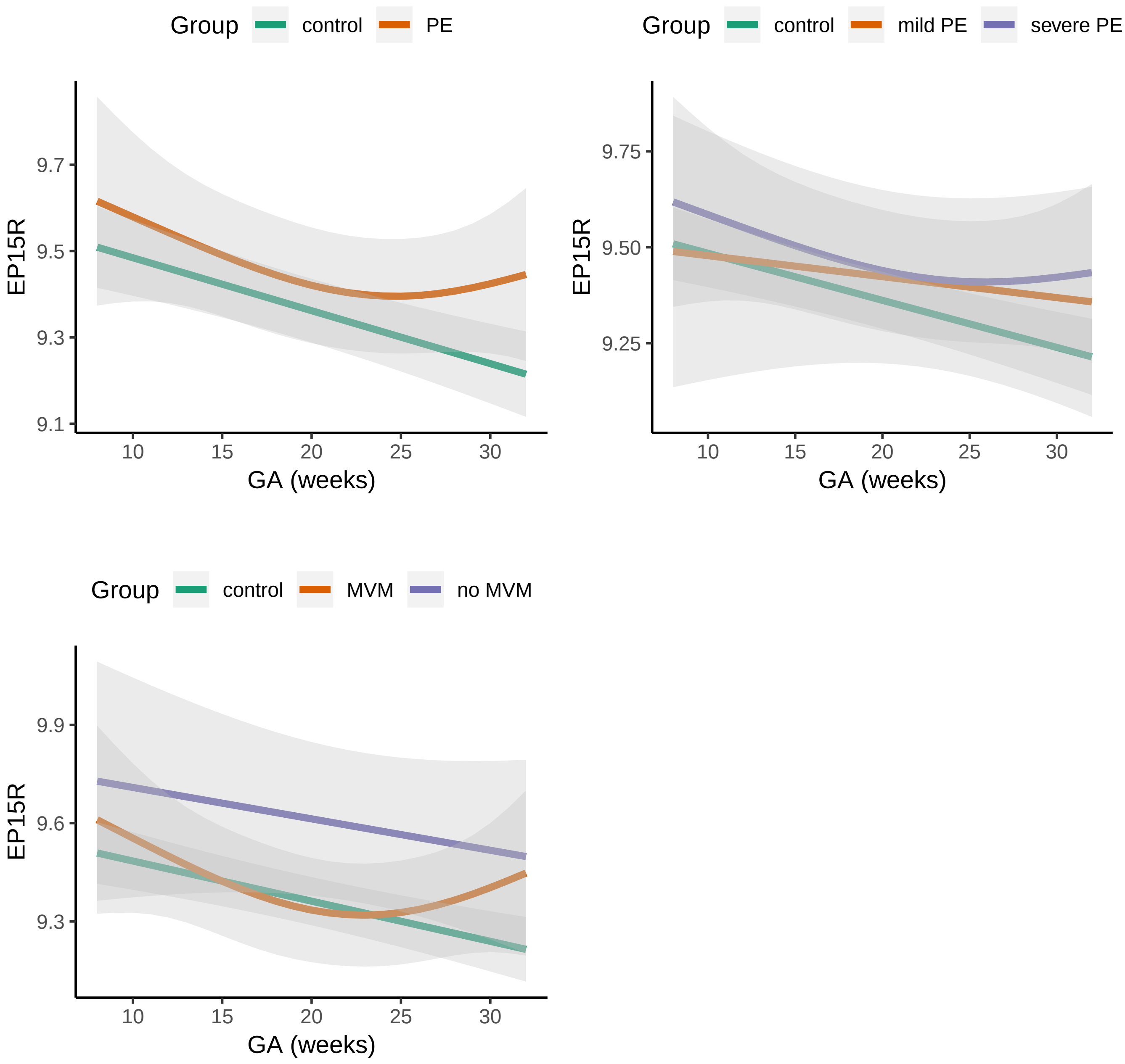

Group
control
PE
EP15R
9.1
9.3
9.5
9.7
10
15
20
25
30
GA (weeks)
Group
control
mild PE
severe PE
EP15R
9.25
9.50
9.75
10
15
20
25
30
GA (weeks)
Group
control
MVM
no MVM
EP15R
9.3
9.6
9.9
10
15
20
25
30
GA (weeks)

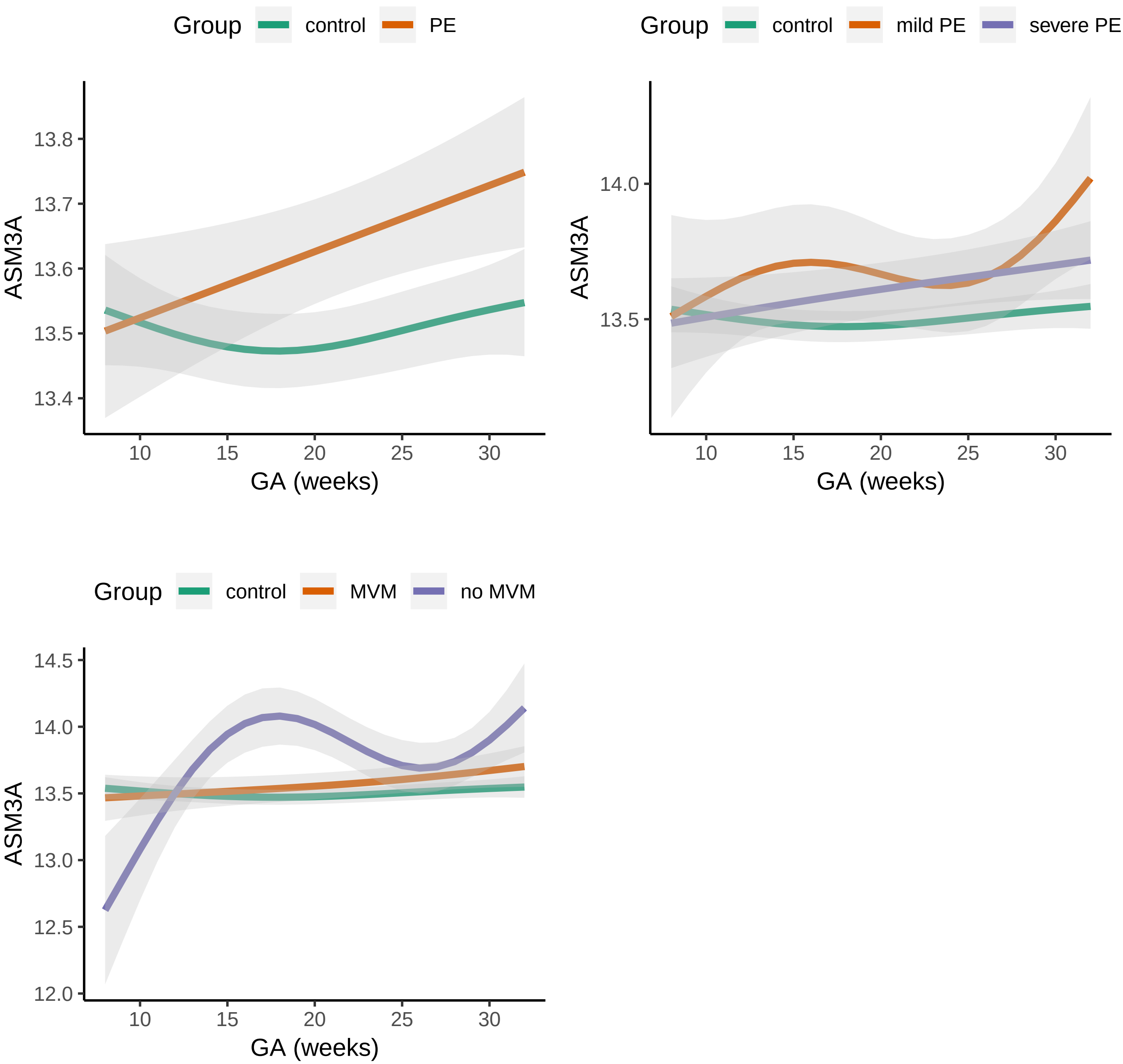

Group
control
PE
ASM3A
13.8
13.7
13.6
13.5
13.4
10
15
20
25
30
GA (weeks)
Group
control
mild PE
severe PE
ASM3A
14.0
13.5
10
15
20
25
30
GA (weeks)
Group
control
MVM
no MVM
ASM3A
14.5
14.0
13.5
13.0
12.5
12.0
10
15
20
25
30
GA (weeks)

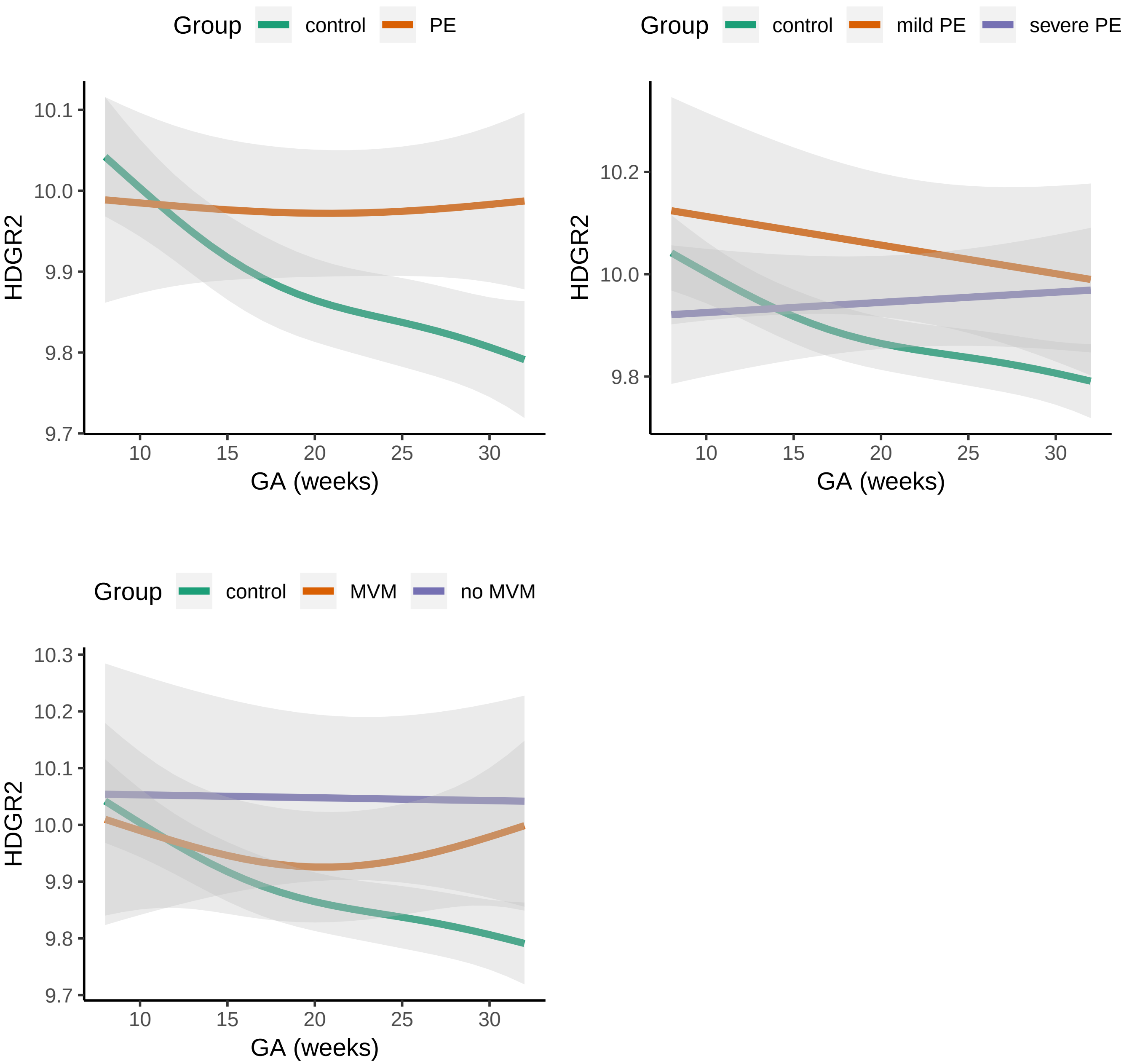
Group
control
PE
HDGR2
GA (weeks)
Group
control
mild PE
severe PE
HDGR2
GA (weeks)
Group
control
MVM
no MVM
HDGR2
GA (weeks)

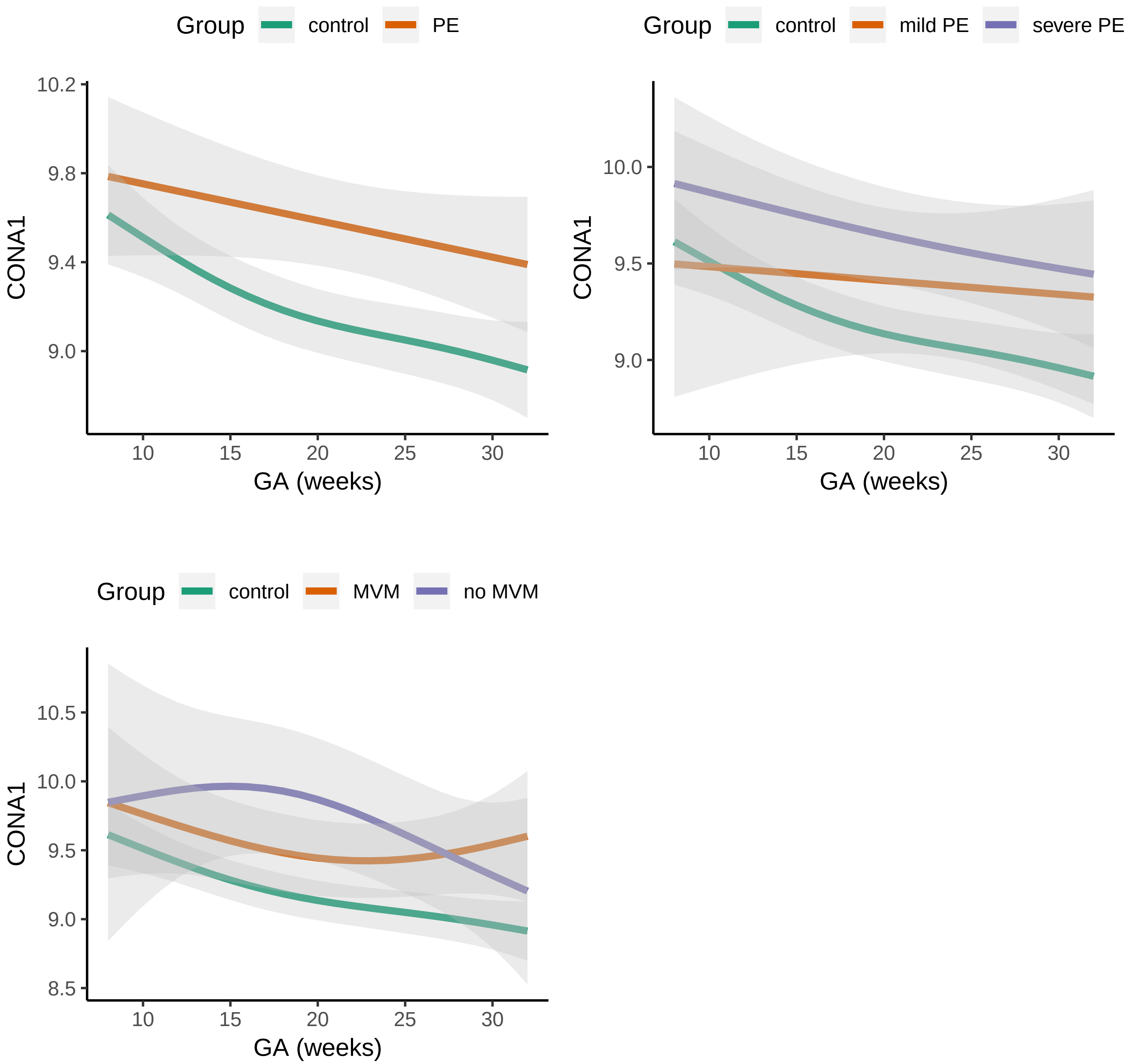
Group
control
PE
10.2
9.8
9.4
9.0
CONA1
10
15
20
25
30
GA (weeks)
Group
control
mild PE
severe PE
10.0
9.5
9.0
CONA1
10
15
20
25
30
GA (weeks)
Group
control
MVM
no MVM
10.5
10.0
9.5
9.0
8.5
CONA1
10
15
20
25
30
GA (weeks)

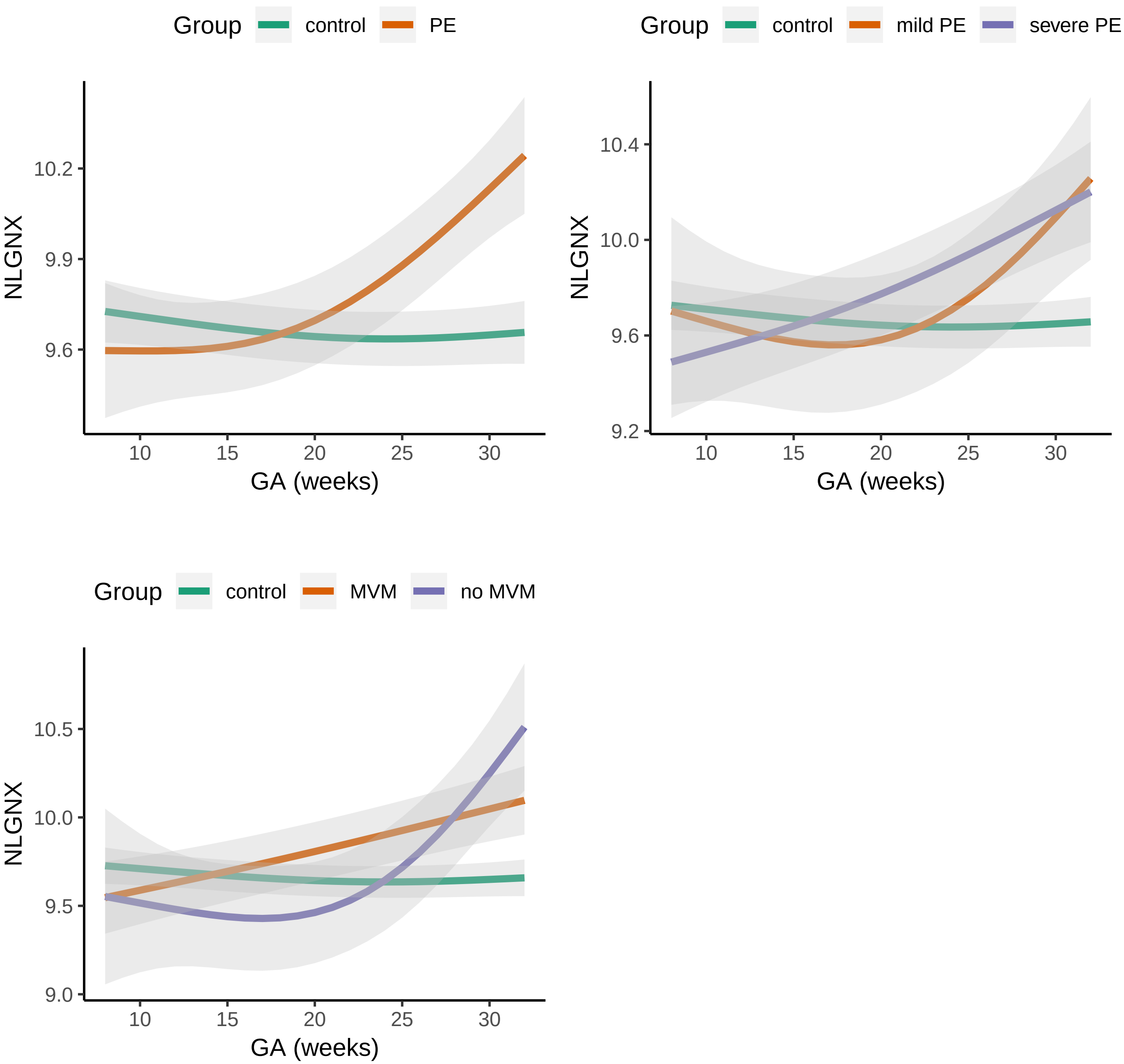
Group
control
PE
NLGNX
10.2
9.9
9.6
10
15
20
25
30
GA (weeks)
Group
control
mild PE
severe PE
NLGNX
10.4
10.0
9.6
9.2
10
15
20
25
30
GA (weeks)
Group
control
MVM
no MVM
NLGNX
10.5
10.0
9.5
9.0
10
15
20
25
30
GA (weeks)

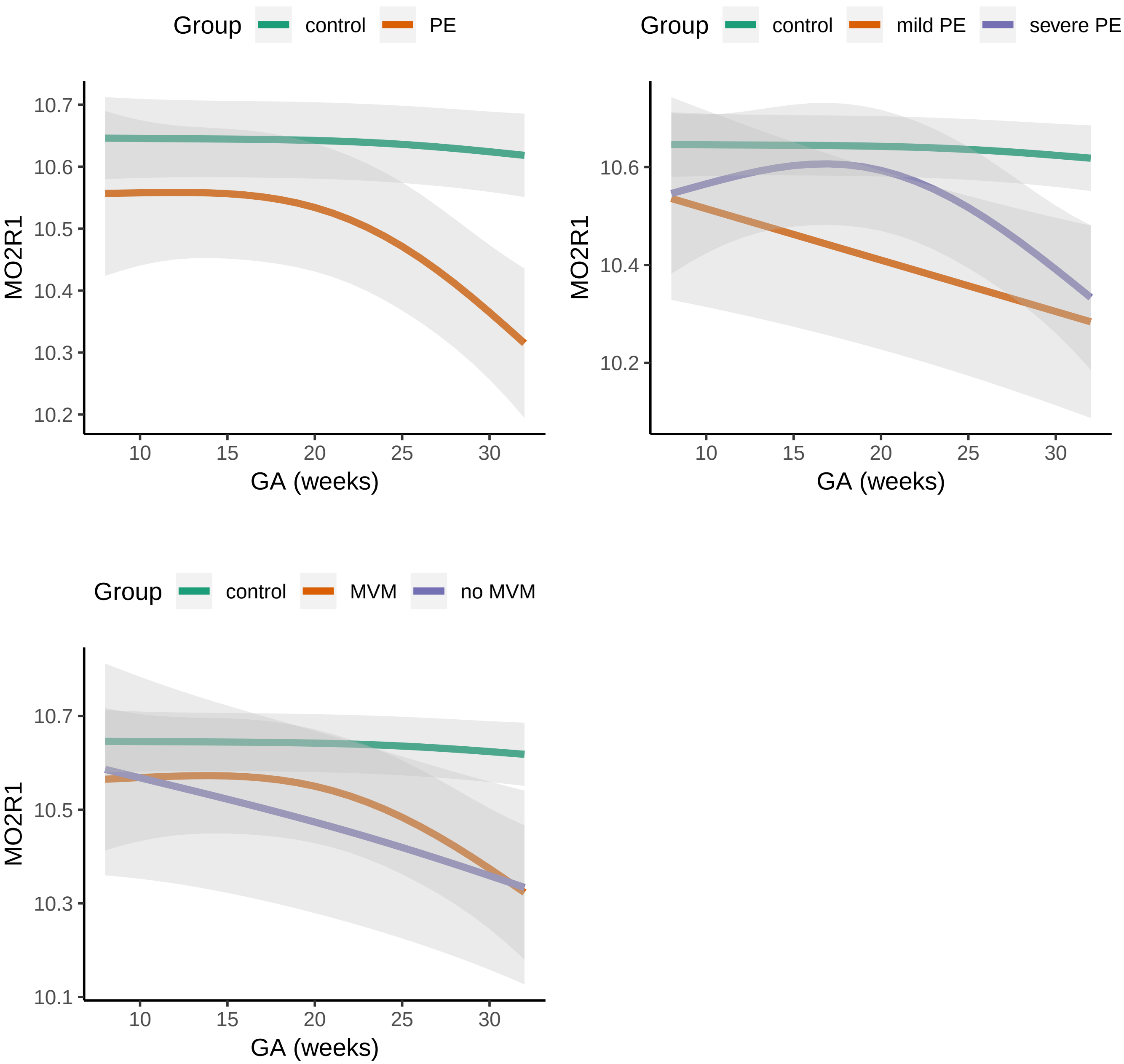

Group
control
PE
MO2R1
10.7
10.6
10.5
10.4
10.3
10.2
10
15
20
25
30
GA (weeks)
Group
control
mild PE
severe PE
MO2R1
10.6
10.4
10.2
GA (weeks)
Group
control
MVM
no MVM
MO2R1
10.7
10.5
10.3
10.1
GA (weeks)

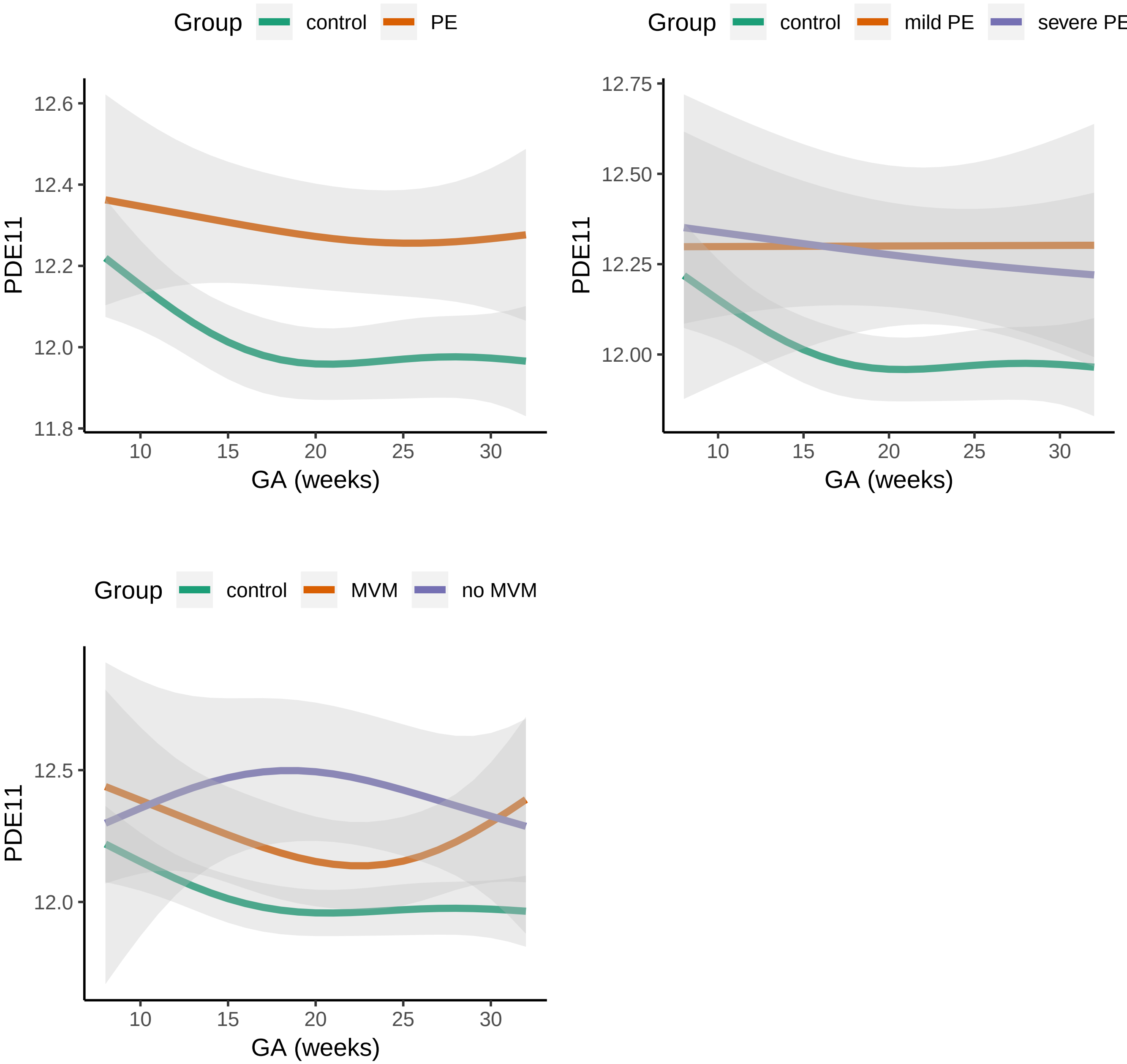

Group
control
PE
12.6
12.4
12.2
12.0
11.8
PDE11
10
15
20
25
30
GA (weeks)
Group
control
mild PE
severe PE
12.75
12.50
12.25
12.00
PDE11
10
15
20
25
30
GA (weeks)
Group
control
MVM
no MVM
12.5
12.0
PDE11
10
15
20
25
30
GA (weeks)

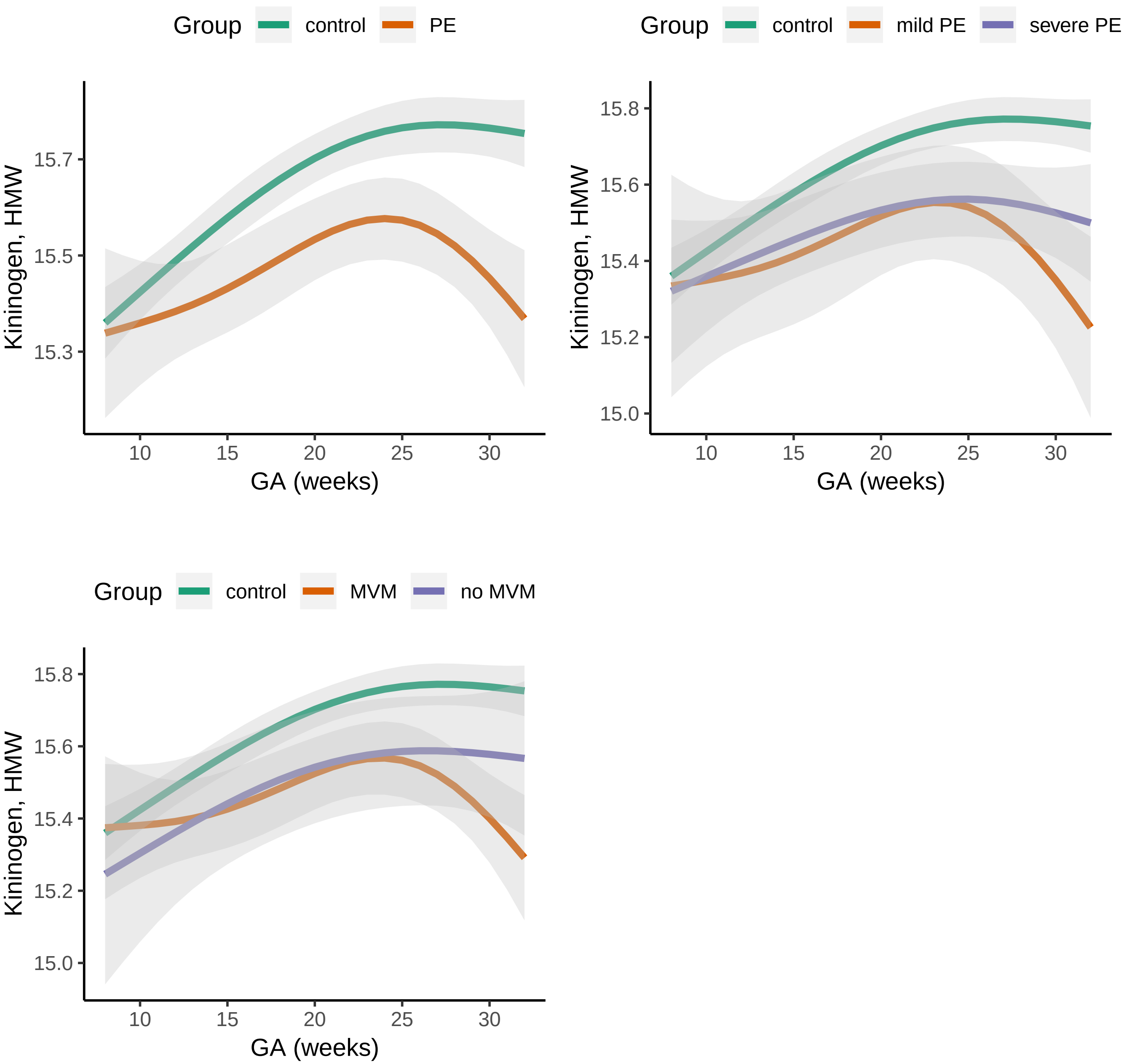

Group
control
PE
Kininogen, HMW
15.3
15.5
15.7
GA (weeks)
10
15
20
25
30
Group
control
mild PE
severe PE
Kininogen, HMW
15.0
15.2
15.4
15.6
15.8
GA (weeks)
Group
control
MVM
no MVM
Kininogen, HMW
GA (weeks)

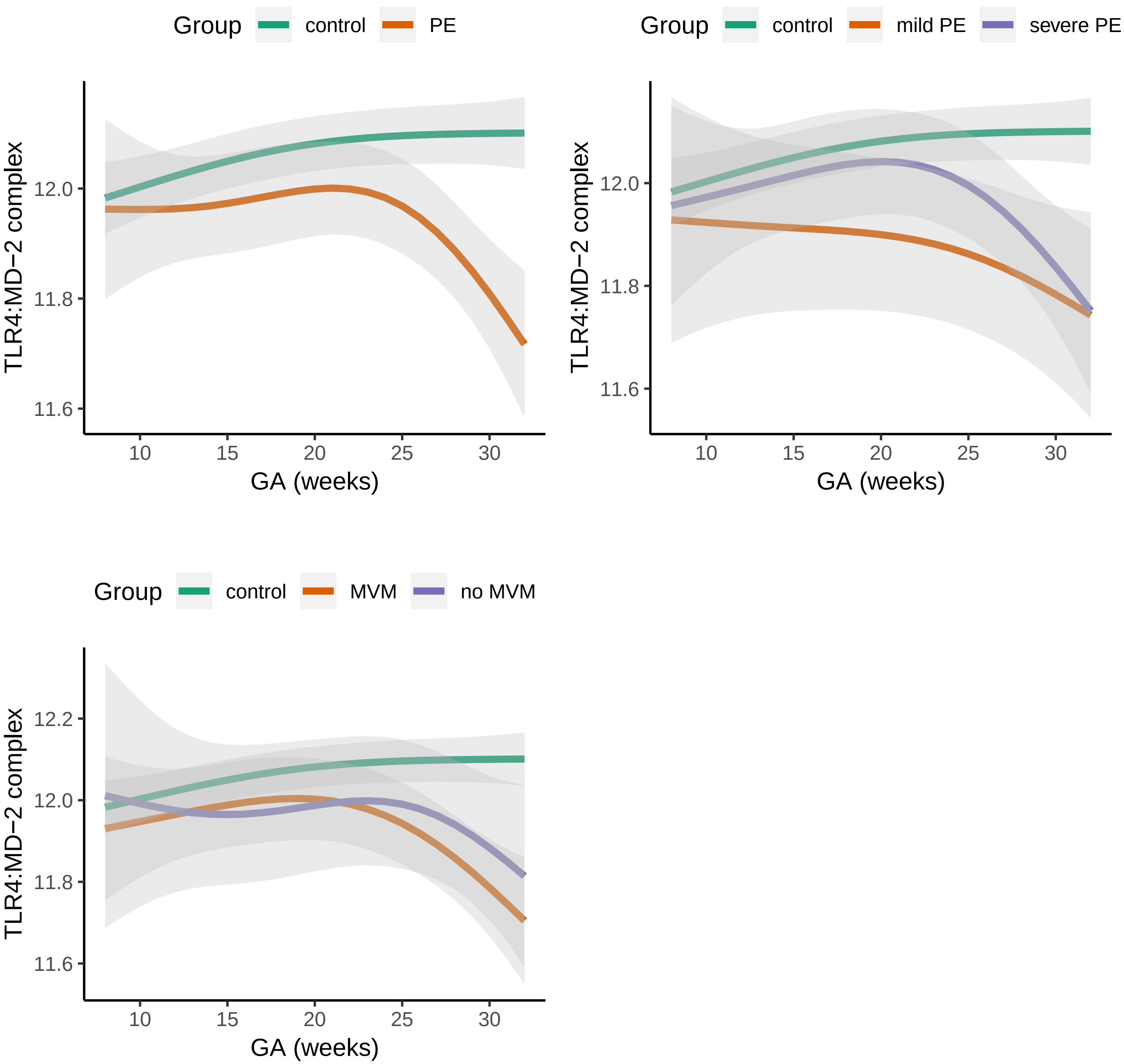
Group
control
PE
TLR4:MD−2 complex
12.0
11.8
11.6
10
15
20
25
30
GA (weeks)
Group
control
mild PE
severe PE
TLR4:MD−2 complex
12.0
11.8
11.6
10
15
20
25
30
GA (weeks)
Group
control
MVM
no MVM
TLR4:MD−2 complex
12.2
12.0
11.8
11.6
10
15
20
25
30
GA (weeks)
